# Supplementary material for: The structural basis of function and regulation of neuronal cotransporters NKCC1 and KCC2
Source: Commun Biol. 2021 Feb 17;4:226. doi: 10.1038/s42003-021-01750-w (PMC7889885; doi:10.1038/s42003-021-01750-w)
Supplement: Supplementary file 2 — Supplementary Information [file 42003_2021_1750_MOESM2_ESM.pdf]

The structural basis of function and regulation of neuronal cotransporters NKCC1 and KCC2

Sensen Zhang[1,*], Jun Zhou[1,*], Yuebin Zhang[2,*], Tianya Liu[1,*], Perrine Friedel[3,*], Wei Zhuo[1], Suma Somasekharan[3], Kasturi Roy[3], Laixing Zhang[1], Yang Liu[1], Xianbin Meng[4], Haiteng Deng[4], Wenwen Zeng[5], Guohui Li[2,#], Biff Forbush[3,#], Maojun Yang[1,6,#]

*These authors contributed equally to this work.

#To whom correspondence should be addressed:

Guohui Li: ghli@dicp.ac.cn

Biff Forbush: biff.forbush@yale.edu

Maojun Yang: maojunyang@tsinghua.edu.cn

This PDF file includes:

Supplementary Figs 1 - 21 and Supplementary Note 1.

Supplementary Figs 19 - 21 are unprocessed original blot and gel images.

Supplementary Figures and Figure legends

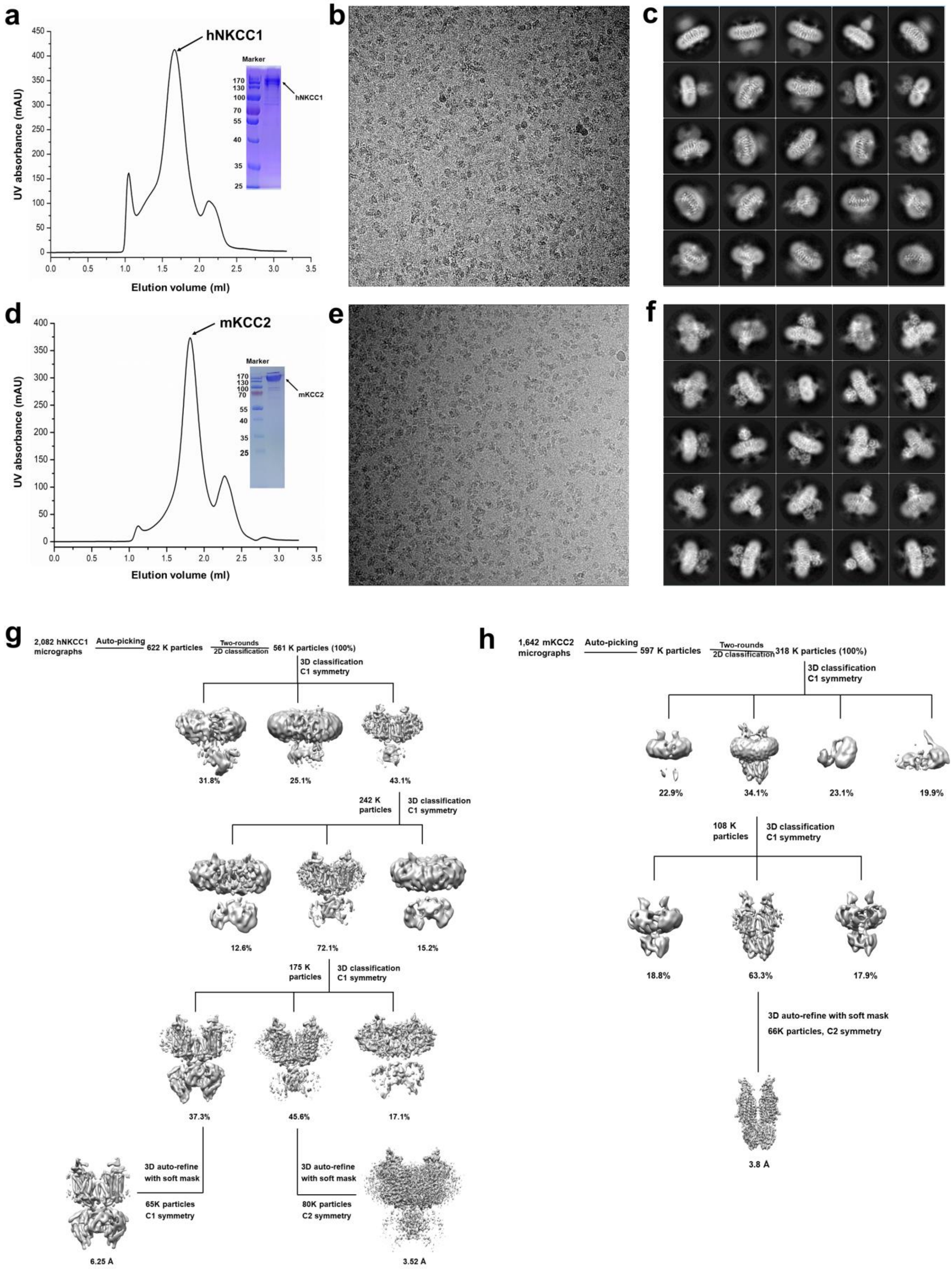

Supplementary Fig. 1. Protein purification and structure determination of hNKCC1 and mKCC2.

a. Size-exclusion chromatography of hNKCC1 in digitonin buffer. The peak corresponding to the hNKCC1 dimer (indicated) was subjected to SDS-PAGE and Coomassie blue staining.
b. Representative cryo-EM micrograph of hNKCC1 in digitonin buffer.
c. 2D class averages of the hNKCC1 sample in digitonin buffer.
d. Size-exclusion chromatography of mKCC2 in digitonin buffer. The peak corresponding to the mKCC2 dimer (indicated) was subjected to SDS-PAGE and Coomassie blue staining.
e. Representative cryo-EM micrograph of mKCC2 in digitonin buffer.
f. 2D class averages of mKCC2 sample in digitonin buffer.
g. The workflow of 2D/3D reconstruction with hNKCC1 cryo-EM data. In brief, 561 k particles were kept after 2D classification, and subjected to three rounds of 3D classification. A final dataset containing 80 k particles were used for high-resolution refinement (see methods for more details).
h. The workflow of 2D/3D reconstruction with mKCC2 cryo-EM data. In brief, 318 k particles were kept after 2D classification, and subjected to two rounds of 3D classification. A final dataset containing 66 k particles were used for high-resolution refinement (see methods for more details).

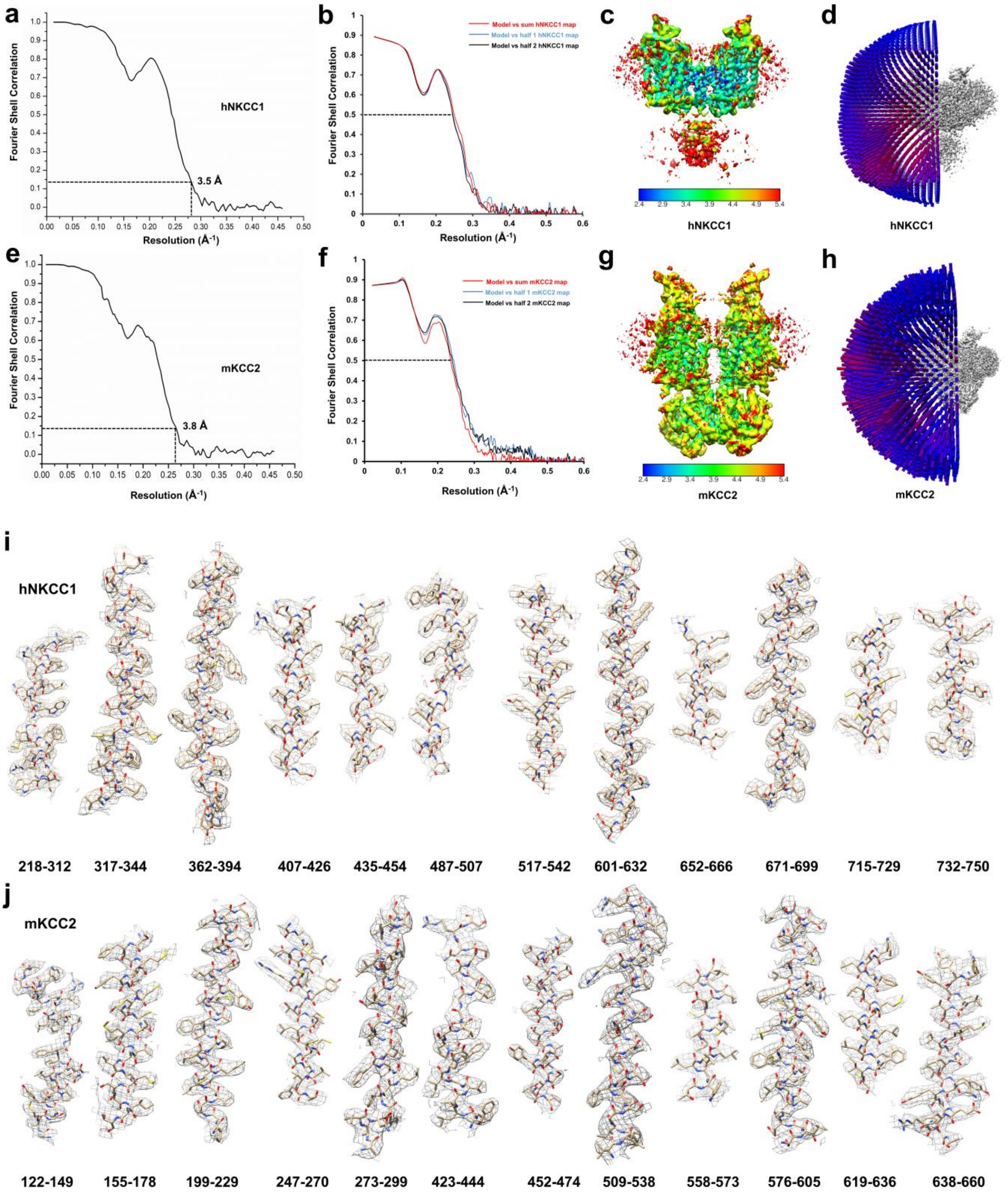

Supplementary Fig. 2. Reconstruction and representative cryo-EM densities of hNKCC1 and mKCC2

a. Gold-standard Fourier Shell correlation (FSC) curve of hNKCC1 after 3D refinement. The resolution estimation was based on the criterion of FSC 0.143 cutoff.

b. Cross-validation of the atomic model with the summed map and the half maps of hNKCC1.

c. Local resolution map of the hNKCC1 after the final 3D density map (3.5 Å).

d. Angular distribution of the hNKCC1 final reconstruction.

e. Gold-standard Fourier Shell correlation (FSC) curve of mKCC2 after 3D refinement similar to the panel (a).

f. Cross-validation of the atomic model with the summed map and the half maps of mKCC2.

g. Local resolution map of the mKCC2 after the final 3D density map (3.8 Å).

h. Angular distribution of the mKCC2 final reconstruction.

i. Density maps of representative transmembrane regions of hNKCC1. Stick style atomic models (gold) were fitted into the cryo-EM density maps (gray mesh). The density maps were contoured at 10.0 σ

j. Density maps of representative transmembrane regions of mKCC2, similar to the panel (i).

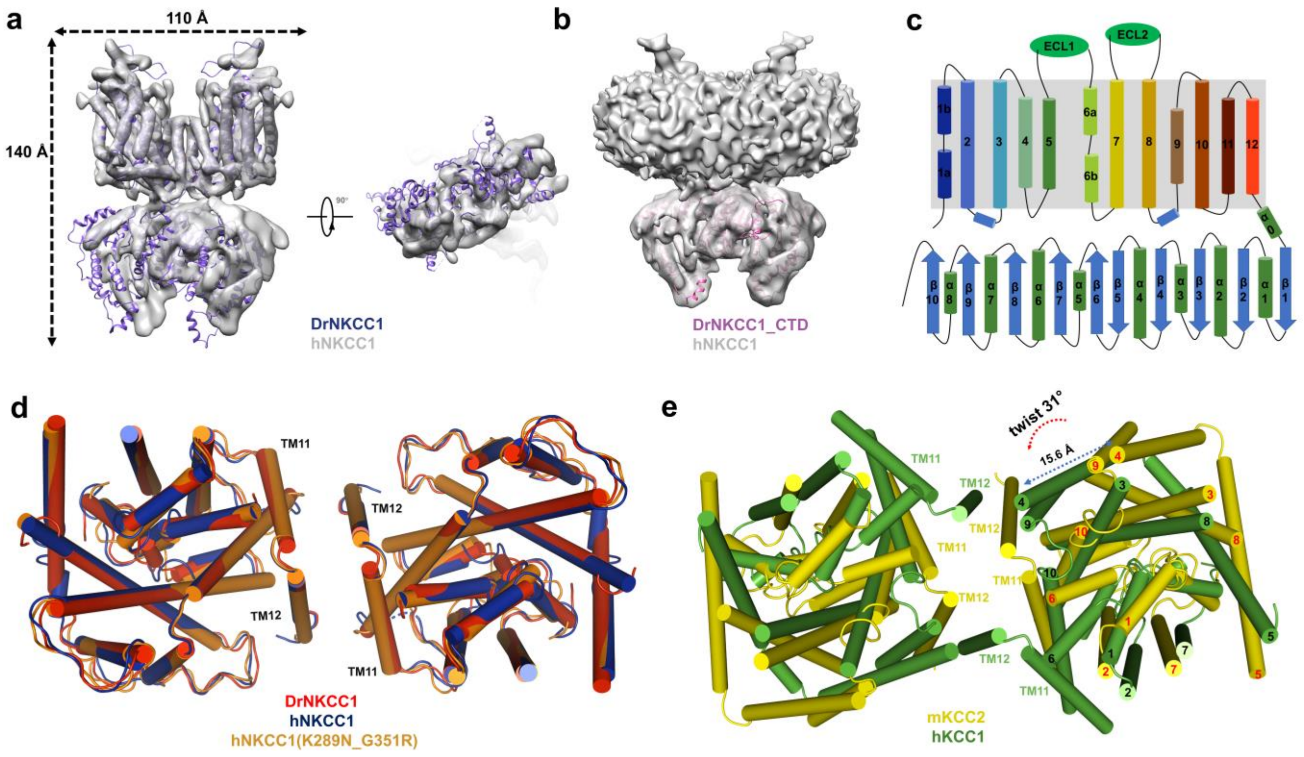

Supplementary Fig. 3. Structure of the hNKCC1 and mKCC2 transporters.

a. Structural comparisons between hNKCC1 medium resolution cryo-EM map (gray) and DrNKCC1 model (blue) aligned by the transmembrane regions, shown from the side view and bottom view.

b. Structural comparisons between hNKCC1 medium resolution cryo-EM map (gray) and DrNKCC1 CTD model (purple) aligned by the CTD regions, shown from the side view.

c. Secondary structure topology of mKCC2 with α helices was shown as cylinders and β sheets

shown as arrows.

d. The TM-domain of the DrNKCC1 (red), previous reported hNKCC1(K289N_G351R) (brown), and the current hNKCC1 (blue) dimers are superimposed. Top view (from the extracellular side).

e. Superimposition of hKCC1 (green) and mKCC2 (yellow) dimers highlights different dimer interfaces -- the dimer interface of hKCC1 is displaced by a 15.6 Å translation 31° rotation relative to that of mKCC2.

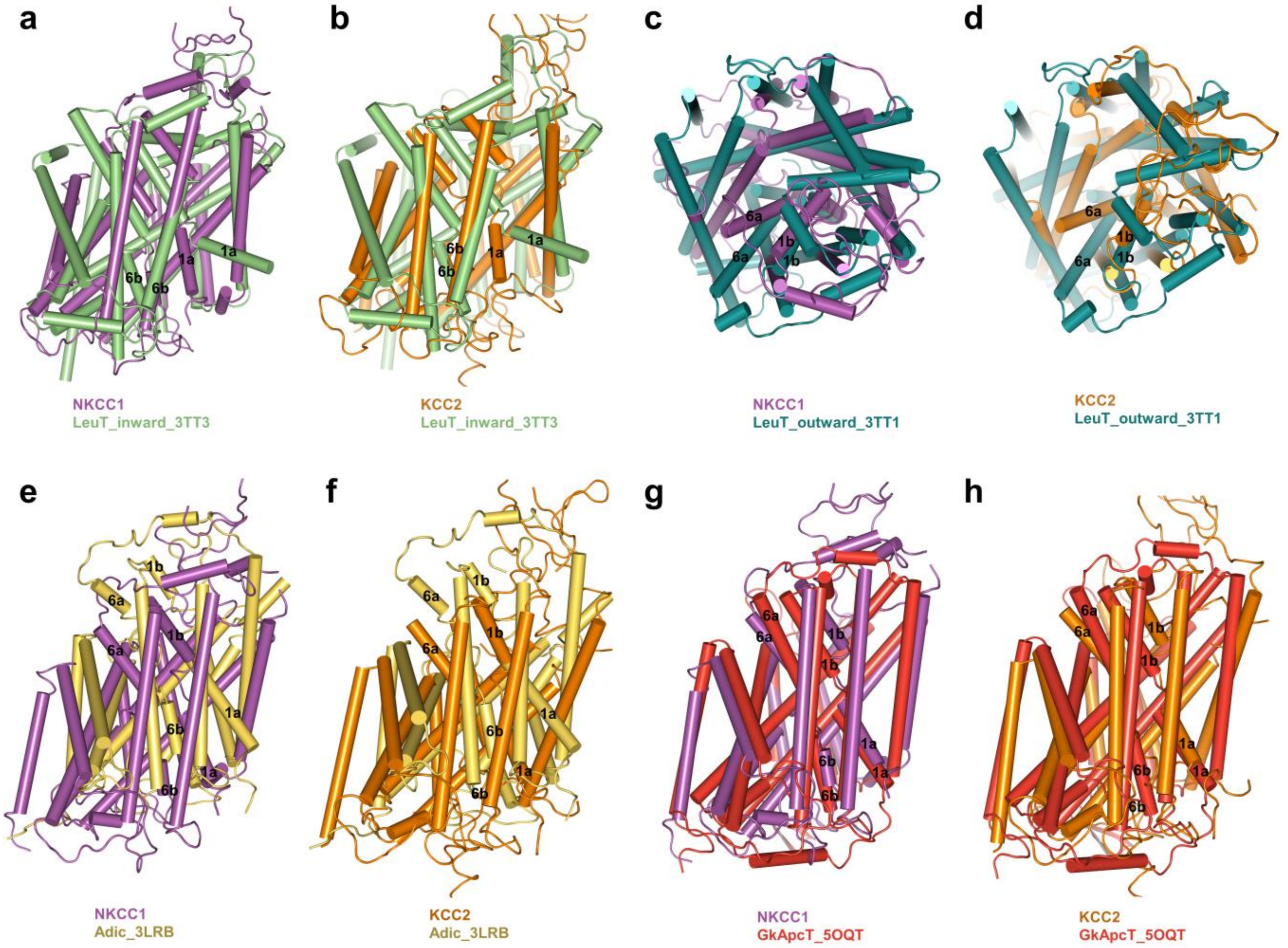

Supplementary Fig. 4. Comparisons of hNKCC1, mKCC2 with other homologous proteins

a-b. Comparison of hNKCC1, mKCC2 with LeuT inward conformation (PDB_code_3TT3).

c-d. Comparison of hNKCC1, mKCC2 with LeuT outward conformation (PDB_code_3TT1).

e-f. Comparison of hNKCC1, mKCC2 with Adic structure (PDB_code_3LRB).

g-h. Comparison of hNKCC1, mKCC2 with GkApcT structure (PDB_code_5OQT).

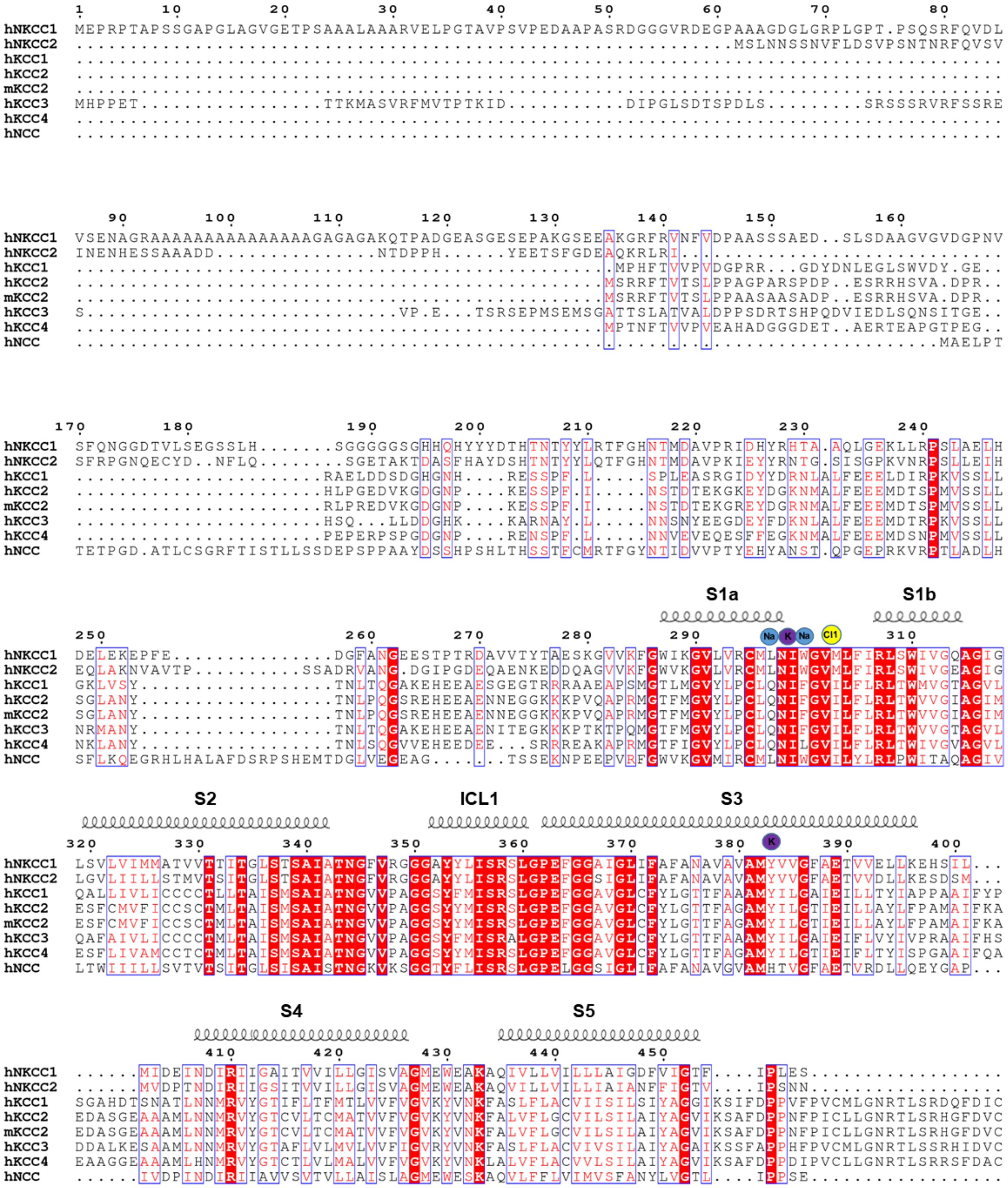
hNKCC1
hNKCC2
hKCC1
hKCC2
mKCC2
hKCC3
hKCC4
hNCC
S1a
S1b
S2
ICL1
S3
S4
S5
Na
K
Cl1

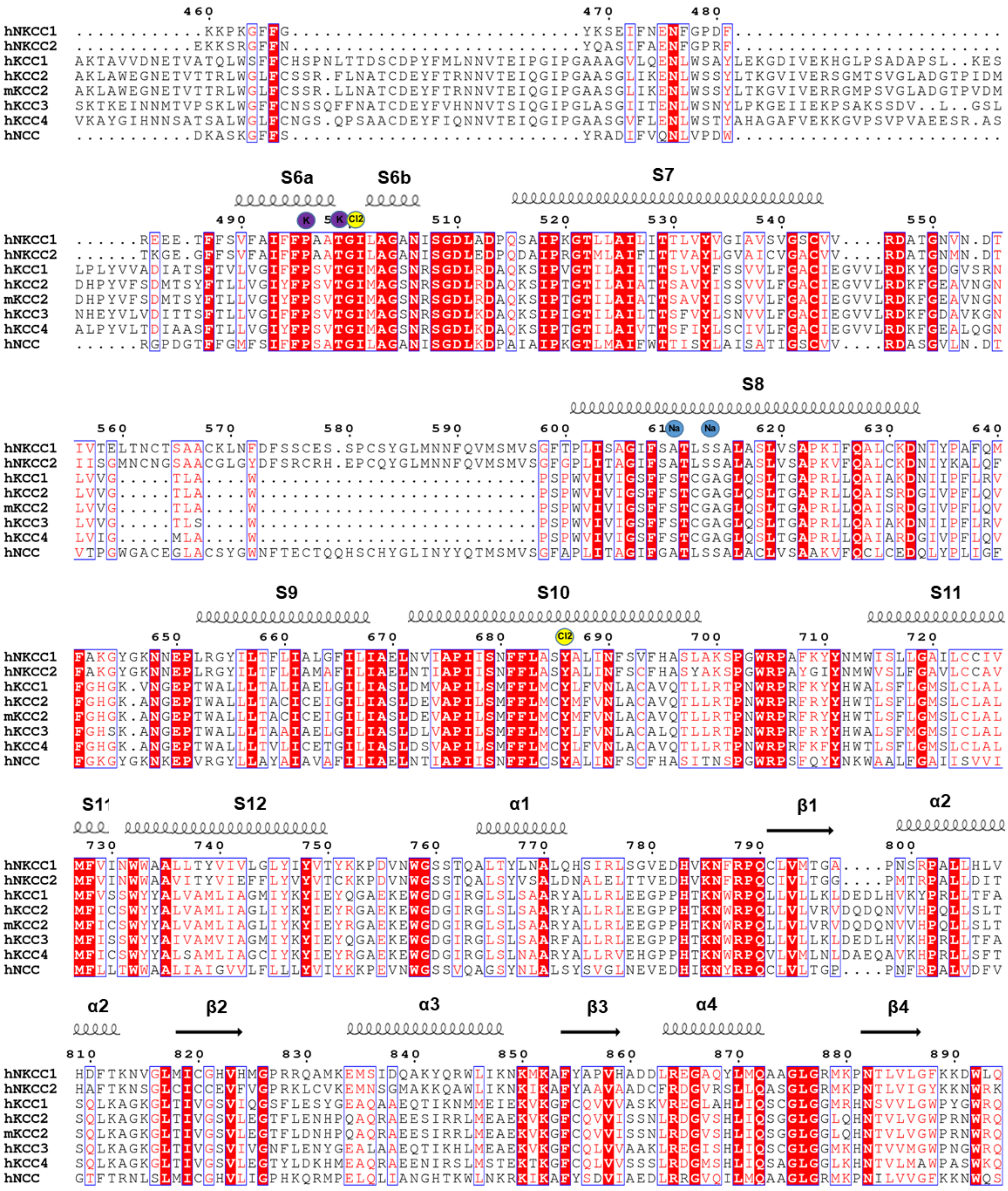
hNKCC1
hNKCC2
hKCC1
hKCC2
mKCC2
hKCC3
hKCC4
hNCC
S6a
S6b
S7
S8
S9
S10
S11
S12
α1
β1
α2
β2
α3
β3
α4
β4
K
Cl2
Na

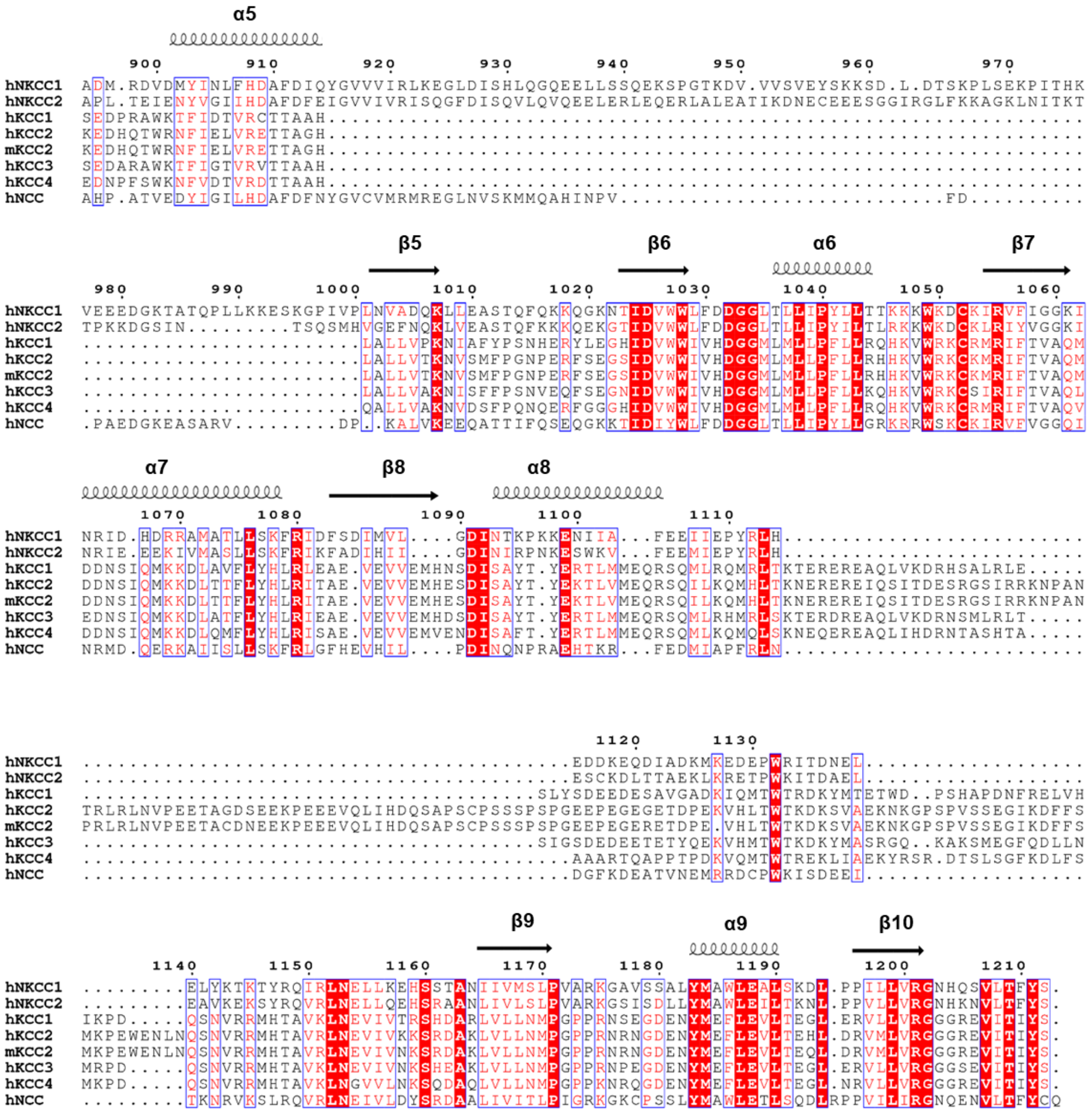

Supplementary Fig. 5. Sequence alignment of cation chloride cotransporters.

Sequence alignment of human NKCC1, human NKCC2, human KCC1, human KCC2, mouse KCC2, human KCC3, human KCC4, and human NCC using ESPript3. Secondary structure elements are indicated above the sequence, shown as arrow (β sheet), helix (α helix). Amino acids proposed to be involved in ion binding are also indicated.

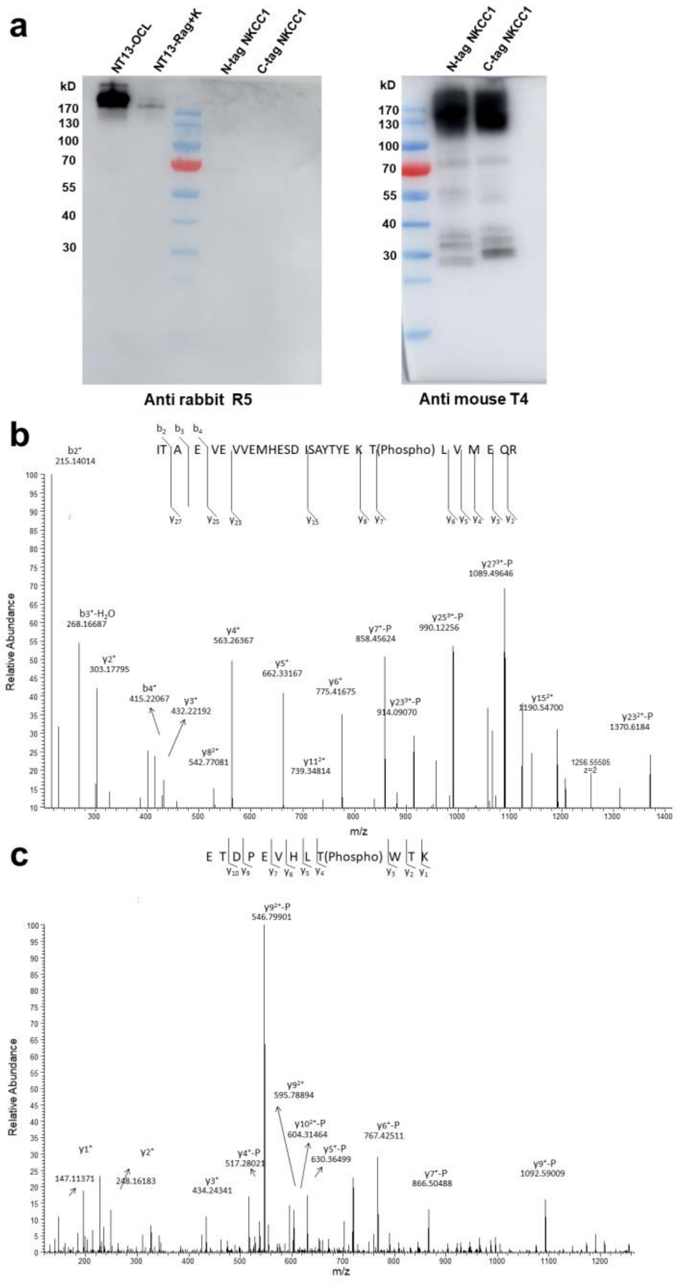

Supplementary Fig. 6. Inactive state identification of hNKCC1 and mKCC2 by western-blotting and mass spectrometry analysis.

a. Western-blotting of hNKCC1 using rabbit R5 and mouse T4 antibody to identify the phosphorylation state of hNKCC1.

b-c: Identification of mKCC2 T929 (b) and T1029 (c) phosphorylation by liquid chromatography mass spectrometry (LC-MS/MS) analysis. The MS/MS spectrum of the phosphorylated peptides ITAEVEVVEMHESDISAYTYEKT(phospho)LVMEQR (b) and ETDPEVHLT(phospho)WTK (c) are shown.

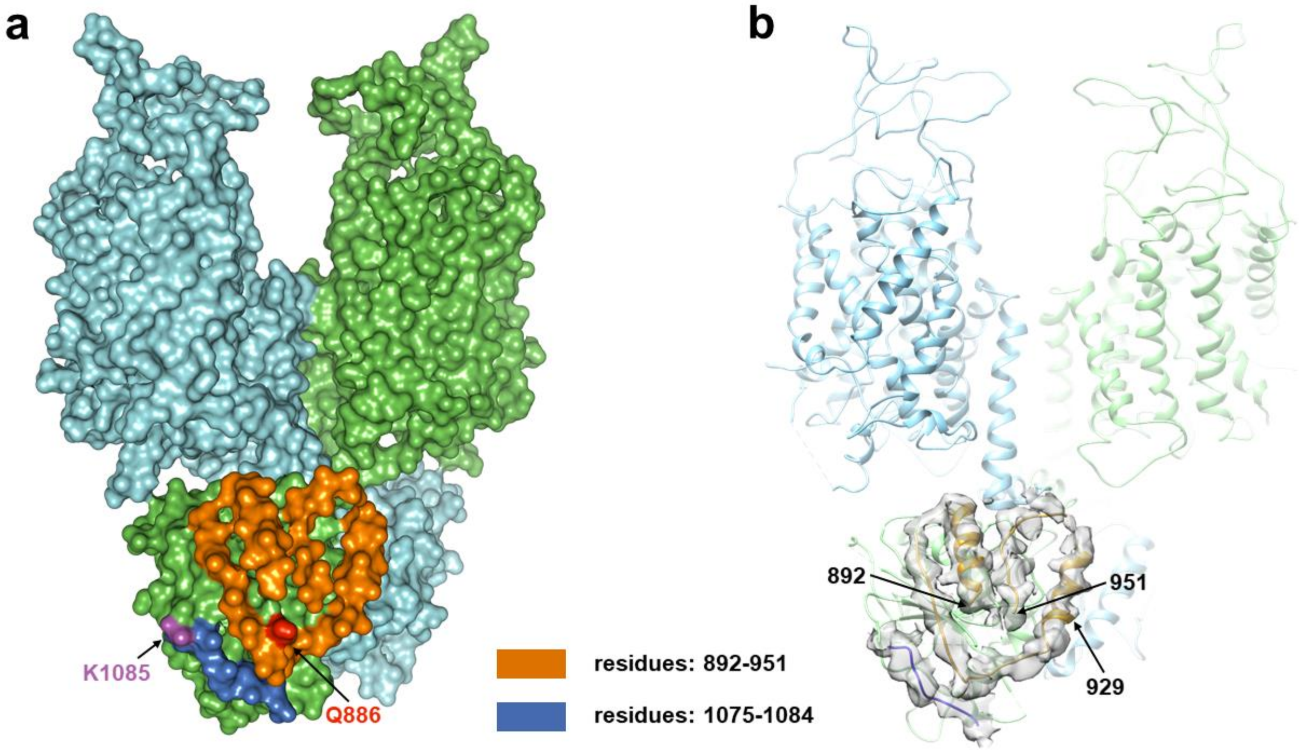

Supplementary Fig. 7. Approximate phosphorylation sites in mKCC2

a. Surface representation of mKCC2 with each monomer colored (blue and green). Residue K1085 and Q886 were labeled in purple and red, respectively. In addition, the approximate location of residues (892-951) and residues (1075-1084) were labelled in brown and blue, respectively.

b. Cryo-EM density map of the representative amino acids around phosphorylation sites. The approximate location of T929 was labeled.

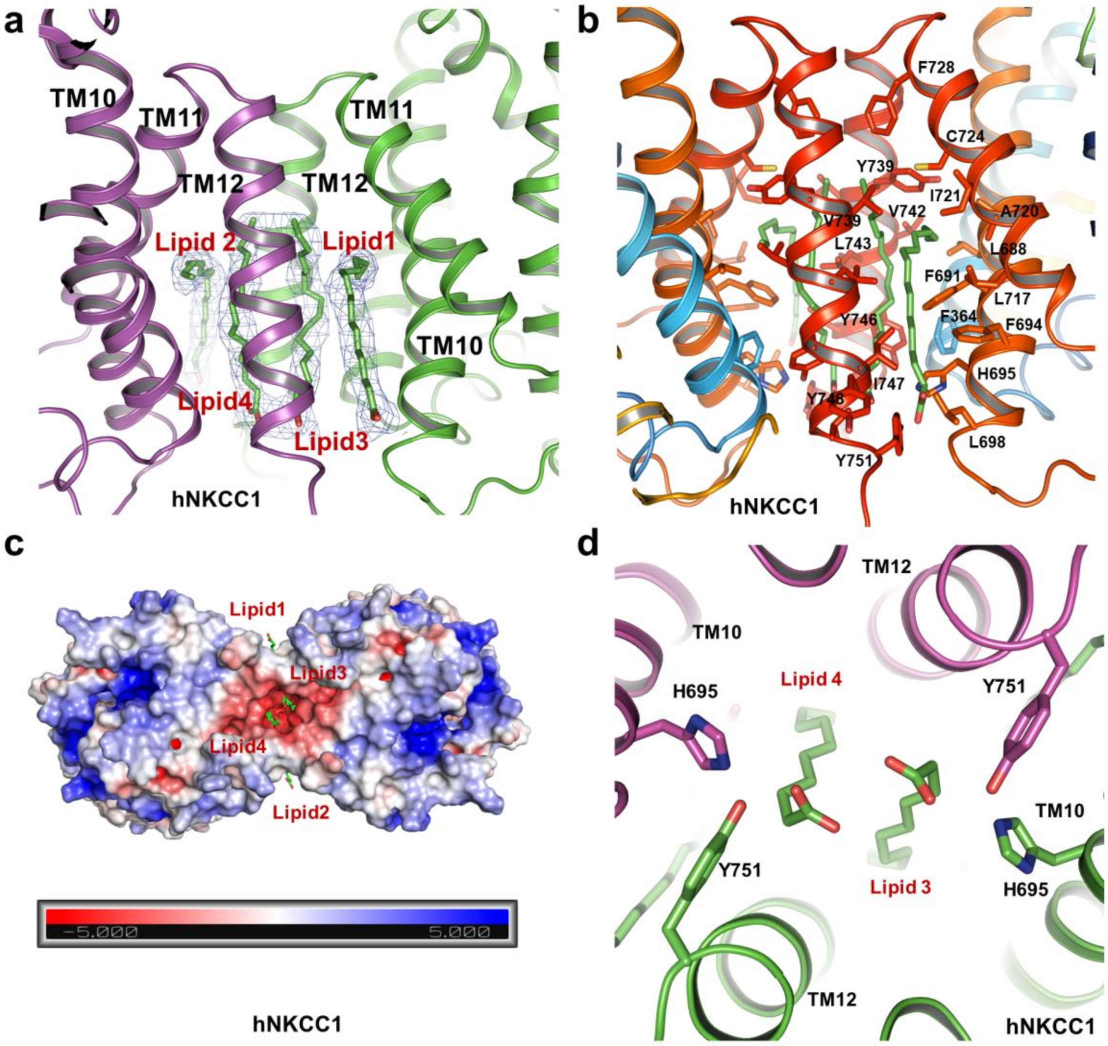

Supplementary Fig. 8. Lipids in the hNKCC1.

a. Four lipids were assigned in the hydrophobic cleft between TM10, TM11, and TM12 of hNKCC1. Lipid molecules were represented with a ribbon model fitted into the blue mesh cryo-EM density (7.0 σ).

b. Hydrophobic amino acids in TM10, TM11, and TM12 interact with four lipids to stabilize the dimeric interface.

c. The bottom view of the surface electrostatic potential of hNKCC1 with lipid molecules showed as ribbon models.

d. The hydrophilic group of Lipid 3 and Lipid 4 interact with adjacent H695 from TM10 and Y751 from TM12.

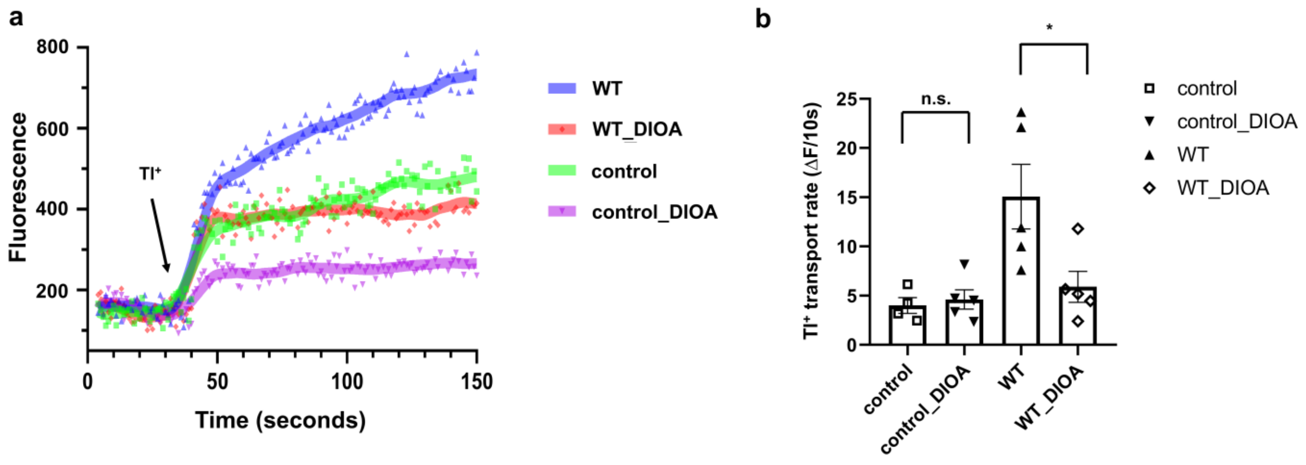

Supplementary Fig. 9. Thallium influx activity mediated by mKCC2.

a. Fluorescence signal measurement between WT, WT_DIOA, control, and control_DIOA. Transport activity was initiated by the Tl^+ addition (arrow indicated).

b. Relative Tl^+ transport rate in WT mKCC2 and control cells with the inhibition by DIOA with dots plotted into the bar graph. DIOA inhibition was evaluated using the unpaired two-tailed Student's *t* test. n.s. = not significant (control, n=5) and $P<0.05$ (WT, n=5); mean ± SEM.

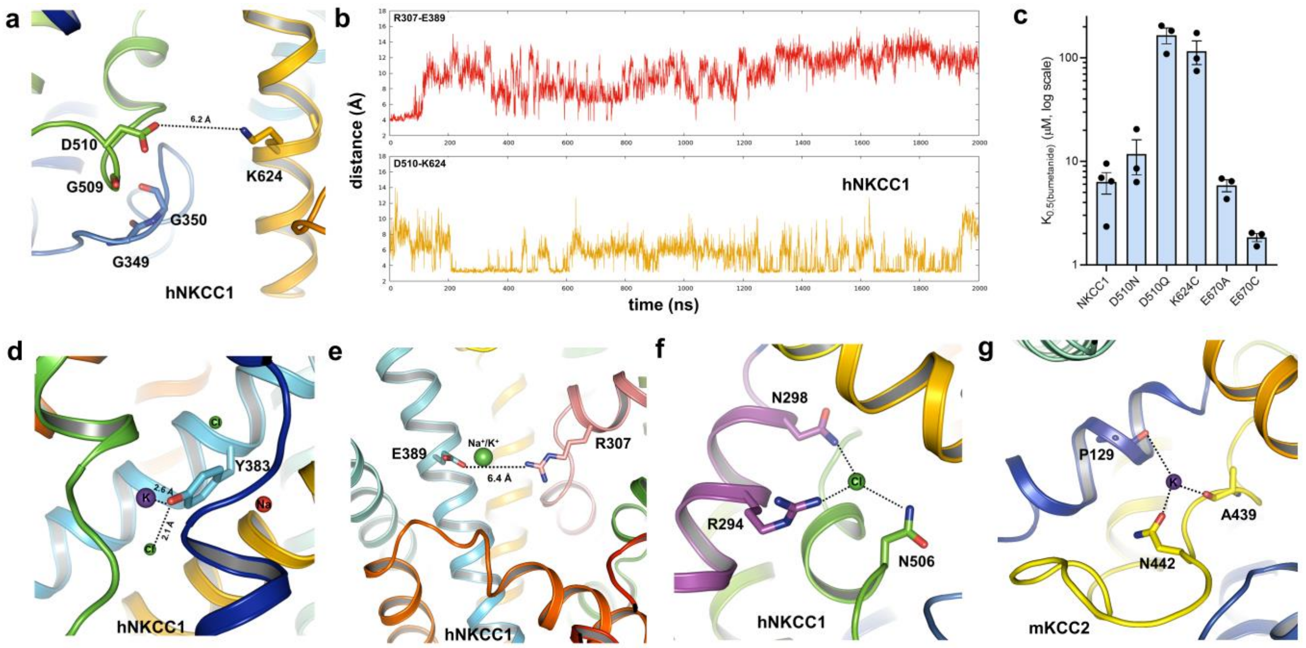

Supplementary Fig. 10. MD simulation studies of hNKCC1 and mKCC2 and bumetanide affinity of hNKCC1 mutants.

a. Main-chain carbonyls of G349, G350, G509 and side chain of D510, play important roles in modulating cation permeation in hNKCC1. In the resolved hNKCC1, the distance between D510 and K624 is approximately 6.2 Å.

b. Representative distance change between R307-E389 and D510-K624 in a 2000 ns MD simulation. From 200-400 ns the distance between D510 and K624 is close enough to form a salt bridge, while the distance between R307 and E389 is large (6-12 Å).

c. Inhibitory $K_{0.5}$ for bumetanide in inhibiting wild type NKCC1 and mutants D510, K624, and E670. Cl- influx into transfected HEK cells was measured at 8 concentrations of bumetanide from 0 to 250 μM, and $K_{0.5(bumetanide)}$ was determined by least squares fit to a single-site model. Values are mean ± SEM from 3-4 experiments. Note that in these experiments bumetanide was present only in the influx medium and $K_{0.5}$ values are much higher (roughly 10 fold for wild type) than values reported when bumetanide is included in an optimal-binding pre-incubation medium.

d. In some MD simulations, the hydroxyl group of Y383 plays a bifunctional role in coordinating the K^+ and Cl^-. The distance between the hydroxyl and the K^+ is 2.6 Å, while the distance from the Cl^- is about 2.1 Å.

e. The salt bridge interaction contributed by E389 and R307 could be replaced by Na^+ or K^+ binding in some of the well-tempered metadynamics simulations.

f. In hNKCC1, a dominant Cl^--binding cavity was located at the intracellular region contributed by the side chains of R294, N298, and N506.

g. In mKCC2, the second major proposed K^+ binding site was located at the intercellular entrance of the tunnel, constituted by the backbone carbonyls of P129, A439 and the side chain of N442.

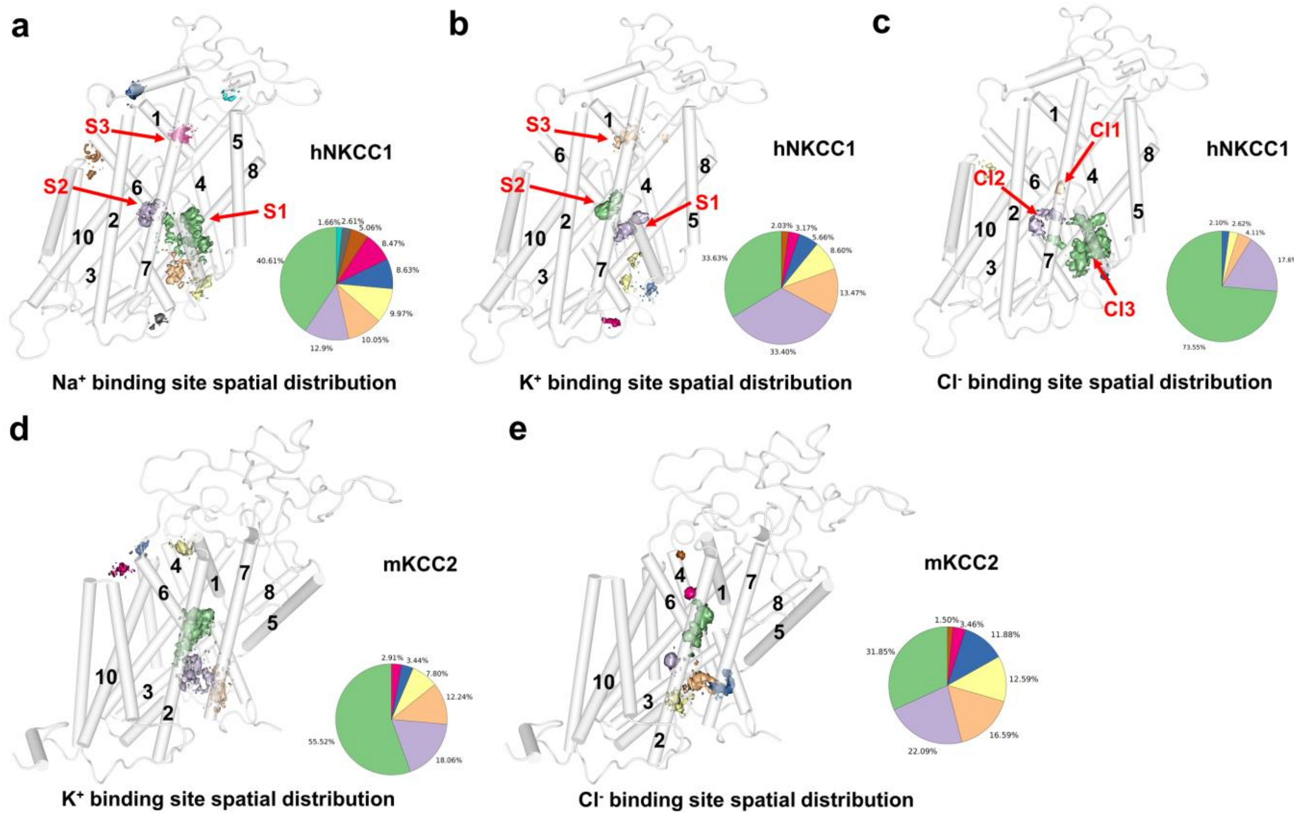

Supplementary Fig. 11. Ion spatial distributions in the proposed binding sites explored by multiple walker Metadynamics simulations.

The ion positions used for calculating the occupancies were filtered based on the following criteria: ions surrounded by at least three residues simultaneously (using a distance cutoff of 4 Å) were considered to belong to a binding site, which was further clustered according to its nearby residue compositions.

a, b, c: Na^+, K^+, and Cl^- binding site spatial distributions in hNKCC1.

d, e: K^+ and Cl^- binding site spatial distributions in mKCC2.

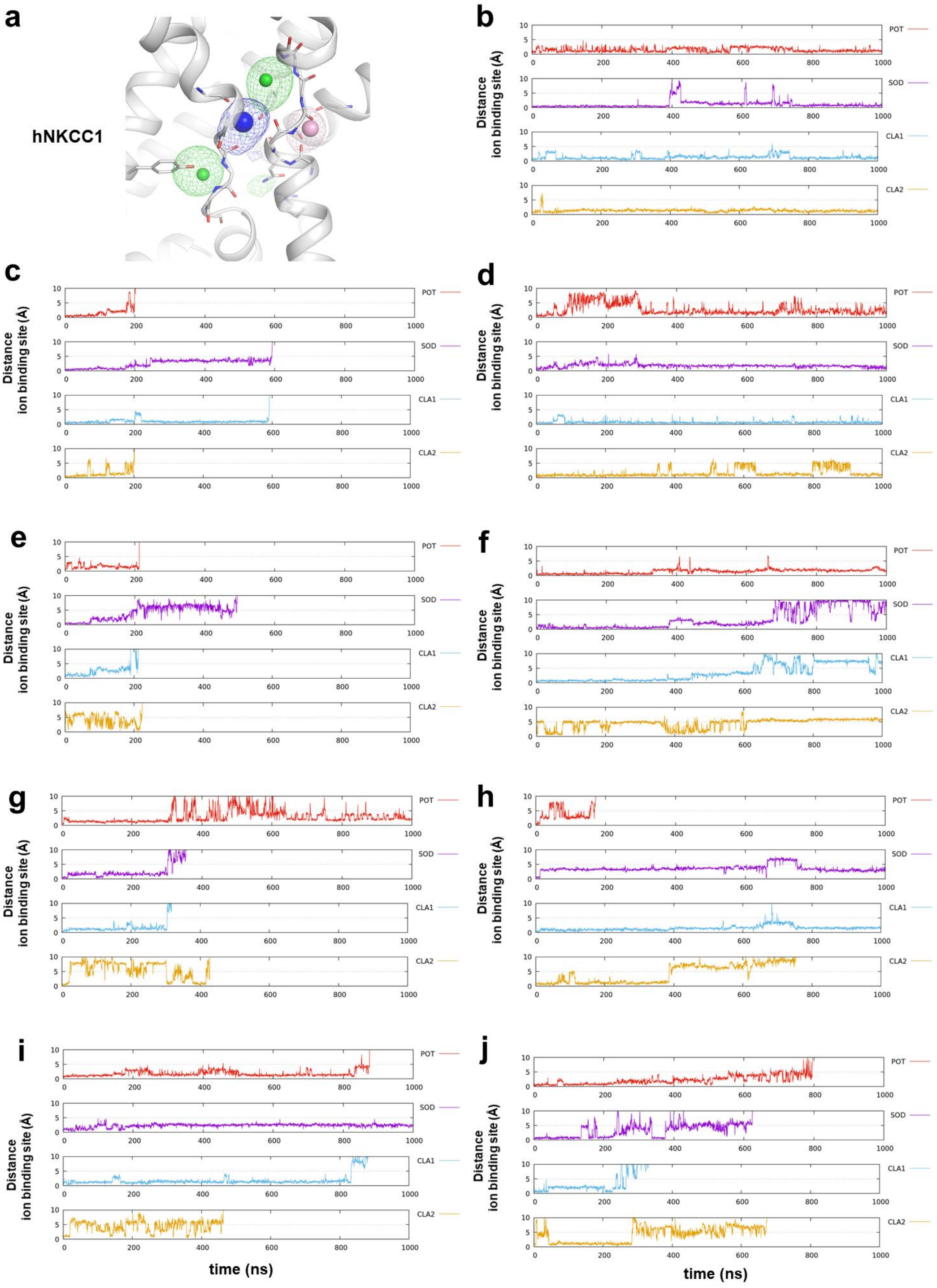

Supplementary Fig. 12. Molecular dynamic simulations of hNKCC1

a. Ion probability densities in hNKCC1 simulations with four ions bounded within the translocation pathway.

b-j. Individual simulation traces of hNKCC1 performed with four ions initially present in the translocation pathway.

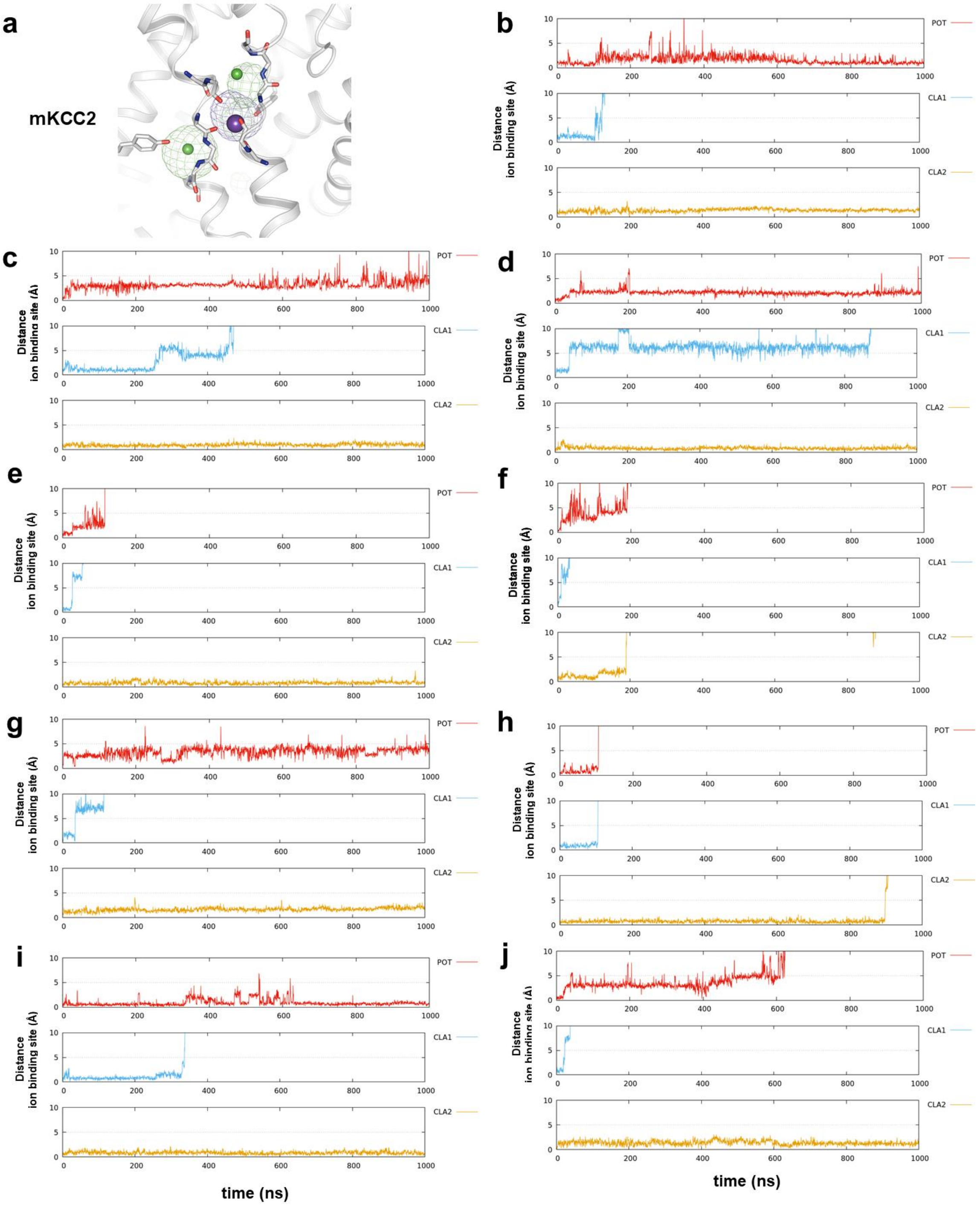

Supplementary Fig. 13. Molecular dynamic simulations of mKCC2

a. Ion probability densities in mKCC2 simulations with three ions bounded within the translocation pathway.

b-j. Individual simulation traces of mKCC2 performed with three ions initially present in the translocation pathway.

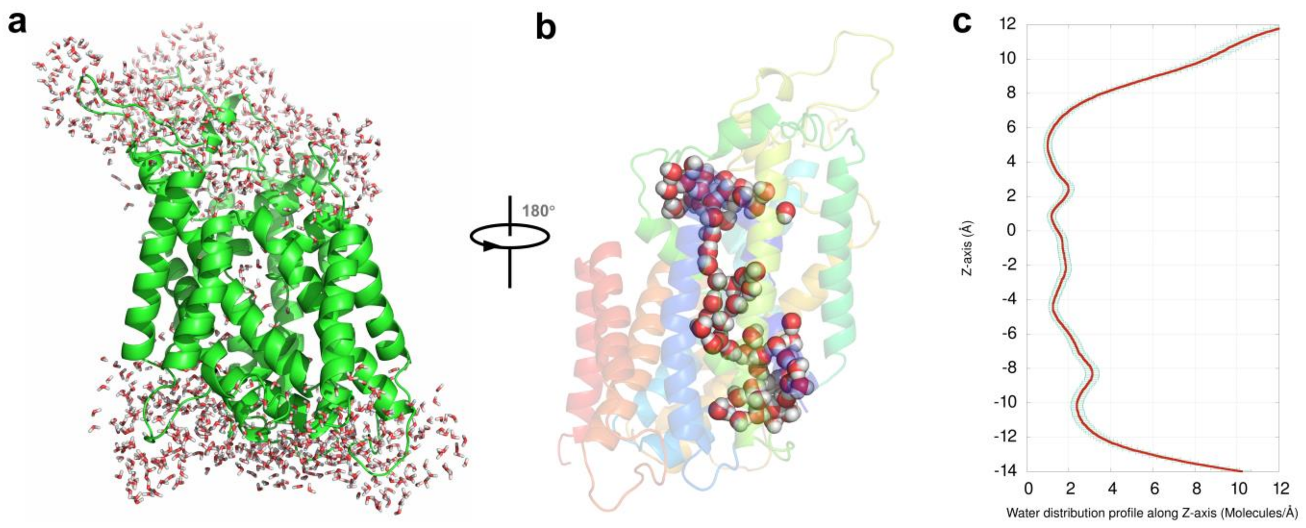

Supplementary Fig. 14. Water permeation pathway in hNKCC1

a. A snapshot of water spatial allocation within 4 Å of the hNKCC1 transporter.

b. Sphere representation of selected water molecules in an apparent permeation pathway through the transporter, from the snapshot in (a).

c. The water distribution profile along the membrane normal (z-axis) from -14 Å to 12 Å.

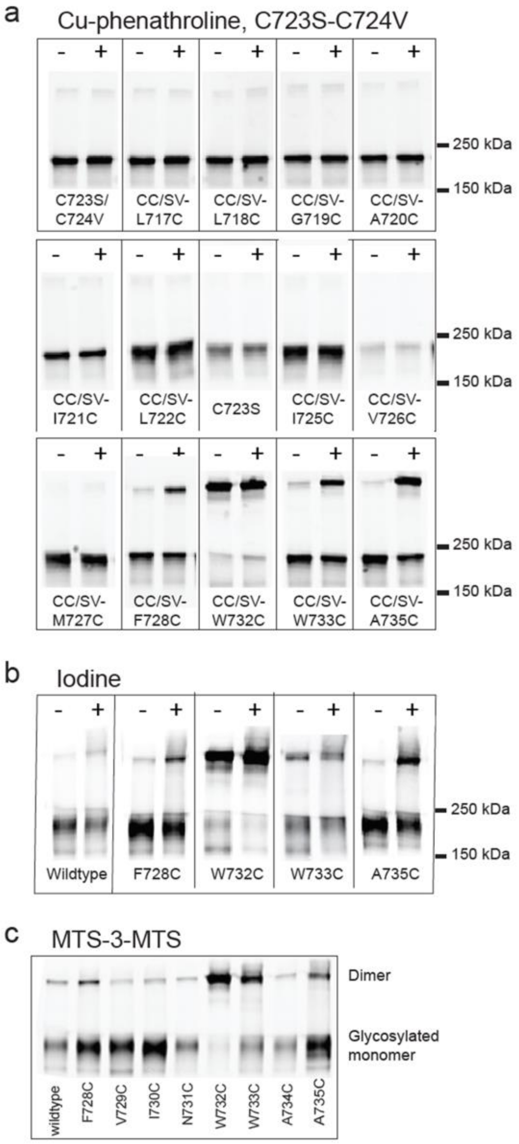

Supplementary Fig. 15. Crosslinking study of the dimeric interface of hNKCC1

Cells were treated to crosslink cysteine residues followed by western blotting with T4 anti-NKCC antibody as described in methods. Representative of 3 experiments.

a. Cupric phenanthroline mediated oxidative crosslinking of wild type hNKCC1 and TM11, TM12 single cysteine substitutions. This panel explores potential interference by native C723 and C724 of which none is seen; constructs were prepared with C723S and C724V substitutions, with no effect on function. Cells were treated with (+) or without (-) 1.5 mM cupric phenanthroline. Monomer and dimer bands of hNKCC1 are indicated.
b. Oxidative crosslinking with (+) or without (-) 1mM iodine.
c. Crosslinking with (+) or without (-) exposure to the bifunctional cysteine crosslinking reagent MTS-3-MTS.

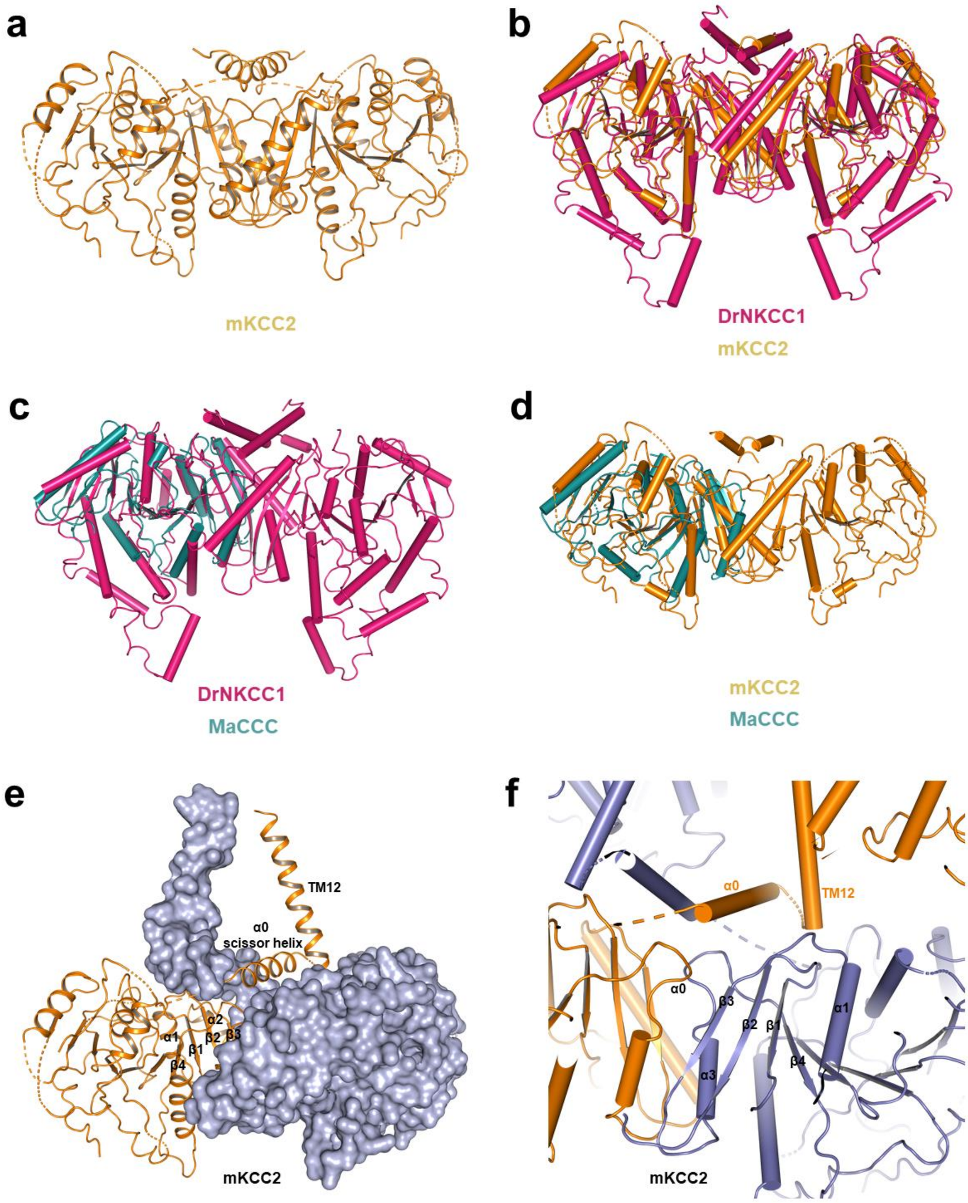

Supplementary Fig. 16. The CTD domain comparison between DrNKCC1, mKCC2, and MaCCC

a. Ribbon representation of the mKCC2 CTD domain from a side view.

b. CTD domain comparison between DrNKCC1 (red) and mKCC2 (brown).

c. CTD domain comparison between DrNKCC1 (red) and MaCCC (green).

d. CTD domain comparison between MaCCC (green) and mKCC2 (brown).

e. The dimer of the CTD domain in mKCC2 with one subunit shown as surface and the other shown as ribbon. α0 scissor helix indicates the helix linker between the TMD and CTD, which pack against each other between the two subunits like a scissor in shape.

f. The TMD and CTD domain interface of mKCC2 with each subunit color-coded (blue and brown).

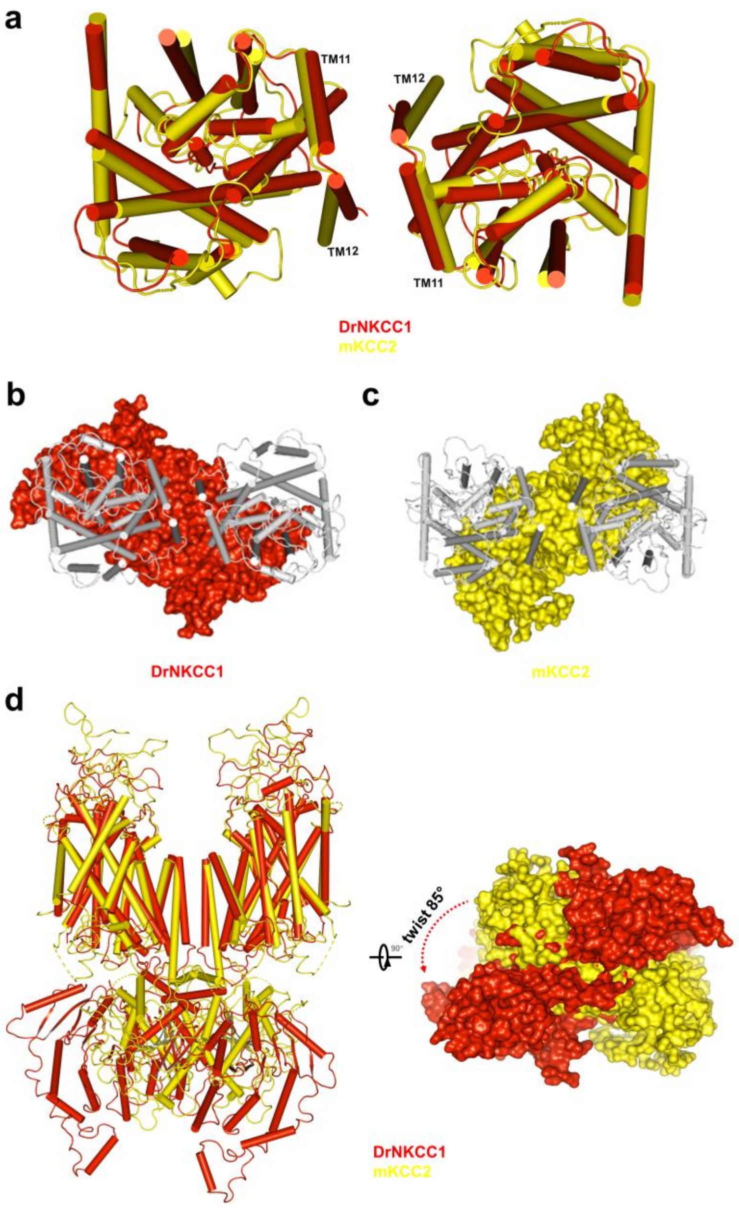

Supplementary Fig. 17. Structure comparison between DrNKCC1 and mKCC2

a. Structural comparison of the dimeric TM regions between DrNKCC1 (red) and mKCC2 (yellow) from a top view.

b. Structure representation of the dimeric DrNKCC1 from a top view with the TMD domain shown as cylinders (gray) and the CTD domain shown as surface (red).

c. Structure representation of the dimeric mKCC2 from a top view with the TMD domain shown as cylinders (gray) and the CTD domain shown as surface (yellow).

d. Left panel shows the structural comparison of the dimeric TM regions between DrNKCC1 (red) and mKCC2 (yellow) from a side view. Right panel shows the bottom view of the comparison. The CTD domains in right panel were shown as surface representation for clarity.

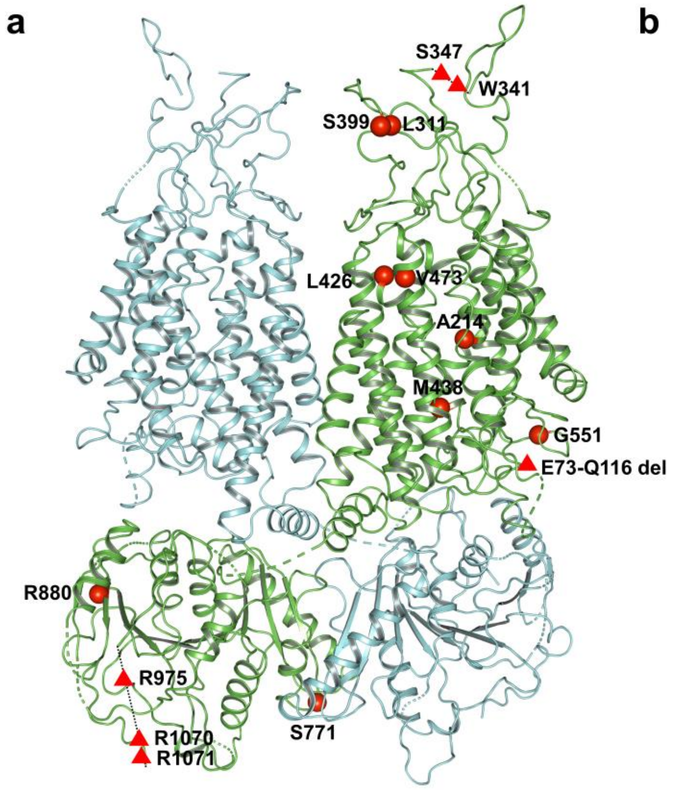

b

Reported mutations in human KCC2b numbering	Equivalent site in current mouse KCC2a model
E50-Q93 del	E73-Q116 del
A191V	A214V
L288H	L311H
W318S	W341S
S323P	S347P
S376L	S399L
L403P	L426P
M415V	M438
V450I	V473I
G528D	G551D
S748 del	S771 del
R857L	R880L
R952H	R975H
R1048W	R1070W
R1049C	R1071C

Supplementary Fig. 18. Disease-related mutations in KCC2

a. Epilepsy related mutations in human KCC2 mapped onto the structure of mKCC2a by sequence alignment. Representative mutations were shown as spheres. Triangles represent the approximate location of mutations in poorly resolved regions.

b. Equivalent mutation sites in mouse KCC2a based on the sequence alignment in human KCC2b numbering.

Figure 2e

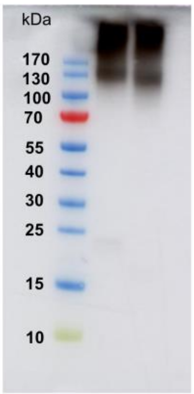

Supplementary Fig. 1a, d

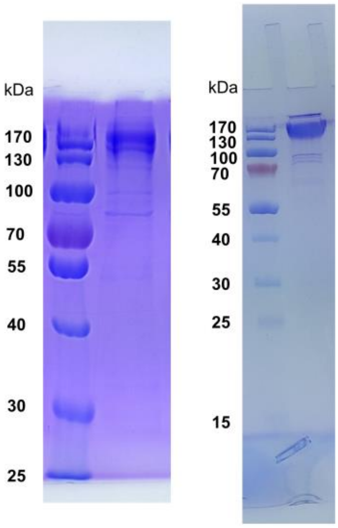

Supplementary Fig. 6a

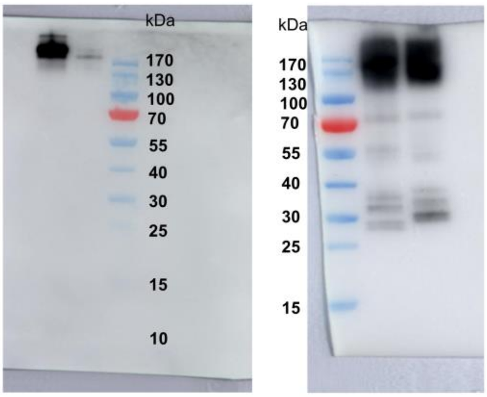

Supplementary Fig. 19. Unprocessed original blot and gel images represented in Fig. 2e and Supplementary Figs. 1a, d, 6a.

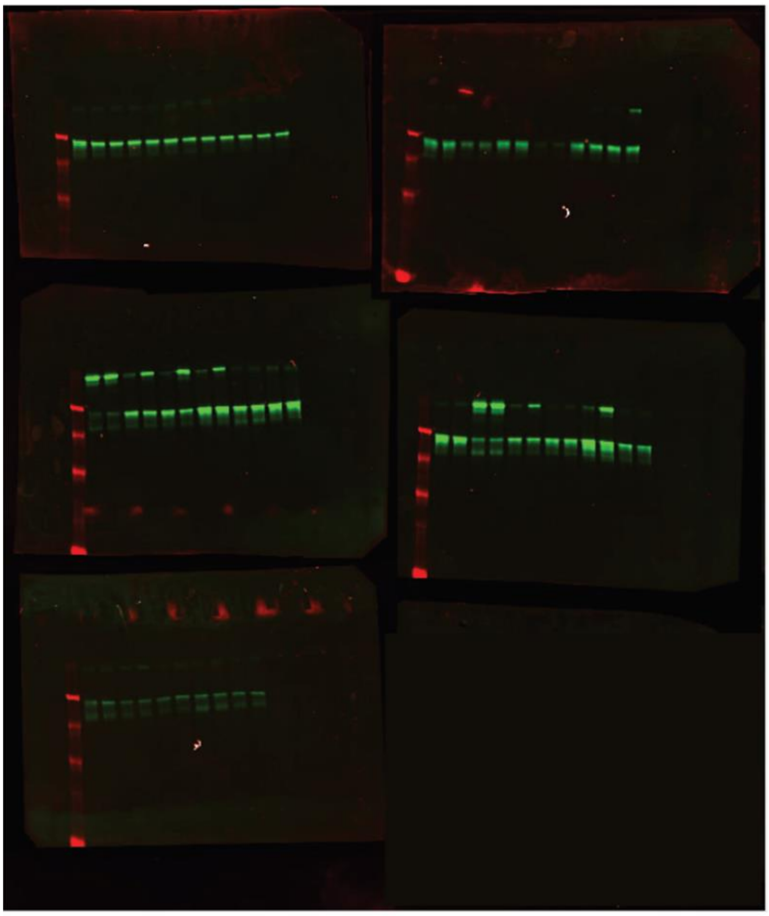

Supplementary Fig. 20. Original blots underlying Fig. 6f and Supplementary Fig. 15a.

Individual blots are rotated into correct orientation from the original Odyssey Licor data which are in Supplementary Data 3. Red: Odyssey 700 nm channel which detects the molecular weight standards. Green: Odyssey 800 nm channel which detects the Odyssey 800 goat anti-mouse secondary bound to the T4 antibody and thus NKCC1. Each of 5 blots has 6 pairs of samples treated without or with Cu-phenanthroline; each pair is of a different hNKCC1 cell line as follows:

Top Left: C723S/C724V, C723S/C724V/L717C, C723S/C724V/L718C, C723S/C724V/G719C, C723S/C724V/A720C, C723S/C724V/I721C

Top Right: C723S/C724V/L722C, C723S, C723S/C724V/I725C, C723S/C724V/V726C, C723S/C724V/M727C, C723S/C724V/F728C

Center Left: C723S/C724V/W732C, C723S/C724V/W733C, C723S/C724V/A735C, F728C, V729C, I730C

Center Right: N731C, W732C, W733C, A734C, A735C, hNKCC1

Bottom Left: L736C, L737C, T738C, Y739C, V740

15b

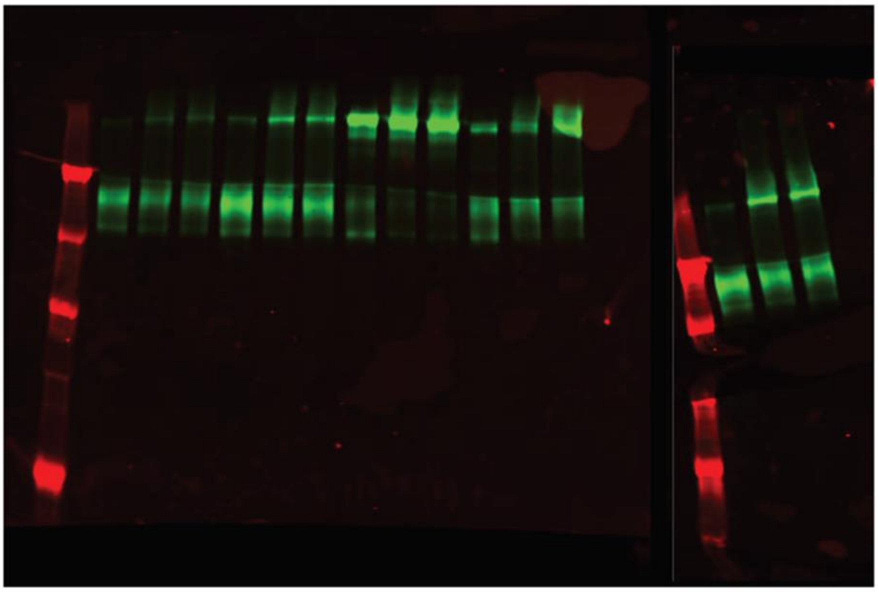

15c

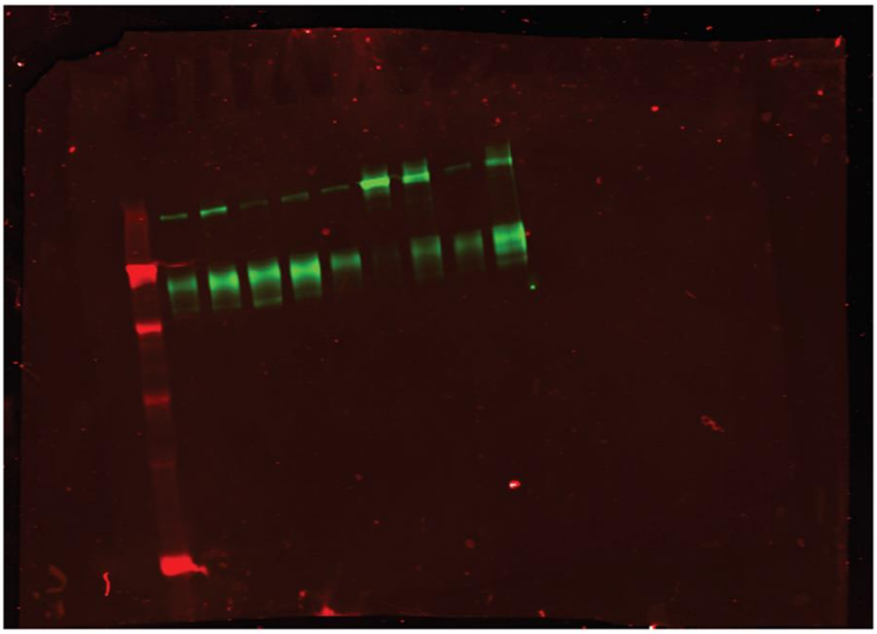

Supplementary Figure 21. Original blots underlying Supplementary Fig. 15b and c.

Individual blots are rotated into correct orientation from the original Odyssey Licor data which are in Supplementary Data 3. Red: Odyssey 700 nm channel which detects the molecular weight standards. Green: Odyssey 800 nm channel which detects the Odyssey 800 goat anti-mouse secondary bound to the T4 antibody and thus NKCC1.

15b. Cell lines as labeled in Supplemental Fig. 15b; note that each cell line has 3 samples, 1 without and 2 with iodine treatment.

15c: Cell lines as labeled in Supplemental Fig. 15c.

Supplementary Note 1: Caveats regarding the activation-inactivation model of Fig. 7. (A). Our analysis relies on the hKCC1 structure[1], obtained with a Strep-tag at the C-terminus. It is possible that the C-terminal tag has altered CTD/TM dimer architecture. (B). NKCC. It is unknown if the phosphorylated N-terminus interacts with the CTD or with intracellular loops of the TM. (C). For both NKCC and KCC the sites which interact with phosphorylated residues are presently unknown.

Supplementary Reference

1 Liu, S. *et al.* Cryo-EM structures of the human cation-chloride cotransporter KCC1. *Science* **366**, 505-+, doi:10.1126/science.aay3129 (2019).
