# Supplementary material for: The structural basis of function and regulation of neuronal cotransporters NKCC1 and KCC2
Source: Commun Biol. 2021 Feb 17;4:226. doi: 10.1038/s42003-021-01750-w (PMC7889885; doi:10.1038/s42003-021-01750-w)
Supplement: Supplementary file 3 — Description of Additional Supplementary Files [file 42003_2021_1750_MOESM3_ESM.pdf]

Description of Additional Supplementary Files

Supplementary Data 1

Source data for Figs. 2f, 3b, 3d, 3e, 4b, 4f, 4g, 6g and Supplementary Figs. 9b, 10c.

Supplementary Data 2

Source data for the MD simulation coordinate file in Supplementary Fig. 14 when analyzing the water number in hNKCC1.

Supplementary Data 3

Original Li-cor Odyssey source data of crosslinking Western blots in Fig.6f and Supplementary Figure 15.
