# Supplementary material for: The structural basis of function and regulation of neuronal cotransporters NKCC1 and KCC2
Source: Commun Biol. 2021 Feb 17;4:226. doi: 10.1038/s42003-021-01750-w (PMC7889885; doi:10.1038/s42003-021-01750-w)
Supplement: Supplementary file 5 — Supplementary Data 2 [file 42003_2021_1750_MOESM5_ESM.docx]

ATOM 1 N TRP X 1 19.688 -2.335 -18.088 0.00 0.00 N

ATOM 2 HT1 TRP X 1 20.700 -2.118 -18.244 0.00 0.00 H

ATOM 3 HT2 TRP X 1 19.053 -2.318 -18.915 0.00 0.00 H

ATOM 4 HT3 TRP X 1 19.665 -3.318 -17.733 0.00 0.00 H

ATOM 5 CA TRP X 1 19.176 -1.295 -17.114 0.00 0.00 C

ATOM 6 HA TRP X 1 19.997 -1.217 -16.405 0.00 0.00 H

ATOM 7 CB TRP X 1 18.933 0.109 -17.950 0.00 0.00 C

ATOM 8 HB1 TRP X 1 18.749 -0.162 -19.010 0.00 0.00 H

ATOM 9 HB2 TRP X 1 19.930 0.618 -17.935 0.00 0.00 H

ATOM 10 CG TRP X 1 17.812 1.009 -17.497 0.00 0.00 C

ATOM 11 CD1 TRP X 1 17.889 2.197 -16.902 0.00 0.00 C

ATOM 12 HD1 TRP X 1 18.816 2.654 -16.579 0.00 0.00 H

ATOM 13 NE1 TRP X 1 16.642 2.760 -16.783 0.00 0.00 N

ATOM 14 HE1 TRP X 1 16.454 3.671 -16.471 0.00 0.00 H

ATOM 15 CE2 TRP X 1 15.760 1.916 -17.317 0.00 0.00 C

ATOM 16 CD2 TRP X 1 16.431 0.748 -17.711 0.00 0.00 C

ATOM 17 CE3 TRP X 1 15.658 -0.261 -18.212 0.00 0.00 C

ATOM 18 HE3 TRP X 1 16.217 -1.180 -18.362 0.00 0.00 H

ATOM 19 CZ3 TRP X 1 14.303 -0.152 -18.407 0.00 0.00 C

ATOM 20 HZ3 TRP X 1 13.732 -0.966 -18.819 0.00 0.00 H

ATOM 21 CZ2 TRP X 1 14.421 2.150 -17.559 0.00 0.00 C

ATOM 22 HZ2 TRP X 1 13.958 3.077 -17.288 0.00 0.00 H

ATOM 23 CH2 TRP X 1 13.724 1.113 -18.192 0.00 0.00 C

ATOM 24 HH2 TRP X 1 12.677 1.238 -18.445 0.00 0.00 H

ATOM 25 C TRP X 1 18.066 -1.638 -16.174 0.00 0.00 C

ATOM 26 O TRP X 1 17.260 -2.493 -16.447 0.00 0.00 O

ATOM 27 N ILE X 2 17.959 -0.981 -15.020 0.00 0.00 N

ATOM 28 HN ILE X 2 18.713 -0.364 -14.817 0.00 0.00 H

ATOM 29 CA ILE X 2 16.947 -1.261 -14.013 0.00 0.00 C

ATOM 30 HA ILE X 2 16.151 -1.789 -14.508 0.00 0.00 H

ATOM 31 CB ILE X 2 17.544 -2.247 -12.914 0.00 0.00 C

ATOM 32 HB ILE X 2 18.276 -2.886 -13.447 0.00 0.00 H

ATOM 33 CG2 ILE X 2 18.162 -1.498 -11.798 0.00 0.00 C

ATOM 34 HG21 ILE X 2 18.791 -0.591 -11.951 0.00 0.00 H

ATOM 35 HG22 ILE X 2 18.768 -2.152 -11.144 0.00 0.00 H

ATOM 36 HG23 ILE X 2 17.404 -1.162 -11.056 0.00 0.00 H

ATOM 37 CG1 ILE X 2 16.523 -3.228 -12.285 0.00 0.00 C

ATOM 38 HG11 ILE X 2 15.827 -3.629 -13.050 0.00 0.00 H

ATOM 39 HG12 ILE X 2 15.905 -2.690 -11.542 0.00 0.00 H

ATOM 40 CD ILE X 2 17.162 -4.400 -11.593 0.00 0.00 C

ATOM 41 HD1 ILE X 2 18.179 -4.097 -11.250 0.00 0.00 H

ATOM 42 HD2 ILE X 2 17.461 -5.226 -12.262 0.00 0.00 H

ATOM 43 HD3 ILE X 2 16.667 -4.809 -10.683 0.00 0.00 H

ATOM 44 C ILE X 2 16.310 0.054 -13.424 0.00 0.00 C

ATOM 45 O ILE X 2 15.201 -0.030 -12.964 0.00 0.00 O

ATOM 46 N LYS X 3 17.049 1.172 -13.367 0.00 0.00 N

ATOM 47 HN LYS X 3 17.982 1.238 -13.713 0.00 0.00 H

ATOM 48 CA LYS X 3 16.746 2.355 -12.632 0.00 0.00 C

ATOM 49 HA LYS X 3 16.983 2.086 -11.610 0.00 0.00 H

ATOM 50 CB LYS X 3 17.724 3.505 -13.055 0.00 0.00 C

ATOM 51 HB1 LYS X 3 17.767 3.512 -14.170 0.00 0.00 H

ATOM 52 HB2 LYS X 3 18.733 3.279 -12.647 0.00 0.00 H

ATOM 53 CG LYS X 3 17.344 4.916 -12.575 0.00 0.00 C

ATOM 54 HG1 LYS X 3 17.008 4.936 -11.514 0.00 0.00 H

ATOM 55 HG2 LYS X 3 16.408 5.223 -13.101 0.00 0.00 H

ATOM 56 CD LYS X 3 18.479 5.911 -12.870 0.00 0.00 C

ATOM 57 HD1 LYS X 3 19.158 5.844 -11.995 0.00 0.00 H

ATOM 58 HD2 LYS X 3 18.104 6.962 -12.820 0.00 0.00 H

ATOM 59 CE LYS X 3 19.220 5.675 -14.205 0.00 0.00 C

ATOM 60 HE1 LYS X 3 18.425 5.582 -14.971 0.00 0.00 H

ATOM 61 HE2 LYS X 3 19.731 4.696 -14.296 0.00 0.00 H

ATOM 62 NZ LYS X 3 20.048 6.777 -14.601 0.00 0.00 N1+

ATOM 63 HZ1 LYS X 3 19.475 7.625 -14.801 0.00 0.00 H

ATOM 64 HZ2 LYS X 3 20.638 6.501 -15.416 0.00 0.00 H

ATOM 65 HZ3 LYS X 3 20.724 7.065 -13.861 0.00 0.00 H

ATOM 66 C LYS X 3 15.236 2.810 -12.726 0.00 0.00 C

ATOM 67 O LYS X 3 14.600 3.006 -11.669 0.00 0.00 O

ATOM 68 N GLY X 4 14.707 2.971 -13.903 0.00 0.00 N

ATOM 69 HN GLY X 4 15.121 2.727 -14.769 0.00 0.00 H

ATOM 70 CA GLY X 4 13.370 3.400 -13.977 0.00 0.00 C

ATOM 71 HA1 GLY X 4 13.157 3.510 -15.035 0.00 0.00 H

ATOM 72 HA2 GLY X 4 13.348 4.416 -13.613 0.00 0.00 H

ATOM 73 C GLY X 4 12.280 2.473 -13.409 0.00 0.00 C

ATOM 74 O GLY X 4 11.468 2.819 -12.512 0.00 0.00 O

ATOM 75 N VAL X 5 12.336 1.190 -13.771 0.00 0.00 N

ATOM 76 HN VAL X 5 13.035 0.822 -14.376 0.00 0.00 H

ATOM 77 CA VAL X 5 11.540 0.164 -13.155 0.00 0.00 C

ATOM 78 HA VAL X 5 10.531 0.493 -13.337 0.00 0.00 H

ATOM 79 CB VAL X 5 11.819 -1.226 -13.778 0.00 0.00 C

ATOM 80 HB VAL X 5 12.905 -1.456 -13.775 0.00 0.00 H

ATOM 81 CG1 VAL X 5 10.995 -2.365 -13.057 0.00 0.00 C

ATOM 82 HG11 VAL X 5 9.952 -2.040 -12.849 0.00 0.00 H

ATOM 83 HG12 VAL X 5 11.470 -2.640 -12.089 0.00 0.00 H

ATOM 84 HG13 VAL X 5 11.070 -3.172 -13.822 0.00 0.00 H

ATOM 85 CG2 VAL X 5 11.289 -1.194 -15.270 0.00 0.00 C

ATOM 86 HG21 VAL X 5 11.492 -2.186 -15.716 0.00 0.00 H

ATOM 87 HG22 VAL X 5 11.686 -0.332 -15.848 0.00 0.00 H

ATOM 88 HG23 VAL X 5 10.209 -0.946 -15.182 0.00 0.00 H

ATOM 89 C VAL X 5 11.636 0.024 -11.663 0.00 0.00 C

ATOM 90 O VAL X 5 10.658 -0.190 -10.939 0.00 0.00 O

ATOM 91 N LEU X 6 12.934 0.056 -11.131 0.00 0.00 N

ATOM 92 HN LEU X 6 13.688 0.217 -11.765 0.00 0.00 H

ATOM 93 CA LEU X 6 13.311 0.008 -9.725 0.00 0.00 C

ATOM 94 HA LEU X 6 12.763 -0.873 -9.424 0.00 0.00 H

ATOM 95 CB LEU X 6 14.790 -0.116 -9.616 0.00 0.00 C

ATOM 96 HB1 LEU X 6 15.345 0.735 -10.076 0.00 0.00 H

ATOM 97 HB2 LEU X 6 15.034 -0.967 -10.289 0.00 0.00 H

ATOM 98 CG LEU X 6 15.389 -0.403 -8.201 0.00 0.00 C

ATOM 99 HG LEU X 6 15.083 0.539 -7.702 0.00 0.00 H

ATOM 100 CD1 LEU X 6 14.787 -1.642 -7.610 0.00 0.00 C

ATOM 101 HD11 LEU X 6 13.720 -1.365 -7.462 0.00 0.00 H

ATOM 102 HD12 LEU X 6 15.181 -2.031 -6.644 0.00 0.00 H

ATOM 103 HD13 LEU X 6 14.899 -2.531 -8.275 0.00 0.00 H

ATOM 104 CD2 LEU X 6 16.936 -0.493 -8.178 0.00 0.00 C

ATOM 105 HD21 LEU X 6 17.428 0.237 -8.858 0.00 0.00 H

ATOM 106 HD22 LEU X 6 17.182 -1.524 -8.506 0.00 0.00 H

ATOM 107 HD23 LEU X 6 17.417 -0.278 -7.202 0.00 0.00 H

ATOM 108 C LEU X 6 12.619 1.118 -8.947 0.00 0.00 C

ATOM 109 O LEU X 6 11.942 0.952 -7.941 0.00 0.00 O

ATOM 110 N VAL X 7 12.778 2.358 -9.415 0.00 0.00 N

ATOM 111 HN VAL X 7 13.388 2.461 -10.202 0.00 0.00 H

ATOM 112 CA VAL X 7 12.331 3.590 -8.850 0.00 0.00 C

ATOM 113 HA VAL X 7 12.669 3.619 -7.827 0.00 0.00 H

ATOM 114 CB VAL X 7 12.946 4.858 -9.553 0.00 0.00 C

ATOM 115 HB VAL X 7 12.976 4.666 -10.646 0.00 0.00 H

ATOM 116 CG1 VAL X 7 12.168 6.143 -9.290 0.00 0.00 C

ATOM 117 HG11 VAL X 7 11.936 6.223 -8.211 0.00 0.00 H

ATOM 118 HG12 VAL X 7 11.238 6.020 -9.887 0.00 0.00 H

ATOM 119 HG13 VAL X 7 12.801 6.991 -9.620 0.00 0.00 H

ATOM 120 CG2 VAL X 7 14.431 5.016 -9.109 0.00 0.00 C

ATOM 121 HG21 VAL X 7 14.932 5.783 -9.740 0.00 0.00 H

ATOM 122 HG22 VAL X 7 14.983 4.075 -9.299 0.00 0.00 H

ATOM 123 HG23 VAL X 7 14.467 5.216 -8.019 0.00 0.00 H

ATOM 124 C VAL X 7 10.770 3.654 -8.733 0.00 0.00 C

ATOM 125 O VAL X 7 10.277 3.923 -7.655 0.00 0.00 O

ATOM 126 N ARG X 8 10.008 3.197 -9.753 0.00 0.00 N

ATOM 127 HN ARG X 8 10.503 3.118 -10.611 0.00 0.00 H

ATOM 128 CA ARG X 8 8.520 3.049 -9.684 0.00 0.00 C

ATOM 129 HA ARG X 8 8.142 4.035 -9.478 0.00 0.00 H

ATOM 130 CB ARG X 8 8.052 2.290 -10.975 0.00 0.00 C

ATOM 131 HB1 ARG X 8 8.750 1.456 -11.186 0.00 0.00 H

ATOM 132 HB2 ARG X 8 8.315 2.898 -11.874 0.00 0.00 H

ATOM 133 CG ARG X 8 6.588 1.994 -11.216 0.00 0.00 C

ATOM 134 HG1 ARG X 8 6.144 1.308 -10.453 0.00 0.00 H

ATOM 135 HG2 ARG X 8 6.567 1.492 -12.203 0.00 0.00 H

ATOM 136 CD ARG X 8 5.769 3.224 -11.236 0.00 0.00 C

ATOM 137 HD1 ARG X 8 6.295 4.112 -11.640 0.00 0.00 H

ATOM 138 HD2 ARG X 8 5.327 3.479 -10.242 0.00 0.00 H

ATOM 139 NE ARG X 8 4.597 2.964 -12.092 0.00 0.00 N

ATOM 140 HE ARG X 8 3.904 2.331 -11.753 0.00 0.00 H

ATOM 141 CZ ARG X 8 4.168 3.815 -13.053 0.00 0.00 C

ATOM 142 NH1 ARG X 8 4.911 4.672 -13.674 0.00 0.00 N1+

ATOM 143 HH11 ARG X 8 5.884 4.557 -13.509 0.00 0.00 H

ATOM 144 HH12 ARG X 8 4.435 5.159 -14.413 0.00 0.00 H

ATOM 145 NH2 ARG X 8 2.931 3.765 -13.396 0.00 0.00 N

ATOM 146 HH21 ARG X 8 2.422 3.172 -12.777 0.00 0.00 H

ATOM 147 HH22 ARG X 8 2.601 4.670 -13.653 0.00 0.00 H

ATOM 148 C ARG X 8 8.100 2.233 -8.486 0.00 0.00 C

ATOM 149 O ARG X 8 7.194 2.622 -7.693 0.00 0.00 O

ATOM 150 N CYS X 9 8.599 0.943 -8.497 0.00 0.00 N

ATOM 151 HN CYS X 9 9.200 0.660 -9.239 0.00 0.00 H

ATOM 152 CA CYS X 9 8.135 -0.101 -7.601 0.00 0.00 C

ATOM 153 HA CYS X 9 7.066 -0.226 -7.720 0.00 0.00 H

ATOM 154 CB CYS X 9 8.645 -1.498 -8.169 0.00 0.00 C

ATOM 155 HB1 CYS X 9 9.747 -1.516 -8.017 0.00 0.00 H

ATOM 156 HB2 CYS X 9 8.430 -1.669 -9.239 0.00 0.00 H

ATOM 157 SG CYS X 9 7.920 -2.929 -7.235 0.00 0.00 S

ATOM 158 HG1 CYS X 9 9.005 -3.155 -6.497 0.00 0.00 H

ATOM 159 C CYS X 9 8.453 0.218 -6.136 0.00 0.00 C

ATOM 160 O CYS X 9 7.513 0.159 -5.358 0.00 0.00 O

ATOM 161 N MET X 10 9.748 0.684 -5.784 0.00 0.00 N

ATOM 162 HN MET X 10 10.513 0.705 -6.428 0.00 0.00 H

ATOM 163 CA MET X 10 9.930 1.291 -4.463 0.00 0.00 C

ATOM 164 HA MET X 10 9.726 0.490 -3.767 0.00 0.00 H

ATOM 165 CB MET X 10 11.402 1.623 -4.355 0.00 0.00 C

ATOM 166 HB1 MET X 10 11.529 2.419 -3.598 0.00 0.00 H

ATOM 167 HB2 MET X 10 11.797 2.229 -5.199 0.00 0.00 H

ATOM 168 CG MET X 10 12.190 0.288 -4.251 0.00 0.00 C

ATOM 169 HG1 MET X 10 12.012 -0.289 -5.190 0.00 0.00 H

ATOM 170 HG2 MET X 10 11.737 -0.367 -3.481 0.00 0.00 H

ATOM 171 SD MET X 10 14.003 0.639 -3.986 0.00 0.00 S

ATOM 172 CE MET X 10 13.907 1.261 -2.273 0.00 0.00 C

ATOM 173 HE1 MET X 10 14.866 1.609 -1.849 0.00 0.00 H

ATOM 174 HE2 MET X 10 13.496 0.371 -1.745 0.00 0.00 H

ATOM 175 HE3 MET X 10 13.127 2.053 -2.230 0.00 0.00 H

ATOM 176 C MET X 10 9.146 2.457 -3.981 0.00 0.00 C

ATOM 177 O MET X 10 8.714 2.486 -2.831 0.00 0.00 O

ATOM 178 N LEU X 11 8.910 3.394 -4.896 0.00 0.00 N

ATOM 179 HN LEU X 11 9.281 3.308 -5.817 0.00 0.00 H

ATOM 180 CA LEU X 11 7.969 4.484 -4.557 0.00 0.00 C

ATOM 181 HA LEU X 11 8.285 4.968 -3.637 0.00 0.00 H

ATOM 182 CB LEU X 11 7.949 5.642 -5.704 0.00 0.00 C

ATOM 183 HB1 LEU X 11 7.208 6.358 -5.268 0.00 0.00 H

ATOM 184 HB2 LEU X 11 7.621 5.182 -6.657 0.00 0.00 H

ATOM 185 CG LEU X 11 9.272 6.423 -5.914 0.00 0.00 C

ATOM 186 HG LEU X 11 10.156 5.757 -5.895 0.00 0.00 H

ATOM 187 CD1 LEU X 11 9.408 7.086 -7.284 0.00 0.00 C

ATOM 188 HD11 LEU X 11 9.440 6.349 -8.119 0.00 0.00 H

ATOM 189 HD12 LEU X 11 10.377 7.626 -7.356 0.00 0.00 H

ATOM 190 HD13 LEU X 11 8.589 7.785 -7.551 0.00 0.00 H

ATOM 191 CD2 LEU X 11 9.344 7.550 -4.772 0.00 0.00 C

ATOM 192 HD21 LEU X 11 10.092 8.327 -5.034 0.00 0.00 H

ATOM 193 HD22 LEU X 11 9.619 7.120 -3.786 0.00 0.00 H

ATOM 194 HD23 LEU X 11 8.388 8.117 -4.722 0.00 0.00 H

ATOM 195 C LEU X 11 6.518 4.039 -4.363 0.00 0.00 C

ATOM 196 O LEU X 11 5.804 4.565 -3.508 0.00 0.00 O

ATOM 197 N ASN X 12 5.987 3.036 -5.110 0.00 0.00 N

ATOM 198 HN ASN X 12 6.397 2.707 -5.961 0.00 0.00 H

ATOM 199 CA ASN X 12 4.690 2.467 -4.720 0.00 0.00 C

ATOM 200 HA ASN X 12 3.983 3.284 -4.761 0.00 0.00 H

ATOM 201 CB ASN X 12 4.427 1.403 -5.797 0.00 0.00 C

ATOM 202 HB1 ASN X 12 5.159 0.576 -5.854 0.00 0.00 H

ATOM 203 HB2 ASN X 12 4.461 1.945 -6.767 0.00 0.00 H

ATOM 204 CG ASN X 12 3.069 0.636 -5.753 0.00 0.00 C

ATOM 205 OD1 ASN X 12 3.085 -0.575 -5.490 0.00 0.00 O

ATOM 206 ND2 ASN X 12 1.957 1.384 -5.914 0.00 0.00 N

ATOM 207 HD21 ASN X 12 1.110 1.008 -5.518 0.00 0.00 H

ATOM 208 HD22 ASN X 12 1.980 2.269 -6.364 0.00 0.00 H

ATOM 209 C ASN X 12 4.710 1.871 -3.277 0.00 0.00 C

ATOM 210 O ASN X 12 3.721 2.009 -2.497 0.00 0.00 O

ATOM 211 N ILE X 13 5.756 1.058 -2.907 0.00 0.00 N

ATOM 212 HN ILE X 13 6.430 0.694 -3.546 0.00 0.00 H

ATOM 213 CA ILE X 13 5.915 0.357 -1.583 0.00 0.00 C

ATOM 214 HA ILE X 13 5.001 -0.209 -1.525 0.00 0.00 H

ATOM 215 CB ILE X 13 6.753 -0.967 -1.807 0.00 0.00 C

ATOM 216 HB ILE X 13 7.698 -0.657 -2.289 0.00 0.00 H

ATOM 217 CG2 ILE X 13 7.067 -1.684 -0.465 0.00 0.00 C

ATOM 218 HG21 ILE X 13 6.197 -1.697 0.227 0.00 0.00 H

ATOM 219 HG22 ILE X 13 7.775 -1.070 0.116 0.00 0.00 H

ATOM 220 HG23 ILE X 13 7.582 -2.665 -0.414 0.00 0.00 H

ATOM 221 CG1 ILE X 13 6.274 -2.017 -2.879 0.00 0.00 C

ATOM 222 HG11 ILE X 13 5.726 -1.437 -3.652 0.00 0.00 H

ATOM 223 HG12 ILE X 13 5.552 -2.726 -2.426 0.00 0.00 H

ATOM 224 CD ILE X 13 7.412 -2.799 -3.519 0.00 0.00 C

ATOM 225 HD1 ILE X 13 7.926 -3.498 -2.832 0.00 0.00 H

ATOM 226 HD2 ILE X 13 8.121 -2.134 -4.050 0.00 0.00 H

ATOM 227 HD3 ILE X 13 6.935 -3.354 -4.351 0.00 0.00 H

ATOM 228 C ILE X 13 6.187 1.144 -0.356 0.00 0.00 C

ATOM 229 O ILE X 13 5.870 0.748 0.836 0.00 0.00 O

ATOM 230 N TRP X 14 6.662 2.365 -0.476 0.00 0.00 N

ATOM 231 HN TRP X 14 6.847 2.647 -1.422 0.00 0.00 H

ATOM 232 CA TRP X 14 6.996 3.214 0.633 0.00 0.00 C

ATOM 233 HA TRP X 14 6.869 2.586 1.508 0.00 0.00 H

ATOM 234 CB TRP X 14 8.480 3.642 0.505 0.00 0.00 C

ATOM 235 HB1 TRP X 14 8.631 4.516 1.189 0.00 0.00 H

ATOM 236 HB2 TRP X 14 8.641 3.967 -0.538 0.00 0.00 H

ATOM 237 CG TRP X 14 9.428 2.545 0.863 0.00 0.00 C

ATOM 238 CD1 TRP X 14 9.250 1.276 1.381 0.00 0.00 C

ATOM 239 HD1 TRP X 14 8.373 0.658 1.407 0.00 0.00 H

ATOM 240 NE1 TRP X 14 10.430 0.711 1.749 0.00 0.00 N

ATOM 241 HE1 TRP X 14 10.599 0.290 2.612 0.00 0.00 H

ATOM 242 CE2 TRP X 14 11.391 1.547 1.324 0.00 0.00 C

ATOM 243 CD2 TRP X 14 10.872 2.703 0.818 0.00 0.00 C

ATOM 244 CE3 TRP X 14 11.661 3.844 0.617 0.00 0.00 C

ATOM 245 HE3 TRP X 14 11.289 4.772 0.202 0.00 0.00 H

ATOM 246 CZ3 TRP X 14 13.038 3.711 0.715 0.00 0.00 C

ATOM 247 HZ3 TRP X 14 13.647 4.552 0.444 0.00 0.00 H

ATOM 248 CZ2 TRP X 14 12.785 1.399 1.315 0.00 0.00 C

ATOM 249 HZ2 TRP X 14 13.305 0.541 1.701 0.00 0.00 H

ATOM 250 CH2 TRP X 14 13.583 2.493 0.916 0.00 0.00 C

ATOM 251 HH2 TRP X 14 14.609 2.229 0.703 0.00 0.00 H

ATOM 252 C TRP X 14 5.992 4.334 0.712 0.00 0.00 C

ATOM 253 O TRP X 14 5.820 5.039 -0.271 0.00 0.00 O

ATOM 254 N GLY X 15 5.192 4.448 1.806 0.00 0.00 N

ATOM 255 HN GLY X 15 5.160 3.683 2.458 0.00 0.00 H

ATOM 256 CA GLY X 15 4.190 5.444 2.024 0.00 0.00 C

ATOM 257 HA1 GLY X 15 3.353 5.242 1.371 0.00 0.00 H

ATOM 258 HA2 GLY X 15 4.568 6.453 2.069 0.00 0.00 H

ATOM 259 C GLY X 15 3.709 5.255 3.406 0.00 0.00 C

ATOM 260 O GLY X 15 4.477 5.081 4.388 0.00 0.00 O

ATOM 261 N VAL X 16 2.383 5.391 3.615 0.00 0.00 N

ATOM 262 HN VAL X 16 1.809 5.600 2.822 0.00 0.00 H

ATOM 263 CA VAL X 16 1.674 5.485 4.812 0.00 0.00 C

ATOM 264 HA VAL X 16 2.168 6.317 5.294 0.00 0.00 H

ATOM 265 CB VAL X 16 0.198 6.018 4.676 0.00 0.00 C

ATOM 266 HB VAL X 16 -0.189 6.170 5.712 0.00 0.00 H

ATOM 267 CG1 VAL X 16 0.238 7.303 3.889 0.00 0.00 C

ATOM 268 HG11 VAL X 16 -0.760 7.781 3.849 0.00 0.00 H

ATOM 269 HG12 VAL X 16 0.528 7.190 2.819 0.00 0.00 H

ATOM 270 HG13 VAL X 16 0.929 7.942 4.476 0.00 0.00 H

ATOM 271 CG2 VAL X 16 -0.812 5.014 4.047 0.00 0.00 C

ATOM 272 HG21 VAL X 16 -1.772 5.473 4.386 0.00 0.00 H

ATOM 273 HG22 VAL X 16 -0.715 3.964 4.380 0.00 0.00 H

ATOM 274 HG23 VAL X 16 -0.675 4.917 2.950 0.00 0.00 H

ATOM 275 C VAL X 16 1.817 4.344 5.760 0.00 0.00 C

ATOM 276 O VAL X 16 1.944 4.452 6.966 0.00 0.00 O

ATOM 277 N MET X 17 1.932 3.073 5.292 0.00 0.00 N

ATOM 278 HN MET X 17 1.751 2.951 4.323 0.00 0.00 H

ATOM 279 CA MET X 17 2.199 2.006 6.168 0.00 0.00 C

ATOM 280 HA MET X 17 1.485 1.967 6.970 0.00 0.00 H

ATOM 281 CB MET X 17 2.140 0.647 5.385 0.00 0.00 C

ATOM 282 HB1 MET X 17 2.657 0.638 4.404 0.00 0.00 H

ATOM 283 HB2 MET X 17 1.066 0.638 5.077 0.00 0.00 H

ATOM 284 CG MET X 17 2.434 -0.678 6.110 0.00 0.00 C

ATOM 285 HG1 MET X 17 3.536 -0.757 6.165 0.00 0.00 H

ATOM 286 HG2 MET X 17 2.049 -1.496 5.467 0.00 0.00 H

ATOM 287 SD MET X 17 1.850 -0.858 7.873 0.00 0.00 S

ATOM 288 CE MET X 17 0.099 -0.710 7.420 0.00 0.00 C

ATOM 289 HE1 MET X 17 -0.110 0.369 7.215 0.00 0.00 H

ATOM 290 HE2 MET X 17 -0.448 -1.111 8.294 0.00 0.00 H

ATOM 291 HE3 MET X 17 0.008 -1.367 6.526 0.00 0.00 H

ATOM 292 C MET X 17 3.547 2.077 6.825 0.00 0.00 C

ATOM 293 O MET X 17 3.628 1.969 8.010 0.00 0.00 O

ATOM 294 N LEU X 18 4.542 2.275 6.003 0.00 0.00 N

ATOM 295 HN LEU X 18 4.358 2.478 5.042 0.00 0.00 H

ATOM 296 CA LEU X 18 5.973 2.474 6.479 0.00 0.00 C

ATOM 297 HA LEU X 18 6.139 1.709 7.233 0.00 0.00 H

ATOM 298 CB LEU X 18 6.987 2.495 5.338 0.00 0.00 C

ATOM 299 HB1 LEU X 18 6.497 3.181 4.614 0.00 0.00 H

ATOM 300 HB2 LEU X 18 6.907 1.561 4.746 0.00 0.00 H

ATOM 301 CG LEU X 18 8.437 2.954 5.596 0.00 0.00 C

ATOM 302 HG LEU X 18 8.430 4.028 5.867 0.00 0.00 H

ATOM 303 CD1 LEU X 18 9.190 2.028 6.637 0.00 0.00 C

ATOM 304 HD11 LEU X 18 8.607 2.085 7.578 0.00 0.00 H

ATOM 305 HD12 LEU X 18 10.253 2.341 6.743 0.00 0.00 H

ATOM 306 HD13 LEU X 18 9.281 0.977 6.304 0.00 0.00 H

ATOM 307 CD2 LEU X 18 9.222 2.987 4.329 0.00 0.00 C

ATOM 308 HD21 LEU X 18 10.230 3.403 4.573 0.00 0.00 H

ATOM 309 HD22 LEU X 18 8.748 3.601 3.541 0.00 0.00 H

ATOM 310 HD23 LEU X 18 9.491 1.958 3.993 0.00 0.00 H

ATOM 311 C LEU X 18 6.184 3.721 7.387 0.00 0.00 C

ATOM 312 O LEU X 18 6.808 3.644 8.448 0.00 0.00 O

ATOM 313 N PHE X 19 5.677 4.885 6.962 0.00 0.00 N

ATOM 314 HN PHE X 19 5.096 4.928 6.149 0.00 0.00 H

ATOM 315 CA PHE X 19 5.945 6.119 7.675 0.00 0.00 C

ATOM 316 HA PHE X 19 6.859 6.071 8.252 0.00 0.00 H

ATOM 317 CB PHE X 19 5.993 7.244 6.567 0.00 0.00 C

ATOM 318 HB1 PHE X 19 5.886 8.278 6.951 0.00 0.00 H

ATOM 319 HB2 PHE X 19 5.131 7.046 5.892 0.00 0.00 H

ATOM 320 CG PHE X 19 7.271 7.179 5.804 0.00 0.00 C

ATOM 321 CD1 PHE X 19 7.386 7.143 4.387 0.00 0.00 C

ATOM 322 HD1 PHE X 19 6.503 7.184 3.784 0.00 0.00 H

ATOM 323 CE1 PHE X 19 8.584 7.006 3.741 0.00 0.00 C

ATOM 324 HE1 PHE X 19 8.708 6.797 2.689 0.00 0.00 H

ATOM 325 CZ PHE X 19 9.781 7.138 4.427 0.00 0.00 C

ATOM 326 HZ PHE X 19 10.728 7.092 3.908 0.00 0.00 H

ATOM 327 CD2 PHE X 19 8.454 7.334 6.457 0.00 0.00 C

ATOM 328 HD2 PHE X 19 8.405 7.521 7.518 0.00 0.00 H

ATOM 329 CE2 PHE X 19 9.686 7.326 5.805 0.00 0.00 C

ATOM 330 HE2 PHE X 19 10.622 7.250 6.336 0.00 0.00 H

ATOM 331 C PHE X 19 4.897 6.574 8.670 0.00 0.00 C

ATOM 332 O PHE X 19 5.047 7.618 9.272 0.00 0.00 O

ATOM 333 N ILE X 20 3.889 5.733 8.938 0.00 0.00 N

ATOM 334 HN ILE X 20 3.693 4.973 8.322 0.00 0.00 H

ATOM 335 CA ILE X 20 2.755 5.887 9.913 0.00 0.00 C

ATOM 336 HA ILE X 20 3.189 6.493 10.707 0.00 0.00 H

ATOM 337 CB ILE X 20 1.467 6.502 9.379 0.00 0.00 C

ATOM 338 HB ILE X 20 0.808 5.732 8.915 0.00 0.00 H

ATOM 339 CG2 ILE X 20 0.673 6.928 10.675 0.00 0.00 C

ATOM 340 HG21 ILE X 20 -0.254 7.374 10.242 0.00 0.00 H

ATOM 341 HG22 ILE X 20 1.290 7.619 11.292 0.00 0.00 H

ATOM 342 HG23 ILE X 20 0.289 6.094 11.304 0.00 0.00 H

ATOM 343 CG1 ILE X 20 1.720 7.614 8.415 0.00 0.00 C

ATOM 344 HG11 ILE X 20 2.320 7.162 7.593 0.00 0.00 H

ATOM 345 HG12 ILE X 20 2.300 8.383 8.982 0.00 0.00 H

ATOM 346 CD ILE X 20 0.495 8.218 7.758 0.00 0.00 C

ATOM 347 HD1 ILE X 20 0.804 8.810 6.875 0.00 0.00 H

ATOM 348 HD2 ILE X 20 -0.046 8.960 8.375 0.00 0.00 H

ATOM 349 HD3 ILE X 20 -0.178 7.385 7.437 0.00 0.00 H

ATOM 350 C ILE X 20 2.473 4.621 10.692 0.00 0.00 C

ATOM 351 O ILE X 20 2.666 4.559 11.927 0.00 0.00 O

ATOM 352 N ARG X 21 2.081 3.457 10.031 0.00 0.00 N

ATOM 353 HN ARG X 21 1.949 3.353 9.046 0.00 0.00 H

ATOM 354 CA ARG X 21 1.476 2.298 10.778 0.00 0.00 C

ATOM 355 HA ARG X 21 1.268 2.672 11.769 0.00 0.00 H

ATOM 356 CB ARG X 21 0.118 1.928 10.019 0.00 0.00 C

ATOM 357 HB1 ARG X 21 -0.170 0.874 10.179 0.00 0.00 H

ATOM 358 HB2 ARG X 21 0.600 2.025 9.017 0.00 0.00 H

ATOM 359 CG ARG X 21 -1.016 2.932 10.151 0.00 0.00 C

ATOM 360 HG1 ARG X 21 -1.898 2.632 9.531 0.00 0.00 H

ATOM 361 HG2 ARG X 21 -0.811 3.909 9.665 0.00 0.00 H

ATOM 362 CD ARG X 21 -1.456 3.249 11.584 0.00 0.00 C

ATOM 363 HD1 ARG X 21 -0.640 3.720 12.187 0.00 0.00 H

ATOM 364 HD2 ARG X 21 -1.576 2.224 12.012 0.00 0.00 H

ATOM 365 NE ARG X 21 -2.772 4.017 11.581 0.00 0.00 N

ATOM 366 HE ARG X 21 -2.878 5.008 11.645 0.00 0.00 H

ATOM 367 CZ ARG X 21 -3.949 3.439 11.511 0.00 0.00 C

ATOM 368 NH1 ARG X 21 -4.143 2.130 11.376 0.00 0.00 N1+

ATOM 369 HH11 ARG X 21 -3.376 1.516 11.551 0.00 0.00 H

ATOM 370 HH12 ARG X 21 -5.096 1.837 11.412 0.00 0.00 H

ATOM 371 NH2 ARG X 21 -4.987 4.203 11.539 0.00 0.00 N

ATOM 372 HH21 ARG X 21 -5.902 3.807 11.516 0.00 0.00 H

ATOM 373 HH22 ARG X 21 -4.936 5.177 11.772 0.00 0.00 H

ATOM 374 C ARG X 21 2.189 1.032 11.131 0.00 0.00 C

ATOM 375 O ARG X 21 1.626 0.254 11.888 0.00 0.00 O

ATOM 376 N LEU X 22 3.466 0.917 10.704 0.00 0.00 N

ATOM 377 HN LEU X 22 3.917 1.527 10.057 0.00 0.00 H

ATOM 378 CA LEU X 22 4.249 -0.277 10.899 0.00 0.00 C

ATOM 379 HA LEU X 22 3.593 -1.043 10.535 0.00 0.00 H

ATOM 380 CB LEU X 22 5.520 -0.044 10.125 0.00 0.00 C

ATOM 381 HB1 LEU X 22 6.133 0.756 10.592 0.00 0.00 H

ATOM 382 HB2 LEU X 22 5.075 0.064 9.121 0.00 0.00 H

ATOM 383 CG LEU X 22 6.528 -1.305 10.147 0.00 0.00 C

ATOM 384 HG LEU X 22 6.755 -1.753 11.139 0.00 0.00 H

ATOM 385 CD1 LEU X 22 6.006 -2.437 9.298 0.00 0.00 C

ATOM 386 HD11 LEU X 22 5.879 -2.010 8.278 0.00 0.00 H

ATOM 387 HD12 LEU X 22 5.078 -2.808 9.776 0.00 0.00 H

ATOM 388 HD13 LEU X 22 6.706 -3.292 9.280 0.00 0.00 H

ATOM 389 CD2 LEU X 22 7.871 -0.733 9.560 0.00 0.00 C

ATOM 390 HD21 LEU X 22 8.625 -1.546 9.575 0.00 0.00 H

ATOM 391 HD22 LEU X 22 8.246 0.081 10.212 0.00 0.00 H

ATOM 392 HD23 LEU X 22 7.572 -0.305 8.582 0.00 0.00 H

ATOM 393 C LEU X 22 4.625 -0.586 12.432 0.00 0.00 C

ATOM 394 O LEU X 22 4.954 -1.717 12.644 0.00 0.00 O

ATOM 395 N SER X 23 4.501 0.383 13.383 0.00 0.00 N

ATOM 396 HN SER X 23 4.469 1.320 13.055 0.00 0.00 H

ATOM 397 CA SER X 23 4.622 0.105 14.805 0.00 0.00 C

ATOM 398 HA SER X 23 5.534 -0.426 15.051 0.00 0.00 H

ATOM 399 CB SER X 23 4.866 1.390 15.735 0.00 0.00 C

ATOM 400 HB1 SER X 23 5.922 1.695 15.648 0.00 0.00 H

ATOM 401 HB2 SER X 23 4.663 1.073 16.782 0.00 0.00 H

ATOM 402 OG SER X 23 3.981 2.363 15.321 0.00 0.00 O

ATOM 403 HG1 SER X 23 3.896 3.031 16.004 0.00 0.00 H

ATOM 404 C SER X 23 3.424 -0.694 15.333 0.00 0.00 C

ATOM 405 O SER X 23 3.602 -1.588 16.137 0.00 0.00 O

ATOM 406 N TRP X 24 2.232 -0.540 14.759 0.00 0.00 N

ATOM 407 HN TRP X 24 1.982 0.227 14.166 0.00 0.00 H

ATOM 408 CA TRP X 24 1.223 -1.421 15.090 0.00 0.00 C

ATOM 409 HA TRP X 24 1.093 -1.383 16.162 0.00 0.00 H

ATOM 410 CB TRP X 24 -0.142 -0.973 14.508 0.00 0.00 C

ATOM 411 HB1 TRP X 24 0.003 -1.030 13.409 0.00 0.00 H

ATOM 412 HB2 TRP X 24 -0.314 0.102 14.711 0.00 0.00 H

ATOM 413 CG TRP X 24 -1.365 -1.754 14.850 0.00 0.00 C

ATOM 414 CD1 TRP X 24 -2.193 -1.459 15.980 0.00 0.00 C

ATOM 415 HD1 TRP X 24 -2.040 -0.664 16.700 0.00 0.00 H

ATOM 416 NE1 TRP X 24 -3.290 -2.328 16.039 0.00 0.00 N

ATOM 417 HE1 TRP X 24 -4.148 -2.230 16.514 0.00 0.00 H

ATOM 418 CE2 TRP X 24 -3.237 -3.216 14.975 0.00 0.00 C

ATOM 419 CD2 TRP X 24 -2.034 -2.848 14.195 0.00 0.00 C

ATOM 420 CE3 TRP X 24 -1.761 -3.609 13.075 0.00 0.00 C

ATOM 421 HE3 TRP X 24 -1.003 -3.338 12.343 0.00 0.00 H

ATOM 422 CZ3 TRP X 24 -2.767 -4.578 12.586 0.00 0.00 C

ATOM 423 HZ3 TRP X 24 -2.602 -5.072 11.639 0.00 0.00 H

ATOM 424 CZ2 TRP X 24 -4.104 -4.201 14.573 0.00 0.00 C

ATOM 425 HZ2 TRP X 24 -4.943 -4.572 15.151 0.00 0.00 H

ATOM 426 CH2 TRP X 24 -3.869 -4.844 13.350 0.00 0.00 C

ATOM 427 HH2 TRP X 24 -4.556 -5.623 13.045 0.00 0.00 H

ATOM 428 C TRP X 24 1.532 -2.852 14.634 0.00 0.00 C

ATOM 429 O TRP X 24 1.399 -3.771 15.457 0.00 0.00 O

ATOM 430 N ILE X 25 1.951 -3.132 13.307 0.00 0.00 N

ATOM 431 HN ILE X 25 2.049 -2.280 12.788 0.00 0.00 H

ATOM 432 CA ILE X 25 2.222 -4.420 12.666 0.00 0.00 C

ATOM 433 HA ILE X 25 1.355 -5.059 12.755 0.00 0.00 H

ATOM 434 CB ILE X 25 2.644 -4.440 11.202 0.00 0.00 C

ATOM 435 HB ILE X 25 3.595 -3.884 11.067 0.00 0.00 H

ATOM 436 CG2 ILE X 25 2.865 -5.884 10.545 0.00 0.00 C

ATOM 437 HG21 ILE X 25 3.815 -6.174 11.041 0.00 0.00 H

ATOM 438 HG22 ILE X 25 3.182 -5.822 9.477 0.00 0.00 H

ATOM 439 HG23 ILE X 25 2.053 -6.642 10.648 0.00 0.00 H

ATOM 440 CG1 ILE X 25 1.614 -3.515 10.352 0.00 0.00 C

ATOM 441 HG11 ILE X 25 2.109 -3.193 9.417 0.00 0.00 H

ATOM 442 HG12 ILE X 25 1.576 -2.545 10.889 0.00 0.00 H

ATOM 443 CD ILE X 25 0.334 -4.168 10.054 0.00 0.00 C

ATOM 444 HD1 ILE X 25 -0.335 -3.483 9.492 0.00 0.00 H

ATOM 445 HD2 ILE X 25 -0.247 -4.518 10.932 0.00 0.00 H

ATOM 446 HD3 ILE X 25 0.552 -5.056 9.411 0.00 0.00 H

ATOM 447 C ILE X 25 3.202 -5.121 13.455 0.00 0.00 C

ATOM 448 O ILE X 25 3.026 -6.295 13.867 0.00 0.00 O

ATOM 449 N VAL X 26 4.395 -4.537 13.758 0.00 0.00 N

ATOM 450 HN VAL X 26 4.633 -3.609 13.460 0.00 0.00 H

ATOM 451 CA VAL X 26 5.424 -5.219 14.531 0.00 0.00 C

ATOM 452 HA VAL X 26 5.425 -6.219 14.123 0.00 0.00 H

ATOM 453 CB VAL X 26 6.782 -4.556 14.262 0.00 0.00 C

ATOM 454 HB VAL X 26 7.547 -5.199 14.761 0.00 0.00 H

ATOM 455 CG1 VAL X 26 6.974 -4.390 12.709 0.00 0.00 C

ATOM 456 HG11 VAL X 26 6.904 -5.339 12.139 0.00 0.00 H

ATOM 457 HG12 VAL X 26 7.996 -4.003 12.503 0.00 0.00 H

ATOM 458 HG13 VAL X 26 6.324 -3.571 12.322 0.00 0.00 H

ATOM 459 CG2 VAL X 26 6.976 -3.199 14.828 0.00 0.00 C

ATOM 460 HG21 VAL X 26 7.849 -2.616 14.481 0.00 0.00 H

ATOM 461 HG22 VAL X 26 7.047 -3.141 15.941 0.00 0.00 H

ATOM 462 HG23 VAL X 26 6.100 -2.533 14.678 0.00 0.00 H

ATOM 463 C VAL X 26 5.114 -5.382 15.979 0.00 0.00 C

ATOM 464 O VAL X 26 5.530 -6.216 16.776 0.00 0.00 O

ATOM 465 N GLY X 27 4.253 -4.485 16.552 0.00 0.00 N

ATOM 466 HN GLY X 27 3.796 -3.760 16.040 0.00 0.00 H

ATOM 467 CA GLY X 27 3.803 -4.650 17.955 0.00 0.00 C

ATOM 468 HA1 GLY X 27 3.205 -3.791 18.216 0.00 0.00 H

ATOM 469 HA2 GLY X 27 4.748 -4.824 18.443 0.00 0.00 H

ATOM 470 C GLY X 27 3.001 -5.870 18.080 0.00 0.00 C

ATOM 471 O GLY X 27 3.347 -6.762 18.882 0.00 0.00 O

ATOM 472 N GLN X 28 1.920 -5.992 17.173 0.00 0.00 N

ATOM 473 HN GLN X 28 1.648 -5.280 16.542 0.00 0.00 H

ATOM 474 CA GLN X 28 1.033 -7.179 17.327 0.00 0.00 C

ATOM 475 HA GLN X 28 0.594 -7.178 18.307 0.00 0.00 H

ATOM 476 CB GLN X 28 -0.152 -7.186 16.271 0.00 0.00 C

ATOM 477 HB1 GLN X 28 -0.746 -8.119 16.147 0.00 0.00 H

ATOM 478 HB2 GLN X 28 0.442 -7.064 15.339 0.00 0.00 H

ATOM 479 CG GLN X 28 -1.151 -6.043 16.398 0.00 0.00 C

ATOM 480 HG1 GLN X 28 -1.771 -6.112 15.488 0.00 0.00 H

ATOM 481 HG2 GLN X 28 -0.566 -5.105 16.420 0.00 0.00 H

ATOM 482 CD GLN X 28 -1.944 -6.122 17.718 0.00 0.00 C

ATOM 483 OE1 GLN X 28 -1.640 -6.985 18.494 0.00 0.00 O

ATOM 484 NE2 GLN X 28 -2.875 -5.094 18.002 0.00 0.00 N

ATOM 485 HE21 GLN X 28 -3.447 -5.255 18.807 0.00 0.00 H

ATOM 486 HE22 GLN X 28 -2.979 -4.390 17.299 0.00 0.00 H

ATOM 487 C GLN X 28 1.657 -8.547 17.113 0.00 0.00 C

ATOM 488 O GLN X 28 1.317 -9.488 17.814 0.00 0.00 O

ATOM 489 N ALA X 29 2.646 -8.619 16.207 0.00 0.00 N

ATOM 490 HN ALA X 29 3.103 -7.826 15.801 0.00 0.00 H

ATOM 491 CA ALA X 29 3.119 -9.891 15.839 0.00 0.00 C

ATOM 492 HA ALA X 29 2.376 -10.625 16.130 0.00 0.00 H

ATOM 493 CB ALA X 29 3.389 -10.025 14.366 0.00 0.00 C

ATOM 494 HB1 ALA X 29 2.461 -9.764 13.824 0.00 0.00 H

ATOM 495 HB2 ALA X 29 3.665 -11.067 14.082 0.00 0.00 H

ATOM 496 HB3 ALA X 29 4.226 -9.329 14.138 0.00 0.00 H

ATOM 497 C ALA X 29 4.471 -10.223 16.543 0.00 0.00 C

ATOM 498 O ALA X 29 4.863 -11.396 16.583 0.00 0.00 O

ATOM 499 N GLY X 30 5.137 -9.194 17.173 0.00 0.00 N

ATOM 500 HN GLY X 30 4.715 -8.291 17.248 0.00 0.00 H

ATOM 501 CA GLY X 30 6.518 -9.368 17.647 0.00 0.00 C

ATOM 502 HA1 GLY X 30 6.570 -10.316 18.160 0.00 0.00 H

ATOM 503 HA2 GLY X 30 6.756 -8.590 18.358 0.00 0.00 H

ATOM 504 C GLY X 30 7.583 -9.432 16.608 0.00 0.00 C

ATOM 505 O GLY X 30 7.479 -9.697 15.375 0.00 0.00 O

ATOM 506 N ILE X 31 8.825 -9.317 17.130 0.00 0.00 N

ATOM 507 HN ILE X 31 9.002 -9.042 18.078 0.00 0.00 H

ATOM 508 CA ILE X 31 9.950 -9.367 16.157 0.00 0.00 C

ATOM 509 HA ILE X 31 9.725 -8.657 15.379 0.00 0.00 H

ATOM 510 CB ILE X 31 11.303 -8.945 16.791 0.00 0.00 C

ATOM 511 HB ILE X 31 11.568 -9.579 17.658 0.00 0.00 H

ATOM 512 CG2 ILE X 31 12.392 -9.027 15.766 0.00 0.00 C

ATOM 513 HG21 ILE X 31 12.037 -8.304 15.003 0.00 0.00 H

ATOM 514 HG22 ILE X 31 12.623 -10.003 15.291 0.00 0.00 H

ATOM 515 HG23 ILE X 31 13.334 -8.736 16.293 0.00 0.00 H

ATOM 516 CG1 ILE X 31 11.273 -7.480 17.285 0.00 0.00 C

ATOM 517 HG11 ILE X 31 10.478 -7.324 18.045 0.00 0.00 H

ATOM 518 HG12 ILE X 31 11.068 -6.792 16.430 0.00 0.00 H

ATOM 519 CD ILE X 31 12.579 -6.856 17.860 0.00 0.00 C

ATOM 520 HD1 ILE X 31 12.846 -7.595 18.654 0.00 0.00 H

ATOM 521 HD2 ILE X 31 12.584 -5.809 18.224 0.00 0.00 H

ATOM 522 HD3 ILE X 31 13.397 -6.887 17.104 0.00 0.00 H

ATOM 523 C ILE X 31 10.155 -10.716 15.311 0.00 0.00 C

ATOM 524 O ILE X 31 10.245 -10.716 14.091 0.00 0.00 O

ATOM 525 N GLY X 32 10.025 -11.889 15.939 0.00 0.00 N

ATOM 526 HN GLY X 32 9.710 -11.871 16.882 0.00 0.00 H

ATOM 527 CA GLY X 32 10.332 -13.161 15.331 0.00 0.00 C

ATOM 528 HA1 GLY X 32 10.203 -13.887 16.122 0.00 0.00 H

ATOM 529 HA2 GLY X 32 11.319 -13.008 14.915 0.00 0.00 H

ATOM 530 C GLY X 32 9.336 -13.537 14.270 0.00 0.00 C

ATOM 531 O GLY X 32 9.690 -14.051 13.234 0.00 0.00 O

ATOM 532 N LEU X 33 7.989 -13.281 14.479 0.00 0.00 N

ATOM 533 HN LEU X 33 7.580 -13.045 15.356 0.00 0.00 H

ATOM 534 CA LEU X 33 7.005 -13.473 13.386 0.00 0.00 C

ATOM 535 HA LEU X 33 7.308 -14.352 12.832 0.00 0.00 H

ATOM 536 CB LEU X 33 5.679 -13.802 14.160 0.00 0.00 C

ATOM 537 HB1 LEU X 33 5.302 -12.857 14.591 0.00 0.00 H

ATOM 538 HB2 LEU X 33 5.875 -14.311 15.133 0.00 0.00 H

ATOM 539 CG LEU X 33 4.480 -14.514 13.402 0.00 0.00 C

ATOM 540 HG LEU X 33 4.381 -13.938 12.455 0.00 0.00 H

ATOM 541 CD1 LEU X 33 4.872 -15.977 13.098 0.00 0.00 C

ATOM 542 HD11 LEU X 33 5.791 -16.115 12.481 0.00 0.00 H

ATOM 543 HD12 LEU X 33 4.068 -16.485 12.520 0.00 0.00 H

ATOM 544 HD13 LEU X 33 4.991 -16.580 14.018 0.00 0.00 H

ATOM 545 CD2 LEU X 33 3.281 -14.581 14.324 0.00 0.00 C

ATOM 546 HD21 LEU X 33 3.635 -15.034 15.279 0.00 0.00 H

ATOM 547 HD22 LEU X 33 2.458 -15.267 14.037 0.00 0.00 H

ATOM 548 HD23 LEU X 33 2.935 -13.586 14.694 0.00 0.00 H

ATOM 549 C LEU X 33 6.979 -12.425 12.346 0.00 0.00 C

ATOM 550 O LEU X 33 6.552 -12.651 11.205 0.00 0.00 O

ATOM 551 N SER X 34 7.481 -11.243 12.604 0.00 0.00 N

ATOM 552 HN SER X 34 7.680 -11.002 13.558 0.00 0.00 H

ATOM 553 CA SER X 34 7.407 -10.100 11.694 0.00 0.00 C

ATOM 554 HA SER X 34 6.446 -10.010 11.210 0.00 0.00 H

ATOM 555 CB SER X 34 7.910 -8.794 12.317 0.00 0.00 C

ATOM 556 HB1 SER X 34 8.085 -7.996 11.558 0.00 0.00 H

ATOM 557 HB2 SER X 34 8.925 -8.817 12.762 0.00 0.00 H

ATOM 558 OG SER X 34 6.993 -8.238 13.229 0.00 0.00 O

ATOM 559 HG1 SER X 34 7.040 -8.925 13.894 0.00 0.00 H

ATOM 560 C SER X 34 8.237 -10.422 10.445 0.00 0.00 C

ATOM 561 O SER X 34 7.892 -10.056 9.334 0.00 0.00 O

ATOM 562 N VAL X 35 9.378 -11.181 10.650 0.00 0.00 N

ATOM 563 HN VAL X 35 9.835 -11.262 11.532 0.00 0.00 H

ATOM 564 CA VAL X 35 10.159 -11.860 9.591 0.00 0.00 C

ATOM 565 HA VAL X 35 10.448 -11.029 8.962 0.00 0.00 H

ATOM 566 CB VAL X 35 11.424 -12.545 10.087 0.00 0.00 C

ATOM 567 HB VAL X 35 11.155 -13.144 10.984 0.00 0.00 H

ATOM 568 CG1 VAL X 35 12.132 -13.321 8.935 0.00 0.00 C

ATOM 569 HG11 VAL X 35 13.166 -13.474 9.320 0.00 0.00 H

ATOM 570 HG12 VAL X 35 12.157 -12.666 8.048 0.00 0.00 H

ATOM 571 HG13 VAL X 35 11.669 -14.288 8.646 0.00 0.00 H

ATOM 572 CG2 VAL X 35 12.373 -11.445 10.652 0.00 0.00 C

ATOM 573 HG21 VAL X 35 13.039 -11.065 9.842 0.00 0.00 H

ATOM 574 HG22 VAL X 35 13.099 -11.889 11.375 0.00 0.00 H

ATOM 575 HG23 VAL X 35 11.879 -10.582 11.135 0.00 0.00 H

ATOM 576 C VAL X 35 9.349 -12.952 8.849 0.00 0.00 C

ATOM 577 O VAL X 35 9.356 -13.023 7.614 0.00 0.00 O

ATOM 578 N LEU X 36 8.553 -13.736 9.550 0.00 0.00 N

ATOM 579 HN LEU X 36 8.496 -13.566 10.531 0.00 0.00 H

ATOM 580 CA LEU X 36 7.687 -14.770 8.889 0.00 0.00 C

ATOM 581 HA LEU X 36 8.296 -15.416 8.283 0.00 0.00 H

ATOM 582 CB LEU X 36 7.126 -15.706 9.979 0.00 0.00 C

ATOM 583 HB1 LEU X 36 6.263 -15.287 10.522 0.00 0.00 H

ATOM 584 HB2 LEU X 36 7.876 -15.943 10.772 0.00 0.00 H

ATOM 585 CG LEU X 36 6.810 -17.118 9.433 0.00 0.00 C

ATOM 586 HG LEU X 36 6.144 -17.032 8.548 0.00 0.00 H

ATOM 587 CD1 LEU X 36 8.032 -17.905 9.226 0.00 0.00 C

ATOM 588 HD11 LEU X 36 8.570 -17.628 8.288 0.00 0.00 H

ATOM 589 HD12 LEU X 36 7.740 -18.953 9.011 0.00 0.00 H

ATOM 590 HD13 LEU X 36 8.726 -17.903 10.088 0.00 0.00 H

ATOM 591 CD2 LEU X 36 5.975 -18.028 10.380 0.00 0.00 C

ATOM 592 HD21 LEU X 36 5.152 -17.294 10.554 0.00 0.00 H

ATOM 593 HD22 LEU X 36 6.421 -18.159 11.385 0.00 0.00 H

ATOM 594 HD23 LEU X 36 5.685 -18.962 9.846 0.00 0.00 H

ATOM 595 C LEU X 36 6.568 -14.159 8.031 0.00 0.00 C

ATOM 596 O LEU X 36 6.270 -14.694 7.006 0.00 0.00 O

ATOM 597 N VAL X 37 6.003 -13.061 8.461 0.00 0.00 N

ATOM 598 HN VAL X 37 6.363 -12.634 9.278 0.00 0.00 H

ATOM 599 CA VAL X 37 5.068 -12.323 7.715 0.00 0.00 C

ATOM 600 HA VAL X 37 4.228 -12.974 7.505 0.00 0.00 H

ATOM 601 CB VAL X 37 4.432 -11.254 8.623 0.00 0.00 C

ATOM 602 HB VAL X 37 5.217 -10.658 9.143 0.00 0.00 H

ATOM 603 CG1 VAL X 37 3.429 -10.288 7.855 0.00 0.00 C

ATOM 604 HG11 VAL X 37 2.826 -9.696 8.566 0.00 0.00 H

ATOM 605 HG12 VAL X 37 2.804 -10.811 7.095 0.00 0.00 H

ATOM 606 HG13 VAL X 37 3.984 -9.469 7.337 0.00 0.00 H

ATOM 607 CG2 VAL X 37 3.680 -11.866 9.832 0.00 0.00 C

ATOM 608 HG21 VAL X 37 2.651 -12.178 9.569 0.00 0.00 H

ATOM 609 HG22 VAL X 37 3.423 -11.054 10.556 0.00 0.00 H

ATOM 610 HG23 VAL X 37 4.265 -12.591 10.428 0.00 0.00 H

ATOM 611 C VAL X 37 5.560 -11.729 6.409 0.00 0.00 C

ATOM 612 O VAL X 37 5.030 -11.917 5.345 0.00 0.00 O

ATOM 613 N ILE X 38 6.714 -11.036 6.559 0.00 0.00 N

ATOM 614 HN ILE X 38 7.028 -10.726 7.450 0.00 0.00 H

ATOM 615 CA ILE X 38 7.531 -10.620 5.451 0.00 0.00 C

ATOM 616 HA ILE X 38 6.967 -9.974 4.802 0.00 0.00 H

ATOM 617 CB ILE X 38 8.662 -9.624 5.972 0.00 0.00 C

ATOM 618 HB ILE X 38 9.159 -9.999 6.888 0.00 0.00 H

ATOM 619 CG2 ILE X 38 9.751 -9.455 4.920 0.00 0.00 C

ATOM 620 HG21 ILE X 38 10.589 -8.746 5.126 0.00 0.00 H

ATOM 621 HG22 ILE X 38 9.253 -9.130 3.988 0.00 0.00 H

ATOM 622 HG23 ILE X 38 10.349 -10.380 4.795 0.00 0.00 H

ATOM 623 CG1 ILE X 38 8.117 -8.270 6.398 0.00 0.00 C

ATOM 624 HG11 ILE X 38 7.174 -8.498 6.935 0.00 0.00 H

ATOM 625 HG12 ILE X 38 7.835 -7.594 5.566 0.00 0.00 H

ATOM 626 CD ILE X 38 8.996 -7.493 7.329 0.00 0.00 C

ATOM 627 HD1 ILE X 38 10.050 -7.244 7.105 0.00 0.00 H

ATOM 628 HD2 ILE X 38 8.990 -7.974 8.338 0.00 0.00 H

ATOM 629 HD3 ILE X 38 8.451 -6.546 7.529 0.00 0.00 H

ATOM 630 C ILE X 38 8.102 -11.719 4.558 0.00 0.00 C

ATOM 631 O ILE X 38 8.028 -11.528 3.328 0.00 0.00 O

ATOM 632 N MET X 39 8.568 -12.834 5.096 0.00 0.00 N

ATOM 633 HN MET X 39 8.848 -12.963 6.043 0.00 0.00 H

ATOM 634 CA MET X 39 8.617 -14.120 4.320 0.00 0.00 C

ATOM 635 HA MET X 39 9.402 -14.049 3.584 0.00 0.00 H

ATOM 636 CB MET X 39 9.225 -15.165 5.283 0.00 0.00 C

ATOM 637 HB1 MET X 39 9.081 -16.180 4.850 0.00 0.00 H

ATOM 638 HB2 MET X 39 8.554 -15.187 6.169 0.00 0.00 H

ATOM 639 CG MET X 39 10.688 -15.010 5.724 0.00 0.00 C

ATOM 640 HG1 MET X 39 10.878 -15.525 6.686 0.00 0.00 H

ATOM 641 HG2 MET X 39 10.965 -13.953 5.906 0.00 0.00 H

ATOM 642 SD MET X 39 11.947 -15.648 4.503 0.00 0.00 S

ATOM 643 CE MET X 39 12.425 -14.144 3.628 0.00 0.00 C

ATOM 644 HE1 MET X 39 12.283 -13.325 4.365 0.00 0.00 H

ATOM 645 HE2 MET X 39 11.698 -14.044 2.786 0.00 0.00 H

ATOM 646 HE3 MET X 39 13.433 -14.327 3.216 0.00 0.00 H

ATOM 647 C MET X 39 7.475 -14.504 3.556 0.00 0.00 C

ATOM 648 O MET X 39 7.496 -14.835 2.380 0.00 0.00 O

ATOM 649 N MET X 40 6.261 -14.682 4.181 0.00 0.00 N

ATOM 650 HN MET X 40 6.112 -14.572 5.166 0.00 0.00 H

ATOM 651 CA MET X 40 5.101 -14.984 3.399 0.00 0.00 C

ATOM 652 HA MET X 40 5.316 -15.895 2.868 0.00 0.00 H

ATOM 653 CB MET X 40 3.874 -15.126 4.434 0.00 0.00 C

ATOM 654 HB1 MET X 40 4.030 -14.281 5.151 0.00 0.00 H

ATOM 655 HB2 MET X 40 4.137 -15.971 5.103 0.00 0.00 H

ATOM 656 CG MET X 40 2.465 -15.327 3.830 0.00 0.00 C

ATOM 657 HG1 MET X 40 2.102 -14.404 3.336 0.00 0.00 H

ATOM 658 HG2 MET X 40 1.774 -15.513 4.684 0.00 0.00 H

ATOM 659 SD MET X 40 2.313 -16.686 2.623 0.00 0.00 S

ATOM 660 CE MET X 40 0.582 -16.595 2.448 0.00 0.00 C

ATOM 661 HE1 MET X 40 0.192 -15.609 2.098 0.00 0.00 H

ATOM 662 HE2 MET X 40 0.057 -16.627 3.432 0.00 0.00 H

ATOM 663 HE3 MET X 40 0.194 -17.267 1.654 0.00 0.00 H

ATOM 664 C MET X 40 4.728 -13.970 2.303 0.00 0.00 C

ATOM 665 O MET X 40 4.312 -14.304 1.201 0.00 0.00 O

ATOM 666 N ALA X 41 4.943 -12.682 2.523 0.00 0.00 N

ATOM 667 HN ALA X 41 5.161 -12.395 3.455 0.00 0.00 H

ATOM 668 CA ALA X 41 4.755 -11.665 1.518 0.00 0.00 C

ATOM 669 HA ALA X 41 3.733 -11.842 1.227 0.00 0.00 H

ATOM 670 CB ALA X 41 4.843 -10.206 1.925 0.00 0.00 C

ATOM 671 HB1 ALA X 41 5.870 -9.970 2.263 0.00 0.00 H

ATOM 672 HB2 ALA X 41 4.097 -10.001 2.717 0.00 0.00 H

ATOM 673 HB3 ALA X 41 4.726 -9.589 1.004 0.00 0.00 H

ATOM 674 C ALA X 41 5.761 -11.824 0.328 0.00 0.00 C

ATOM 675 O ALA X 41 5.405 -11.545 -0.829 0.00 0.00 O

ATOM 676 N THR X 42 6.967 -12.240 0.608 0.00 0.00 N

ATOM 677 HN THR X 42 7.160 -12.525 1.545 0.00 0.00 H

ATOM 678 CA THR X 42 8.032 -12.653 -0.337 0.00 0.00 C

ATOM 679 HA THR X 42 8.293 -11.831 -0.988 0.00 0.00 H

ATOM 680 CB THR X 42 9.344 -12.838 0.393 0.00 0.00 C

ATOM 681 HB THR X 42 9.213 -13.613 1.186 0.00 0.00 H

ATOM 682 OG1 THR X 42 9.703 -11.604 1.015 0.00 0.00 O

ATOM 683 HG1 THR X 42 9.217 -11.572 1.837 0.00 0.00 H

ATOM 684 CG2 THR X 42 10.614 -13.294 -0.457 0.00 0.00 C

ATOM 685 HG21 THR X 42 10.879 -12.523 -1.225 0.00 0.00 H

ATOM 686 HG22 THR X 42 10.476 -14.278 -0.951 0.00 0.00 H

ATOM 687 HG23 THR X 42 11.430 -13.250 0.295 0.00 0.00 H

ATOM 688 C THR X 42 7.597 -13.808 -1.155 0.00 0.00 C

ATOM 689 O THR X 42 7.710 -13.878 -2.378 0.00 0.00 O

ATOM 690 N VAL X 43 6.907 -14.812 -0.549 0.00 0.00 N

ATOM 691 HN VAL X 43 6.676 -14.759 0.421 0.00 0.00 H

ATOM 692 CA VAL X 43 6.542 -16.012 -1.245 0.00 0.00 C

ATOM 693 HA VAL X 43 7.365 -16.445 -1.781 0.00 0.00 H

ATOM 694 CB VAL X 43 6.202 -17.038 -0.206 0.00 0.00 C

ATOM 695 HB VAL X 43 5.460 -16.635 0.513 0.00 0.00 H

ATOM 696 CG1 VAL X 43 5.587 -18.307 -0.975 0.00 0.00 C

ATOM 697 HG11 VAL X 43 5.478 -19.255 -0.400 0.00 0.00 H

ATOM 698 HG12 VAL X 43 6.332 -18.548 -1.765 0.00 0.00 H

ATOM 699 HG13 VAL X 43 4.552 -18.020 -1.258 0.00 0.00 H

ATOM 700 CG2 VAL X 43 7.448 -17.489 0.585 0.00 0.00 C

ATOM 701 HG21 VAL X 43 8.247 -17.950 -0.043 0.00 0.00 H

ATOM 702 HG22 VAL X 43 7.086 -18.288 1.274 0.00 0.00 H

ATOM 703 HG23 VAL X 43 7.912 -16.634 1.113 0.00 0.00 H

ATOM 704 C VAL X 43 5.511 -15.905 -2.312 0.00 0.00 C

ATOM 705 O VAL X 43 5.632 -16.426 -3.414 0.00 0.00 O

ATOM 706 N VAL X 44 4.330 -15.157 -2.148 0.00 0.00 N

ATOM 707 HN VAL X 44 4.019 -14.761 -1.291 0.00 0.00 H

ATOM 708 CA VAL X 44 3.370 -14.823 -3.142 0.00 0.00 C

ATOM 709 HA VAL X 44 3.199 -15.798 -3.590 0.00 0.00 H

ATOM 710 CB VAL X 44 2.033 -14.335 -2.705 0.00 0.00 C

ATOM 711 HB VAL X 44 1.363 -13.897 -3.473 0.00 0.00 H

ATOM 712 CG1 VAL X 44 1.351 -15.560 -1.972 0.00 0.00 C

ATOM 713 HG11 VAL X 44 0.369 -15.217 -1.588 0.00 0.00 H

ATOM 714 HG12 VAL X 44 1.969 -16.081 -1.205 0.00 0.00 H

ATOM 715 HG13 VAL X 44 1.208 -16.428 -2.656 0.00 0.00 H

ATOM 716 CG2 VAL X 44 2.087 -13.158 -1.638 0.00 0.00 C

ATOM 717 HG21 VAL X 44 2.761 -12.365 -2.041 0.00 0.00 H

ATOM 718 HG22 VAL X 44 2.573 -13.465 -0.698 0.00 0.00 H

ATOM 719 HG23 VAL X 44 1.082 -12.726 -1.454 0.00 0.00 H

ATOM 720 C VAL X 44 3.992 -13.978 -4.229 0.00 0.00 C

ATOM 721 O VAL X 44 3.752 -14.207 -5.419 0.00 0.00 O

ATOM 722 N THR X 45 4.890 -13.036 -3.851 0.00 0.00 N

ATOM 723 HN THR X 45 5.062 -12.918 -2.872 0.00 0.00 H

ATOM 724 CA THR X 45 5.687 -12.165 -4.719 0.00 0.00 C

ATOM 725 HA THR X 45 4.916 -11.684 -5.308 0.00 0.00 H

ATOM 726 CB THR X 45 6.358 -11.061 -4.003 0.00 0.00 C

ATOM 727 HB THR X 45 7.217 -11.355 -3.365 0.00 0.00 H

ATOM 728 OG1 THR X 45 5.460 -10.251 -3.268 0.00 0.00 O

ATOM 729 HG1 THR X 45 5.254 -10.618 -2.399 0.00 0.00 H

ATOM 730 CG2 THR X 45 7.053 -10.098 -5.019 0.00 0.00 C

ATOM 731 HG21 THR X 45 6.384 -9.990 -5.906 0.00 0.00 H

ATOM 732 HG22 THR X 45 7.998 -10.527 -5.422 0.00 0.00 H

ATOM 733 HG23 THR X 45 7.215 -9.092 -4.574 0.00 0.00 H

ATOM 734 C THR X 45 6.660 -12.819 -5.648 0.00 0.00 C

ATOM 735 O THR X 45 6.648 -12.596 -6.853 0.00 0.00 O

ATOM 736 N THR X 46 7.545 -13.740 -5.208 0.00 0.00 N

ATOM 737 HN THR X 46 7.472 -14.095 -4.275 0.00 0.00 H

ATOM 738 CA THR X 46 8.588 -14.546 -5.998 0.00 0.00 C

ATOM 739 HA THR X 46 9.089 -13.768 -6.550 0.00 0.00 H

ATOM 740 CB THR X 46 9.546 -15.249 -5.030 0.00 0.00 C

ATOM 741 HB THR X 46 10.025 -16.063 -5.610 0.00 0.00 H

ATOM 742 OG1 THR X 46 8.861 -15.848 -3.876 0.00 0.00 O

ATOM 743 HG1 THR X 46 8.735 -15.090 -3.305 0.00 0.00 H

ATOM 744 CG2 THR X 46 10.625 -14.276 -4.604 0.00 0.00 C

ATOM 745 HG21 THR X 46 10.243 -13.470 -3.943 0.00 0.00 H

ATOM 746 HG22 THR X 46 11.224 -13.833 -5.425 0.00 0.00 H

ATOM 747 HG23 THR X 46 11.405 -14.689 -3.927 0.00 0.00 H

ATOM 748 C THR X 46 7.930 -15.591 -6.958 0.00 0.00 C

ATOM 749 O THR X 46 8.465 -15.837 -7.982 0.00 0.00 O

ATOM 750 N ILE X 47 6.754 -16.249 -6.569 0.00 0.00 N

ATOM 751 HN ILE X 47 6.538 -16.271 -5.603 0.00 0.00 H

ATOM 752 CA ILE X 47 6.017 -17.013 -7.567 0.00 0.00 C

ATOM 753 HA ILE X 47 6.537 -17.783 -8.114 0.00 0.00 H

ATOM 754 CB ILE X 47 4.869 -17.948 -6.954 0.00 0.00 C

ATOM 755 HB ILE X 47 4.101 -17.442 -6.336 0.00 0.00 H

ATOM 756 CG2 ILE X 47 4.151 -18.723 -8.120 0.00 0.00 C

ATOM 757 HG21 ILE X 47 3.702 -19.607 -7.627 0.00 0.00 H

ATOM 758 HG22 ILE X 47 4.877 -19.025 -8.908 0.00 0.00 H

ATOM 759 HG23 ILE X 47 3.398 -17.958 -8.405 0.00 0.00 H

ATOM 760 CG1 ILE X 47 5.520 -18.934 -5.941 0.00 0.00 C

ATOM 761 HG11 ILE X 47 6.025 -18.287 -5.193 0.00 0.00 H

ATOM 762 HG12 ILE X 47 6.276 -19.535 -6.494 0.00 0.00 H

ATOM 763 CD ILE X 47 4.520 -19.787 -5.106 0.00 0.00 C

ATOM 764 HD1 ILE X 47 3.717 -19.140 -4.692 0.00 0.00 H

ATOM 765 HD2 ILE X 47 5.060 -20.136 -4.197 0.00 0.00 H

ATOM 766 HD3 ILE X 47 3.952 -20.578 -5.630 0.00 0.00 H

ATOM 767 C ILE X 47 5.333 -16.187 -8.613 0.00 0.00 C

ATOM 768 O ILE X 47 5.590 -16.452 -9.838 0.00 0.00 O

ATOM 769 N THR X 48 4.638 -15.139 -8.275 0.00 0.00 N

ATOM 770 HN THR X 48 4.380 -15.080 -7.309 0.00 0.00 H

ATOM 771 CA THR X 48 3.964 -14.174 -9.239 0.00 0.00 C

ATOM 772 HA THR X 48 3.391 -14.784 -9.917 0.00 0.00 H

ATOM 773 CB THR X 48 2.963 -13.131 -8.601 0.00 0.00 C

ATOM 774 HB THR X 48 3.506 -12.443 -7.912 0.00 0.00 H

ATOM 775 OG1 THR X 48 1.963 -13.784 -7.800 0.00 0.00 O

ATOM 776 HG1 THR X 48 2.499 -14.023 -7.040 0.00 0.00 H

ATOM 777 CG2 THR X 48 2.296 -12.254 -9.710 0.00 0.00 C

ATOM 778 HG21 THR X 48 1.619 -11.554 -9.175 0.00 0.00 H

ATOM 779 HG22 THR X 48 1.818 -12.789 -10.563 0.00 0.00 H

ATOM 780 HG23 THR X 48 3.097 -11.579 -10.091 0.00 0.00 H

ATOM 781 C THR X 48 4.975 -13.636 -10.132 0.00 0.00 C

ATOM 782 O THR X 48 4.678 -13.319 -11.305 0.00 0.00 O

ATOM 783 N GLY X 49 6.298 -13.459 -9.637 0.00 0.00 N

ATOM 784 HN GLY X 49 6.450 -13.633 -8.661 0.00 0.00 H

ATOM 785 CA GLY X 49 7.384 -12.870 -10.354 0.00 0.00 C

ATOM 786 HA1 GLY X 49 8.056 -12.486 -9.600 0.00 0.00 H

ATOM 787 HA2 GLY X 49 7.003 -12.215 -11.125 0.00 0.00 H

ATOM 788 C GLY X 49 8.084 -13.780 -11.296 0.00 0.00 C

ATOM 789 O GLY X 49 8.521 -13.395 -12.362 0.00 0.00 O

ATOM 790 N LEU X 50 8.226 -15.062 -10.923 0.00 0.00 N

ATOM 791 HN LEU X 50 8.008 -15.345 -10.001 0.00 0.00 H

ATOM 792 CA LEU X 50 8.484 -16.081 -11.916 0.00 0.00 C

ATOM 793 HA LEU X 50 9.328 -15.704 -12.474 0.00 0.00 H

ATOM 794 CB LEU X 50 8.713 -17.390 -11.119 0.00 0.00 C

ATOM 795 HB1 LEU X 50 7.800 -17.615 -10.528 0.00 0.00 H

ATOM 796 HB2 LEU X 50 9.533 -17.261 -10.382 0.00 0.00 H

ATOM 797 CG LEU X 50 9.296 -18.634 -11.809 0.00 0.00 C

ATOM 798 HG LEU X 50 8.614 -18.904 -12.652 0.00 0.00 H

ATOM 799 CD1 LEU X 50 10.768 -18.550 -12.259 0.00 0.00 C

ATOM 800 HD11 LEU X 50 11.237 -18.606 -11.256 0.00 0.00 H

ATOM 801 HD12 LEU X 50 11.131 -17.721 -12.909 0.00 0.00 H

ATOM 802 HD13 LEU X 50 11.149 -19.417 -12.835 0.00 0.00 H

ATOM 803 CD2 LEU X 50 9.071 -19.854 -10.886 0.00 0.00 C

ATOM 804 HD21 LEU X 50 7.999 -19.867 -10.619 0.00 0.00 H

ATOM 805 HD22 LEU X 50 9.700 -19.708 -9.980 0.00 0.00 H

ATOM 806 HD23 LEU X 50 9.213 -20.776 -11.494 0.00 0.00 H

ATOM 807 C LEU X 50 7.441 -16.328 -13.042 0.00 0.00 C

ATOM 808 O LEU X 50 7.802 -16.446 -14.224 0.00 0.00 O

ATOM 809 N SER X 51 6.117 -16.259 -12.717 0.00 0.00 N

ATOM 810 HN SER X 51 5.778 -16.367 -11.779 0.00 0.00 H

ATOM 811 CA SER X 51 5.066 -16.110 -13.739 0.00 0.00 C

ATOM 812 HA SER X 51 5.019 -16.981 -14.382 0.00 0.00 H

ATOM 813 CB SER X 51 3.679 -16.210 -13.107 0.00 0.00 C

ATOM 814 HB1 SER X 51 2.900 -16.076 -13.887 0.00 0.00 H

ATOM 815 HB2 SER X 51 3.448 -15.280 -12.539 0.00 0.00 H

ATOM 816 OG SER X 51 3.441 -17.440 -12.457 0.00 0.00 O

ATOM 817 HG1 SER X 51 3.085 -17.080 -11.642 0.00 0.00 H

ATOM 818 C SER X 51 5.183 -14.926 -14.661 0.00 0.00 C

ATOM 819 O SER X 51 4.988 -14.970 -15.869 0.00 0.00 O

ATOM 820 N THR X 52 5.543 -13.652 -14.102 0.00 0.00 N

ATOM 821 HN THR X 52 5.482 -13.711 -13.107 0.00 0.00 H

ATOM 822 CA THR X 52 5.851 -12.432 -14.815 0.00 0.00 C

ATOM 823 HA THR X 52 5.012 -12.134 -15.430 0.00 0.00 H

ATOM 824 CB THR X 52 6.136 -11.283 -13.842 0.00 0.00 C

ATOM 825 HB THR X 52 7.162 -11.339 -13.413 0.00 0.00 H

ATOM 826 OG1 THR X 52 5.135 -11.123 -12.933 0.00 0.00 O

ATOM 827 HG1 THR X 52 5.011 -11.935 -12.429 0.00 0.00 H

ATOM 828 CG2 THR X 52 5.993 -10.054 -14.755 0.00 0.00 C

ATOM 829 HG21 THR X 52 5.020 -9.942 -15.282 0.00 0.00 H

ATOM 830 HG22 THR X 52 6.792 -9.966 -15.521 0.00 0.00 H

ATOM 831 HG23 THR X 52 6.183 -9.121 -14.182 0.00 0.00 H

ATOM 832 C THR X 52 7.077 -12.704 -15.600 0.00 0.00 C

ATOM 833 O THR X 52 7.247 -12.194 -16.664 0.00 0.00 O

ATOM 834 N SER X 53 8.002 -13.401 -15.131 0.00 0.00 N

ATOM 835 HN SER X 53 8.024 -13.790 -14.205 0.00 0.00 H

ATOM 836 CA SER X 53 9.148 -13.834 -15.995 0.00 0.00 C

ATOM 837 HA SER X 53 9.596 -12.923 -16.380 0.00 0.00 H

ATOM 838 CB SER X 53 10.296 -14.471 -15.122 0.00 0.00 C

ATOM 839 HB1 SER X 53 10.228 -15.541 -14.836 0.00 0.00 H

ATOM 840 HB2 SER X 53 10.496 -13.871 -14.202 0.00 0.00 H

ATOM 841 OG SER X 53 11.568 -14.372 -15.816 0.00 0.00 O

ATOM 842 HG1 SER X 53 11.826 -15.245 -16.103 0.00 0.00 H

ATOM 843 C SER X 53 8.845 -14.793 -17.174 0.00 0.00 C

ATOM 844 O SER X 53 9.190 -14.583 -18.360 0.00 0.00 O

ATOM 845 N ALA X 54 7.953 -15.783 -16.964 0.00 0.00 N

ATOM 846 HN ALA X 54 7.718 -15.989 -16.018 0.00 0.00 H

ATOM 847 CA ALA X 54 7.282 -16.615 -17.899 0.00 0.00 C

ATOM 848 HA ALA X 54 8.067 -17.196 -18.360 0.00 0.00 H

ATOM 849 CB ALA X 54 6.438 -17.623 -17.098 0.00 0.00 C

ATOM 850 HB1 ALA X 54 7.097 -18.231 -16.451 0.00 0.00 H

ATOM 851 HB2 ALA X 54 5.803 -18.248 -17.761 0.00 0.00 H

ATOM 852 HB3 ALA X 54 5.893 -16.936 -16.416 0.00 0.00 H

ATOM 853 C ALA X 54 6.546 -15.919 -19.024 0.00 0.00 C

ATOM 854 O ALA X 54 6.795 -16.164 -20.150 0.00 0.00 O

ATOM 855 N ILE X 55 5.755 -14.806 -18.629 0.00 0.00 N

ATOM 856 HN ILE X 55 5.573 -14.827 -17.640 0.00 0.00 H

ATOM 857 CA ILE X 55 5.066 -14.029 -19.653 0.00 0.00 C

ATOM 858 HA ILE X 55 4.698 -14.718 -20.404 0.00 0.00 H

ATOM 859 CB ILE X 55 3.869 -13.256 -19.140 0.00 0.00 C

ATOM 860 HB ILE X 55 3.425 -12.580 -19.898 0.00 0.00 H

ATOM 861 CG2 ILE X 55 2.625 -14.138 -18.904 0.00 0.00 C

ATOM 862 HG21 ILE X 55 2.294 -14.612 -19.857 0.00 0.00 H

ATOM 863 HG22 ILE X 55 1.847 -13.576 -18.351 0.00 0.00 H

ATOM 864 HG23 ILE X 55 2.927 -14.969 -18.242 0.00 0.00 H

ATOM 865 CG1 ILE X 55 4.285 -12.468 -17.907 0.00 0.00 C

ATOM 866 HG11 ILE X 55 5.209 -11.872 -18.057 0.00 0.00 H

ATOM 867 HG12 ILE X 55 4.328 -13.167 -17.049 0.00 0.00 H

ATOM 868 CD ILE X 55 3.242 -11.284 -17.706 0.00 0.00 C

ATOM 869 HD1 ILE X 55 3.396 -10.558 -18.534 0.00 0.00 H

ATOM 870 HD2 ILE X 55 3.472 -10.690 -16.803 0.00 0.00 H

ATOM 871 HD3 ILE X 55 2.200 -11.680 -17.642 0.00 0.00 H

ATOM 872 C ILE X 55 6.090 -13.199 -20.423 0.00 0.00 C

ATOM 873 O ILE X 55 6.025 -13.039 -21.661 0.00 0.00 O

ATOM 874 N ALA X 56 7.151 -12.636 -19.730 0.00 0.00 N

ATOM 875 HN ALA X 56 7.367 -12.831 -18.776 0.00 0.00 H

ATOM 876 CA ALA X 56 7.989 -11.670 -20.368 0.00 0.00 C

ATOM 877 HA ALA X 56 7.432 -10.919 -20.912 0.00 0.00 H

ATOM 878 CB ALA X 56 8.824 -10.693 -19.354 0.00 0.00 C

ATOM 879 HB1 ALA X 56 9.087 -9.905 -20.083 0.00 0.00 H

ATOM 880 HB2 ALA X 56 9.669 -11.155 -18.808 0.00 0.00 H

ATOM 881 HB3 ALA X 56 8.100 -10.321 -18.601 0.00 0.00 H

ATOM 882 C ALA X 56 9.110 -12.217 -21.328 0.00 0.00 C

ATOM 883 O ALA X 56 9.596 -11.478 -22.171 0.00 0.00 O

ATOM 884 N THR X 57 9.408 -13.491 -21.211 0.00 0.00 N

ATOM 885 HN THR X 57 9.172 -14.006 -20.396 0.00 0.00 H

ATOM 886 CA THR X 57 10.504 -14.151 -21.905 0.00 0.00 C

ATOM 887 HA THR X 57 11.050 -13.311 -22.314 0.00 0.00 H

ATOM 888 CB THR X 57 11.394 -15.048 -21.053 0.00 0.00 C

ATOM 889 HB THR X 57 12.061 -15.650 -21.706 0.00 0.00 H

ATOM 890 OG1 THR X 57 10.604 -15.965 -20.372 0.00 0.00 O

ATOM 891 HG1 THR X 57 9.800 -15.558 -20.033 0.00 0.00 H

ATOM 892 CG2 THR X 57 12.156 -14.185 -19.988 0.00 0.00 C

ATOM 893 HG21 THR X 57 12.799 -14.933 -19.485 0.00 0.00 H

ATOM 894 HG22 THR X 57 11.379 -13.798 -19.303 0.00 0.00 H

ATOM 895 HG23 THR X 57 12.781 -13.447 -20.544 0.00 0.00 H

ATOM 896 C THR X 57 9.990 -14.868 -23.113 0.00 0.00 C

ATOM 897 O THR X 57 10.739 -15.512 -23.856 0.00 0.00 O

ATOM 898 N ASN X 58 8.724 -14.799 -23.508 0.00 0.00 N

ATOM 899 HN ASN X 58 8.145 -14.212 -22.945 0.00 0.00 H

ATOM 900 CA ASN X 58 8.039 -15.439 -24.558 0.00 0.00 C

ATOM 901 HA ASN X 58 8.783 -15.869 -25.215 0.00 0.00 H

ATOM 902 CB ASN X 58 7.218 -16.497 -23.791 0.00 0.00 C

ATOM 903 HB1 ASN X 58 6.689 -17.167 -24.500 0.00 0.00 H

ATOM 904 HB2 ASN X 58 6.530 -16.108 -23.005 0.00 0.00 H

ATOM 905 CG ASN X 58 8.093 -17.551 -23.122 0.00 0.00 C

ATOM 906 OD1 ASN X 58 9.120 -17.945 -23.659 0.00 0.00 O

ATOM 907 ND2 ASN X 58 7.739 -18.049 -21.958 0.00 0.00 N

ATOM 908 HD21 ASN X 58 7.113 -17.558 -21.343 0.00 0.00 H

ATOM 909 HD22 ASN X 58 8.125 -18.873 -21.559 0.00 0.00 H

ATOM 910 C ASN X 58 7.128 -14.442 -25.198 0.00 0.00 C

ATOM 911 O ASN X 58 6.138 -13.919 -24.695 0.00 0.00 O

ATOM 912 N GLY X 59 7.417 -14.231 -26.461 0.00 0.00 N

ATOM 913 HN GLY X 59 8.252 -14.568 -26.881 0.00 0.00 H

ATOM 914 CA GLY X 59 6.570 -13.374 -27.376 0.00 0.00 C

ATOM 915 HA1 GLY X 59 5.536 -13.285 -27.081 0.00 0.00 H

ATOM 916 HA2 GLY X 59 6.604 -13.850 -28.349 0.00 0.00 H

ATOM 917 C GLY X 59 7.121 -11.908 -27.484 0.00 0.00 C

ATOM 918 O GLY X 59 8.236 -11.604 -27.161 0.00 0.00 O

ATOM 919 N PHE X 60 6.311 -11.020 -28.176 0.00 0.00 N

ATOM 920 HN PHE X 60 5.346 -11.224 -28.318 0.00 0.00 H

ATOM 921 CA PHE X 60 6.792 -9.690 -28.403 0.00 0.00 C

ATOM 922 HA PHE X 60 7.865 -9.805 -28.460 0.00 0.00 H

ATOM 923 CB PHE X 60 6.333 -9.272 -29.801 0.00 0.00 C

ATOM 924 HB1 PHE X 60 5.291 -9.653 -29.890 0.00 0.00 H

ATOM 925 HB2 PHE X 60 6.948 -9.768 -30.578 0.00 0.00 H

ATOM 926 CG PHE X 60 6.220 -7.743 -30.016 0.00 0.00 C

ATOM 927 CD1 PHE X 60 5.011 -7.022 -30.085 0.00 0.00 C

ATOM 928 HD1 PHE X 60 4.104 -7.613 -30.064 0.00 0.00 H

ATOM 929 CE1 PHE X 60 4.904 -5.677 -30.347 0.00 0.00 C

ATOM 930 HE1 PHE X 60 3.961 -5.148 -30.455 0.00 0.00 H

ATOM 931 CZ PHE X 60 6.101 -5.010 -30.594 0.00 0.00 C

ATOM 932 HZ PHE X 60 6.151 -3.965 -30.889 0.00 0.00 H

ATOM 933 CD2 PHE X 60 7.417 -6.994 -30.197 0.00 0.00 C

ATOM 934 HD2 PHE X 60 8.392 -7.449 -30.232 0.00 0.00 H

ATOM 935 CE2 PHE X 60 7.334 -5.637 -30.579 0.00 0.00 C

ATOM 936 HE2 PHE X 60 8.267 -5.093 -30.704 0.00 0.00 H

ATOM 937 C PHE X 60 6.330 -8.796 -27.242 0.00 0.00 C

ATOM 938 O PHE X 60 5.109 -8.545 -27.060 0.00 0.00 O

ATOM 939 N VAL X 61 7.205 -8.347 -26.390 0.00 0.00 N

ATOM 940 HN VAL X 61 8.123 -8.724 -26.366 0.00 0.00 H

ATOM 941 CA VAL X 61 6.907 -7.423 -25.274 0.00 0.00 C

ATOM 942 HA VAL X 61 5.863 -7.544 -25.048 0.00 0.00 H

ATOM 943 CB VAL X 61 7.875 -7.678 -24.093 0.00 0.00 C

ATOM 944 HB VAL X 61 8.919 -7.526 -24.443 0.00 0.00 H

ATOM 945 CG1 VAL X 61 7.491 -6.566 -23.050 0.00 0.00 C

ATOM 946 HG11 VAL X 61 7.933 -5.584 -23.329 0.00 0.00 H

ATOM 947 HG12 VAL X 61 7.735 -6.777 -21.991 0.00 0.00 H

ATOM 948 HG13 VAL X 61 6.392 -6.489 -23.135 0.00 0.00 H

ATOM 949 CG2 VAL X 61 7.579 -9.071 -23.469 0.00 0.00 C

ATOM 950 HG21 VAL X 61 8.184 -9.317 -22.570 0.00 0.00 H

ATOM 951 HG22 VAL X 61 8.006 -9.794 -24.191 0.00 0.00 H

ATOM 952 HG23 VAL X 61 6.507 -9.156 -23.197 0.00 0.00 H

ATOM 953 C VAL X 61 7.192 -6.023 -25.800 0.00 0.00 C

ATOM 954 O VAL X 61 8.407 -5.654 -25.843 0.00 0.00 O

ATOM 955 N ARG X 62 6.103 -5.222 -25.952 0.00 0.00 N

ATOM 956 HN ARG X 62 5.188 -5.597 -25.825 0.00 0.00 H

ATOM 957 CA ARG X 62 6.185 -3.793 -26.078 0.00 0.00 C

ATOM 958 HA ARG X 62 7.175 -3.492 -26.403 0.00 0.00 H

ATOM 959 CB ARG X 62 5.106 -3.294 -27.146 0.00 0.00 C

ATOM 960 HB1 ARG X 62 4.184 -3.922 -27.064 0.00 0.00 H

ATOM 961 HB2 ARG X 62 5.620 -3.390 -28.122 0.00 0.00 H

ATOM 962 CG ARG X 62 4.661 -1.798 -27.133 0.00 0.00 C

ATOM 963 HG1 ARG X 62 4.317 -1.510 -26.111 0.00 0.00 H

ATOM 964 HG2 ARG X 62 3.925 -1.615 -27.941 0.00 0.00 H

ATOM 965 CD ARG X 62 5.735 -0.778 -27.477 0.00 0.00 C

ATOM 966 HD1 ARG X 62 5.949 -0.812 -28.570 0.00 0.00 H

ATOM 967 HD2 ARG X 62 6.610 -1.128 -26.884 0.00 0.00 H

ATOM 968 NE ARG X 62 5.209 0.590 -27.292 0.00 0.00 N

ATOM 969 HE ARG X 62 4.272 0.865 -27.090 0.00 0.00 H

ATOM 970 CZ ARG X 62 5.968 1.713 -27.213 0.00 0.00 C

ATOM 971 NH1 ARG X 62 7.185 1.663 -27.581 0.00 0.00 N1+

ATOM 972 HH11 ARG X 62 7.589 0.786 -27.822 0.00 0.00 H

ATOM 973 HH12 ARG X 62 7.708 2.509 -27.490 0.00 0.00 H

ATOM 974 NH2 ARG X 62 5.435 2.691 -26.590 0.00 0.00 N

ATOM 975 HH21 ARG X 62 4.485 2.568 -26.313 0.00 0.00 H

ATOM 976 HH22 ARG X 62 6.001 3.517 -26.584 0.00 0.00 H

ATOM 977 C ARG X 62 5.993 -3.174 -24.709 0.00 0.00 C

ATOM 978 O ARG X 62 6.680 -2.175 -24.478 0.00 0.00 O

ATOM 979 N GLY X 63 5.099 -3.635 -23.828 0.00 0.00 N

ATOM 980 HN GLY X 63 4.515 -4.410 -24.072 0.00 0.00 H

ATOM 981 CA GLY X 63 4.720 -2.937 -22.645 0.00 0.00 C

ATOM 982 HA1 GLY X 63 5.649 -2.639 -22.171 0.00 0.00 H

ATOM 983 HA2 GLY X 63 4.165 -3.591 -21.987 0.00 0.00 H

ATOM 984 C GLY X 63 3.890 -1.732 -22.967 0.00 0.00 C

ATOM 985 O GLY X 63 2.921 -1.747 -23.727 0.00 0.00 O

ATOM 986 N GLY X 64 4.213 -0.586 -22.363 0.00 0.00 N

ATOM 987 HN GLY X 64 5.058 -0.565 -21.829 0.00 0.00 H

ATOM 988 CA GLY X 64 3.407 0.642 -22.371 0.00 0.00 C

ATOM 989 HA1 GLY X 64 2.893 0.775 -23.303 0.00 0.00 H

ATOM 990 HA2 GLY X 64 4.058 1.487 -22.205 0.00 0.00 H

ATOM 991 C GLY X 64 2.483 0.703 -21.246 0.00 0.00 C

ATOM 992 O GLY X 64 1.512 1.485 -21.313 0.00 0.00 O

ATOM 993 N GLY X 65 2.921 0.059 -20.147 0.00 0.00 N

ATOM 994 HN GLY X 65 3.710 -0.548 -20.202 0.00 0.00 H

ATOM 995 CA GLY X 65 1.996 -0.111 -19.017 0.00 0.00 C

ATOM 996 HA1 GLY X 65 1.079 0.450 -19.114 0.00 0.00 H

ATOM 997 HA2 GLY X 65 2.525 0.128 -18.098 0.00 0.00 H

ATOM 998 C GLY X 65 1.623 -1.565 -18.982 0.00 0.00 C

ATOM 999 O GLY X 65 1.924 -2.393 -19.851 0.00 0.00 O

ATOM 1000 N ALA X 66 1.077 -2.062 -17.849 0.00 0.00 N

ATOM 1001 HN ALA X 66 0.677 -1.447 -17.167 0.00 0.00 H

ATOM 1002 CA ALA X 66 1.052 -3.455 -17.477 0.00 0.00 C

ATOM 1003 HA ALA X 66 1.996 -3.894 -17.768 0.00 0.00 H

ATOM 1004 CB ALA X 66 0.922 -3.523 -15.863 0.00 0.00 C

ATOM 1005 HB1 ALA X 66 -0.079 -3.242 -15.477 0.00 0.00 H

ATOM 1006 HB2 ALA X 66 1.807 -2.934 -15.524 0.00 0.00 H

ATOM 1007 HB3 ALA X 66 0.997 -4.614 -15.712 0.00 0.00 H

ATOM 1008 C ALA X 66 -0.058 -4.237 -18.121 0.00 0.00 C

ATOM 1009 O ALA X 66 0.164 -5.409 -18.472 0.00 0.00 O

ATOM 1010 N TYR X 67 -1.188 -3.685 -18.391 0.00 0.00 N

ATOM 1011 HN TYR X 67 -1.286 -2.724 -18.143 0.00 0.00 H

ATOM 1012 CA TYR X 67 -2.265 -4.260 -19.096 0.00 0.00 C

ATOM 1013 HA TYR X 67 -2.535 -5.179 -18.580 0.00 0.00 H

ATOM 1014 CB TYR X 67 -3.527 -3.349 -19.022 0.00 0.00 C

ATOM 1015 HB1 TYR X 67 -3.221 -2.310 -19.279 0.00 0.00 H

ATOM 1016 HB2 TYR X 67 -3.915 -3.427 -17.980 0.00 0.00 H

ATOM 1017 CG TYR X 67 -4.681 -3.685 -19.902 0.00 0.00 C

ATOM 1018 CD1 TYR X 67 -5.588 -4.686 -19.531 0.00 0.00 C

ATOM 1019 HD1 TYR X 67 -5.387 -5.331 -18.690 0.00 0.00 H

ATOM 1020 CE1 TYR X 67 -6.592 -5.051 -20.382 0.00 0.00 C

ATOM 1021 HE1 TYR X 67 -7.307 -5.837 -20.166 0.00 0.00 H

ATOM 1022 CZ TYR X 67 -6.769 -4.406 -21.594 0.00 0.00 C

ATOM 1023 OH TYR X 67 -7.747 -4.874 -22.526 0.00 0.00 O

ATOM 1024 HH TYR X 67 -7.509 -4.570 -23.398 0.00 0.00 H

ATOM 1025 CD2 TYR X 67 -4.794 -2.989 -21.140 0.00 0.00 C

ATOM 1026 HD2 TYR X 67 -4.134 -2.172 -21.394 0.00 0.00 H

ATOM 1027 CE2 TYR X 67 -5.898 -3.365 -22.022 0.00 0.00 C

ATOM 1028 HE2 TYR X 67 -5.984 -2.930 -23.008 0.00 0.00 H

ATOM 1029 C TYR X 67 -1.955 -4.725 -20.533 0.00 0.00 C

ATOM 1030 O TYR X 67 -2.281 -5.812 -20.950 0.00 0.00 O

ATOM 1031 N TYR X 68 -1.217 -3.887 -21.264 0.00 0.00 N

ATOM 1032 HN TYR X 68 -1.077 -2.968 -20.897 0.00 0.00 H

ATOM 1033 CA TYR X 68 -0.679 -4.127 -22.611 0.00 0.00 C

ATOM 1034 HA TYR X 68 -1.553 -4.482 -23.147 0.00 0.00 H

ATOM 1035 CB TYR X 68 0.083 -2.890 -23.205 0.00 0.00 C

ATOM 1036 HB1 TYR X 68 0.822 -3.251 -23.947 0.00 0.00 H

ATOM 1037 HB2 TYR X 68 0.629 -2.343 -22.416 0.00 0.00 H

ATOM 1038 CG TYR X 68 -1.012 -2.061 -23.732 0.00 0.00 C

ATOM 1039 CD1 TYR X 68 -1.506 -1.068 -22.925 0.00 0.00 C

ATOM 1040 HD1 TYR X 68 -1.188 -0.848 -21.917 0.00 0.00 H

ATOM 1041 CE1 TYR X 68 -2.512 -0.217 -23.383 0.00 0.00 C

ATOM 1042 HE1 TYR X 68 -2.838 0.619 -22.769 0.00 0.00 H

ATOM 1043 CZ TYR X 68 -2.932 -0.381 -24.719 0.00 0.00 C

ATOM 1044 OH TYR X 68 -4.109 0.254 -25.121 0.00 0.00 O

ATOM 1045 HH TYR X 68 -4.607 -0.432 -25.574 0.00 0.00 H

ATOM 1046 CD2 TYR X 68 -1.359 -2.241 -25.099 0.00 0.00 C

ATOM 1047 HD2 TYR X 68 -1.056 -3.120 -25.653 0.00 0.00 H

ATOM 1048 CE2 TYR X 68 -2.426 -1.411 -25.503 0.00 0.00 C

ATOM 1049 HE2 TYR X 68 -2.898 -1.698 -26.432 0.00 0.00 H

ATOM 1050 C TYR X 68 0.179 -5.374 -22.646 0.00 0.00 C

ATOM 1051 O TYR X 68 -0.053 -6.326 -23.324 0.00 0.00 O

ATOM 1052 N LEU X 69 1.121 -5.452 -21.631 0.00 0.00 N

ATOM 1053 HN LEU X 69 1.254 -4.727 -20.969 0.00 0.00 H

ATOM 1054 CA LEU X 69 1.992 -6.602 -21.379 0.00 0.00 C

ATOM 1055 HA LEU X 69 2.488 -6.836 -22.308 0.00 0.00 H

ATOM 1056 CB LEU X 69 3.116 -6.078 -20.303 0.00 0.00 C

ATOM 1057 HB1 LEU X 69 2.568 -5.502 -19.534 0.00 0.00 H

ATOM 1058 HB2 LEU X 69 3.864 -5.420 -20.810 0.00 0.00 H

ATOM 1059 CG LEU X 69 3.845 -7.280 -19.569 0.00 0.00 C

ATOM 1060 HG LEU X 69 3.129 -8.045 -19.193 0.00 0.00 H

ATOM 1061 CD1 LEU X 69 4.800 -7.991 -20.557 0.00 0.00 C

ATOM 1062 HD11 LEU X 69 4.224 -8.510 -21.352 0.00 0.00 H

ATOM 1063 HD12 LEU X 69 5.505 -8.733 -20.139 0.00 0.00 H

ATOM 1064 HD13 LEU X 69 5.398 -7.247 -21.126 0.00 0.00 H

ATOM 1065 CD2 LEU X 69 4.657 -6.789 -18.364 0.00 0.00 C

ATOM 1066 HD21 LEU X 69 5.221 -7.647 -17.933 0.00 0.00 H

ATOM 1067 HD22 LEU X 69 4.055 -6.238 -17.610 0.00 0.00 H

ATOM 1068 HD23 LEU X 69 5.370 -6.038 -18.739 0.00 0.00 H

ATOM 1069 C LEU X 69 1.265 -7.870 -21.001 0.00 0.00 C

ATOM 1070 O LEU X 69 1.538 -8.993 -21.512 0.00 0.00 O

ATOM 1071 N ILE X 70 0.311 -7.767 -20.034 0.00 0.00 N

ATOM 1072 HN ILE X 70 0.322 -6.876 -19.598 0.00 0.00 H

ATOM 1073 CA ILE X 70 -0.633 -8.843 -19.591 0.00 0.00 C

ATOM 1074 HA ILE X 70 -0.093 -9.765 -19.411 0.00 0.00 H

ATOM 1075 CB ILE X 70 -1.389 -8.342 -18.366 0.00 0.00 C

ATOM 1076 HB ILE X 70 -1.442 -7.238 -18.435 0.00 0.00 H

ATOM 1077 CG2 ILE X 70 -2.882 -8.708 -18.182 0.00 0.00 C

ATOM 1078 HG21 ILE X 70 -3.135 -9.778 -18.048 0.00 0.00 H

ATOM 1079 HG22 ILE X 70 -3.529 -8.437 -19.047 0.00 0.00 H

ATOM 1080 HG23 ILE X 70 -3.256 -8.112 -17.325 0.00 0.00 H

ATOM 1081 CG1 ILE X 70 -0.439 -8.783 -17.215 0.00 0.00 C

ATOM 1082 HG11 ILE X 70 0.575 -8.346 -17.285 0.00 0.00 H

ATOM 1083 HG12 ILE X 70 -0.363 -9.890 -17.103 0.00 0.00 H

ATOM 1084 CD ILE X 70 -0.923 -8.286 -15.849 0.00 0.00 C

ATOM 1085 HD1 ILE X 70 -1.356 -7.286 -16.064 0.00 0.00 H

ATOM 1086 HD2 ILE X 70 -0.084 -8.363 -15.118 0.00 0.00 H

ATOM 1087 HD3 ILE X 70 -1.676 -8.952 -15.373 0.00 0.00 H

ATOM 1088 C ILE X 70 -1.637 -9.245 -20.622 0.00 0.00 C

ATOM 1089 O ILE X 70 -2.014 -10.430 -20.715 0.00 0.00 O

ATOM 1090 N SER X 71 -2.133 -8.444 -21.526 0.00 0.00 N

ATOM 1091 HN SER X 71 -1.852 -7.487 -21.478 0.00 0.00 H

ATOM 1092 CA SER X 71 -3.214 -8.693 -22.393 0.00 0.00 C

ATOM 1093 HA SER X 71 -3.923 -9.431 -22.038 0.00 0.00 H

ATOM 1094 CB SER X 71 -4.113 -7.465 -22.653 0.00 0.00 C

ATOM 1095 HB1 SER X 71 -4.525 -7.273 -21.643 0.00 0.00 H

ATOM 1096 HB2 SER X 71 -5.004 -7.733 -23.267 0.00 0.00 H

ATOM 1097 OG SER X 71 -3.382 -6.318 -23.134 0.00 0.00 O

ATOM 1098 HG1 SER X 71 -2.980 -6.008 -22.314 0.00 0.00 H

ATOM 1099 C SER X 71 -2.771 -9.182 -23.774 0.00 0.00 C

ATOM 1100 O SER X 71 -3.411 -9.922 -24.460 0.00 0.00 O

ATOM 1101 N ARG X 72 -1.558 -8.879 -24.196 0.00 0.00 N

ATOM 1102 HN ARG X 72 -1.010 -8.396 -23.523 0.00 0.00 H

ATOM 1103 CA ARG X 72 -1.007 -9.382 -25.403 0.00 0.00 C

ATOM 1104 HA ARG X 72 -1.764 -9.850 -26.009 0.00 0.00 H

ATOM 1105 CB ARG X 72 -0.366 -8.377 -26.388 0.00 0.00 C

ATOM 1106 HB1 ARG X 72 0.103 -8.976 -27.204 0.00 0.00 H

ATOM 1107 HB2 ARG X 72 0.531 -7.887 -25.950 0.00 0.00 H

ATOM 1108 CG ARG X 72 -1.232 -7.252 -27.021 0.00 0.00 C

ATOM 1109 HG1 ARG X 72 -1.966 -7.672 -27.738 0.00 0.00 H

ATOM 1110 HG2 ARG X 72 -0.549 -6.614 -27.624 0.00 0.00 H

ATOM 1111 CD ARG X 72 -2.084 -6.353 -26.044 0.00 0.00 C

ATOM 1112 HD1 ARG X 72 -1.365 -5.797 -25.408 0.00 0.00 H

ATOM 1113 HD2 ARG X 72 -2.919 -6.978 -25.661 0.00 0.00 H

ATOM 1114 NE ARG X 72 -2.648 -5.312 -26.901 0.00 0.00 N

ATOM 1115 HE ARG X 72 -2.294 -5.193 -27.828 0.00 0.00 H

ATOM 1116 CZ ARG X 72 -3.901 -4.878 -26.643 0.00 0.00 C

ATOM 1117 NH1 ARG X 72 -4.498 -5.110 -25.453 0.00 0.00 N1+

ATOM 1118 HH11 ARG X 72 -3.944 -5.256 -24.637 0.00 0.00 H

ATOM 1119 HH12 ARG X 72 -5.459 -4.837 -25.404 0.00 0.00 H

ATOM 1120 NH2 ARG X 72 -4.531 -4.098 -27.499 0.00 0.00 N

ATOM 1121 HH21 ARG X 72 -4.155 -3.837 -28.383 0.00 0.00 H

ATOM 1122 HH22 ARG X 72 -5.497 -3.940 -27.293 0.00 0.00 H

ATOM 1123 C ARG X 72 -0.122 -10.592 -25.125 0.00 0.00 C

ATOM 1124 O ARG X 72 0.567 -11.064 -25.997 0.00 0.00 O

ATOM 1125 N SER X 73 -0.266 -11.178 -23.862 0.00 0.00 N

ATOM 1126 HN SER X 73 -0.779 -10.720 -23.152 0.00 0.00 H

ATOM 1127 CA SER X 73 0.235 -12.505 -23.538 0.00 0.00 C

ATOM 1128 HA SER X 73 0.681 -12.808 -24.467 0.00 0.00 H

ATOM 1129 CB SER X 73 1.380 -12.440 -22.470 0.00 0.00 C

ATOM 1130 HB1 SER X 73 2.292 -12.044 -22.961 0.00 0.00 H

ATOM 1131 HB2 SER X 73 1.688 -13.461 -22.157 0.00 0.00 H

ATOM 1132 OG SER X 73 1.070 -11.683 -21.284 0.00 0.00 O

ATOM 1133 HG1 SER X 73 1.382 -10.775 -21.391 0.00 0.00 H

ATOM 1134 C SER X 73 -0.816 -13.450 -23.197 0.00 0.00 C

ATOM 1135 O SER X 73 -1.400 -14.104 -23.973 0.00 0.00 O

ATOM 1136 N LEU X 74 -1.259 -13.332 -21.882 0.00 0.00 N

ATOM 1137 HN LEU X 74 -0.705 -12.758 -21.285 0.00 0.00 H

ATOM 1138 CA LEU X 74 -2.315 -14.102 -21.220 0.00 0.00 C

ATOM 1139 HA LEU X 74 -2.053 -15.141 -21.334 0.00 0.00 H

ATOM 1140 CB LEU X 74 -2.402 -13.574 -19.793 0.00 0.00 C

ATOM 1141 HB1 LEU X 74 -3.125 -14.168 -19.207 0.00 0.00 H

ATOM 1142 HB2 LEU X 74 -2.737 -12.515 -19.708 0.00 0.00 H

ATOM 1143 CG LEU X 74 -1.063 -13.789 -18.977 0.00 0.00 C

ATOM 1144 HG LEU X 74 -0.263 -13.258 -19.520 0.00 0.00 H

ATOM 1145 CD1 LEU X 74 -1.167 -13.251 -17.573 0.00 0.00 C

ATOM 1146 HD11 LEU X 74 -1.485 -12.192 -17.738 0.00 0.00 H

ATOM 1147 HD12 LEU X 74 -0.214 -13.424 -17.034 0.00 0.00 H

ATOM 1148 HD13 LEU X 74 -1.943 -13.785 -16.987 0.00 0.00 H

ATOM 1149 CD2 LEU X 74 -0.638 -15.280 -18.943 0.00 0.00 C

ATOM 1150 HD21 LEU X 74 -1.219 -15.852 -18.182 0.00 0.00 H

ATOM 1151 HD22 LEU X 74 0.417 -15.475 -18.623 0.00 0.00 H

ATOM 1152 HD23 LEU X 74 -0.829 -15.818 -19.896 0.00 0.00 H

ATOM 1153 C LEU X 74 -3.679 -13.994 -21.820 0.00 0.00 C

ATOM 1154 O LEU X 74 -4.514 -14.820 -21.402 0.00 0.00 O

ATOM 1155 N GLY X 75 -4.028 -13.071 -22.726 0.00 0.00 N

ATOM 1156 HN GLY X 75 -3.295 -12.423 -22.890 0.00 0.00 H

ATOM 1157 CA GLY X 75 -5.309 -13.052 -23.502 0.00 0.00 C

ATOM 1158 HA1 GLY X 75 -5.525 -14.062 -23.801 0.00 0.00 H

ATOM 1159 HA2 GLY X 75 -5.052 -12.523 -24.406 0.00 0.00 H

ATOM 1160 C GLY X 75 -6.538 -12.424 -22.831 0.00 0.00 C

ATOM 1161 O GLY X 75 -6.425 -12.298 -21.597 0.00 0.00 O

ATOM 1162 N PRO X 76 -7.643 -12.088 -23.468 0.00 0.00 N

ATOM 1163 CD PRO X 76 -7.673 -12.119 -24.941 0.00 0.00 C

ATOM 1164 HD1 PRO X 76 -7.292 -13.072 -25.350 0.00 0.00 H

ATOM 1165 HD2 PRO X 76 -7.193 -11.200 -25.332 0.00 0.00 H

ATOM 1166 CA PRO X 76 -8.684 -11.208 -22.934 0.00 0.00 C

ATOM 1167 HA PRO X 76 -8.238 -10.358 -22.448 0.00 0.00 H

ATOM 1168 CB PRO X 76 -9.572 -10.921 -24.179 0.00 0.00 C

ATOM 1169 HB1 PRO X 76 -9.340 -9.934 -24.633 0.00 0.00 H

ATOM 1170 HB2 PRO X 76 -10.665 -10.824 -23.982 0.00 0.00 H

ATOM 1171 CG PRO X 76 -9.151 -11.905 -25.267 0.00 0.00 C

ATOM 1172 HG1 PRO X 76 -9.671 -12.868 -25.089 0.00 0.00 H

ATOM 1173 HG2 PRO X 76 -9.141 -11.535 -26.309 0.00 0.00 H

ATOM 1174 C PRO X 76 -9.553 -11.779 -21.832 0.00 0.00 C

ATOM 1175 O PRO X 76 -10.162 -10.963 -21.136 0.00 0.00 O

ATOM 1176 N GLU X 77 -9.554 -13.135 -21.651 0.00 0.00 N

ATOM 1177 HN GLU X 77 -8.940 -13.687 -22.213 0.00 0.00 H

ATOM 1178 CA GLU X 77 -10.510 -13.916 -20.766 0.00 0.00 C

ATOM 1179 HA GLU X 77 -11.475 -13.503 -20.999 0.00 0.00 H

ATOM 1180 CB GLU X 77 -10.279 -15.445 -20.861 0.00 0.00 C

ATOM 1181 HB1 GLU X 77 -10.651 -15.966 -19.950 0.00 0.00 H

ATOM 1182 HB2 GLU X 77 -9.178 -15.607 -20.871 0.00 0.00 H

ATOM 1183 CG GLU X 77 -11.024 -16.180 -22.043 0.00 0.00 C

ATOM 1184 HG1 GLU X 77 -10.760 -15.557 -22.922 0.00 0.00 H

ATOM 1185 HG2 GLU X 77 -12.130 -16.164 -21.933 0.00 0.00 H

ATOM 1186 CD GLU X 77 -10.488 -17.559 -22.266 0.00 0.00 C

ATOM 1187 OE1 GLU X 77 -10.644 -18.527 -21.513 0.00 0.00 O

ATOM 1188 OE2 GLU X 77 -9.912 -17.732 -23.401 0.00 0.00 O1-

ATOM 1189 C GLU X 77 -10.380 -13.422 -19.323 0.00 0.00 C

ATOM 1190 O GLU X 77 -11.390 -12.893 -18.762 0.00 0.00 O

ATOM 1191 N PHE X 78 -9.157 -13.404 -18.797 0.00 0.00 N

ATOM 1192 HN PHE X 78 -8.315 -13.694 -19.261 0.00 0.00 H

ATOM 1193 CA PHE X 78 -8.828 -12.901 -17.545 0.00 0.00 C

ATOM 1194 HA PHE X 78 -9.737 -12.604 -17.049 0.00 0.00 H

ATOM 1195 CB PHE X 78 -8.027 -13.850 -16.638 0.00 0.00 C

ATOM 1196 HB1 PHE X 78 -7.905 -13.467 -15.602 0.00 0.00 H

ATOM 1197 HB2 PHE X 78 -7.049 -14.052 -17.123 0.00 0.00 H

ATOM 1198 CG PHE X 78 -8.670 -15.182 -16.690 0.00 0.00 C

ATOM 1199 CD1 PHE X 78 -9.946 -15.340 -16.053 0.00 0.00 C

ATOM 1200 HD1 PHE X 78 -10.392 -14.519 -15.491 0.00 0.00 H

ATOM 1201 CE1 PHE X 78 -10.546 -16.660 -15.960 0.00 0.00 C

ATOM 1202 HE1 PHE X 78 -11.502 -16.810 -15.500 0.00 0.00 H

ATOM 1203 CZ PHE X 78 -9.903 -17.783 -16.420 0.00 0.00 C

ATOM 1204 HZ PHE X 78 -10.450 -18.705 -16.248 0.00 0.00 H

ATOM 1205 CD2 PHE X 78 -8.120 -16.341 -17.390 0.00 0.00 C

ATOM 1206 HD2 PHE X 78 -7.122 -16.327 -17.817 0.00 0.00 H

ATOM 1207 CE2 PHE X 78 -8.693 -17.649 -17.084 0.00 0.00 C

ATOM 1208 HE2 PHE X 78 -8.245 -18.456 -17.637 0.00 0.00 H

ATOM 1209 C PHE X 78 -7.957 -11.617 -17.706 0.00 0.00 C

ATOM 1210 O PHE X 78 -7.842 -10.814 -16.840 0.00 0.00 O

ATOM 1211 N GLY X 79 -7.493 -11.361 -18.963 0.00 0.00 N

ATOM 1212 HN GLY X 79 -7.630 -11.961 -19.745 0.00 0.00 H

ATOM 1213 CA GLY X 79 -6.818 -10.033 -19.179 0.00 0.00 C

ATOM 1214 HA1 GLY X 79 -6.579 -9.947 -20.227 0.00 0.00 H

ATOM 1215 HA2 GLY X 79 -5.925 -9.946 -18.573 0.00 0.00 H

ATOM 1216 C GLY X 79 -7.745 -8.828 -18.947 0.00 0.00 C

ATOM 1217 O GLY X 79 -7.323 -7.738 -18.530 0.00 0.00 O

ATOM 1218 N GLY X 80 -9.050 -8.968 -19.171 0.00 0.00 N

ATOM 1219 HN GLY X 80 -9.346 -9.855 -19.517 0.00 0.00 H

ATOM 1220 CA GLY X 80 -10.004 -7.968 -18.978 0.00 0.00 C

ATOM 1221 HA1 GLY X 80 -10.992 -8.283 -19.287 0.00 0.00 H

ATOM 1222 HA2 GLY X 80 -9.695 -7.041 -19.450 0.00 0.00 H

ATOM 1223 C GLY X 80 -10.370 -7.544 -17.565 0.00 0.00 C

ATOM 1224 O GLY X 80 -10.570 -6.390 -17.189 0.00 0.00 O

ATOM 1225 N ALA X 81 -10.570 -8.547 -16.683 0.00 0.00 N

ATOM 1226 HN ALA X 81 -10.608 -9.504 -16.946 0.00 0.00 H

ATOM 1227 CA ALA X 81 -10.710 -8.456 -15.273 0.00 0.00 C

ATOM 1228 HA ALA X 81 -11.526 -7.787 -15.074 0.00 0.00 H

ATOM 1229 CB ALA X 81 -11.144 -9.821 -14.655 0.00 0.00 C

ATOM 1230 HB1 ALA X 81 -10.347 -10.587 -14.678 0.00 0.00 H

ATOM 1231 HB2 ALA X 81 -12.025 -10.219 -15.199 0.00 0.00 H

ATOM 1232 HB3 ALA X 81 -11.399 -9.744 -13.579 0.00 0.00 H

ATOM 1233 C ALA X 81 -9.520 -7.909 -14.534 0.00 0.00 C

ATOM 1234 O ALA X 81 -9.771 -7.079 -13.709 0.00 0.00 O

ATOM 1235 N ILE X 82 -8.256 -8.293 -14.915 0.00 0.00 N

ATOM 1236 HN ILE X 82 -8.145 -8.961 -15.635 0.00 0.00 H

ATOM 1237 CA ILE X 82 -7.114 -7.776 -14.240 0.00 0.00 C

ATOM 1238 HA ILE X 82 -7.168 -7.905 -13.169 0.00 0.00 H

ATOM 1239 CB ILE X 82 -5.723 -8.423 -14.600 0.00 0.00 C

ATOM 1240 HB ILE X 82 -5.794 -8.480 -15.713 0.00 0.00 H

ATOM 1241 CG2 ILE X 82 -4.539 -7.596 -14.072 0.00 0.00 C

ATOM 1242 HG21 ILE X 82 -4.413 -6.595 -14.528 0.00 0.00 H

ATOM 1243 HG22 ILE X 82 -3.682 -8.273 -14.266 0.00 0.00 H

ATOM 1244 HG23 ILE X 82 -4.567 -7.534 -12.970 0.00 0.00 H

ATOM 1245 CG1 ILE X 82 -5.855 -9.887 -14.053 0.00 0.00 C

ATOM 1246 HG11 ILE X 82 -6.879 -10.224 -14.344 0.00 0.00 H

ATOM 1247 HG12 ILE X 82 -5.755 -9.945 -12.950 0.00 0.00 H

ATOM 1248 CD ILE X 82 -4.709 -10.720 -14.488 0.00 0.00 C

ATOM 1249 HD1 ILE X 82 -4.523 -10.639 -15.582 0.00 0.00 H

ATOM 1250 HD2 ILE X 82 -5.058 -11.760 -14.285 0.00 0.00 H

ATOM 1251 HD3 ILE X 82 -3.675 -10.569 -14.103 0.00 0.00 H

ATOM 1252 C ILE X 82 -6.954 -6.337 -14.580 0.00 0.00 C

ATOM 1253 O ILE X 82 -6.792 -5.535 -13.678 0.00 0.00 O

ATOM 1254 N GLY X 83 -7.180 -5.988 -15.858 0.00 0.00 N

ATOM 1255 HN GLY X 83 -7.494 -6.758 -16.406 0.00 0.00 H

ATOM 1256 CA GLY X 83 -7.374 -4.569 -16.296 0.00 0.00 C

ATOM 1257 HA1 GLY X 83 -7.754 -4.625 -17.312 0.00 0.00 H

ATOM 1258 HA2 GLY X 83 -6.448 -4.039 -16.149 0.00 0.00 H

ATOM 1259 C GLY X 83 -8.350 -3.780 -15.427 0.00 0.00 C

ATOM 1260 O GLY X 83 -8.024 -2.703 -14.908 0.00 0.00 O

ATOM 1261 N LEU X 84 -9.482 -4.289 -15.224 0.00 0.00 N

ATOM 1262 HN LEU X 84 -9.781 -5.169 -15.591 0.00 0.00 H

ATOM 1263 CA LEU X 84 -10.649 -3.659 -14.405 0.00 0.00 C

ATOM 1264 HA LEU X 84 -10.706 -2.647 -14.771 0.00 0.00 H

ATOM 1265 CB LEU X 84 -11.913 -4.378 -14.637 0.00 0.00 C

ATOM 1266 HB1 LEU X 84 -11.884 -5.286 -13.998 0.00 0.00 H

ATOM 1267 HB2 LEU X 84 -11.897 -4.731 -15.690 0.00 0.00 H

ATOM 1268 CG LEU X 84 -13.238 -3.712 -14.200 0.00 0.00 C

ATOM 1269 HG LEU X 84 -13.131 -3.362 -13.153 0.00 0.00 H

ATOM 1270 CD1 LEU X 84 -13.703 -2.542 -14.954 0.00 0.00 C

ATOM 1271 HD11 LEU X 84 -13.569 -2.725 -16.046 0.00 0.00 H

ATOM 1272 HD12 LEU X 84 -13.013 -1.678 -14.835 0.00 0.00 H

ATOM 1273 HD13 LEU X 84 -14.724 -2.157 -14.768 0.00 0.00 H

ATOM 1274 CD2 LEU X 84 -14.279 -4.854 -14.198 0.00 0.00 C

ATOM 1275 HD21 LEU X 84 -13.894 -5.697 -13.575 0.00 0.00 H

ATOM 1276 HD22 LEU X 84 -14.321 -5.267 -15.223 0.00 0.00 H

ATOM 1277 HD23 LEU X 84 -15.317 -4.562 -13.907 0.00 0.00 H

ATOM 1278 C LEU X 84 -10.441 -3.513 -12.789 0.00 0.00 C

ATOM 1279 O LEU X 84 -10.723 -2.507 -12.211 0.00 0.00 O

ATOM 1280 N ILE X 85 -9.906 -4.523 -12.180 0.00 0.00 N

ATOM 1281 HN ILE X 85 -9.585 -5.283 -12.740 0.00 0.00 H

ATOM 1282 CA ILE X 85 -9.436 -4.665 -10.839 0.00 0.00 C

ATOM 1283 HA ILE X 85 -10.176 -4.299 -10.137 0.00 0.00 H

ATOM 1284 CB ILE X 85 -9.250 -6.126 -10.409 0.00 0.00 C

ATOM 1285 HB ILE X 85 -8.474 -6.528 -11.104 0.00 0.00 H

ATOM 1286 CG2 ILE X 85 -8.544 -6.217 -8.965 0.00 0.00 C

ATOM 1287 HG21 ILE X 85 -9.261 -5.704 -8.281 0.00 0.00 H

ATOM 1288 HG22 ILE X 85 -7.520 -5.795 -9.012 0.00 0.00 H

ATOM 1289 HG23 ILE X 85 -8.538 -7.262 -8.592 0.00 0.00 H

ATOM 1290 CG1 ILE X 85 -10.561 -7.013 -10.584 0.00 0.00 C

ATOM 1291 HG11 ILE X 85 -10.996 -6.740 -11.577 0.00 0.00 H

ATOM 1292 HG12 ILE X 85 -11.335 -6.782 -9.817 0.00 0.00 H

ATOM 1293 CD ILE X 85 -10.297 -8.551 -10.544 0.00 0.00 C

ATOM 1294 HD1 ILE X 85 -9.318 -8.754 -11.048 0.00 0.00 H

ATOM 1295 HD2 ILE X 85 -11.095 -9.211 -10.940 0.00 0.00 H

ATOM 1296 HD3 ILE X 85 -10.091 -8.848 -9.493 0.00 0.00 H

ATOM 1297 C ILE X 85 -8.158 -3.846 -10.562 0.00 0.00 C

ATOM 1298 O ILE X 85 -7.998 -3.177 -9.561 0.00 0.00 O

ATOM 1299 N PHE X 86 -7.234 -3.717 -11.629 0.00 0.00 N

ATOM 1300 HN PHE X 86 -7.351 -4.271 -12.442 0.00 0.00 H

ATOM 1301 CA PHE X 86 -6.077 -2.841 -11.581 0.00 0.00 C

ATOM 1302 HA PHE X 86 -5.624 -2.960 -10.607 0.00 0.00 H

ATOM 1303 CB PHE X 86 -5.021 -3.368 -12.691 0.00 0.00 C

ATOM 1304 HB1 PHE X 86 -5.470 -3.215 -13.698 0.00 0.00 H

ATOM 1305 HB2 PHE X 86 -4.866 -4.442 -12.452 0.00 0.00 H

ATOM 1306 CG PHE X 86 -3.656 -2.650 -12.509 0.00 0.00 C

ATOM 1307 CD1 PHE X 86 -2.980 -2.431 -11.220 0.00 0.00 C

ATOM 1308 HD1 PHE X 86 -3.555 -2.630 -10.332 0.00 0.00 H

ATOM 1309 CE1 PHE X 86 -1.733 -1.724 -11.161 0.00 0.00 C

ATOM 1310 HE1 PHE X 86 -1.296 -1.587 -10.185 0.00 0.00 H

ATOM 1311 CZ PHE X 86 -1.017 -1.457 -12.331 0.00 0.00 C

ATOM 1312 HZ PHE X 86 -0.008 -1.098 -12.244 0.00 0.00 H

ATOM 1313 CD2 PHE X 86 -2.943 -2.170 -13.613 0.00 0.00 C

ATOM 1314 HD2 PHE X 86 -3.462 -2.152 -14.558 0.00 0.00 H

ATOM 1315 CE2 PHE X 86 -1.661 -1.632 -13.629 0.00 0.00 C

ATOM 1316 HE2 PHE X 86 -1.091 -1.337 -14.504 0.00 0.00 H

ATOM 1317 C PHE X 86 -6.321 -1.381 -11.483 0.00 0.00 C

ATOM 1318 O PHE X 86 -5.863 -0.670 -10.664 0.00 0.00 O

ATOM 1319 N ALA X 87 -7.271 -0.917 -12.293 0.00 0.00 N

ATOM 1320 HN ALA X 87 -7.628 -1.310 -13.143 0.00 0.00 H

ATOM 1321 CA ALA X 87 -7.853 0.379 -12.191 0.00 0.00 C

ATOM 1322 HA ALA X 87 -6.973 0.990 -12.367 0.00 0.00 H

ATOM 1323 CB ALA X 87 -8.793 0.624 -13.489 0.00 0.00 C

ATOM 1324 HB1 ALA X 87 -9.155 1.672 -13.437 0.00 0.00 H

ATOM 1325 HB2 ALA X 87 -9.619 -0.116 -13.540 0.00 0.00 H

ATOM 1326 HB3 ALA X 87 -8.163 0.458 -14.397 0.00 0.00 H

ATOM 1327 C ALA X 87 -8.639 0.672 -10.925 0.00 0.00 C

ATOM 1328 O ALA X 87 -8.609 1.711 -10.295 0.00 0.00 O

ATOM 1329 N PHE X 88 -9.386 -0.291 -10.438 0.00 0.00 N

ATOM 1330 HN PHE X 88 -9.427 -1.169 -10.899 0.00 0.00 H

ATOM 1331 CA PHE X 88 -10.068 -0.238 -9.216 0.00 0.00 C

ATOM 1332 HA PHE X 88 -10.755 0.584 -9.063 0.00 0.00 H

ATOM 1333 CB PHE X 88 -10.983 -1.570 -9.187 0.00 0.00 C

ATOM 1334 HB1 PHE X 88 -10.369 -2.479 -9.362 0.00 0.00 H

ATOM 1335 HB2 PHE X 88 -11.708 -1.456 -10.019 0.00 0.00 H

ATOM 1336 CG PHE X 88 -11.675 -1.798 -7.856 0.00 0.00 C

ATOM 1337 CD1 PHE X 88 -12.947 -1.194 -7.591 0.00 0.00 C

ATOM 1338 HD1 PHE X 88 -13.289 -0.593 -8.415 0.00 0.00 H

ATOM 1339 CE1 PHE X 88 -13.638 -1.393 -6.403 0.00 0.00 C

ATOM 1340 HE1 PHE X 88 -14.576 -0.902 -6.206 0.00 0.00 H

ATOM 1341 CZ PHE X 88 -13.103 -2.274 -5.470 0.00 0.00 C

ATOM 1342 HZ PHE X 88 -13.678 -2.385 -4.558 0.00 0.00 H

ATOM 1343 CD2 PHE X 88 -11.102 -2.530 -6.817 0.00 0.00 C

ATOM 1344 HD2 PHE X 88 -10.105 -2.876 -7.038 0.00 0.00 H

ATOM 1345 CE2 PHE X 88 -11.871 -2.896 -5.677 0.00 0.00 C

ATOM 1346 HE2 PHE X 88 -11.422 -3.574 -4.958 0.00 0.00 H

ATOM 1347 C PHE X 88 -9.113 -0.039 -7.935 0.00 0.00 C

ATOM 1348 O PHE X 88 -9.414 0.717 -7.060 0.00 0.00 O

ATOM 1349 N ALA X 89 -7.965 -0.792 -7.891 0.00 0.00 N

ATOM 1350 HN ALA X 89 -7.914 -1.538 -8.542 0.00 0.00 H

ATOM 1351 CA ALA X 89 -6.956 -0.825 -6.881 0.00 0.00 C

ATOM 1352 HA ALA X 89 -7.527 -0.996 -5.985 0.00 0.00 H

ATOM 1353 CB ALA X 89 -5.869 -1.849 -7.189 0.00 0.00 C

ATOM 1354 HB1 ALA X 89 -6.293 -2.879 -7.247 0.00 0.00 H

ATOM 1355 HB2 ALA X 89 -5.040 -1.930 -6.448 0.00 0.00 H

ATOM 1356 HB3 ALA X 89 -5.502 -1.651 -8.220 0.00 0.00 H

ATOM 1357 C ALA X 89 -6.222 0.517 -6.840 0.00 0.00 C

ATOM 1358 O ALA X 89 -5.890 1.017 -5.799 0.00 0.00 O

ATOM 1359 N ASN X 90 -6.036 1.157 -8.011 0.00 0.00 N

ATOM 1360 HN ASN X 90 -6.241 0.684 -8.859 0.00 0.00 H

ATOM 1361 CA ASN X 90 -5.496 2.516 -8.134 0.00 0.00 C

ATOM 1362 HA ASN X 90 -4.656 2.624 -7.474 0.00 0.00 H

ATOM 1363 CB ASN X 90 -5.122 2.914 -9.682 0.00 0.00 C

ATOM 1364 HB1 ASN X 90 -4.997 4.020 -9.721 0.00 0.00 H

ATOM 1365 HB2 ASN X 90 -5.947 2.608 -10.356 0.00 0.00 H

ATOM 1366 CG ASN X 90 -3.710 2.461 -10.143 0.00 0.00 C

ATOM 1367 OD1 ASN X 90 -2.849 2.444 -9.261 0.00 0.00 O

ATOM 1368 ND2 ASN X 90 -3.516 1.970 -11.379 0.00 0.00 N

ATOM 1369 HD21 ASN X 90 -2.597 1.787 -11.710 0.00 0.00 H

ATOM 1370 HD22 ASN X 90 -4.301 1.662 -11.918 0.00 0.00 H

ATOM 1371 C ASN X 90 -6.460 3.528 -7.550 0.00 0.00 C

ATOM 1372 O ASN X 90 -6.044 4.621 -7.083 0.00 0.00 O

ATOM 1373 N ALA X 91 -7.771 3.350 -7.671 0.00 0.00 N

ATOM 1374 HN ALA X 91 -8.163 2.584 -8.167 0.00 0.00 H

ATOM 1375 CA ALA X 91 -8.793 4.088 -6.864 0.00 0.00 C

ATOM 1376 HA ALA X 91 -8.715 5.127 -7.156 0.00 0.00 H

ATOM 1377 CB ALA X 91 -10.155 3.731 -7.503 0.00 0.00 C

ATOM 1378 HB1 ALA X 91 -10.509 2.700 -7.302 0.00 0.00 H

ATOM 1379 HB2 ALA X 91 -10.162 3.669 -8.608 0.00 0.00 H

ATOM 1380 HB3 ALA X 91 -10.905 4.467 -7.139 0.00 0.00 H

ATOM 1381 C ALA X 91 -8.671 3.959 -5.330 0.00 0.00 C

ATOM 1382 O ALA X 91 -8.588 4.989 -4.673 0.00 0.00 O

ATOM 1383 N VAL X 92 -8.414 2.720 -4.766 0.00 0.00 N

ATOM 1384 HN VAL X 92 -8.416 1.902 -5.336 0.00 0.00 H

ATOM 1385 CA VAL X 92 -8.313 2.402 -3.362 0.00 0.00 C

ATOM 1386 HA VAL X 92 -9.062 2.938 -2.794 0.00 0.00 H

ATOM 1387 CB VAL X 92 -8.427 0.951 -3.067 0.00 0.00 C

ATOM 1388 HB VAL X 92 -7.589 0.341 -3.469 0.00 0.00 H

ATOM 1389 CG1 VAL X 92 -8.511 0.734 -1.529 0.00 0.00 C

ATOM 1390 HG11 VAL X 92 -8.822 -0.308 -1.353 0.00 0.00 H

ATOM 1391 HG12 VAL X 92 -9.280 1.357 -1.019 0.00 0.00 H

ATOM 1392 HG13 VAL X 92 -7.517 0.882 -1.058 0.00 0.00 H

ATOM 1393 CG2 VAL X 92 -9.693 0.530 -3.762 0.00 0.00 C

ATOM 1394 HG21 VAL X 92 -9.414 0.326 -4.827 0.00 0.00 H

ATOM 1395 HG22 VAL X 92 -10.559 1.207 -3.619 0.00 0.00 H

ATOM 1396 HG23 VAL X 92 -9.995 -0.412 -3.258 0.00 0.00 H

ATOM 1397 C VAL X 92 -6.999 2.893 -2.828 0.00 0.00 C

ATOM 1398 O VAL X 92 -6.798 3.314 -1.725 0.00 0.00 O

ATOM 1399 N ALA X 93 -5.928 3.079 -3.762 0.00 0.00 N

ATOM 1400 HN ALA X 93 -6.101 2.556 -4.603 0.00 0.00 H

ATOM 1401 CA ALA X 93 -4.674 3.557 -3.294 0.00 0.00 C

ATOM 1402 HA ALA X 93 -4.478 2.936 -2.435 0.00 0.00 H

ATOM 1403 CB ALA X 93 -3.670 3.332 -4.510 0.00 0.00 C

ATOM 1404 HB1 ALA X 93 -2.662 3.692 -4.233 0.00 0.00 H

ATOM 1405 HB2 ALA X 93 -4.043 3.812 -5.440 0.00 0.00 H

ATOM 1406 HB3 ALA X 93 -3.582 2.228 -4.599 0.00 0.00 H

ATOM 1407 C ALA X 93 -4.679 5.063 -3.062 0.00 0.00 C

ATOM 1408 O ALA X 93 -3.998 5.508 -2.170 0.00 0.00 O

ATOM 1409 N VAL X 94 -5.481 5.853 -3.797 0.00 0.00 N

ATOM 1410 HN VAL X 94 -5.832 5.488 -4.647 0.00 0.00 H

ATOM 1411 CA VAL X 94 -5.928 7.142 -3.437 0.00 0.00 C

ATOM 1412 HA VAL X 94 -5.034 7.745 -3.390 0.00 0.00 H

ATOM 1413 CB VAL X 94 -6.768 7.865 -4.565 0.00 0.00 C

ATOM 1414 HB VAL X 94 -7.524 7.095 -4.818 0.00 0.00 H

ATOM 1415 CG1 VAL X 94 -7.397 9.102 -4.221 0.00 0.00 C

ATOM 1416 HG11 VAL X 94 -8.215 9.132 -3.476 0.00 0.00 H

ATOM 1417 HG12 VAL X 94 -8.002 9.436 -5.096 0.00 0.00 H

ATOM 1418 HG13 VAL X 94 -6.768 9.991 -3.980 0.00 0.00 H

ATOM 1419 CG2 VAL X 94 -5.835 8.066 -5.767 0.00 0.00 C

ATOM 1420 HG21 VAL X 94 -5.632 7.141 -6.338 0.00 0.00 H

ATOM 1421 HG22 VAL X 94 -4.820 8.335 -5.391 0.00 0.00 H

ATOM 1422 HG23 VAL X 94 -6.294 8.827 -6.442 0.00 0.00 H

ATOM 1423 C VAL X 94 -6.702 7.232 -2.126 0.00 0.00 C

ATOM 1424 O VAL X 94 -6.297 8.014 -1.227 0.00 0.00 O

ATOM 1425 N ALA X 95 -7.589 6.302 -1.859 0.00 0.00 N

ATOM 1426 HN ALA X 95 -7.805 5.634 -2.574 0.00 0.00 H

ATOM 1427 CA ALA X 95 -8.252 6.236 -0.626 0.00 0.00 C

ATOM 1428 HA ALA X 95 -8.623 7.224 -0.359 0.00 0.00 H

ATOM 1429 CB ALA X 95 -9.426 5.230 -0.748 0.00 0.00 C

ATOM 1430 HB1 ALA X 95 -10.224 5.701 -1.370 0.00 0.00 H

ATOM 1431 HB2 ALA X 95 -9.930 5.042 0.228 0.00 0.00 H

ATOM 1432 HB3 ALA X 95 -9.135 4.205 -1.072 0.00 0.00 H

ATOM 1433 C ALA X 95 -7.234 6.004 0.649 0.00 0.00 C

ATOM 1434 O ALA X 95 -7.279 6.676 1.608 0.00 0.00 O

ATOM 1435 N MET X 96 -6.318 5.046 0.420 0.00 0.00 N

ATOM 1436 HN MET X 96 -6.354 4.441 -0.370 0.00 0.00 H

ATOM 1437 CA MET X 96 -5.219 4.679 1.290 0.00 0.00 C

ATOM 1438 HA MET X 96 -5.626 4.333 2.236 0.00 0.00 H

ATOM 1439 CB MET X 96 -4.370 3.490 0.766 0.00 0.00 C

ATOM 1440 HB1 MET X 96 -3.365 3.427 1.233 0.00 0.00 H

ATOM 1441 HB2 MET X 96 -4.126 3.749 -0.283 0.00 0.00 H

ATOM 1442 CG MET X 96 -5.073 2.173 0.938 0.00 0.00 C

ATOM 1443 HG1 MET X 96 -6.129 2.267 0.585 0.00 0.00 H

ATOM 1444 HG2 MET X 96 -5.153 2.043 2.037 0.00 0.00 H

ATOM 1445 SD MET X 96 -4.392 0.831 -0.058 0.00 0.00 S

ATOM 1446 CE MET X 96 -5.032 -0.358 1.161 0.00 0.00 C

ATOM 1447 HE1 MET X 96 -4.280 -0.349 1.968 0.00 0.00 H

ATOM 1448 HE2 MET X 96 -4.932 -1.353 0.663 0.00 0.00 H

ATOM 1449 HE3 MET X 96 -6.050 -0.139 1.529 0.00 0.00 H

ATOM 1450 C MET X 96 -4.282 5.799 1.630 0.00 0.00 C

ATOM 1451 O MET X 96 -4.008 5.999 2.796 0.00 0.00 O

ATOM 1452 N TYR X 97 -3.781 6.596 0.695 0.00 0.00 N

ATOM 1453 HN TYR X 97 -4.032 6.395 -0.247 0.00 0.00 H

ATOM 1454 CA TYR X 97 -2.931 7.709 0.965 0.00 0.00 C

ATOM 1455 HA TYR X 97 -2.236 7.447 1.744 0.00 0.00 H

ATOM 1456 CB TYR X 97 -2.148 8.288 -0.197 0.00 0.00 C

ATOM 1457 HB1 TYR X 97 -1.442 9.046 0.213 0.00 0.00 H

ATOM 1458 HB2 TYR X 97 -2.830 8.759 -0.938 0.00 0.00 H

ATOM 1459 CG TYR X 97 -1.343 7.160 -0.861 0.00 0.00 C

ATOM 1460 CD1 TYR X 97 -0.301 6.567 -0.113 0.00 0.00 C

ATOM 1461 HD1 TYR X 97 -0.172 6.833 0.921 0.00 0.00 H

ATOM 1462 CE1 TYR X 97 0.553 5.612 -0.723 0.00 0.00 C

ATOM 1463 HE1 TYR X 97 1.395 5.261 -0.154 0.00 0.00 H

ATOM 1464 CZ TYR X 97 0.342 5.335 -2.048 0.00 0.00 C

ATOM 1465 OH TYR X 97 1.298 4.451 -2.722 0.00 0.00 O

ATOM 1466 HH TYR X 97 2.097 4.417 -2.189 0.00 0.00 H

ATOM 1467 CD2 TYR X 97 -1.470 6.923 -2.210 0.00 0.00 C

ATOM 1468 HD2 TYR X 97 -2.216 7.404 -2.828 0.00 0.00 H

ATOM 1469 CE2 TYR X 97 -0.674 6.052 -2.811 0.00 0.00 C

ATOM 1470 HE2 TYR X 97 -0.778 5.859 -3.869 0.00 0.00 H

ATOM 1471 C TYR X 97 -3.722 8.895 1.536 0.00 0.00 C

ATOM 1472 O TYR X 97 -3.195 9.531 2.449 0.00 0.00 O

ATOM 1473 N VAL X 98 -4.962 9.267 1.114 0.00 0.00 N

ATOM 1474 HN VAL X 98 -5.450 8.673 0.486 0.00 0.00 H

ATOM 1475 CA VAL X 98 -5.669 10.457 1.664 0.00 0.00 C

ATOM 1476 HA VAL X 98 -4.848 11.149 1.738 0.00 0.00 H

ATOM 1477 CB VAL X 98 -6.835 10.837 0.765 0.00 0.00 C

ATOM 1478 HB VAL X 98 -7.466 9.975 0.461 0.00 0.00 H

ATOM 1479 CG1 VAL X 98 -7.710 11.830 1.481 0.00 0.00 C

ATOM 1480 HG11 VAL X 98 -8.426 12.361 0.823 0.00 0.00 H

ATOM 1481 HG12 VAL X 98 -7.075 12.618 1.931 0.00 0.00 H

ATOM 1482 HG13 VAL X 98 -8.359 11.358 2.248 0.00 0.00 H

ATOM 1483 CG2 VAL X 98 -6.323 11.487 -0.502 0.00 0.00 C

ATOM 1484 HG21 VAL X 98 -5.840 12.474 -0.334 0.00 0.00 H

ATOM 1485 HG22 VAL X 98 -7.146 11.451 -1.245 0.00 0.00 H

ATOM 1486 HG23 VAL X 98 -5.590 10.847 -1.045 0.00 0.00 H

ATOM 1487 C VAL X 98 -6.254 10.184 3.110 0.00 0.00 C

ATOM 1488 O VAL X 98 -6.279 11.091 3.924 0.00 0.00 O

ATOM 1489 N VAL X 99 -6.629 8.954 3.463 0.00 0.00 N

ATOM 1490 HN VAL X 99 -6.560 8.197 2.826 0.00 0.00 H

ATOM 1491 CA VAL X 99 -7.063 8.692 4.875 0.00 0.00 C

ATOM 1492 HA VAL X 99 -7.643 9.562 5.127 0.00 0.00 H

ATOM 1493 CB VAL X 99 -8.076 7.534 4.983 0.00 0.00 C

ATOM 1494 HB VAL X 99 -8.569 7.525 5.978 0.00 0.00 H

ATOM 1495 CG1 VAL X 99 -9.263 7.771 4.031 0.00 0.00 C

ATOM 1496 HG11 VAL X 99 -9.843 8.647 4.403 0.00 0.00 H

ATOM 1497 HG12 VAL X 99 -9.980 6.918 4.029 0.00 0.00 H

ATOM 1498 HG13 VAL X 99 -8.875 7.973 3.011 0.00 0.00 H

ATOM 1499 CG2 VAL X 99 -7.448 6.184 4.707 0.00 0.00 C

ATOM 1500 HG21 VAL X 99 -6.647 6.128 3.941 0.00 0.00 H

ATOM 1501 HG22 VAL X 99 -8.126 5.359 4.375 0.00 0.00 H

ATOM 1502 HG23 VAL X 99 -7.013 5.705 5.613 0.00 0.00 H

ATOM 1503 C VAL X 99 -5.994 8.738 5.997 0.00 0.00 C

ATOM 1504 O VAL X 99 -6.254 9.194 7.128 0.00 0.00 O

ATOM 1505 N GLY X 100 -4.718 8.373 5.606 0.00 0.00 N

ATOM 1506 HN GLY X 100 -4.672 8.055 4.664 0.00 0.00 H

ATOM 1507 CA GLY X 100 -3.538 8.371 6.450 0.00 0.00 C

ATOM 1508 HA1 GLY X 100 -2.796 7.706 6.031 0.00 0.00 H

ATOM 1509 HA2 GLY X 100 -3.792 7.974 7.427 0.00 0.00 H

ATOM 1510 C GLY X 100 -3.037 9.763 6.574 0.00 0.00 C

ATOM 1511 O GLY X 100 -2.489 10.229 7.641 0.00 0.00 O

ATOM 1512 N PHE X 101 -3.135 10.549 5.473 0.00 0.00 N

ATOM 1513 HN PHE X 101 -3.580 10.119 4.697 0.00 0.00 H

ATOM 1514 CA PHE X 101 -2.900 11.958 5.425 0.00 0.00 C

ATOM 1515 HA PHE X 101 -1.914 12.105 5.845 0.00 0.00 H

ATOM 1516 CB PHE X 101 -2.599 12.398 4.017 0.00 0.00 C

ATOM 1517 HB1 PHE X 101 -3.515 12.144 3.450 0.00 0.00 H

ATOM 1518 HB2 PHE X 101 -1.801 11.861 3.465 0.00 0.00 H

ATOM 1519 CG PHE X 101 -2.486 13.942 3.877 0.00 0.00 C

ATOM 1520 CD1 PHE X 101 -1.485 14.591 4.608 0.00 0.00 C

ATOM 1521 HD1 PHE X 101 -0.813 13.956 5.167 0.00 0.00 H

ATOM 1522 CE1 PHE X 101 -1.227 15.878 4.359 0.00 0.00 C

ATOM 1523 HE1 PHE X 101 -0.341 16.334 4.779 0.00 0.00 H

ATOM 1524 CZ PHE X 101 -2.036 16.704 3.561 0.00 0.00 C

ATOM 1525 HZ PHE X 101 -1.762 17.740 3.431 0.00 0.00 H

ATOM 1526 CD2 PHE X 101 -3.427 14.689 3.140 0.00 0.00 C

ATOM 1527 HD2 PHE X 101 -4.184 14.241 2.512 0.00 0.00 H

ATOM 1528 CE2 PHE X 101 -3.169 16.115 2.932 0.00 0.00 C

ATOM 1529 HE2 PHE X 101 -3.910 16.754 2.466 0.00 0.00 H

ATOM 1530 C PHE X 101 -3.856 12.767 6.346 0.00 0.00 C

ATOM 1531 O PHE X 101 -3.460 13.467 7.300 0.00 0.00 O

ATOM 1532 N ALA X 102 -5.110 12.572 6.262 0.00 0.00 N

ATOM 1533 HN ALA X 102 -5.329 12.045 5.436 0.00 0.00 H

ATOM 1534 CA ALA X 102 -6.217 12.988 7.081 0.00 0.00 C

ATOM 1535 HA ALA X 102 -6.163 14.063 7.157 0.00 0.00 H

ATOM 1536 CB ALA X 102 -7.590 12.582 6.575 0.00 0.00 C

ATOM 1537 HB1 ALA X 102 -7.905 13.235 5.732 0.00 0.00 H

ATOM 1538 HB2 ALA X 102 -8.404 12.712 7.325 0.00 0.00 H

ATOM 1539 HB3 ALA X 102 -7.562 11.511 6.307 0.00 0.00 H

ATOM 1540 C ALA X 102 -6.081 12.527 8.508 0.00 0.00 C

ATOM 1541 O ALA X 102 -6.315 13.301 9.449 0.00 0.00 O

ATOM 1542 N GLU X 103 -5.583 11.279 8.807 0.00 0.00 N

ATOM 1543 HN GLU X 103 -5.274 10.659 8.085 0.00 0.00 H

ATOM 1544 CA GLU X 103 -5.275 10.817 10.181 0.00 0.00 C

ATOM 1545 HA GLU X 103 -6.111 11.027 10.832 0.00 0.00 H

ATOM 1546 CB GLU X 103 -5.065 9.341 10.222 0.00 0.00 C

ATOM 1547 HB1 GLU X 103 -4.415 8.995 9.398 0.00 0.00 H

ATOM 1548 HB2 GLU X 103 -6.034 8.826 10.039 0.00 0.00 H

ATOM 1549 CG GLU X 103 -4.597 8.851 11.676 0.00 0.00 C

ATOM 1550 HG1 GLU X 103 -5.339 9.142 12.453 0.00 0.00 H

ATOM 1551 HG2 GLU X 103 -3.669 9.359 12.006 0.00 0.00 H

ATOM 1552 CD GLU X 103 -4.319 7.347 11.887 0.00 0.00 C

ATOM 1553 OE1 GLU X 103 -5.159 6.670 12.526 0.00 0.00 O

ATOM 1554 OE2 GLU X 103 -3.286 6.877 11.295 0.00 0.00 O1-

ATOM 1555 C GLU X 103 -4.124 11.653 10.756 0.00 0.00 C

ATOM 1556 O GLU X 103 -4.195 12.156 11.843 0.00 0.00 O

ATOM 1557 N THR X 104 -3.034 11.864 10.004 0.00 0.00 N

ATOM 1558 HN THR X 104 -3.026 11.475 9.083 0.00 0.00 H

ATOM 1559 CA THR X 104 -1.963 12.791 10.311 0.00 0.00 C

ATOM 1560 HA THR X 104 -1.577 12.416 11.245 0.00 0.00 H

ATOM 1561 CB THR X 104 -0.771 12.568 9.337 0.00 0.00 C

ATOM 1562 HB THR X 104 -1.144 12.718 8.298 0.00 0.00 H

ATOM 1563 OG1 THR X 104 -0.258 11.208 9.463 0.00 0.00 O

ATOM 1564 HG1 THR X 104 -1.083 10.736 9.586 0.00 0.00 H

ATOM 1565 CG2 THR X 104 0.351 13.512 9.790 0.00 0.00 C

ATOM 1566 HG21 THR X 104 0.142 14.597 9.715 0.00 0.00 H

ATOM 1567 HG22 THR X 104 1.253 13.347 9.153 0.00 0.00 H

ATOM 1568 HG23 THR X 104 0.659 13.332 10.843 0.00 0.00 H

ATOM 1569 C THR X 104 -2.354 14.267 10.443 0.00 0.00 C

ATOM 1570 O THR X 104 -1.803 15.002 11.260 0.00 0.00 O

ATOM 1571 N VAL X 105 -3.295 14.843 9.616 0.00 0.00 N

ATOM 1572 HN VAL X 105 -3.660 14.425 8.797 0.00 0.00 H

ATOM 1573 CA VAL X 105 -3.655 16.246 9.685 0.00 0.00 C

ATOM 1574 HA VAL X 105 -2.684 16.709 9.831 0.00 0.00 H

ATOM 1575 CB VAL X 105 -4.176 16.730 8.356 0.00 0.00 C

ATOM 1576 HB VAL X 105 -5.021 16.032 8.169 0.00 0.00 H

ATOM 1577 CG1 VAL X 105 -4.901 18.135 8.446 0.00 0.00 C

ATOM 1578 HG11 VAL X 105 -5.318 18.572 7.504 0.00 0.00 H

ATOM 1579 HG12 VAL X 105 -4.130 18.855 8.795 0.00 0.00 H

ATOM 1580 HG13 VAL X 105 -5.816 18.193 9.078 0.00 0.00 H

ATOM 1581 CG2 VAL X 105 -3.072 16.789 7.313 0.00 0.00 C

ATOM 1582 HG21 VAL X 105 -2.159 17.275 7.734 0.00 0.00 H

ATOM 1583 HG22 VAL X 105 -3.193 17.378 6.384 0.00 0.00 H

ATOM 1584 HG23 VAL X 105 -2.868 15.751 6.961 0.00 0.00 H

ATOM 1585 C VAL X 105 -4.469 16.512 10.889 0.00 0.00 C

ATOM 1586 O VAL X 105 -4.372 17.642 11.410 0.00 0.00 O

ATOM 1587 N VAL X 106 -5.183 15.454 11.315 0.00 0.00 N

ATOM 1588 HN VAL X 106 -5.315 14.566 10.876 0.00 0.00 H

ATOM 1589 CA VAL X 106 -6.004 15.324 12.494 0.00 0.00 C

ATOM 1590 HA VAL X 106 -6.642 16.194 12.498 0.00 0.00 H

ATOM 1591 CB VAL X 106 -7.047 14.228 12.403 0.00 0.00 C

ATOM 1592 HB VAL X 106 -6.740 13.265 11.926 0.00 0.00 H

ATOM 1593 CG1 VAL X 106 -7.783 13.895 13.625 0.00 0.00 C

ATOM 1594 HG11 VAL X 106 -8.540 13.095 13.505 0.00 0.00 H

ATOM 1595 HG12 VAL X 106 -8.232 14.845 13.997 0.00 0.00 H

ATOM 1596 HG13 VAL X 106 -7.095 13.519 14.410 0.00 0.00 H

ATOM 1597 CG2 VAL X 106 -8.204 14.787 11.534 0.00 0.00 C

ATOM 1598 HG21 VAL X 106 -7.901 15.254 10.568 0.00 0.00 H

ATOM 1599 HG22 VAL X 106 -8.762 15.539 12.129 0.00 0.00 H

ATOM 1600 HG23 VAL X 106 -8.847 13.897 11.351 0.00 0.00 H

ATOM 1601 C VAL X 106 -5.144 15.386 13.683 0.00 0.00 C

ATOM 1602 O VAL X 106 -5.481 16.018 14.713 0.00 0.00 O

ATOM 1603 N GLU X 107 -3.968 14.760 13.641 0.00 0.00 N

ATOM 1604 HN GLU X 107 -3.618 14.414 12.775 0.00 0.00 H

ATOM 1605 CA GLU X 107 -3.025 14.744 14.743 0.00 0.00 C

ATOM 1606 HA GLU X 107 -3.501 14.484 15.676 0.00 0.00 H

ATOM 1607 CB GLU X 107 -1.833 13.743 14.446 0.00 0.00 C

ATOM 1608 HB1 GLU X 107 -1.177 13.940 15.321 0.00 0.00 H

ATOM 1609 HB2 GLU X 107 -1.431 14.082 13.469 0.00 0.00 H

ATOM 1610 CG GLU X 107 -2.136 12.250 14.552 0.00 0.00 C

ATOM 1611 HG1 GLU X 107 -1.314 11.607 14.170 0.00 0.00 H

ATOM 1612 HG2 GLU X 107 -2.989 11.965 13.903 0.00 0.00 H

ATOM 1613 CD GLU X 107 -2.406 11.813 16.013 0.00 0.00 C

ATOM 1614 OE1 GLU X 107 -1.475 11.262 16.726 0.00 0.00 O

ATOM 1615 OE2 GLU X 107 -3.597 12.033 16.433 0.00 0.00 O1-

ATOM 1616 C GLU X 107 -2.497 16.189 15.027 0.00 0.00 C

ATOM 1617 O GLU X 107 -2.270 16.533 16.148 0.00 0.00 O

ATOM 1618 N LEU X 108 -2.207 17.074 13.992 0.00 0.00 N

ATOM 1619 HN LEU X 108 -2.217 16.731 13.056 0.00 0.00 H

ATOM 1620 CA LEU X 108 -1.953 18.514 14.076 0.00 0.00 C

ATOM 1621 HA LEU X 108 -1.172 18.630 14.818 0.00 0.00 H

ATOM 1622 CB LEU X 108 -1.431 19.049 12.726 0.00 0.00 C

ATOM 1623 HB1 LEU X 108 -1.671 20.142 12.688 0.00 0.00 H

ATOM 1624 HB2 LEU X 108 -1.966 18.486 11.939 0.00 0.00 H

ATOM 1625 CG LEU X 108 0.152 19.071 12.514 0.00 0.00 C

ATOM 1626 HG LEU X 108 0.582 18.051 12.598 0.00 0.00 H

ATOM 1627 CD1 LEU X 108 0.357 19.566 11.082 0.00 0.00 C

ATOM 1628 HD11 LEU X 108 -0.073 18.839 10.358 0.00 0.00 H

ATOM 1629 HD12 LEU X 108 1.441 19.747 10.926 0.00 0.00 H

ATOM 1630 HD13 LEU X 108 -0.183 20.529 11.030 0.00 0.00 H

ATOM 1631 CD2 LEU X 108 0.872 20.025 13.477 0.00 0.00 C

ATOM 1632 HD21 LEU X 108 0.759 19.698 14.535 0.00 0.00 H

ATOM 1633 HD22 LEU X 108 0.472 21.053 13.395 0.00 0.00 H

ATOM 1634 HD23 LEU X 108 1.956 20.000 13.234 0.00 0.00 H

ATOM 1635 C LEU X 108 -3.093 19.235 14.639 0.00 0.00 C

ATOM 1636 O LEU X 108 -3.001 20.050 15.533 0.00 0.00 O

ATOM 1637 N LEU X 109 -4.342 18.959 14.209 0.00 0.00 N

ATOM 1638 HN LEU X 109 -4.505 18.343 13.435 0.00 0.00 H

ATOM 1639 CA LEU X 109 -5.546 19.475 14.910 0.00 0.00 C

ATOM 1640 HA LEU X 109 -5.555 20.550 14.848 0.00 0.00 H

ATOM 1641 CB LEU X 109 -6.880 19.031 14.199 0.00 0.00 C

ATOM 1642 HB1 LEU X 109 -7.756 19.473 14.709 0.00 0.00 H

ATOM 1643 HB2 LEU X 109 -7.101 17.948 14.348 0.00 0.00 H

ATOM 1644 CG LEU X 109 -7.133 19.368 12.707 0.00 0.00 C

ATOM 1645 HG LEU X 109 -6.449 18.695 12.151 0.00 0.00 H

ATOM 1646 CD1 LEU X 109 -8.600 19.098 12.233 0.00 0.00 C

ATOM 1647 HD11 LEU X 109 -9.285 19.832 12.704 0.00 0.00 H

ATOM 1648 HD12 LEU X 109 -8.882 18.071 12.552 0.00 0.00 H

ATOM 1649 HD13 LEU X 109 -8.764 19.211 11.139 0.00 0.00 H

ATOM 1650 CD2 LEU X 109 -6.745 20.873 12.267 0.00 0.00 C

ATOM 1651 HD21 LEU X 109 -6.966 21.123 11.208 0.00 0.00 H

ATOM 1652 HD22 LEU X 109 -5.642 20.968 12.361 0.00 0.00 H

ATOM 1653 HD23 LEU X 109 -7.174 21.680 12.902 0.00 0.00 H

ATOM 1654 C LEU X 109 -5.623 19.201 16.351 0.00 0.00 C

ATOM 1655 O LEU X 109 -5.789 20.219 17.072 0.00 0.00 O

ATOM 1656 N LYS X 110 -5.425 17.919 16.801 0.00 0.00 N

ATOM 1657 HN LYS X 110 -5.357 17.224 16.088 0.00 0.00 H

ATOM 1658 CA LYS X 110 -5.436 17.438 18.092 0.00 0.00 C

ATOM 1659 HA LYS X 110 -6.243 17.764 18.738 0.00 0.00 H

ATOM 1660 CB LYS X 110 -5.670 15.823 18.214 0.00 0.00 C

ATOM 1661 HB1 LYS X 110 -5.499 15.541 19.279 0.00 0.00 H

ATOM 1662 HB2 LYS X 110 -4.961 15.361 17.495 0.00 0.00 H

ATOM 1663 CG LYS X 110 -7.031 15.272 17.739 0.00 0.00 C

ATOM 1664 HG1 LYS X 110 -7.161 14.190 17.978 0.00 0.00 H

ATOM 1665 HG2 LYS X 110 -7.133 15.366 16.643 0.00 0.00 H

ATOM 1666 CD LYS X 110 -8.190 16.070 18.336 0.00 0.00 C

ATOM 1667 HD1 LYS X 110 -8.158 17.099 17.918 0.00 0.00 H

ATOM 1668 HD2 LYS X 110 -8.163 16.193 19.439 0.00 0.00 H

ATOM 1669 CE LYS X 110 -9.571 15.597 17.904 0.00 0.00 C

ATOM 1670 HE1 LYS X 110 -9.694 15.471 16.810 0.00 0.00 H

ATOM 1671 HE2 LYS X 110 -10.293 16.363 18.258 0.00 0.00 H

ATOM 1672 NZ LYS X 110 -9.987 14.418 18.733 0.00 0.00 N1+

ATOM 1673 HZ1 LYS X 110 -9.465 14.383 19.629 0.00 0.00 H

ATOM 1674 HZ2 LYS X 110 -9.790 13.507 18.281 0.00 0.00 H

ATOM 1675 HZ3 LYS X 110 -11.021 14.398 18.853 0.00 0.00 H

ATOM 1676 C LYS X 110 -4.295 17.895 19.033 0.00 0.00 C

ATOM 1677 O LYS X 110 -4.457 18.373 20.112 0.00 0.00 O

ATOM 1678 N GLU X 111 -3.105 18.004 18.369 0.00 0.00 N

ATOM 1679 HN GLU X 111 -3.053 17.677 17.429 0.00 0.00 H

ATOM 1680 CA GLU X 111 -1.895 18.606 18.985 0.00 0.00 C

ATOM 1681 HA GLU X 111 -1.611 18.100 19.897 0.00 0.00 H

ATOM 1682 CB GLU X 111 -0.628 18.367 18.141 0.00 0.00 C

ATOM 1683 HB1 GLU X 111 -0.915 18.535 17.081 0.00 0.00 H

ATOM 1684 HB2 GLU X 111 -0.470 17.281 18.281 0.00 0.00 H

ATOM 1685 CG GLU X 111 0.488 19.447 18.383 0.00 0.00 C

ATOM 1686 HG1 GLU X 111 0.485 19.346 19.498 0.00 0.00 H

ATOM 1687 HG2 GLU X 111 0.184 20.477 18.110 0.00 0.00 H

ATOM 1688 CD GLU X 111 1.849 19.218 17.861 0.00 0.00 C

ATOM 1689 OE1 GLU X 111 2.286 18.039 17.644 0.00 0.00 O

ATOM 1690 OE2 GLU X 111 2.520 20.266 17.653 0.00 0.00 O1-

ATOM 1691 C GLU X 111 -2.123 20.064 19.418 0.00 0.00 C

ATOM 1692 O GLU X 111 -1.735 20.496 20.490 0.00 0.00 O

ATOM 1693 N HSD X 112 -2.909 20.767 18.590 0.00 0.00 N

ATOM 1694 HN HSD X 112 -3.035 20.398 17.673 0.00 0.00 H

ATOM 1695 CA HSD X 112 -3.122 22.175 18.766 0.00 0.00 C

ATOM 1696 HA HSD X 112 -2.446 22.627 19.475 0.00 0.00 H

ATOM 1697 CB HSD X 112 -2.996 22.925 17.379 0.00 0.00 C

ATOM 1698 HB1 HSD X 112 -3.391 23.936 17.581 0.00 0.00 H

ATOM 1699 HB2 HSD X 112 -3.559 22.422 16.554 0.00 0.00 H

ATOM 1700 ND1 HSD X 112 -0.518 23.294 17.635 0.00 0.00 N

ATOM 1701 HD1 HSD X 112 -0.568 23.692 18.551 0.00 0.00 H

ATOM 1702 CG HSD X 112 -1.627 22.897 16.875 0.00 0.00 C

ATOM 1703 CE1 HSD X 112 0.523 23.081 16.845 0.00 0.00 C

ATOM 1704 HE1 HSD X 112 1.562 23.310 17.074 0.00 0.00 H

ATOM 1705 NE2 HSD X 112 0.233 22.438 15.707 0.00 0.00 N

ATOM 1706 CD2 HSD X 112 -1.096 22.328 15.720 0.00 0.00 C

ATOM 1707 HD2 HSD X 112 -1.676 21.846 14.953 0.00 0.00 H

ATOM 1708 C HSD X 112 -4.621 22.344 19.428 0.00 0.00 C

ATOM 1709 O HSD X 112 -5.046 23.480 19.548 0.00 0.00 O

ATOM 1710 N SER X 113 -5.275 21.231 19.874 0.00 0.00 N

ATOM 1711 HN SER X 113 -4.750 20.393 19.775 0.00 0.00 H

ATOM 1712 CA SER X 113 -6.604 21.309 20.550 0.00 0.00 C

ATOM 1713 HA SER X 113 -6.971 20.304 20.647 0.00 0.00 H

ATOM 1714 CB SER X 113 -6.315 21.676 21.969 0.00 0.00 C

ATOM 1715 HB1 SER X 113 -6.017 22.756 21.960 0.00 0.00 H

ATOM 1716 HB2 SER X 113 -5.499 21.071 22.410 0.00 0.00 H

ATOM 1717 OG SER X 113 -7.431 21.557 22.903 0.00 0.00 O

ATOM 1718 HG1 SER X 113 -8.065 22.245 22.708 0.00 0.00 H

ATOM 1719 C SER X 113 -7.684 22.103 19.775 0.00 0.00 C

ATOM 1720 O SER X 113 -8.292 23.077 20.299 0.00 0.00 O

ATOM 1721 N ILE X 114 -7.961 21.776 18.496 0.00 0.00 N

ATOM 1722 HN ILE X 114 -7.462 21.023 18.086 0.00 0.00 H

ATOM 1723 CA ILE X 114 -9.040 22.256 17.641 0.00 0.00 C

ATOM 1724 HA ILE X 114 -9.454 23.043 18.239 0.00 0.00 H

ATOM 1725 CB ILE X 114 -8.486 22.738 16.359 0.00 0.00 C

ATOM 1726 HB ILE X 114 -7.883 21.906 15.925 0.00 0.00 H

ATOM 1727 CG2 ILE X 114 -9.538 23.113 15.231 0.00 0.00 C

ATOM 1728 HG21 ILE X 114 -9.031 23.366 14.271 0.00 0.00 H

ATOM 1729 HG22 ILE X 114 -10.215 23.920 15.577 0.00 0.00 H

ATOM 1730 HG23 ILE X 114 -10.066 22.162 15.015 0.00 0.00 H

ATOM 1731 CG1 ILE X 114 -7.443 23.926 16.595 0.00 0.00 C

ATOM 1732 HG11 ILE X 114 -6.812 23.991 15.682 0.00 0.00 H

ATOM 1733 HG12 ILE X 114 -6.735 23.678 17.415 0.00 0.00 H

ATOM 1734 CD ILE X 114 -8.180 25.210 17.004 0.00 0.00 C

ATOM 1735 HD1 ILE X 114 -8.568 25.095 18.038 0.00 0.00 H

ATOM 1736 HD2 ILE X 114 -9.065 25.364 16.353 0.00 0.00 H

ATOM 1737 HD3 ILE X 114 -7.476 26.070 16.933 0.00 0.00 H

ATOM 1738 C ILE X 114 -10.027 21.139 17.476 0.00 0.00 C

ATOM 1739 O ILE X 114 -9.638 20.038 17.178 0.00 0.00 O

ATOM 1740 N LEU X 115 -11.269 21.423 17.816 0.00 0.00 N

ATOM 1741 HN LEU X 115 -11.608 22.280 18.216 0.00 0.00 H

ATOM 1742 CA LEU X 115 -12.282 20.450 17.860 0.00 0.00 C

ATOM 1743 HA LEU X 115 -11.906 19.475 17.573 0.00 0.00 H

ATOM 1744 CB LEU X 115 -12.875 20.140 19.258 0.00 0.00 C

ATOM 1745 HB1 LEU X 115 -13.285 21.058 19.737 0.00 0.00 H

ATOM 1746 HB2 LEU X 115 -12.032 19.814 19.916 0.00 0.00 H

ATOM 1747 CG LEU X 115 -13.773 18.835 19.489 0.00 0.00 C

ATOM 1748 HG LEU X 115 -14.596 18.728 18.751 0.00 0.00 H

ATOM 1749 CD1 LEU X 115 -12.895 17.560 19.246 0.00 0.00 C

ATOM 1750 HD11 LEU X 115 -11.941 17.633 19.809 0.00 0.00 H

ATOM 1751 HD12 LEU X 115 -12.894 17.395 18.148 0.00 0.00 H

ATOM 1752 HD13 LEU X 115 -13.356 16.677 19.728 0.00 0.00 H

ATOM 1753 CD2 LEU X 115 -14.290 18.893 20.910 0.00 0.00 C

ATOM 1754 HD21 LEU X 115 -13.568 18.961 21.761 0.00 0.00 H

ATOM 1755 HD22 LEU X 115 -14.906 17.978 20.934 0.00 0.00 H

ATOM 1756 HD23 LEU X 115 -14.910 19.816 21.033 0.00 0.00 H

ATOM 1757 C LEU X 115 -13.394 20.819 16.797 0.00 0.00 C

ATOM 1758 O LEU X 115 -14.116 21.774 17.038 0.00 0.00 O

ATOM 1759 N MET X 116 -13.473 20.093 15.698 0.00 0.00 N

ATOM 1760 HN MET X 116 -12.817 19.375 15.484 0.00 0.00 H

ATOM 1761 CA MET X 116 -14.521 20.179 14.731 0.00 0.00 C

ATOM 1762 HA MET X 116 -14.930 21.176 14.731 0.00 0.00 H

ATOM 1763 CB MET X 116 -13.991 19.851 13.318 0.00 0.00 C

ATOM 1764 HB1 MET X 116 -14.761 20.115 12.551 0.00 0.00 H

ATOM 1765 HB2 MET X 116 -13.751 18.785 13.466 0.00 0.00 H

ATOM 1766 CG MET X 116 -12.708 20.505 12.992 0.00 0.00 C

ATOM 1767 HG1 MET X 116 -11.895 20.186 13.676 0.00 0.00 H

ATOM 1768 HG2 MET X 116 -12.806 21.600 13.160 0.00 0.00 H

ATOM 1769 SD MET X 116 -12.128 20.328 11.292 0.00 0.00 S

ATOM 1770 CE MET X 116 -11.958 18.525 11.259 0.00 0.00 C

ATOM 1771 HE1 MET X 116 -11.055 18.226 11.821 0.00 0.00 H

ATOM 1772 HE2 MET X 116 -11.883 18.174 10.202 0.00 0.00 H

ATOM 1773 HE3 MET X 116 -12.811 17.940 11.669 0.00 0.00 H

ATOM 1774 C MET X 116 -15.640 19.185 14.957 0.00 0.00 C

ATOM 1775 O MET X 116 -16.777 19.578 15.149 0.00 0.00 O

ATOM 1776 N ILE X 117 -15.440 17.821 14.898 0.00 0.00 N

ATOM 1777 HN ILE X 117 -14.488 17.503 14.912 0.00 0.00 H

ATOM 1778 CA ILE X 117 -16.549 16.935 15.148 0.00 0.00 C

ATOM 1779 HA ILE X 117 -17.311 17.339 15.794 0.00 0.00 H

ATOM 1780 CB ILE X 117 -17.310 16.569 13.910 0.00 0.00 C

ATOM 1781 HB ILE X 117 -17.617 17.502 13.391 0.00 0.00 H

ATOM 1782 CG2 ILE X 117 -16.389 15.893 12.896 0.00 0.00 C

ATOM 1783 HG21 ILE X 117 -16.192 14.868 13.288 0.00 0.00 H

ATOM 1784 HG22 ILE X 117 -15.494 16.490 12.631 0.00 0.00 H

ATOM 1785 HG23 ILE X 117 -17.017 15.562 12.053 0.00 0.00 H

ATOM 1786 CG1 ILE X 117 -18.510 15.637 14.184 0.00 0.00 C

ATOM 1787 HG11 ILE X 117 -18.104 14.636 14.436 0.00 0.00 H

ATOM 1788 HG12 ILE X 117 -18.754 15.528 13.096 0.00 0.00 H

ATOM 1789 CD ILE X 117 -19.747 16.249 14.946 0.00 0.00 C

ATOM 1790 HD1 ILE X 117 -20.166 17.148 14.457 0.00 0.00 H

ATOM 1791 HD2 ILE X 117 -19.441 16.594 15.954 0.00 0.00 H

ATOM 1792 HD3 ILE X 117 -20.498 15.437 15.059 0.00 0.00 H

ATOM 1793 C ILE X 117 -16.137 15.671 15.832 0.00 0.00 C

ATOM 1794 O ILE X 117 -16.956 15.100 16.526 0.00 0.00 O

ATOM 1795 N ASP X 118 -14.775 15.191 15.679 0.00 0.00 N

ATOM 1796 HN ASP X 118 -14.268 15.620 14.940 0.00 0.00 H

ATOM 1797 CA ASP X 118 -14.003 14.377 16.564 0.00 0.00 C

ATOM 1798 HA ASP X 118 -12.981 14.713 16.683 0.00 0.00 H

ATOM 1799 CB ASP X 118 -14.613 14.261 18.047 0.00 0.00 C

ATOM 1800 HB1 ASP X 118 -15.473 13.562 18.101 0.00 0.00 H

ATOM 1801 HB2 ASP X 118 -15.005 15.266 18.280 0.00 0.00 H

ATOM 1802 CG ASP X 118 -13.695 13.942 19.177 0.00 0.00 C

ATOM 1803 OD1 ASP X 118 -14.098 13.928 20.313 0.00 0.00 O

ATOM 1804 OD2 ASP X 118 -12.453 13.683 18.926 0.00 0.00 O1-

ATOM 1805 C ASP X 118 -13.880 13.019 15.952 0.00 0.00 C

ATOM 1806 O ASP X 118 -14.641 12.506 15.136 0.00 0.00 O

ATOM 1807 N GLU X 119 -12.845 12.275 16.517 0.00 0.00 N

ATOM 1808 HN GLU X 119 -12.487 12.575 17.393 0.00 0.00 H

ATOM 1809 CA GLU X 119 -12.463 10.967 16.242 0.00 0.00 C

ATOM 1810 HA GLU X 119 -11.417 10.864 16.484 0.00 0.00 H

ATOM 1811 CB GLU X 119 -13.350 9.885 16.984 0.00 0.00 C

ATOM 1812 HB1 GLU X 119 -12.948 8.890 16.690 0.00 0.00 H

ATOM 1813 HB2 GLU X 119 -14.402 9.920 16.624 0.00 0.00 H

ATOM 1814 CG GLU X 119 -13.248 9.963 18.532 0.00 0.00 C

ATOM 1815 HG1 GLU X 119 -13.948 10.798 18.753 0.00 0.00 H

ATOM 1816 HG2 GLU X 119 -12.176 10.219 18.713 0.00 0.00 H

ATOM 1817 CD GLU X 119 -13.586 8.733 19.232 0.00 0.00 C

ATOM 1818 OE1 GLU X 119 -14.786 8.319 19.310 0.00 0.00 O

ATOM 1819 OE2 GLU X 119 -12.753 8.012 19.885 0.00 0.00 O1-

ATOM 1820 C GLU X 119 -12.300 10.621 14.720 0.00 0.00 C

ATOM 1821 O GLU X 119 -11.462 11.212 14.077 0.00 0.00 O

ATOM 1822 N ILE X 120 -13.084 9.670 14.164 0.00 0.00 N

ATOM 1823 HN ILE X 120 -13.658 9.088 14.735 0.00 0.00 H

ATOM 1824 CA ILE X 120 -13.050 9.285 12.802 0.00 0.00 C

ATOM 1825 HA ILE X 120 -12.101 9.361 12.275 0.00 0.00 H

ATOM 1826 CB ILE X 120 -13.516 7.863 12.815 0.00 0.00 C

ATOM 1827 HB ILE X 120 -12.791 7.337 13.474 0.00 0.00 H

ATOM 1828 CG2 ILE X 120 -14.922 7.655 13.371 0.00 0.00 C

ATOM 1829 HG21 ILE X 120 -15.660 8.029 12.641 0.00 0.00 H

ATOM 1830 HG22 ILE X 120 -15.098 8.068 14.393 0.00 0.00 H

ATOM 1831 HG23 ILE X 120 -15.157 6.575 13.518 0.00 0.00 H

ATOM 1832 CG1 ILE X 120 -13.479 7.116 11.447 0.00 0.00 C

ATOM 1833 HG11 ILE X 120 -14.228 7.574 10.761 0.00 0.00 H

ATOM 1834 HG12 ILE X 120 -13.808 6.080 11.662 0.00 0.00 H

ATOM 1835 CD ILE X 120 -12.089 6.824 10.943 0.00 0.00 C

ATOM 1836 HD1 ILE X 120 -11.699 7.727 10.432 0.00 0.00 H

ATOM 1837 HD2 ILE X 120 -12.082 6.010 10.191 0.00 0.00 H

ATOM 1838 HD3 ILE X 120 -11.524 6.667 11.892 0.00 0.00 H

ATOM 1839 C ILE X 120 -13.897 10.214 11.882 0.00 0.00 C

ATOM 1840 O ILE X 120 -13.570 10.377 10.707 0.00 0.00 O

ATOM 1841 N ASN X 121 -14.910 10.945 12.428 0.00 0.00 N

ATOM 1842 HN ASN X 121 -15.186 10.869 13.381 0.00 0.00 H

ATOM 1843 CA ASN X 121 -15.585 11.889 11.667 0.00 0.00 C

ATOM 1844 HA ASN X 121 -15.889 11.538 10.689 0.00 0.00 H

ATOM 1845 CB ASN X 121 -16.966 12.310 12.184 0.00 0.00 C

ATOM 1846 HB1 ASN X 121 -17.395 12.914 11.361 0.00 0.00 H

ATOM 1847 HB2 ASN X 121 -16.983 12.869 13.143 0.00 0.00 H

ATOM 1848 CG ASN X 121 -17.945 11.123 12.257 0.00 0.00 C

ATOM 1849 OD1 ASN X 121 -17.939 10.380 11.329 0.00 0.00 O

ATOM 1850 ND2 ASN X 121 -18.567 10.946 13.450 0.00 0.00 N

ATOM 1851 HD21 ASN X 121 -18.569 11.629 14.191 0.00 0.00 H

ATOM 1852 HD22 ASN X 121 -19.096 10.121 13.619 0.00 0.00 H

ATOM 1853 C ASN X 121 -14.741 13.095 11.245 0.00 0.00 C

ATOM 1854 O ASN X 121 -14.857 13.552 10.124 0.00 0.00 O

ATOM 1855 N ASP X 122 -13.722 13.554 12.001 0.00 0.00 N

ATOM 1856 HN ASP X 122 -13.556 13.084 12.867 0.00 0.00 H

ATOM 1857 CA ASP X 122 -12.764 14.551 11.616 0.00 0.00 C

ATOM 1858 HA ASP X 122 -13.366 15.385 11.274 0.00 0.00 H

ATOM 1859 CB ASP X 122 -11.758 14.814 12.767 0.00 0.00 C

ATOM 1860 HB1 ASP X 122 -10.912 15.391 12.337 0.00 0.00 H

ATOM 1861 HB2 ASP X 122 -11.353 13.841 13.138 0.00 0.00 H

ATOM 1862 CG ASP X 122 -12.184 15.524 13.994 0.00 0.00 C

ATOM 1863 OD1 ASP X 122 -11.410 15.465 14.990 0.00 0.00 O

ATOM 1864 OD2 ASP X 122 -13.169 16.236 13.920 0.00 0.00 O1-

ATOM 1865 C ASP X 122 -11.917 14.143 10.446 0.00 0.00 C

ATOM 1866 O ASP X 122 -11.611 14.834 9.516 0.00 0.00 O

ATOM 1867 N ILE X 123 -11.518 12.878 10.402 0.00 0.00 N

ATOM 1868 HN ILE X 123 -11.614 12.469 11.316 0.00 0.00 H

ATOM 1869 CA ILE X 123 -10.808 12.142 9.359 0.00 0.00 C

ATOM 1870 HA ILE X 123 -9.962 12.787 9.156 0.00 0.00 H

ATOM 1871 CB ILE X 123 -10.070 10.867 9.967 0.00 0.00 C

ATOM 1872 HB ILE X 123 -10.902 10.194 10.254 0.00 0.00 H

ATOM 1873 CG2 ILE X 123 -9.414 10.171 8.775 0.00 0.00 C

ATOM 1874 HG21 ILE X 123 -9.029 9.193 9.123 0.00 0.00 H

ATOM 1875 HG22 ILE X 123 -8.518 10.659 8.336 0.00 0.00 H

ATOM 1876 HG23 ILE X 123 -10.141 9.948 7.964 0.00 0.00 H

ATOM 1877 CG1 ILE X 123 -9.109 11.222 11.037 0.00 0.00 C

ATOM 1878 HG11 ILE X 123 -9.536 11.848 11.846 0.00 0.00 H

ATOM 1879 HG12 ILE X 123 -8.246 11.758 10.581 0.00 0.00 H

ATOM 1880 CD ILE X 123 -8.443 9.989 11.766 0.00 0.00 C

ATOM 1881 HD1 ILE X 123 -7.764 9.482 11.047 0.00 0.00 H

ATOM 1882 HD2 ILE X 123 -9.264 9.314 12.074 0.00 0.00 H

ATOM 1883 HD3 ILE X 123 -7.812 10.390 12.584 0.00 0.00 H

ATOM 1884 C ILE X 123 -11.502 12.005 8.052 0.00 0.00 C

ATOM 1885 O ILE X 123 -10.899 12.234 6.984 0.00 0.00 O

ATOM 1886 N ARG X 124 -12.865 11.693 8.085 0.00 0.00 N

ATOM 1887 HN ARG X 124 -13.205 11.396 8.975 0.00 0.00 H

ATOM 1888 CA ARG X 124 -13.757 11.865 6.989 0.00 0.00 C

ATOM 1889 HA ARG X 124 -13.393 11.244 6.172 0.00 0.00 H

ATOM 1890 CB ARG X 124 -15.142 11.274 7.300 0.00 0.00 C

ATOM 1891 HB1 ARG X 124 -15.913 11.590 6.570 0.00 0.00 H

ATOM 1892 HB2 ARG X 124 -15.382 11.769 8.269 0.00 0.00 H

ATOM 1893 CG ARG X 124 -15.214 9.676 7.550 0.00 0.00 C

ATOM 1894 HG1 ARG X 124 -14.213 9.217 7.719 0.00 0.00 H

ATOM 1895 HG2 ARG X 124 -15.729 9.419 6.603 0.00 0.00 H

ATOM 1896 CD ARG X 124 -16.253 9.247 8.664 0.00 0.00 C

ATOM 1897 HD1 ARG X 124 -17.264 9.723 8.674 0.00 0.00 H

ATOM 1898 HD2 ARG X 124 -15.777 9.506 9.637 0.00 0.00 H

ATOM 1899 NE ARG X 124 -16.379 7.755 8.639 0.00 0.00 N

ATOM 1900 HE ARG X 124 -16.032 7.303 7.819 0.00 0.00 H

ATOM 1901 CZ ARG X 124 -16.652 6.969 9.689 0.00 0.00 C

ATOM 1902 NH1 ARG X 124 -17.143 7.360 10.769 0.00 0.00 N1+

ATOM 1903 HH11 ARG X 124 -17.348 6.636 11.420 0.00 0.00 H

ATOM 1904 HH12 ARG X 124 -17.358 8.326 10.913 0.00 0.00 H

ATOM 1905 NH2 ARG X 124 -16.317 5.699 9.568 0.00 0.00 N

ATOM 1906 HH21 ARG X 124 -15.923 5.302 8.740 0.00 0.00 H

ATOM 1907 HH22 ARG X 124 -16.465 5.077 10.329 0.00 0.00 H

ATOM 1908 C ARG X 124 -13.866 13.261 6.410 0.00 0.00 C

ATOM 1909 O ARG X 124 -13.542 13.396 5.217 0.00 0.00 O

ATOM 1910 N ILE X 125 -14.081 14.322 7.244 0.00 0.00 N

ATOM 1911 HN ILE X 125 -14.426 14.085 8.149 0.00 0.00 H

ATOM 1912 CA ILE X 125 -14.066 15.782 6.876 0.00 0.00 C

ATOM 1913 HA ILE X 125 -14.905 15.942 6.220 0.00 0.00 H

ATOM 1914 CB ILE X 125 -14.390 16.784 7.974 0.00 0.00 C

ATOM 1915 HB ILE X 125 -13.614 16.758 8.766 0.00 0.00 H

ATOM 1916 CG2 ILE X 125 -14.571 18.189 7.286 0.00 0.00 C

ATOM 1917 HG21 ILE X 125 -15.356 18.245 6.501 0.00 0.00 H

ATOM 1918 HG22 ILE X 125 -13.602 18.496 6.847 0.00 0.00 H

ATOM 1919 HG23 ILE X 125 -14.845 18.893 8.097 0.00 0.00 H

ATOM 1920 CG1 ILE X 125 -15.693 16.354 8.697 0.00 0.00 C

ATOM 1921 HG11 ILE X 125 -15.509 15.434 9.292 0.00 0.00 H

ATOM 1922 HG12 ILE X 125 -16.424 16.024 7.923 0.00 0.00 H

ATOM 1923 CD ILE X 125 -16.245 17.493 9.620 0.00 0.00 C

ATOM 1924 HD1 ILE X 125 -15.472 17.932 10.280 0.00 0.00 H

ATOM 1925 HD2 ILE X 125 -17.144 17.126 10.161 0.00 0.00 H

ATOM 1926 HD3 ILE X 125 -16.591 18.350 9.001 0.00 0.00 H

ATOM 1927 C ILE X 125 -12.749 16.235 6.082 0.00 0.00 C

ATOM 1928 O ILE X 125 -12.792 16.795 5.002 0.00 0.00 O

ATOM 1929 N ILE X 126 -11.539 15.994 6.715 0.00 0.00 N

ATOM 1930 HN ILE X 126 -11.560 15.650 7.652 0.00 0.00 H

ATOM 1931 CA ILE X 126 -10.265 16.405 6.080 0.00 0.00 C

ATOM 1932 HA ILE X 126 -10.161 17.466 5.900 0.00 0.00 H

ATOM 1933 CB ILE X 126 -9.016 16.153 6.979 0.00 0.00 C

ATOM 1934 HB ILE X 126 -8.955 15.102 7.308 0.00 0.00 H

ATOM 1935 CG2 ILE X 126 -7.680 16.523 6.193 0.00 0.00 C

ATOM 1936 HG21 ILE X 126 -6.845 16.382 6.909 0.00 0.00 H

ATOM 1937 HG22 ILE X 126 -7.819 17.567 5.836 0.00 0.00 H

ATOM 1938 HG23 ILE X 126 -7.512 15.915 5.274 0.00 0.00 H

ATOM 1939 CG1 ILE X 126 -9.072 16.844 8.298 0.00 0.00 C

ATOM 1940 HG11 ILE X 126 -8.126 16.629 8.829 0.00 0.00 H

ATOM 1941 HG12 ILE X 126 -9.793 16.310 8.961 0.00 0.00 H

ATOM 1942 CD ILE X 126 -9.434 18.370 8.218 0.00 0.00 C

ATOM 1943 HD1 ILE X 126 -10.435 18.535 7.765 0.00 0.00 H

ATOM 1944 HD2 ILE X 126 -8.825 18.900 7.453 0.00 0.00 H

ATOM 1945 HD3 ILE X 126 -9.330 18.891 9.203 0.00 0.00 H

ATOM 1946 C ILE X 126 -10.073 15.586 4.754 0.00 0.00 C

ATOM 1947 O ILE X 126 -9.840 16.045 3.630 0.00 0.00 O

ATOM 1948 N GLY X 127 -10.404 14.314 4.853 0.00 0.00 N

ATOM 1949 HN GLY X 127 -10.803 13.813 5.609 0.00 0.00 H

ATOM 1950 CA GLY X 127 -10.166 13.464 3.695 0.00 0.00 C

ATOM 1951 HA1 GLY X 127 -10.406 12.481 4.047 0.00 0.00 H

ATOM 1952 HA2 GLY X 127 -9.119 13.569 3.430 0.00 0.00 H

ATOM 1953 C GLY X 127 -11.026 13.705 2.507 0.00 0.00 C

ATOM 1954 O GLY X 127 -10.546 13.818 1.372 0.00 0.00 O

ATOM 1955 N ALA X 128 -12.301 13.909 2.697 0.00 0.00 N

ATOM 1956 HN ALA X 128 -12.685 13.890 3.614 0.00 0.00 H

ATOM 1957 CA ALA X 128 -13.132 14.190 1.569 0.00 0.00 C

ATOM 1958 HA ALA X 128 -13.050 13.406 0.844 0.00 0.00 H

ATOM 1959 CB ALA X 128 -14.622 14.375 2.130 0.00 0.00 C

ATOM 1960 HB1 ALA X 128 -15.348 14.526 1.305 0.00 0.00 H

ATOM 1961 HB2 ALA X 128 -14.729 15.236 2.828 0.00 0.00 H

ATOM 1962 HB3 ALA X 128 -14.744 13.428 2.694 0.00 0.00 H

ATOM 1963 C ALA X 128 -12.749 15.507 0.758 0.00 0.00 C

ATOM 1964 O ALA X 128 -12.715 15.487 -0.498 0.00 0.00 O

ATOM 1965 N ILE X 129 -12.408 16.674 1.423 0.00 0.00 N

ATOM 1966 HN ILE X 129 -12.346 16.612 2.422 0.00 0.00 H

ATOM 1967 CA ILE X 129 -11.892 17.828 0.808 0.00 0.00 C

ATOM 1968 HA ILE X 129 -12.582 18.221 0.071 0.00 0.00 H

ATOM 1969 CB ILE X 129 -11.813 19.119 1.789 0.00 0.00 C

ATOM 1970 HB ILE X 129 -12.877 19.179 2.103 0.00 0.00 H

ATOM 1971 CG2 ILE X 129 -11.028 18.891 3.080 0.00 0.00 C

ATOM 1972 HG21 ILE X 129 -11.575 18.108 3.647 0.00 0.00 H

ATOM 1973 HG22 ILE X 129 -10.879 19.832 3.649 0.00 0.00 H

ATOM 1974 HG23 ILE X 129 -10.090 18.332 2.836 0.00 0.00 H

ATOM 1975 CG1 ILE X 129 -11.431 20.478 1.116 0.00 0.00 C

ATOM 1976 HG11 ILE X 129 -10.526 20.307 0.500 0.00 0.00 H

ATOM 1977 HG12 ILE X 129 -11.164 21.127 1.979 0.00 0.00 H

ATOM 1978 CD ILE X 129 -12.545 21.080 0.259 0.00 0.00 C

ATOM 1979 HD1 ILE X 129 -13.501 21.065 0.821 0.00 0.00 H

ATOM 1980 HD2 ILE X 129 -12.689 20.371 -0.577 0.00 0.00 H

ATOM 1981 HD3 ILE X 129 -12.211 22.113 -0.005 0.00 0.00 H

ATOM 1982 C ILE X 129 -10.530 17.623 0.022 0.00 0.00 C

ATOM 1983 O ILE X 129 -10.372 18.140 -1.104 0.00 0.00 O

ATOM 1984 N THR X 130 -9.577 16.854 0.621 0.00 0.00 N

ATOM 1985 HN THR X 130 -9.865 16.533 1.518 0.00 0.00 H

ATOM 1986 CA THR X 130 -8.300 16.487 0.039 0.00 0.00 C

ATOM 1987 HA THR X 130 -7.778 17.424 -0.077 0.00 0.00 H

ATOM 1988 CB THR X 130 -7.462 15.605 0.985 0.00 0.00 C

ATOM 1989 HB THR X 130 -7.860 14.561 1.041 0.00 0.00 H

ATOM 1990 OG1 THR X 130 -7.447 15.972 2.334 0.00 0.00 O

ATOM 1991 HG1 THR X 130 -8.348 16.078 2.631 0.00 0.00 H

ATOM 1992 CG2 THR X 130 -6.014 15.589 0.449 0.00 0.00 C

ATOM 1993 HG21 THR X 130 -5.754 16.670 0.446 0.00 0.00 H

ATOM 1994 HG22 THR X 130 -5.868 15.090 -0.531 0.00 0.00 H

ATOM 1995 HG23 THR X 130 -5.289 15.062 1.097 0.00 0.00 H

ATOM 1996 C THR X 130 -8.420 15.870 -1.385 0.00 0.00 C

ATOM 1997 O THR X 130 -7.635 16.187 -2.268 0.00 0.00 O

ATOM 1998 N VAL X 131 -9.128 14.713 -1.527 0.00 0.00 N

ATOM 1999 HN VAL X 131 -9.571 14.362 -0.705 0.00 0.00 H

ATOM 2000 CA VAL X 131 -9.382 13.988 -2.736 0.00 0.00 C

ATOM 2001 HA VAL X 131 -8.401 13.698 -3.084 0.00 0.00 H

ATOM 2002 CB VAL X 131 -10.155 12.659 -2.490 0.00 0.00 C

ATOM 2003 HB VAL X 131 -9.473 12.038 -1.871 0.00 0.00 H

ATOM 2004 CG1 VAL X 131 -11.448 12.650 -1.653 0.00 0.00 C

ATOM 2005 HG11 VAL X 131 -12.257 13.199 -2.181 0.00 0.00 H

ATOM 2006 HG12 VAL X 131 -11.368 13.190 -0.682 0.00 0.00 H

ATOM 2007 HG13 VAL X 131 -11.800 11.606 -1.551 0.00 0.00 H

ATOM 2008 CG2 VAL X 131 -10.498 11.896 -3.798 0.00 0.00 C

ATOM 2009 HG21 VAL X 131 -10.646 10.835 -3.501 0.00 0.00 H

ATOM 2010 HG22 VAL X 131 -9.581 11.888 -4.433 0.00 0.00 H

ATOM 2011 HG23 VAL X 131 -11.356 12.391 -4.307 0.00 0.00 H

ATOM 2012 C VAL X 131 -10.051 14.846 -3.794 0.00 0.00 C

ATOM 2013 O VAL X 131 -9.777 14.747 -4.947 0.00 0.00 O

ATOM 2014 N VAL X 132 -10.952 15.780 -3.369 0.00 0.00 N

ATOM 2015 HN VAL X 132 -11.218 15.797 -2.411 0.00 0.00 H

ATOM 2016 CA VAL X 132 -11.541 16.753 -4.254 0.00 0.00 C

ATOM 2017 HA VAL X 132 -11.913 16.204 -5.105 0.00 0.00 H

ATOM 2018 CB VAL X 132 -12.710 17.501 -3.534 0.00 0.00 C

ATOM 2019 HB VAL X 132 -12.449 17.841 -2.510 0.00 0.00 H

ATOM 2020 CG1 VAL X 132 -13.177 18.853 -4.280 0.00 0.00 C

ATOM 2021 HG11 VAL X 132 -12.465 19.698 -4.309 0.00 0.00 H

ATOM 2022 HG12 VAL X 132 -14.029 19.369 -3.792 0.00 0.00 H

ATOM 2023 HG13 VAL X 132 -13.446 18.563 -5.328 0.00 0.00 H

ATOM 2024 CG2 VAL X 132 -13.967 16.646 -3.564 0.00 0.00 C

ATOM 2025 HG21 VAL X 132 -14.732 17.262 -3.033 0.00 0.00 H

ATOM 2026 HG22 VAL X 132 -13.885 15.671 -3.047 0.00 0.00 H

ATOM 2027 HG23 VAL X 132 -14.183 16.568 -4.651 0.00 0.00 H

ATOM 2028 C VAL X 132 -10.448 17.657 -4.820 0.00 0.00 C

ATOM 2029 O VAL X 132 -10.260 17.921 -6.006 0.00 0.00 O

ATOM 2030 N ILE X 133 -9.498 18.048 -3.956 0.00 0.00 N

ATOM 2031 HN ILE X 133 -9.559 17.902 -2.975 0.00 0.00 H

ATOM 2032 CA ILE X 133 -8.319 18.885 -4.205 0.00 0.00 C

ATOM 2033 HA ILE X 133 -8.738 19.732 -4.721 0.00 0.00 H

ATOM 2034 CB ILE X 133 -7.649 19.461 -3.021 0.00 0.00 C

ATOM 2035 HB ILE X 133 -7.629 18.827 -2.110 0.00 0.00 H

ATOM 2036 CG2 ILE X 133 -6.151 19.788 -3.242 0.00 0.00 C

ATOM 2037 HG21 ILE X 133 -5.993 20.558 -4.032 0.00 0.00 H

ATOM 2038 HG22 ILE X 133 -5.612 18.854 -3.495 0.00 0.00 H

ATOM 2039 HG23 ILE X 133 -5.711 20.211 -2.315 0.00 0.00 H

ATOM 2040 CG1 ILE X 133 -8.470 20.818 -2.636 0.00 0.00 C

ATOM 2041 HG11 ILE X 133 -9.537 20.569 -2.787 0.00 0.00 H

ATOM 2042 HG12 ILE X 133 -8.174 21.707 -3.238 0.00 0.00 H

ATOM 2043 CD ILE X 133 -8.290 20.995 -1.096 0.00 0.00 C

ATOM 2044 HD1 ILE X 133 -8.676 21.956 -0.688 0.00 0.00 H

ATOM 2045 HD2 ILE X 133 -7.222 20.926 -0.804 0.00 0.00 H

ATOM 2046 HD3 ILE X 133 -8.806 20.154 -0.582 0.00 0.00 H

ATOM 2047 C ILE X 133 -7.433 18.188 -5.202 0.00 0.00 C

ATOM 2048 O ILE X 133 -7.069 18.732 -6.256 0.00 0.00 O

ATOM 2049 N LEU X 134 -7.103 16.932 -4.850 0.00 0.00 N

ATOM 2050 HN LEU X 134 -7.367 16.629 -3.942 0.00 0.00 H

ATOM 2051 CA LEU X 134 -6.263 16.149 -5.725 0.00 0.00 C

ATOM 2052 HA LEU X 134 -5.354 16.702 -5.935 0.00 0.00 H

ATOM 2053 CB LEU X 134 -5.795 14.782 -5.008 0.00 0.00 C

ATOM 2054 HB1 LEU X 134 -5.250 14.191 -5.777 0.00 0.00 H

ATOM 2055 HB2 LEU X 134 -6.732 14.219 -4.821 0.00 0.00 H

ATOM 2056 CG LEU X 134 -4.945 14.763 -3.745 0.00 0.00 C

ATOM 2057 HG LEU X 134 -5.610 15.189 -2.969 0.00 0.00 H

ATOM 2058 CD1 LEU X 134 -4.418 13.468 -3.400 0.00 0.00 C

ATOM 2059 HD11 LEU X 134 -5.296 12.820 -3.231 0.00 0.00 H

ATOM 2060 HD12 LEU X 134 -3.838 13.539 -2.454 0.00 0.00 H

ATOM 2061 HD13 LEU X 134 -3.633 13.051 -4.073 0.00 0.00 H

ATOM 2062 CD2 LEU X 134 -3.733 15.807 -3.874 0.00 0.00 C

ATOM 2063 HD21 LEU X 134 -3.004 15.444 -3.115 0.00 0.00 H

ATOM 2064 HD22 LEU X 134 -4.043 16.858 -3.705 0.00 0.00 H

ATOM 2065 HD23 LEU X 134 -3.401 15.774 -4.924 0.00 0.00 H

ATOM 2066 C LEU X 134 -6.896 15.726 -7.036 0.00 0.00 C

ATOM 2067 O LEU X 134 -6.193 15.425 -8.038 0.00 0.00 O

ATOM 2068 N LEU X 135 -8.264 15.745 -7.207 0.00 0.00 N

ATOM 2069 HN LEU X 135 -8.871 15.764 -6.417 0.00 0.00 H

ATOM 2070 CA LEU X 135 -8.926 15.600 -8.550 0.00 0.00 C

ATOM 2071 HA LEU X 135 -8.482 14.801 -9.114 0.00 0.00 H

ATOM 2072 CB LEU X 135 -10.450 15.314 -8.344 0.00 0.00 C

ATOM 2073 HB1 LEU X 135 -10.801 16.066 -7.606 0.00 0.00 H

ATOM 2074 HB2 LEU X 135 -10.505 14.329 -7.851 0.00 0.00 H

ATOM 2075 CG LEU X 135 -11.230 15.319 -9.609 0.00 0.00 C

ATOM 2076 HG LEU X 135 -11.233 16.331 -10.060 0.00 0.00 H

ATOM 2077 CD1 LEU X 135 -10.788 14.164 -10.553 0.00 0.00 C

ATOM 2078 HD11 LEU X 135 -9.715 14.300 -10.808 0.00 0.00 H

ATOM 2079 HD12 LEU X 135 -11.299 14.156 -11.546 0.00 0.00 H

ATOM 2080 HD13 LEU X 135 -10.686 13.209 -9.984 0.00 0.00 H

ATOM 2081 CD2 LEU X 135 -12.763 15.048 -9.426 0.00 0.00 C

ATOM 2082 HD21 LEU X 135 -13.378 15.202 -10.330 0.00 0.00 H

ATOM 2083 HD22 LEU X 135 -13.174 15.552 -8.526 0.00 0.00 H

ATOM 2084 HD23 LEU X 135 -12.901 13.975 -9.168 0.00 0.00 H

ATOM 2085 C LEU X 135 -8.738 16.852 -9.409 0.00 0.00 C

ATOM 2086 O LEU X 135 -8.359 16.776 -10.562 0.00 0.00 O

ATOM 2087 N GLY X 136 -8.957 17.966 -8.882 0.00 0.00 N

ATOM 2088 HN GLY X 136 -9.186 18.069 -7.915 0.00 0.00 H

ATOM 2089 CA GLY X 136 -8.653 19.240 -9.528 0.00 0.00 C

ATOM 2090 HA1 GLY X 136 -8.851 20.142 -8.974 0.00 0.00 H

ATOM 2091 HA2 GLY X 136 -9.177 19.353 -10.470 0.00 0.00 H

ATOM 2092 C GLY X 136 -7.220 19.454 -10.001 0.00 0.00 C

ATOM 2093 O GLY X 136 -6.941 19.913 -11.079 0.00 0.00 O

ATOM 2094 N ILE X 137 -6.179 19.047 -9.124 0.00 0.00 N

ATOM 2095 HN ILE X 137 -6.364 18.714 -8.195 0.00 0.00 H

ATOM 2096 CA ILE X 137 -4.757 19.037 -9.321 0.00 0.00 C

ATOM 2097 HA ILE X 137 -4.443 19.988 -9.733 0.00 0.00 H

ATOM 2098 CB ILE X 137 -4.003 18.908 -8.041 0.00 0.00 C

ATOM 2099 HB ILE X 137 -4.500 18.201 -7.342 0.00 0.00 H

ATOM 2100 CG2 ILE X 137 -2.538 18.626 -8.306 0.00 0.00 C

ATOM 2101 HG21 ILE X 137 -1.964 18.906 -7.395 0.00 0.00 H

ATOM 2102 HG22 ILE X 137 -2.201 19.161 -9.212 0.00 0.00 H

ATOM 2103 HG23 ILE X 137 -2.417 17.539 -8.523 0.00 0.00 H

ATOM 2104 CG1 ILE X 137 -4.189 20.267 -7.214 0.00 0.00 C

ATOM 2105 HG11 ILE X 137 -5.220 20.687 -7.253 0.00 0.00 H

ATOM 2106 HG12 ILE X 137 -3.546 20.995 -7.756 0.00 0.00 H

ATOM 2107 CD ILE X 137 -3.661 20.190 -5.770 0.00 0.00 C

ATOM 2108 HD1 ILE X 137 -4.091 19.388 -5.128 0.00 0.00 H

ATOM 2109 HD2 ILE X 137 -3.873 21.110 -5.189 0.00 0.00 H

ATOM 2110 HD3 ILE X 137 -2.556 20.067 -5.691 0.00 0.00 H

ATOM 2111 C ILE X 137 -4.407 18.032 -10.396 0.00 0.00 C

ATOM 2112 O ILE X 137 -3.456 18.281 -11.075 0.00 0.00 O

ATOM 2113 N SER X 138 -5.173 16.907 -10.546 0.00 0.00 N

ATOM 2114 HN SER X 138 -5.833 16.673 -9.846 0.00 0.00 H

ATOM 2115 CA SER X 138 -4.927 15.828 -11.475 0.00 0.00 C

ATOM 2116 HA SER X 138 -3.881 15.642 -11.309 0.00 0.00 H

ATOM 2117 CB SER X 138 -5.762 14.539 -11.259 0.00 0.00 C

ATOM 2118 HB1 SER X 138 -5.547 13.894 -12.141 0.00 0.00 H

ATOM 2119 HB2 SER X 138 -6.842 14.803 -11.143 0.00 0.00 H

ATOM 2120 OG SER X 138 -5.231 13.827 -10.136 0.00 0.00 O

ATOM 2121 HG1 SER X 138 -5.411 14.415 -9.398 0.00 0.00 H

ATOM 2122 C SER X 138 -5.104 16.179 -12.980 0.00 0.00 C

ATOM 2123 O SER X 138 -4.325 15.662 -13.818 0.00 0.00 O

ATOM 2124 N VAL X 139 -6.123 17.012 -13.409 0.00 0.00 N

ATOM 2125 HN VAL X 139 -6.795 17.468 -12.827 0.00 0.00 H

ATOM 2126 CA VAL X 139 -6.228 17.333 -14.772 0.00 0.00 C

ATOM 2127 HA VAL X 139 -5.765 16.661 -15.473 0.00 0.00 H

ATOM 2128 CB VAL X 139 -7.666 17.452 -15.108 0.00 0.00 C

ATOM 2129 HB VAL X 139 -7.746 17.709 -16.182 0.00 0.00 H

ATOM 2130 CG1 VAL X 139 -8.293 16.127 -14.927 0.00 0.00 C

ATOM 2131 HG11 VAL X 139 -9.290 15.930 -15.386 0.00 0.00 H

ATOM 2132 HG12 VAL X 139 -8.534 15.935 -13.852 0.00 0.00 H

ATOM 2133 HG13 VAL X 139 -7.640 15.303 -15.285 0.00 0.00 H

ATOM 2134 CG2 VAL X 139 -8.363 18.525 -14.227 0.00 0.00 C

ATOM 2135 HG21 VAL X 139 -7.832 19.499 -14.304 0.00 0.00 H

ATOM 2136 HG22 VAL X 139 -8.349 18.224 -13.157 0.00 0.00 H

ATOM 2137 HG23 VAL X 139 -9.388 18.786 -14.568 0.00 0.00 H

ATOM 2138 C VAL X 139 -5.532 18.705 -15.029 0.00 0.00 C

ATOM 2139 O VAL X 139 -5.414 19.260 -16.132 0.00 0.00 O

ATOM 2140 N ALA X 140 -4.985 19.387 -13.976 0.00 0.00 N

ATOM 2141 HN ALA X 140 -5.078 19.043 -13.046 0.00 0.00 H

ATOM 2142 CA ALA X 140 -4.077 20.485 -14.263 0.00 0.00 C

ATOM 2143 HA ALA X 140 -4.491 21.066 -15.062 0.00 0.00 H

ATOM 2144 CB ALA X 140 -4.174 21.522 -13.132 0.00 0.00 C

ATOM 2145 HB1 ALA X 140 -5.251 21.702 -12.939 0.00 0.00 H

ATOM 2146 HB2 ALA X 140 -3.734 22.464 -13.520 0.00 0.00 H

ATOM 2147 HB3 ALA X 140 -3.614 21.250 -12.206 0.00 0.00 H

ATOM 2148 C ALA X 140 -2.577 20.096 -14.517 0.00 0.00 C

ATOM 2149 O ALA X 140 -1.782 20.763 -15.099 0.00 0.00 O

ATOM 2150 N GLY X 141 -2.218 18.829 -14.293 0.00 0.00 N

ATOM 2151 HN GLY X 141 -2.826 18.153 -13.891 0.00 0.00 H

ATOM 2152 CA GLY X 141 -1.011 18.236 -14.605 0.00 0.00 C

ATOM 2153 HA1 GLY X 141 -0.641 17.953 -13.629 0.00 0.00 H

ATOM 2154 HA2 GLY X 141 -0.288 18.810 -15.159 0.00 0.00 H

ATOM 2155 C GLY X 141 -1.138 17.040 -15.457 0.00 0.00 C

ATOM 2156 O GLY X 141 -2.099 16.882 -16.157 0.00 0.00 O

ATOM 2157 N MET X 142 -0.108 16.143 -15.275 0.00 0.00 N

ATOM 2158 HN MET X 142 0.641 16.389 -14.665 0.00 0.00 H

ATOM 2159 CA MET X 142 -0.225 14.777 -15.832 0.00 0.00 C

ATOM 2160 HA MET X 142 -0.975 14.275 -15.236 0.00 0.00 H

ATOM 2161 CB MET X 142 -0.402 14.753 -17.332 0.00 0.00 C

ATOM 2162 HB1 MET X 142 -1.365 15.251 -17.571 0.00 0.00 H

ATOM 2163 HB2 MET X 142 -0.429 13.722 -17.740 0.00 0.00 H

ATOM 2164 CG MET X 142 0.663 15.657 -18.032 0.00 0.00 C

ATOM 2165 HG1 MET X 142 1.446 14.918 -18.311 0.00 0.00 H

ATOM 2166 HG2 MET X 142 1.140 16.448 -17.417 0.00 0.00 H

ATOM 2167 SD MET X 142 0.122 16.393 -19.622 0.00 0.00 S

ATOM 2168 CE MET X 142 1.843 17.040 -19.921 0.00 0.00 C

ATOM 2169 HE1 MET X 142 2.265 17.510 -19.008 0.00 0.00 H

ATOM 2170 HE2 MET X 142 1.837 17.672 -20.830 0.00 0.00 H

ATOM 2171 HE3 MET X 142 2.556 16.224 -20.162 0.00 0.00 H

ATOM 2172 C MET X 142 1.048 14.092 -15.638 0.00 0.00 C

ATOM 2173 O MET X 142 1.229 13.041 -16.234 0.00 0.00 O

ATOM 2174 N GLU X 143 2.047 14.619 -14.909 0.00 0.00 N

ATOM 2175 HN GLU X 143 2.014 15.437 -14.352 0.00 0.00 H

ATOM 2176 CA GLU X 143 3.357 14.086 -15.013 0.00 0.00 C

ATOM 2177 HA GLU X 143 3.466 13.173 -15.575 0.00 0.00 H

ATOM 2178 CB GLU X 143 4.330 15.280 -15.449 0.00 0.00 C

ATOM 2179 HB1 GLU X 143 5.406 15.036 -15.316 0.00 0.00 H

ATOM 2180 HB2 GLU X 143 4.038 16.174 -14.845 0.00 0.00 H

ATOM 2181 CG GLU X 143 4.165 15.547 -16.997 0.00 0.00 C

ATOM 2182 HG1 GLU X 143 3.084 15.795 -17.081 0.00 0.00 H

ATOM 2183 HG2 GLU X 143 4.199 14.687 -17.693 0.00 0.00 H

ATOM 2184 CD GLU X 143 4.949 16.684 -17.461 0.00 0.00 C

ATOM 2185 OE1 GLU X 143 5.933 16.518 -18.240 0.00 0.00 O

ATOM 2186 OE2 GLU X 143 4.686 17.852 -17.023 0.00 0.00 O1-

ATOM 2187 C GLU X 143 3.866 13.629 -13.606 0.00 0.00 C

ATOM 2188 O GLU X 143 5.063 13.414 -13.421 0.00 0.00 O

ATOM 2189 N TRP X 144 2.920 13.383 -12.665 0.00 0.00 N

ATOM 2190 HN TRP X 144 1.987 13.607 -12.933 0.00 0.00 H

ATOM 2191 CA TRP X 144 3.025 12.816 -11.335 0.00 0.00 C

ATOM 2192 HA TRP X 144 3.424 13.553 -10.645 0.00 0.00 H

ATOM 2193 CB TRP X 144 1.604 12.647 -10.773 0.00 0.00 C

ATOM 2194 HB1 TRP X 144 1.538 12.515 -9.673 0.00 0.00 H

ATOM 2195 HB2 TRP X 144 1.137 11.712 -11.166 0.00 0.00 H

ATOM 2196 CG TRP X 144 0.747 13.779 -11.053 0.00 0.00 C

ATOM 2197 CD1 TRP X 144 -0.282 13.870 -11.988 0.00 0.00 C

ATOM 2198 HD1 TRP X 144 -0.450 13.196 -12.808 0.00 0.00 H

ATOM 2199 NE1 TRP X 144 -0.801 15.183 -11.890 0.00 0.00 N

ATOM 2200 HE1 TRP X 144 -1.640 15.477 -12.283 0.00 0.00 H

ATOM 2201 CE2 TRP X 144 -0.159 15.826 -10.965 0.00 0.00 C

ATOM 2202 CD2 TRP X 144 0.873 15.053 -10.418 0.00 0.00 C

ATOM 2203 CE3 TRP X 144 1.696 15.511 -9.421 0.00 0.00 C

ATOM 2204 HE3 TRP X 144 2.323 14.834 -8.864 0.00 0.00 H

ATOM 2205 CZ3 TRP X 144 1.568 16.824 -9.028 0.00 0.00 C

ATOM 2206 HZ3 TRP X 144 2.310 17.255 -8.361 0.00 0.00 H

ATOM 2207 CZ2 TRP X 144 -0.098 17.273 -10.772 0.00 0.00 C

ATOM 2208 HZ2 TRP X 144 -0.741 17.969 -11.292 0.00 0.00 H

ATOM 2209 CH2 TRP X 144 0.774 17.739 -9.690 0.00 0.00 C

ATOM 2210 HH2 TRP X 144 0.872 18.785 -9.426 0.00 0.00 H

ATOM 2211 C TRP X 144 3.902 11.553 -11.135 0.00 0.00 C

ATOM 2212 O TRP X 144 4.481 11.399 -10.064 0.00 0.00 O

ATOM 2213 N GLU X 145 3.943 10.641 -12.149 0.00 0.00 N

ATOM 2214 HN GLU X 145 3.567 10.870 -13.042 0.00 0.00 H

ATOM 2215 CA GLU X 145 4.635 9.308 -12.086 0.00 0.00 C

ATOM 2216 HA GLU X 145 4.833 9.025 -11.068 0.00 0.00 H

ATOM 2217 CB GLU X 145 3.718 8.216 -12.658 0.00 0.00 C

ATOM 2218 HB1 GLU X 145 2.704 8.171 -12.211 0.00 0.00 H

ATOM 2219 HB2 GLU X 145 4.160 7.273 -12.255 0.00 0.00 H

ATOM 2220 CG GLU X 145 3.600 8.179 -14.191 0.00 0.00 C

ATOM 2221 HG1 GLU X 145 4.628 8.092 -14.607 0.00 0.00 H

ATOM 2222 HG2 GLU X 145 3.000 9.031 -14.585 0.00 0.00 H

ATOM 2223 CD GLU X 145 2.828 7.051 -14.765 0.00 0.00 C

ATOM 2224 OE1 GLU X 145 1.956 6.592 -14.053 0.00 0.00 O

ATOM 2225 OE2 GLU X 145 3.277 6.427 -15.753 0.00 0.00 O1-

ATOM 2226 C GLU X 145 5.948 9.536 -12.916 0.00 0.00 C

ATOM 2227 O GLU X 145 6.803 8.635 -12.964 0.00 0.00 O

ATOM 2228 N ALA X 146 6.147 10.618 -13.661 0.00 0.00 N

ATOM 2229 HN ALA X 146 5.516 11.377 -13.528 0.00 0.00 H

ATOM 2230 CA ALA X 146 7.097 10.609 -14.797 0.00 0.00 C

ATOM 2231 HA ALA X 146 7.570 9.646 -14.853 0.00 0.00 H

ATOM 2232 CB ALA X 146 6.255 10.901 -16.058 0.00 0.00 C

ATOM 2233 HB1 ALA X 146 6.931 11.316 -16.838 0.00 0.00 H

ATOM 2234 HB2 ALA X 146 5.499 11.670 -15.778 0.00 0.00 H

ATOM 2235 HB3 ALA X 146 5.735 9.986 -16.396 0.00 0.00 H

ATOM 2236 C ALA X 146 8.217 11.614 -14.600 0.00 0.00 C

ATOM 2237 O ALA X 146 9.216 11.570 -15.184 0.00 0.00 O

ATOM 2238 N LYS X 147 8.043 12.592 -13.696 0.00 0.00 N

ATOM 2239 HN LYS X 147 7.156 12.644 -13.242 0.00 0.00 H

ATOM 2240 CA LYS X 147 8.948 13.709 -13.400 0.00 0.00 C

ATOM 2241 HA LYS X 147 9.866 13.639 -13.960 0.00 0.00 H

ATOM 2242 CB LYS X 147 8.320 15.087 -13.791 0.00 0.00 C

ATOM 2243 HB1 LYS X 147 9.027 15.916 -13.578 0.00 0.00 H

ATOM 2244 HB2 LYS X 147 7.409 15.231 -13.175 0.00 0.00 H

ATOM 2245 CG LYS X 147 8.021 15.233 -15.396 0.00 0.00 C

ATOM 2246 HG1 LYS X 147 7.728 16.278 -15.619 0.00 0.00 H

ATOM 2247 HG2 LYS X 147 7.276 14.442 -15.597 0.00 0.00 H

ATOM 2248 CD LYS X 147 9.283 15.039 -16.234 0.00 0.00 C

ATOM 2249 HD1 LYS X 147 9.698 14.044 -15.981 0.00 0.00 H

ATOM 2250 HD2 LYS X 147 10.007 15.873 -16.150 0.00 0.00 H

ATOM 2251 CE LYS X 147 8.874 14.853 -17.698 0.00 0.00 C

ATOM 2252 HE1 LYS X 147 8.094 14.091 -17.863 0.00 0.00 H

ATOM 2253 HE2 LYS X 147 9.702 14.493 -18.339 0.00 0.00 H

ATOM 2254 NZ LYS X 147 8.451 16.175 -18.255 0.00 0.00 N1+

ATOM 2255 HZ1 LYS X 147 8.725 16.504 -19.205 0.00 0.00 H

ATOM 2256 HZ2 LYS X 147 8.711 16.884 -17.538 0.00 0.00 H

ATOM 2257 HZ3 LYS X 147 7.413 16.126 -18.325 0.00 0.00 H

ATOM 2258 C LYS X 147 9.355 13.717 -11.904 0.00 0.00 C

ATOM 2259 O LYS X 147 8.481 13.568 -11.042 0.00 0.00 O

ATOM 2260 N ALA X 148 10.628 13.952 -11.619 0.00 0.00 N

ATOM 2261 HN ALA X 148 11.223 14.058 -12.408 0.00 0.00 H

ATOM 2262 CA ALA X 148 11.245 14.220 -10.310 0.00 0.00 C

ATOM 2263 HA ALA X 148 12.277 14.462 -10.475 0.00 0.00 H

ATOM 2264 CB ALA X 148 10.539 15.379 -9.637 0.00 0.00 C

ATOM 2265 HB1 ALA X 148 10.953 15.822 -8.704 0.00 0.00 H

ATOM 2266 HB2 ALA X 148 9.496 15.191 -9.295 0.00 0.00 H

ATOM 2267 HB3 ALA X 148 10.471 16.227 -10.356 0.00 0.00 H

ATOM 2268 C ALA X 148 11.257 13.040 -9.363 0.00 0.00 C

ATOM 2269 O ALA X 148 11.404 13.209 -8.159 0.00 0.00 O

ATOM 2270 N GLN X 149 11.141 11.818 -9.940 0.00 0.00 N

ATOM 2271 HN GLN X 149 11.262 11.642 -10.913 0.00 0.00 H

ATOM 2272 CA GLN X 149 10.904 10.593 -9.169 0.00 0.00 C

ATOM 2273 HA GLN X 149 10.143 10.828 -8.441 0.00 0.00 H

ATOM 2274 CB GLN X 149 10.560 9.365 -10.082 0.00 0.00 C

ATOM 2275 HB1 GLN X 149 10.334 8.479 -9.453 0.00 0.00 H

ATOM 2276 HB2 GLN X 149 11.322 9.136 -10.861 0.00 0.00 H

ATOM 2277 CG GLN X 149 9.120 9.644 -10.913 0.00 0.00 C

ATOM 2278 HG1 GLN X 149 9.087 8.734 -11.552 0.00 0.00 H

ATOM 2279 HG2 GLN X 149 9.154 10.485 -11.641 0.00 0.00 H

ATOM 2280 CD GLN X 149 7.937 9.737 -10.050 0.00 0.00 C

ATOM 2281 OE1 GLN X 149 7.345 8.868 -9.372 0.00 0.00 O

ATOM 2282 NE2 GLN X 149 7.386 10.980 -10.042 0.00 0.00 N

ATOM 2283 HE21 GLN X 149 6.495 11.169 -9.627 0.00 0.00 H

ATOM 2284 HE22 GLN X 149 7.864 11.684 -10.581 0.00 0.00 H

ATOM 2285 C GLN X 149 11.986 10.185 -8.156 0.00 0.00 C

ATOM 2286 O GLN X 149 11.757 9.893 -6.982 0.00 0.00 O

ATOM 2287 N ILE X 150 13.254 10.164 -8.480 0.00 0.00 N

ATOM 2288 HN ILE X 150 13.623 10.691 -9.241 0.00 0.00 H

ATOM 2289 CA ILE X 150 14.292 10.021 -7.521 0.00 0.00 C

ATOM 2290 HA ILE X 150 14.116 9.075 -7.044 0.00 0.00 H

ATOM 2291 CB ILE X 150 15.658 9.861 -8.268 0.00 0.00 C

ATOM 2292 HB ILE X 150 15.910 10.821 -8.748 0.00 0.00 H

ATOM 2293 CG2 ILE X 150 16.689 9.440 -7.281 0.00 0.00 C

ATOM 2294 HG21 ILE X 150 16.381 8.537 -6.721 0.00 0.00 H

ATOM 2295 HG22 ILE X 150 17.026 10.275 -6.634 0.00 0.00 H

ATOM 2296 HG23 ILE X 150 17.631 9.150 -7.794 0.00 0.00 H

ATOM 2297 CG1 ILE X 150 15.445 8.855 -9.425 0.00 0.00 C

ATOM 2298 HG11 ILE X 150 14.573 9.028 -10.096 0.00 0.00 H

ATOM 2299 HG12 ILE X 150 15.253 7.877 -8.927 0.00 0.00 H

ATOM 2300 CD ILE X 150 16.622 8.790 -10.320 0.00 0.00 C

ATOM 2301 HD1 ILE X 150 16.879 9.789 -10.721 0.00 0.00 H

ATOM 2302 HD2 ILE X 150 16.336 8.034 -11.077 0.00 0.00 H

ATOM 2303 HD3 ILE X 150 17.517 8.368 -9.821 0.00 0.00 H

ATOM 2304 C ILE X 150 14.467 11.038 -6.450 0.00 0.00 C

ATOM 2305 O ILE X 150 14.774 10.704 -5.276 0.00 0.00 O

ATOM 2306 N VAL X 151 14.153 12.299 -6.750 0.00 0.00 N

ATOM 2307 HN VAL X 151 13.773 12.552 -7.635 0.00 0.00 H

ATOM 2308 CA VAL X 151 14.200 13.387 -5.768 0.00 0.00 C

ATOM 2309 HA VAL X 151 15.220 13.566 -5.457 0.00 0.00 H

ATOM 2310 CB VAL X 151 13.733 14.833 -6.187 0.00 0.00 C

ATOM 2311 HB VAL X 151 12.659 14.654 -6.404 0.00 0.00 H

ATOM 2312 CG1 VAL X 151 13.916 15.850 -5.056 0.00 0.00 C

ATOM 2313 HG11 VAL X 151 13.515 16.884 -5.188 0.00 0.00 H

ATOM 2314 HG12 VAL X 151 14.996 16.097 -4.894 0.00 0.00 H

ATOM 2315 HG13 VAL X 151 13.496 15.624 -4.058 0.00 0.00 H

ATOM 2316 CG2 VAL X 151 14.409 15.179 -7.548 0.00 0.00 C

ATOM 2317 HG21 VAL X 151 14.006 14.586 -8.399 0.00 0.00 H

ATOM 2318 HG22 VAL X 151 15.515 15.122 -7.605 0.00 0.00 H

ATOM 2319 HG23 VAL X 151 14.095 16.219 -7.782 0.00 0.00 H

ATOM 2320 C VAL X 151 13.309 13.122 -4.527 0.00 0.00 C

ATOM 2321 O VAL X 151 13.791 13.106 -3.395 0.00 0.00 O

ATOM 2322 N LEU X 152 12.079 12.672 -4.733 0.00 0.00 N

ATOM 2323 HN LEU X 152 11.749 12.732 -5.680 0.00 0.00 H

ATOM 2324 CA LEU X 152 11.053 12.152 -3.812 0.00 0.00 C

ATOM 2325 HA LEU X 152 11.106 12.933 -3.062 0.00 0.00 H

ATOM 2326 CB LEU X 152 9.776 11.902 -4.620 0.00 0.00 C

ATOM 2327 HB1 LEU X 152 8.974 11.439 -4.012 0.00 0.00 H

ATOM 2328 HB2 LEU X 152 9.964 11.164 -5.428 0.00 0.00 H

ATOM 2329 CG LEU X 152 9.178 13.137 -5.312 0.00 0.00 C

ATOM 2330 HG LEU X 152 9.987 13.472 -6.002 0.00 0.00 H

ATOM 2331 CD1 LEU X 152 7.902 12.819 -6.176 0.00 0.00 C

ATOM 2332 HD11 LEU X 152 7.254 12.114 -5.615 0.00 0.00 H

ATOM 2333 HD12 LEU X 152 8.234 12.420 -7.152 0.00 0.00 H

ATOM 2334 HD13 LEU X 152 7.353 13.772 -6.349 0.00 0.00 H

ATOM 2335 CD2 LEU X 152 8.877 14.387 -4.369 0.00 0.00 C

ATOM 2336 HD21 LEU X 152 8.248 14.074 -3.510 0.00 0.00 H

ATOM 2337 HD22 LEU X 152 8.319 15.189 -4.886 0.00 0.00 H

ATOM 2338 HD23 LEU X 152 9.854 14.826 -4.070 0.00 0.00 H

ATOM 2339 C LEU X 152 11.488 10.799 -3.100 0.00 0.00 C

ATOM 2340 O LEU X 152 11.411 10.797 -1.878 0.00 0.00 O

ATOM 2341 N LEU X 153 12.120 9.897 -3.828 0.00 0.00 N

ATOM 2342 HN LEU X 153 12.334 9.977 -4.798 0.00 0.00 H

ATOM 2343 CA LEU X 153 12.679 8.704 -3.164 0.00 0.00 C

ATOM 2344 HA LEU X 153 11.815 8.307 -2.657 0.00 0.00 H

ATOM 2345 CB LEU X 153 13.250 7.620 -4.219 0.00 0.00 C

ATOM 2346 HB1 LEU X 153 14.023 8.109 -4.842 0.00 0.00 H

ATOM 2347 HB2 LEU X 153 12.377 7.546 -4.904 0.00 0.00 H

ATOM 2348 CG LEU X 153 13.705 6.325 -3.700 0.00 0.00 C

ATOM 2349 HG LEU X 153 14.694 6.425 -3.206 0.00 0.00 H

ATOM 2350 CD1 LEU X 153 12.835 5.615 -2.629 0.00 0.00 C

ATOM 2351 HD11 LEU X 153 11.832 5.463 -3.091 0.00 0.00 H

ATOM 2352 HD12 LEU X 153 12.940 6.080 -1.620 0.00 0.00 H

ATOM 2353 HD13 LEU X 153 13.244 4.603 -2.477 0.00 0.00 H

ATOM 2354 CD2 LEU X 153 14.152 5.383 -4.869 0.00 0.00 C

ATOM 2355 HD21 LEU X 153 13.251 5.033 -5.418 0.00 0.00 H

ATOM 2356 HD22 LEU X 153 14.576 4.476 -4.380 0.00 0.00 H

ATOM 2357 HD23 LEU X 153 14.952 5.697 -5.579 0.00 0.00 H

ATOM 2358 C LEU X 153 13.777 9.022 -2.147 0.00 0.00 C

ATOM 2359 O LEU X 153 13.876 8.435 -1.100 0.00 0.00 O

ATOM 2360 N VAL X 154 14.685 9.929 -2.502 0.00 0.00 N

ATOM 2361 HN VAL X 154 14.615 10.402 -3.378 0.00 0.00 H

ATOM 2362 CA VAL X 154 15.799 10.381 -1.644 0.00 0.00 C

ATOM 2363 HA VAL X 154 16.268 9.490 -1.251 0.00 0.00 H

ATOM 2364 CB VAL X 154 16.850 11.175 -2.459 0.00 0.00 C

ATOM 2365 HB VAL X 154 16.208 11.793 -3.115 0.00 0.00 H

ATOM 2366 CG1 VAL X 154 17.692 12.058 -1.539 0.00 0.00 C

ATOM 2367 HG11 VAL X 154 18.182 11.373 -0.815 0.00 0.00 H

ATOM 2368 HG12 VAL X 154 17.110 12.874 -1.062 0.00 0.00 H

ATOM 2369 HG13 VAL X 154 18.485 12.418 -2.232 0.00 0.00 H

ATOM 2370 CG2 VAL X 154 17.645 10.211 -3.367 0.00 0.00 C

ATOM 2371 HG21 VAL X 154 18.425 9.663 -2.782 0.00 0.00 H

ATOM 2372 HG22 VAL X 154 18.150 10.774 -4.176 0.00 0.00 H

ATOM 2373 HG23 VAL X 154 16.970 9.506 -3.888 0.00 0.00 H

ATOM 2374 C VAL X 154 15.333 11.114 -0.403 0.00 0.00 C

ATOM 2375 O VAL X 154 15.846 10.869 0.768 0.00 0.00 O

ATOM 2376 N ILE X 155 14.267 11.934 -0.533 0.00 0.00 N

ATOM 2377 HN ILE X 155 13.925 12.097 -1.458 0.00 0.00 H

ATOM 2378 CA ILE X 155 13.498 12.462 0.608 0.00 0.00 C

ATOM 2379 HA ILE X 155 14.164 12.910 1.326 0.00 0.00 H

ATOM 2380 CB ILE X 155 12.430 13.509 0.372 0.00 0.00 C

ATOM 2381 HB ILE X 155 11.530 13.194 -0.180 0.00 0.00 H

ATOM 2382 CG2 ILE X 155 11.830 13.882 1.844 0.00 0.00 C

ATOM 2383 HG21 ILE X 155 11.231 13.123 2.393 0.00 0.00 H

ATOM 2384 HG22 ILE X 155 11.275 14.849 1.800 0.00 0.00 H

ATOM 2385 HG23 ILE X 155 12.710 14.223 2.421 0.00 0.00 H

ATOM 2386 CG1 ILE X 155 13.061 14.783 -0.279 0.00 0.00 C

ATOM 2387 HG11 ILE X 155 13.592 14.534 -1.223 0.00 0.00 H

ATOM 2388 HG12 ILE X 155 13.589 15.397 0.473 0.00 0.00 H

ATOM 2389 CD ILE X 155 11.911 15.664 -0.867 0.00 0.00 C

ATOM 2390 HD1 ILE X 155 11.436 16.215 -0.027 0.00 0.00 H

ATOM 2391 HD2 ILE X 155 11.107 15.074 -1.339 0.00 0.00 H

ATOM 2392 HD3 ILE X 155 12.296 16.559 -1.418 0.00 0.00 H

ATOM 2393 C ILE X 155 12.852 11.398 1.536 0.00 0.00 C

ATOM 2394 O ILE X 155 12.963 11.490 2.792 0.00 0.00 O

ATOM 2395 N LEU X 156 12.326 10.229 0.961 0.00 0.00 N

ATOM 2396 HN LEU X 156 12.218 10.105 -0.020 0.00 0.00 H

ATOM 2397 CA LEU X 156 11.856 9.028 1.715 0.00 0.00 C

ATOM 2398 HA LEU X 156 11.174 9.208 2.536 0.00 0.00 H

ATOM 2399 CB LEU X 156 11.189 7.978 0.765 0.00 0.00 C

ATOM 2400 HB1 LEU X 156 10.963 7.091 1.384 0.00 0.00 H

ATOM 2401 HB2 LEU X 156 11.921 7.555 0.036 0.00 0.00 H

ATOM 2402 CG LEU X 156 9.962 8.367 -0.040 0.00 0.00 C

ATOM 2403 HG LEU X 156 10.286 9.120 -0.791 0.00 0.00 H

ATOM 2404 CD1 LEU X 156 9.292 7.211 -0.783 0.00 0.00 C

ATOM 2405 HD11 LEU X 156 10.123 6.810 -1.391 0.00 0.00 H

ATOM 2406 HD12 LEU X 156 8.382 7.571 -1.319 0.00 0.00 H

ATOM 2407 HD13 LEU X 156 9.038 6.330 -0.157 0.00 0.00 H

ATOM 2408 CD2 LEU X 156 8.833 8.970 0.835 0.00 0.00 C

ATOM 2409 HD21 LEU X 156 9.066 9.792 1.542 0.00 0.00 H

ATOM 2410 HD22 LEU X 156 8.452 8.197 1.540 0.00 0.00 H

ATOM 2411 HD23 LEU X 156 7.918 9.250 0.273 0.00 0.00 H

ATOM 2412 C LEU X 156 13.076 8.386 2.466 0.00 0.00 C

ATOM 2413 O LEU X 156 13.001 8.223 3.658 0.00 0.00 O

ATOM 2414 N LEU X 157 14.225 8.184 1.768 0.00 0.00 N

ATOM 2415 HN LEU X 157 14.338 8.341 0.792 0.00 0.00 H

ATOM 2416 CA LEU X 157 15.446 7.628 2.351 0.00 0.00 C

ATOM 2417 HA LEU X 157 15.069 6.728 2.824 0.00 0.00 H

ATOM 2418 CB LEU X 157 16.464 7.333 1.272 0.00 0.00 C

ATOM 2419 HB1 LEU X 157 17.489 7.299 1.679 0.00 0.00 H

ATOM 2420 HB2 LEU X 157 16.456 8.198 0.569 0.00 0.00 H

ATOM 2421 CG LEU X 157 16.209 6.060 0.391 0.00 0.00 C

ATOM 2422 HG LEU X 157 15.161 5.907 0.055 0.00 0.00 H

ATOM 2423 CD1 LEU X 157 17.101 6.017 -0.809 0.00 0.00 C

ATOM 2424 HD11 LEU X 157 16.981 5.090 -1.416 0.00 0.00 H

ATOM 2425 HD12 LEU X 157 18.143 6.158 -0.443 0.00 0.00 H

ATOM 2426 HD13 LEU X 157 16.908 6.900 -1.453 0.00 0.00 H

ATOM 2427 CD2 LEU X 157 16.482 4.902 1.365 0.00 0.00 C

ATOM 2428 HD21 LEU X 157 15.752 4.759 2.192 0.00 0.00 H

ATOM 2429 HD22 LEU X 157 17.498 4.986 1.793 0.00 0.00 H

ATOM 2430 HD23 LEU X 157 16.502 3.963 0.784 0.00 0.00 H

ATOM 2431 C LEU X 157 16.016 8.446 3.498 0.00 0.00 C

ATOM 2432 O LEU X 157 16.419 7.883 4.477 0.00 0.00 O

ATOM 2433 N LEU X 158 15.853 9.762 3.474 0.00 0.00 N

ATOM 2434 HN LEU X 158 15.292 10.143 2.732 0.00 0.00 H

ATOM 2435 CA LEU X 158 16.295 10.673 4.510 0.00 0.00 C

ATOM 2436 HA LEU X 158 17.183 10.233 4.918 0.00 0.00 H

ATOM 2437 CB LEU X 158 16.436 12.091 3.937 0.00 0.00 C

ATOM 2438 HB1 LEU X 158 15.530 12.099 3.293 0.00 0.00 H

ATOM 2439 HB2 LEU X 158 17.261 12.299 3.217 0.00 0.00 H

ATOM 2440 CG LEU X 158 16.517 13.355 4.800 0.00 0.00 C

ATOM 2441 HG LEU X 158 15.531 13.444 5.301 0.00 0.00 H

ATOM 2442 CD1 LEU X 158 17.552 13.426 5.825 0.00 0.00 C

ATOM 2443 HD11 LEU X 158 17.438 12.532 6.477 0.00 0.00 H

ATOM 2444 HD12 LEU X 158 17.310 14.214 6.563 0.00 0.00 H

ATOM 2445 HD13 LEU X 158 18.577 13.521 5.427 0.00 0.00 H

ATOM 2446 CD2 LEU X 158 16.632 14.633 3.870 0.00 0.00 C

ATOM 2447 HD21 LEU X 158 16.432 15.489 4.548 0.00 0.00 H

ATOM 2448 HD22 LEU X 158 15.921 14.657 3.015 0.00 0.00 H

ATOM 2449 HD23 LEU X 158 17.681 14.725 3.505 0.00 0.00 H

ATOM 2450 C LEU X 158 15.333 10.642 5.687 0.00 0.00 C

ATOM 2451 O LEU X 158 15.755 10.892 6.846 0.00 0.00 O

ATOM 2452 N ALA X 159 14.027 10.395 5.471 0.00 0.00 N

ATOM 2453 HN ALA X 159 13.667 10.081 4.595 0.00 0.00 H

ATOM 2454 CA ALA X 159 13.107 10.249 6.660 0.00 0.00 C

ATOM 2455 HA ALA X 159 13.243 11.013 7.411 0.00 0.00 H

ATOM 2456 CB ALA X 159 11.722 10.446 6.194 0.00 0.00 C

ATOM 2457 HB1 ALA X 159 10.943 10.202 6.961 0.00 0.00 H

ATOM 2458 HB2 ALA X 159 11.582 9.770 5.316 0.00 0.00 H

ATOM 2459 HB3 ALA X 159 11.396 11.473 5.918 0.00 0.00 H

ATOM 2460 C ALA X 159 13.409 8.929 7.386 0.00 0.00 C

ATOM 2461 O ALA X 159 13.392 8.816 8.651 0.00 0.00 O

ATOM 2462 N ILE X 160 13.713 7.874 6.602 0.00 0.00 N

ATOM 2463 HN ILE X 160 13.629 8.056 5.623 0.00 0.00 H

ATOM 2464 CA ILE X 160 14.234 6.532 7.161 0.00 0.00 C

ATOM 2465 HA ILE X 160 13.545 6.124 7.879 0.00 0.00 H

ATOM 2466 CB ILE X 160 14.476 5.495 6.026 0.00 0.00 C

ATOM 2467 HB ILE X 160 15.194 5.933 5.299 0.00 0.00 H

ATOM 2468 CG2 ILE X 160 15.102 4.176 6.595 0.00 0.00 C

ATOM 2469 HG21 ILE X 160 15.232 3.436 5.782 0.00 0.00 H

ATOM 2470 HG22 ILE X 160 14.405 3.691 7.318 0.00 0.00 H

ATOM 2471 HG23 ILE X 160 16.119 4.311 7.030 0.00 0.00 H

ATOM 2472 CG1 ILE X 160 13.100 5.090 5.369 0.00 0.00 C

ATOM 2473 HG11 ILE X 160 12.577 6.059 5.187 0.00 0.00 H

ATOM 2474 HG12 ILE X 160 12.490 4.478 6.069 0.00 0.00 H

ATOM 2475 CD ILE X 160 13.269 4.325 4.017 0.00 0.00 C

ATOM 2476 HD1 ILE X 160 12.297 4.221 3.494 0.00 0.00 H

ATOM 2477 HD2 ILE X 160 13.809 3.366 4.190 0.00 0.00 H

ATOM 2478 HD3 ILE X 160 13.884 5.012 3.393 0.00 0.00 H

ATOM 2479 C ILE X 160 15.541 6.653 7.941 0.00 0.00 C

ATOM 2480 O ILE X 160 15.767 5.938 8.930 0.00 0.00 O

ATOM 2481 N GLY X 161 16.475 7.524 7.370 0.00 0.00 N

ATOM 2482 HN GLY X 161 16.430 7.772 6.411 0.00 0.00 H

ATOM 2483 CA GLY X 161 17.696 7.981 8.017 0.00 0.00 C

ATOM 2484 HA1 GLY X 161 18.196 8.698 7.379 0.00 0.00 H

ATOM 2485 HA2 GLY X 161 18.311 7.109 8.193 0.00 0.00 H

ATOM 2486 C GLY X 161 17.524 8.769 9.368 0.00 0.00 C

ATOM 2487 O GLY X 161 18.203 8.512 10.356 0.00 0.00 O

ATOM 2488 N ASP X 162 16.505 9.594 9.403 0.00 0.00 N

ATOM 2489 HN ASP X 162 16.003 9.771 8.560 0.00 0.00 H

ATOM 2490 CA ASP X 162 16.103 10.346 10.559 0.00 0.00 C

ATOM 2491 HA ASP X 162 16.981 10.913 10.860 0.00 0.00 H

ATOM 2492 CB ASP X 162 14.953 11.299 10.139 0.00 0.00 C

ATOM 2493 HB1 ASP X 162 14.274 10.817 9.411 0.00 0.00 H

ATOM 2494 HB2 ASP X 162 15.543 11.990 9.492 0.00 0.00 H

ATOM 2495 CG ASP X 162 14.115 11.923 11.165 0.00 0.00 C

ATOM 2496 OD1 ASP X 162 14.124 13.179 11.355 0.00 0.00 O

ATOM 2497 OD2 ASP X 162 13.267 11.173 11.748 0.00 0.00 O1-

ATOM 2498 C ASP X 162 15.730 9.411 11.720 0.00 0.00 C

ATOM 2499 O ASP X 162 16.022 9.724 12.911 0.00 0.00 O

ATOM 2500 N PHE X 163 15.090 8.276 11.413 0.00 0.00 N

ATOM 2501 HN PHE X 163 14.747 8.231 10.471 0.00 0.00 H

ATOM 2502 CA PHE X 163 14.597 7.311 12.321 0.00 0.00 C

ATOM 2503 HA PHE X 163 14.121 7.888 13.097 0.00 0.00 H

ATOM 2504 CB PHE X 163 13.747 6.196 11.687 0.00 0.00 C

ATOM 2505 HB1 PHE X 163 14.281 5.566 10.950 0.00 0.00 H

ATOM 2506 HB2 PHE X 163 13.028 6.766 11.056 0.00 0.00 H

ATOM 2507 CG PHE X 163 12.985 5.373 12.646 0.00 0.00 C

ATOM 2508 CD1 PHE X 163 11.620 5.519 12.589 0.00 0.00 C

ATOM 2509 HD1 PHE X 163 11.192 6.299 11.986 0.00 0.00 H

ATOM 2510 CE1 PHE X 163 10.777 4.747 13.344 0.00 0.00 C

ATOM 2511 HE1 PHE X 163 9.701 4.797 13.385 0.00 0.00 H

ATOM 2512 CZ PHE X 163 11.355 3.801 14.297 0.00 0.00 C

ATOM 2513 HZ PHE X 163 10.676 3.377 15.027 0.00 0.00 H

ATOM 2514 CD2 PHE X 163 13.559 4.326 13.414 0.00 0.00 C

ATOM 2515 HD2 PHE X 163 14.590 4.008 13.315 0.00 0.00 H

ATOM 2516 CE2 PHE X 163 12.686 3.519 14.239 0.00 0.00 C

ATOM 2517 HE2 PHE X 163 13.010 2.602 14.708 0.00 0.00 H

ATOM 2518 C PHE X 163 15.755 6.683 12.952 0.00 0.00 C

ATOM 2519 O PHE X 163 15.926 6.566 14.158 0.00 0.00 O

ATOM 2520 N VAL X 164 16.706 6.176 12.142 0.00 0.00 N

ATOM 2521 HN VAL X 164 16.631 6.378 11.173 0.00 0.00 H

ATOM 2522 CA VAL X 164 17.796 5.416 12.584 0.00 0.00 C

ATOM 2523 HA VAL X 164 17.399 4.574 13.131 0.00 0.00 H

ATOM 2524 CB VAL X 164 18.519 4.856 11.359 0.00 0.00 C

ATOM 2525 HB VAL X 164 18.724 5.693 10.650 0.00 0.00 H

ATOM 2526 CG1 VAL X 164 19.704 4.027 11.805 0.00 0.00 C

ATOM 2527 HG11 VAL X 164 20.541 4.629 12.200 0.00 0.00 H

ATOM 2528 HG12 VAL X 164 20.073 3.438 10.937 0.00 0.00 H

ATOM 2529 HG13 VAL X 164 19.390 3.211 12.491 0.00 0.00 H

ATOM 2530 CG2 VAL X 164 17.576 3.860 10.546 0.00 0.00 C

ATOM 2531 HG21 VAL X 164 18.159 3.695 9.612 0.00 0.00 H

ATOM 2532 HG22 VAL X 164 16.555 4.279 10.366 0.00 0.00 H

ATOM 2533 HG23 VAL X 164 17.427 2.935 11.136 0.00 0.00 H

ATOM 2534 C VAL X 164 18.733 6.102 13.538 0.00 0.00 C

ATOM 2535 O VAL X 164 19.093 5.527 14.553 0.00 0.00 O

ATOM 2536 N ILE X 165 19.147 7.316 13.321 0.00 0.00 N

ATOM 2537 HN ILE X 165 19.032 7.783 12.451 0.00 0.00 H

ATOM 2538 CA ILE X 165 20.044 7.994 14.241 0.00 0.00 C

ATOM 2539 HA ILE X 165 20.638 7.233 14.709 0.00 0.00 H

ATOM 2540 CB ILE X 165 20.769 9.153 13.527 0.00 0.00 C

ATOM 2541 HB ILE X 165 21.356 8.647 12.727 0.00 0.00 H

ATOM 2542 CG2 ILE X 165 19.814 10.139 12.934 0.00 0.00 C

ATOM 2543 HG21 ILE X 165 18.935 9.720 12.392 0.00 0.00 H

ATOM 2544 HG22 ILE X 165 20.325 10.874 12.286 0.00 0.00 H

ATOM 2545 HG23 ILE X 165 19.467 10.884 13.683 0.00 0.00 H

ATOM 2546 CG1 ILE X 165 21.792 9.734 14.447 0.00 0.00 C

ATOM 2547 HG11 ILE X 165 21.194 10.220 15.247 0.00 0.00 H

ATOM 2548 HG12 ILE X 165 22.206 10.524 13.794 0.00 0.00 H

ATOM 2549 CD ILE X 165 22.881 8.887 15.056 0.00 0.00 C

ATOM 2550 HD1 ILE X 165 23.536 8.497 14.246 0.00 0.00 H

ATOM 2551 HD2 ILE X 165 22.472 8.042 15.642 0.00 0.00 H

ATOM 2552 HD3 ILE X 165 23.604 9.457 15.681 0.00 0.00 H

ATOM 2553 C ILE X 165 19.287 8.552 15.442 0.00 0.00 C

ATOM 2554 O ILE X 165 19.765 8.708 16.532 0.00 0.00 O

ATOM 2555 N GLY X 166 18.027 8.901 15.210 0.00 0.00 N

ATOM 2556 HN GLY X 166 17.736 8.722 14.273 0.00 0.00 H

ATOM 2557 CA GLY X 166 17.064 9.446 16.178 0.00 0.00 C

ATOM 2558 HA1 GLY X 166 16.150 9.530 15.599 0.00 0.00 H

ATOM 2559 HA2 GLY X 166 17.479 10.328 16.618 0.00 0.00 H

ATOM 2560 C GLY X 166 16.651 8.472 17.246 0.00 0.00 C

ATOM 2561 O GLY X 166 16.340 8.823 18.389 0.00 0.00 O

ATOM 2562 N THR X 167 16.788 7.215 16.861 0.00 0.00 N

ATOM 2563 HN THR X 167 16.981 6.947 15.922 0.00 0.00 H

ATOM 2564 CA THR X 167 16.695 6.187 17.836 0.00 0.00 C

ATOM 2565 HA THR X 167 16.075 6.527 18.643 0.00 0.00 H

ATOM 2566 CB THR X 167 16.028 4.857 17.388 0.00 0.00 C

ATOM 2567 HB THR X 167 16.160 3.941 18.007 0.00 0.00 H

ATOM 2568 OG1 THR X 167 16.412 4.448 16.086 0.00 0.00 O

ATOM 2569 HG1 THR X 167 16.155 5.105 15.430 0.00 0.00 H

ATOM 2570 CG2 THR X 167 14.537 5.157 17.306 0.00 0.00 C

ATOM 2571 HG21 THR X 167 14.267 5.587 18.297 0.00 0.00 H

ATOM 2572 HG22 THR X 167 13.990 4.186 17.210 0.00 0.00 H

ATOM 2573 HG23 THR X 167 14.298 5.823 16.450 0.00 0.00 H

ATOM 2574 C THR X 167 17.957 5.798 18.473 0.00 0.00 C

ATOM 2575 O THR X 167 17.993 5.062 19.444 0.00 0.00 O

ATOM 2576 N PHE X 168 19.119 6.371 18.058 0.00 0.00 N

ATOM 2577 HN PHE X 168 19.091 6.887 17.204 0.00 0.00 H

ATOM 2578 CA PHE X 168 20.317 6.312 18.848 0.00 0.00 C

ATOM 2579 HA PHE X 168 20.360 5.360 19.360 0.00 0.00 H

ATOM 2580 CB PHE X 168 21.525 6.302 17.851 0.00 0.00 C

ATOM 2581 HB1 PHE X 168 21.406 7.261 17.311 0.00 0.00 H

ATOM 2582 HB2 PHE X 168 21.470 5.450 17.126 0.00 0.00 H

ATOM 2583 CG PHE X 168 22.854 6.163 18.570 0.00 0.00 C

ATOM 2584 CD1 PHE X 168 23.381 5.008 19.138 0.00 0.00 C

ATOM 2585 HD1 PHE X 168 22.929 4.050 18.941 0.00 0.00 H

ATOM 2586 CE1 PHE X 168 24.600 4.942 19.860 0.00 0.00 C

ATOM 2587 HE1 PHE X 168 25.060 3.988 20.105 0.00 0.00 H

ATOM 2588 CZ PHE X 168 25.347 6.139 20.094 0.00 0.00 C

ATOM 2589 HZ PHE X 168 26.262 6.142 20.688 0.00 0.00 H

ATOM 2590 CD2 PHE X 168 23.593 7.359 18.753 0.00 0.00 C

ATOM 2591 HD2 PHE X 168 23.221 8.356 18.566 0.00 0.00 H

ATOM 2592 CE2 PHE X 168 24.739 7.304 19.557 0.00 0.00 C

ATOM 2593 HE2 PHE X 168 25.240 8.244 19.731 0.00 0.00 H

ATOM 2594 C PHE X 168 20.336 7.632 19.720 0.00 0.00 C

ATOM 2595 O PHE X 168 20.274 7.354 20.972 0.00 0.00 O

ATOM 2596 N ILE X 169 20.238 8.827 19.207 0.00 0.00 N

ATOM 2597 HN ILE X 169 20.147 8.858 18.217 0.00 0.00 H

ATOM 2598 CA ILE X 169 20.210 10.019 20.082 0.00 0.00 C

ATOM 2599 HA ILE X 169 21.042 9.868 20.748 0.00 0.00 H

ATOM 2600 CB ILE X 169 20.529 11.295 19.367 0.00 0.00 C

ATOM 2601 HB ILE X 169 19.853 11.448 18.497 0.00 0.00 H

ATOM 2602 CG2 ILE X 169 20.353 12.586 20.190 0.00 0.00 C

ATOM 2603 HG21 ILE X 169 20.764 12.383 21.196 0.00 0.00 H

ATOM 2604 HG22 ILE X 169 19.288 12.877 20.336 0.00 0.00 H

ATOM 2605 HG23 ILE X 169 20.833 13.489 19.769 0.00 0.00 H

ATOM 2606 CG1 ILE X 169 21.942 11.143 18.725 0.00 0.00 C

ATOM 2607 HG11 ILE X 169 22.211 12.052 18.139 0.00 0.00 H

ATOM 2608 HG12 ILE X 169 21.975 10.365 17.933 0.00 0.00 H

ATOM 2609 CD ILE X 169 23.040 10.926 19.726 0.00 0.00 C

ATOM 2610 HD1 ILE X 169 24.053 10.992 19.258 0.00 0.00 H

ATOM 2611 HD2 ILE X 169 22.828 9.943 20.202 0.00 0.00 H

ATOM 2612 HD3 ILE X 169 22.966 11.615 20.590 0.00 0.00 H

ATOM 2613 C ILE X 169 18.965 10.127 20.998 0.00 0.00 C

ATOM 2614 O ILE X 169 17.893 10.033 20.423 0.00 0.00 O

ATOM 2615 N PRO X 170 19.003 10.287 22.312 0.00 0.00 N

ATOM 2616 CD PRO X 170 20.210 10.136 23.180 0.00 0.00 C

ATOM 2617 HD1 PRO X 170 20.991 10.895 22.973 0.00 0.00 H

ATOM 2618 HD2 PRO X 170 20.655 9.127 23.104 0.00 0.00 H

ATOM 2619 CA PRO X 170 17.789 10.181 23.114 0.00 0.00 C

ATOM 2620 HA PRO X 170 17.010 9.527 22.760 0.00 0.00 H

ATOM 2621 CB PRO X 170 18.356 9.769 24.474 0.00 0.00 C

ATOM 2622 HB1 PRO X 170 18.278 8.656 24.418 0.00 0.00 H

ATOM 2623 HB2 PRO X 170 17.695 10.004 25.333 0.00 0.00 H

ATOM 2624 CG PRO X 170 19.824 10.119 24.651 0.00 0.00 C

ATOM 2625 HG1 PRO X 170 19.856 11.116 25.142 0.00 0.00 H

ATOM 2626 HG2 PRO X 170 20.567 9.505 25.196 0.00 0.00 H

ATOM 2627 C PRO X 170 17.035 11.495 23.297 0.00 0.00 C

ATOM 2628 O PRO X 170 17.024 12.107 24.354 0.00 0.00 O

ATOM 2629 N LEU X 171 16.237 11.773 22.254 0.00 0.00 N

ATOM 2630 HN LEU X 171 16.241 11.104 21.513 0.00 0.00 H

ATOM 2631 CA LEU X 171 15.346 12.842 22.036 0.00 0.00 C

ATOM 2632 HA LEU X 171 15.899 13.768 22.125 0.00 0.00 H

ATOM 2633 CB LEU X 171 14.944 12.775 20.524 0.00 0.00 C

ATOM 2634 HB1 LEU X 171 14.096 13.446 20.276 0.00 0.00 H

ATOM 2635 HB2 LEU X 171 14.552 11.738 20.429 0.00 0.00 H

ATOM 2636 CG LEU X 171 16.090 13.041 19.462 0.00 0.00 C

ATOM 2637 HG LEU X 171 16.986 12.535 19.876 0.00 0.00 H

ATOM 2638 CD1 LEU X 171 15.817 12.506 18.057 0.00 0.00 C

ATOM 2639 HD11 LEU X 171 14.731 12.664 17.877 0.00 0.00 H

ATOM 2640 HD12 LEU X 171 15.924 11.396 18.092 0.00 0.00 H

ATOM 2641 HD13 LEU X 171 16.405 13.032 17.279 0.00 0.00 H

ATOM 2642 CD2 LEU X 171 16.634 14.480 19.366 0.00 0.00 C

ATOM 2643 HD21 LEU X 171 17.601 14.567 18.830 0.00 0.00 H

ATOM 2644 HD22 LEU X 171 16.908 14.782 20.394 0.00 0.00 H

ATOM 2645 HD23 LEU X 171 16.035 15.208 18.778 0.00 0.00 H

ATOM 2646 C LEU X 171 14.165 13.125 22.947 0.00 0.00 C

ATOM 2647 O LEU X 171 13.340 12.253 23.156 0.00 0.00 O

ATOM 2648 N GLU X 172 13.948 14.351 23.577 0.00 0.00 N

ATOM 2649 HN GLU X 172 14.639 15.060 23.444 0.00 0.00 H

ATOM 2650 CA GLU X 172 12.985 14.568 24.627 0.00 0.00 C

ATOM 2651 HA GLU X 172 13.128 13.869 25.435 0.00 0.00 H

ATOM 2652 CB GLU X 172 13.229 16.067 25.179 0.00 0.00 C

ATOM 2653 HB1 GLU X 172 12.409 16.412 25.853 0.00 0.00 H

ATOM 2654 HB2 GLU X 172 13.390 16.769 24.324 0.00 0.00 H

ATOM 2655 CG GLU X 172 14.549 16.129 25.995 0.00 0.00 C

ATOM 2656 HG1 GLU X 172 14.765 17.130 26.406 0.00 0.00 H

ATOM 2657 HG2 GLU X 172 15.360 15.667 25.388 0.00 0.00 H

ATOM 2658 CD GLU X 172 14.492 15.412 27.286 0.00 0.00 C

ATOM 2659 OE1 GLU X 172 13.422 15.251 27.956 0.00 0.00 O

ATOM 2660 OE2 GLU X 172 15.583 14.836 27.655 0.00 0.00 O1-

ATOM 2661 C GLU X 172 11.508 14.427 24.082 0.00 0.00 C

ATOM 2662 O GLU X 172 10.597 14.181 24.856 0.00 0.00 O

ATOM 2663 N SER X 173 11.256 14.548 22.767 0.00 0.00 N

ATOM 2664 HN SER X 173 11.981 14.874 22.159 0.00 0.00 H

ATOM 2665 CA SER X 173 9.957 14.208 22.294 0.00 0.00 C

ATOM 2666 HA SER X 173 9.193 14.393 23.025 0.00 0.00 H

ATOM 2667 CB SER X 173 9.408 15.120 21.045 0.00 0.00 C

ATOM 2668 HB1 SER X 173 8.378 14.809 20.792 0.00 0.00 H

ATOM 2669 HB2 SER X 173 10.129 14.883 20.232 0.00 0.00 H

ATOM 2670 OG SER X 173 9.351 16.455 21.434 0.00 0.00 O

ATOM 2671 HG1 SER X 173 10.276 16.692 21.479 0.00 0.00 H

ATOM 2672 C SER X 173 9.755 12.784 21.984 0.00 0.00 C

ATOM 2673 O SER X 173 8.630 12.460 21.832 0.00 0.00 O

ATOM 2674 N LYS X 174 10.762 11.941 22.150 0.00 0.00 N

ATOM 2675 HN LYS X 174 11.683 12.237 22.382 0.00 0.00 H

ATOM 2676 CA LYS X 174 10.688 10.476 21.977 0.00 0.00 C

ATOM 2677 HA LYS X 174 9.691 10.325 21.586 0.00 0.00 H

ATOM 2678 CB LYS X 174 11.777 9.916 21.074 0.00 0.00 C

ATOM 2679 HB1 LYS X 174 11.857 8.816 20.874 0.00 0.00 H

ATOM 2680 HB2 LYS X 174 12.756 10.047 21.586 0.00 0.00 H

ATOM 2681 CG LYS X 174 11.786 10.712 19.712 0.00 0.00 C

ATOM 2682 HG1 LYS X 174 11.964 11.789 19.902 0.00 0.00 H

ATOM 2683 HG2 LYS X 174 10.760 10.855 19.308 0.00 0.00 H

ATOM 2684 CD LYS X 174 12.770 10.170 18.575 0.00 0.00 C

ATOM 2685 HD1 LYS X 174 12.563 9.083 18.544 0.00 0.00 H

ATOM 2686 HD2 LYS X 174 13.847 10.326 18.793 0.00 0.00 H

ATOM 2687 CE LYS X 174 12.530 10.886 17.221 0.00 0.00 C

ATOM 2688 HE1 LYS X 174 13.503 10.748 16.691 0.00 0.00 H

ATOM 2689 HE2 LYS X 174 12.638 11.901 17.649 0.00 0.00 H

ATOM 2690 NZ LYS X 174 11.270 10.692 16.653 0.00 0.00 N1+

ATOM 2691 HZ1 LYS X 174 11.118 9.673 16.583 0.00 0.00 H

ATOM 2692 HZ2 LYS X 174 11.160 11.209 15.760 0.00 0.00 H

ATOM 2693 HZ3 LYS X 174 10.504 10.960 17.297 0.00 0.00 H

ATOM 2694 C LYS X 174 10.797 9.814 23.303 0.00 0.00 C

ATOM 2695 O LYS X 174 10.108 8.843 23.544 0.00 0.00 O

ATOM 2696 N LYS X 175 11.524 10.342 24.305 0.00 0.00 N

ATOM 2697 HN LYS X 175 12.275 10.979 24.153 0.00 0.00 H

ATOM 2698 CA LYS X 175 11.369 9.937 25.706 0.00 0.00 C

ATOM 2699 HA LYS X 175 11.805 8.960 25.818 0.00 0.00 H

ATOM 2700 CB LYS X 175 12.283 10.898 26.579 0.00 0.00 C

ATOM 2701 HB1 LYS X 175 11.944 10.746 27.631 0.00 0.00 H

ATOM 2702 HB2 LYS X 175 12.017 11.953 26.389 0.00 0.00 H

ATOM 2703 CG LYS X 175 13.846 10.757 26.507 0.00 0.00 C

ATOM 2704 HG1 LYS X 175 14.226 10.866 25.462 0.00 0.00 H

ATOM 2705 HG2 LYS X 175 14.201 9.843 27.019 0.00 0.00 H

ATOM 2706 CD LYS X 175 14.650 11.811 27.351 0.00 0.00 C

ATOM 2707 HD1 LYS X 175 14.473 12.706 26.718 0.00 0.00 H

ATOM 2708 HD2 LYS X 175 15.735 11.613 27.422 0.00 0.00 H

ATOM 2709 CE LYS X 175 14.068 12.019 28.766 0.00 0.00 C

ATOM 2710 HE1 LYS X 175 13.864 11.135 29.397 0.00 0.00 H

ATOM 2711 HE2 LYS X 175 13.075 12.500 28.636 0.00 0.00 H

ATOM 2712 NZ LYS X 175 14.825 12.920 29.524 0.00 0.00 N1+

ATOM 2713 HZ1 LYS X 175 15.780 12.548 29.664 0.00 0.00 H

ATOM 2714 HZ2 LYS X 175 14.308 13.062 30.416 0.00 0.00 H

ATOM 2715 HZ3 LYS X 175 14.928 13.878 29.129 0.00 0.00 H

ATOM 2716 C LYS X 175 9.941 9.567 26.240 0.00 0.00 C

ATOM 2717 O LYS X 175 9.673 8.492 26.675 0.00 0.00 O

ATOM 2718 N PRO X 176 8.909 10.461 26.310 0.00 0.00 N

ATOM 2719 CD PRO X 176 8.933 11.906 25.965 0.00 0.00 C

ATOM 2720 HD1 PRO X 176 8.711 12.104 24.889 0.00 0.00 H

ATOM 2721 HD2 PRO X 176 9.983 12.216 26.119 0.00 0.00 H

ATOM 2722 CA PRO X 176 7.620 9.990 26.675 0.00 0.00 C

ATOM 2723 HA PRO X 176 7.691 9.440 27.596 0.00 0.00 H

ATOM 2724 CB PRO X 176 6.862 11.265 26.941 0.00 0.00 C

ATOM 2725 HB1 PRO X 176 6.316 11.265 27.909 0.00 0.00 H

ATOM 2726 HB2 PRO X 176 6.151 11.448 26.105 0.00 0.00 H

ATOM 2727 CG PRO X 176 7.902 12.366 26.820 0.00 0.00 C

ATOM 2728 HG1 PRO X 176 7.451 13.377 26.774 0.00 0.00 H

ATOM 2729 HG2 PRO X 176 8.339 12.590 27.820 0.00 0.00 H

ATOM 2730 C PRO X 176 6.802 9.169 25.652 0.00 0.00 C

ATOM 2731 O PRO X 176 5.632 8.801 25.922 0.00 0.00 O

ATOM 2732 N LYS X 177 7.332 8.771 24.426 0.00 0.00 N

ATOM 2733 HN LYS X 177 8.238 9.114 24.199 0.00 0.00 H

ATOM 2734 CA LYS X 177 6.671 7.830 23.529 0.00 0.00 C

ATOM 2735 HA LYS X 177 5.618 8.032 23.683 0.00 0.00 H

ATOM 2736 CB LYS X 177 6.875 8.274 22.090 0.00 0.00 C

ATOM 2737 HB1 LYS X 177 6.308 7.565 21.450 0.00 0.00 H

ATOM 2738 HB2 LYS X 177 7.926 8.257 21.712 0.00 0.00 H

ATOM 2739 CG LYS X 177 6.264 9.616 21.612 0.00 0.00 C

ATOM 2740 HG1 LYS X 177 6.415 9.623 20.509 0.00 0.00 H

ATOM 2741 HG2 LYS X 177 6.818 10.471 22.059 0.00 0.00 H

ATOM 2742 CD LYS X 177 4.728 9.768 21.898 0.00 0.00 C

ATOM 2743 HD1 LYS X 177 4.571 9.754 22.990 0.00 0.00 H

ATOM 2744 HD2 LYS X 177 4.255 8.888 21.410 0.00 0.00 H

ATOM 2745 CE LYS X 177 4.218 11.089 21.401 0.00 0.00 C

ATOM 2746 HE1 LYS X 177 4.600 11.995 21.907 0.00 0.00 H

ATOM 2747 HE2 LYS X 177 3.127 11.219 21.566 0.00 0.00 H

ATOM 2748 NZ LYS X 177 4.514 11.341 19.965 0.00 0.00 N1+

ATOM 2749 HZ1 LYS X 177 4.015 12.199 19.652 0.00 0.00 H

ATOM 2750 HZ2 LYS X 177 4.144 10.505 19.476 0.00 0.00 H

ATOM 2751 HZ3 LYS X 177 5.545 11.404 19.822 0.00 0.00 H

ATOM 2752 C LYS X 177 7.086 6.399 23.861 0.00 0.00 C

ATOM 2753 O LYS X 177 6.863 5.421 23.125 0.00 0.00 O

ATOM 2754 N GLY X 178 7.757 6.147 25.100 0.00 0.00 N

ATOM 2755 HN GLY X 178 7.968 6.884 25.729 0.00 0.00 H

ATOM 2756 CA GLY X 178 8.170 4.799 25.517 0.00 0.00 C

ATOM 2757 HA1 GLY X 178 7.392 4.144 25.153 0.00 0.00 H

ATOM 2758 HA2 GLY X 178 8.122 4.821 26.599 0.00 0.00 H

ATOM 2759 C GLY X 178 9.437 4.418 25.055 0.00 0.00 C

ATOM 2760 O GLY X 178 9.765 3.230 25.119 0.00 0.00 O

ATOM 2761 N PHE X 179 10.299 5.382 24.632 0.00 0.00 N

ATOM 2762 HN PHE X 179 9.911 6.280 24.419 0.00 0.00 H

ATOM 2763 CA PHE X 179 11.682 5.142 24.446 0.00 0.00 C

ATOM 2764 HA PHE X 179 11.742 4.354 23.718 0.00 0.00 H

ATOM 2765 CB PHE X 179 12.255 6.351 23.692 0.00 0.00 C

ATOM 2766 HB1 PHE X 179 12.197 7.092 24.513 0.00 0.00 H

ATOM 2767 HB2 PHE X 179 11.632 6.675 22.836 0.00 0.00 H

ATOM 2768 CG PHE X 179 13.729 6.238 23.205 0.00 0.00 C

ATOM 2769 CD1 PHE X 179 14.734 6.915 23.774 0.00 0.00 C

ATOM 2770 HD1 PHE X 179 14.595 7.481 24.679 0.00 0.00 H

ATOM 2771 CE1 PHE X 179 16.045 6.807 23.319 0.00 0.00 C

ATOM 2772 HE1 PHE X 179 16.825 7.221 23.941 0.00 0.00 H

ATOM 2773 CZ PHE X 179 16.354 6.041 22.244 0.00 0.00 C

ATOM 2774 HZ PHE X 179 17.383 6.088 21.910 0.00 0.00 H

ATOM 2775 CD2 PHE X 179 13.978 5.485 21.992 0.00 0.00 C

ATOM 2776 HD2 PHE X 179 13.174 5.008 21.462 0.00 0.00 H

ATOM 2777 CE2 PHE X 179 15.302 5.339 21.551 0.00 0.00 C

ATOM 2778 HE2 PHE X 179 15.508 4.760 20.664 0.00 0.00 H

ATOM 2779 C PHE X 179 12.602 4.847 25.654 0.00 0.00 C

ATOM 2780 O PHE X 179 12.547 5.377 26.772 0.00 0.00 O

ATOM 2781 N PHE X 180 13.498 3.912 25.512 0.00 0.00 N

ATOM 2782 HN PHE X 180 13.645 3.244 24.786 0.00 0.00 H

ATOM 2783 CA PHE X 180 14.481 3.662 26.573 0.00 0.00 C

ATOM 2784 HA PHE X 180 14.518 4.370 27.387 0.00 0.00 H

ATOM 2785 CB PHE X 180 14.107 2.361 27.274 0.00 0.00 C

ATOM 2786 HB1 PHE X 180 14.218 1.493 26.588 0.00 0.00 H

ATOM 2787 HB2 PHE X 180 13.042 2.262 27.578 0.00 0.00 H

ATOM 2788 CG PHE X 180 14.914 2.093 28.438 0.00 0.00 C

ATOM 2789 CD1 PHE X 180 16.243 1.643 28.482 0.00 0.00 C

ATOM 2790 HD1 PHE X 180 16.747 1.323 27.591 0.00 0.00 H

ATOM 2791 CE1 PHE X 180 16.981 1.513 29.660 0.00 0.00 C

ATOM 2792 HE1 PHE X 180 17.994 1.149 29.583 0.00 0.00 H

ATOM 2793 CZ PHE X 180 16.339 1.795 30.852 0.00 0.00 C

ATOM 2794 HZ PHE X 180 17.022 1.689 31.689 0.00 0.00 H

ATOM 2795 CD2 PHE X 180 14.330 2.405 29.735 0.00 0.00 C

ATOM 2796 HD2 PHE X 180 13.338 2.839 29.699 0.00 0.00 H

ATOM 2797 CE2 PHE X 180 15.044 2.257 30.902 0.00 0.00 C

ATOM 2798 HE2 PHE X 180 14.463 2.498 31.770 0.00 0.00 H

ATOM 2799 C PHE X 180 15.922 3.598 26.069 0.00 0.00 C

ATOM 2800 O PHE X 180 16.839 3.977 26.830 0.00 0.00 O

ATOM 2801 N GLY X 181 16.021 3.285 24.821 0.00 0.00 N

ATOM 2802 HN GLY X 181 15.135 3.189 24.377 0.00 0.00 H

ATOM 2803 CA GLY X 181 17.174 3.162 23.977 0.00 0.00 C

ATOM 2804 HA1 GLY X 181 17.950 3.890 24.184 0.00 0.00 H

ATOM 2805 HA2 GLY X 181 16.956 3.245 22.919 0.00 0.00 H

ATOM 2806 C GLY X 181 17.834 1.811 23.977 0.00 0.00 C

ATOM 2807 O GLY X 181 17.578 0.973 24.922 0.00 0.00 O

ATOM 2808 N TYR X 182 18.571 1.532 22.957 0.00 0.00 N

ATOM 2809 HN TYR X 182 18.822 2.354 22.446 0.00 0.00 H

ATOM 2810 CA TYR X 182 18.930 0.200 22.359 0.00 0.00 C

ATOM 2811 HA TYR X 182 18.019 -0.282 22.049 0.00 0.00 H

ATOM 2812 CB TYR X 182 19.965 0.322 21.143 0.00 0.00 C

ATOM 2813 HB1 TYR X 182 20.466 -0.601 20.805 0.00 0.00 H

ATOM 2814 HB2 TYR X 182 20.769 0.974 21.540 0.00 0.00 H

ATOM 2815 CG TYR X 182 19.285 0.789 19.848 0.00 0.00 C

ATOM 2816 CD1 TYR X 182 19.726 1.946 19.265 0.00 0.00 C

ATOM 2817 HD1 TYR X 182 20.537 2.551 19.636 0.00 0.00 H

ATOM 2818 CE1 TYR X 182 19.219 2.319 17.945 0.00 0.00 C

ATOM 2819 HE1 TYR X 182 19.610 3.185 17.426 0.00 0.00 H

ATOM 2820 CZ TYR X 182 18.214 1.524 17.313 0.00 0.00 C

ATOM 2821 OH TYR X 182 17.628 1.866 16.057 0.00 0.00 O

ATOM 2822 HH TYR X 182 17.668 2.825 16.059 0.00 0.00 H

ATOM 2823 CD2 TYR X 182 18.281 -0.048 19.260 0.00 0.00 C

ATOM 2824 HD2 TYR X 182 18.006 -0.974 19.745 0.00 0.00 H

ATOM 2825 CE2 TYR X 182 17.752 0.355 17.996 0.00 0.00 C

ATOM 2826 HE2 TYR X 182 17.033 -0.227 17.432 0.00 0.00 H

ATOM 2827 C TYR X 182 19.508 -0.750 23.372 0.00 0.00 C

ATOM 2828 O TYR X 182 20.586 -0.521 23.938 0.00 0.00 O

ATOM 2829 N LYS X 183 18.750 -1.826 23.714 0.00 0.00 N

ATOM 2830 HN LYS X 183 17.846 -2.044 23.347 0.00 0.00 H

ATOM 2831 CA LYS X 183 19.152 -2.800 24.699 0.00 0.00 C

ATOM 2832 HA LYS X 183 20.181 -2.744 25.040 0.00 0.00 H

ATOM 2833 CB LYS X 183 18.123 -2.545 25.810 0.00 0.00 C

ATOM 2834 HB1 LYS X 183 17.117 -2.830 25.457 0.00 0.00 H

ATOM 2835 HB2 LYS X 183 17.955 -1.447 25.900 0.00 0.00 H

ATOM 2836 CG LYS X 183 18.489 -3.132 27.230 0.00 0.00 C

ATOM 2837 HG1 LYS X 183 19.117 -4.049 27.107 0.00 0.00 H

ATOM 2838 HG2 LYS X 183 17.446 -3.300 27.550 0.00 0.00 H

ATOM 2839 CD LYS X 183 19.154 -2.045 28.027 0.00 0.00 C

ATOM 2840 HD1 LYS X 183 18.540 -1.117 27.989 0.00 0.00 H

ATOM 2841 HD2 LYS X 183 20.183 -1.870 27.653 0.00 0.00 H

ATOM 2842 CE LYS X 183 19.220 -2.333 29.464 0.00 0.00 C

ATOM 2843 HE1 LYS X 183 18.253 -2.626 29.912 0.00 0.00 H

ATOM 2844 HE2 LYS X 183 19.756 -1.448 29.867 0.00 0.00 H

ATOM 2845 NZ LYS X 183 20.083 -3.485 29.655 0.00 0.00 N1+

ATOM 2846 HZ1 LYS X 183 19.760 -4.424 29.329 0.00 0.00 H

ATOM 2847 HZ2 LYS X 183 20.290 -3.619 30.664 0.00 0.00 H

ATOM 2848 HZ3 LYS X 183 20.927 -3.231 29.085 0.00 0.00 H

ATOM 2849 C LYS X 183 18.956 -4.233 24.208 0.00 0.00 C

ATOM 2850 O LYS X 183 18.172 -4.451 23.280 0.00 0.00 O

ATOM 2851 N SER X 184 19.615 -5.211 24.809 0.00 0.00 N

ATOM 2852 HN SER X 184 20.387 -5.106 25.431 0.00 0.00 H

ATOM 2853 CA SER X 184 19.341 -6.645 24.602 0.00 0.00 C

ATOM 2854 HA SER X 184 19.234 -6.851 23.546 0.00 0.00 H

ATOM 2855 CB SER X 184 20.418 -7.443 25.267 0.00 0.00 C

ATOM 2856 HB1 SER X 184 20.481 -7.227 26.348 0.00 0.00 H

ATOM 2857 HB2 SER X 184 21.371 -7.060 24.841 0.00 0.00 H

ATOM 2858 OG SER X 184 20.238 -8.905 25.021 0.00 0.00 O

ATOM 2859 HG1 SER X 184 19.893 -9.328 25.808 0.00 0.00 H

ATOM 2860 C SER X 184 18.008 -7.022 25.238 0.00 0.00 C

ATOM 2861 O SER X 184 17.196 -7.727 24.591 0.00 0.00 O

ATOM 2862 N GLU X 185 17.729 -6.617 26.441 0.00 0.00 N

ATOM 2863 HN GLU X 185 18.361 -6.145 27.063 0.00 0.00 H

ATOM 2864 CA GLU X 185 16.433 -6.954 27.017 0.00 0.00 C

ATOM 2865 HA GLU X 185 16.445 -8.025 27.180 0.00 0.00 H

ATOM 2866 CB GLU X 185 16.400 -6.322 28.413 0.00 0.00 C

ATOM 2867 HB1 GLU X 185 15.387 -6.404 28.853 0.00 0.00 H

ATOM 2868 HB2 GLU X 185 16.600 -5.227 28.453 0.00 0.00 H

ATOM 2869 CG GLU X 185 17.311 -7.123 29.423 0.00 0.00 C

ATOM 2870 HG1 GLU X 185 17.233 -8.230 29.316 0.00 0.00 H

ATOM 2871 HG2 GLU X 185 17.014 -6.980 30.480 0.00 0.00 H

ATOM 2872 CD GLU X 185 18.804 -6.653 29.464 0.00 0.00 C

ATOM 2873 OE1 GLU X 185 19.309 -5.775 28.745 0.00 0.00 O

ATOM 2874 OE2 GLU X 185 19.308 -7.191 30.492 0.00 0.00 O1-

ATOM 2875 C GLU X 185 15.156 -6.650 26.192 0.00 0.00 C

ATOM 2876 O GLU X 185 14.283 -7.456 26.070 0.00 0.00 O

ATOM 2877 N ILE X 186 15.087 -5.457 25.622 0.00 0.00 N

ATOM 2878 HN ILE X 186 15.865 -4.849 25.737 0.00 0.00 H

ATOM 2879 CA ILE X 186 13.890 -4.914 25.001 0.00 0.00 C

ATOM 2880 HA ILE X 186 13.062 -4.971 25.691 0.00 0.00 H

ATOM 2881 CB ILE X 186 14.117 -3.451 24.687 0.00 0.00 C

ATOM 2882 HB ILE X 186 14.964 -3.261 23.991 0.00 0.00 H

ATOM 2883 CG2 ILE X 186 12.899 -2.964 23.846 0.00 0.00 C

ATOM 2884 HG21 ILE X 186 11.878 -3.103 24.268 0.00 0.00 H

ATOM 2885 HG22 ILE X 186 12.936 -3.386 22.820 0.00 0.00 H

ATOM 2886 HG23 ILE X 186 13.037 -1.882 23.658 0.00 0.00 H

ATOM 2887 CG1 ILE X 186 14.077 -2.584 25.976 0.00 0.00 C

ATOM 2888 HG11 ILE X 186 14.814 -2.957 26.716 0.00 0.00 H

ATOM 2889 HG12 ILE X 186 13.069 -2.694 26.448 0.00 0.00 H

ATOM 2890 CD ILE X 186 14.398 -1.168 25.826 0.00 0.00 C

ATOM 2891 HD1 ILE X 186 13.675 -0.545 25.252 0.00 0.00 H

ATOM 2892 HD2 ILE X 186 15.356 -1.102 25.271 0.00 0.00 H

ATOM 2893 HD3 ILE X 186 14.581 -0.635 26.779 0.00 0.00 H

ATOM 2894 C ILE X 186 13.544 -5.680 23.764 0.00 0.00 C

ATOM 2895 O ILE X 186 12.456 -6.082 23.481 0.00 0.00 O

ATOM 2896 N PHE X 187 14.542 -6.034 22.868 0.00 0.00 N

ATOM 2897 HN PHE X 187 15.444 -5.696 23.146 0.00 0.00 H

ATOM 2898 CA PHE X 187 14.415 -6.959 21.787 0.00 0.00 C

ATOM 2899 HA PHE X 187 13.644 -6.463 21.226 0.00 0.00 H

ATOM 2900 CB PHE X 187 15.789 -7.161 21.144 0.00 0.00 C

ATOM 2901 HB1 PHE X 187 16.510 -7.339 21.970 0.00 0.00 H

ATOM 2902 HB2 PHE X 187 16.043 -6.186 20.673 0.00 0.00 H

ATOM 2903 CG PHE X 187 15.998 -8.173 20.085 0.00 0.00 C

ATOM 2904 CD1 PHE X 187 16.233 -9.534 20.402 0.00 0.00 C

ATOM 2905 HD1 PHE X 187 16.473 -9.701 21.437 0.00 0.00 H

ATOM 2906 CE1 PHE X 187 16.292 -10.528 19.406 0.00 0.00 C

ATOM 2907 HE1 PHE X 187 16.415 -11.573 19.660 0.00 0.00 H

ATOM 2908 CZ PHE X 187 16.193 -10.133 18.109 0.00 0.00 C

ATOM 2909 HZ PHE X 187 16.449 -10.909 17.402 0.00 0.00 H

ATOM 2910 CD2 PHE X 187 15.853 -7.886 18.759 0.00 0.00 C

ATOM 2911 HD2 PHE X 187 15.556 -6.928 18.371 0.00 0.00 H

ATOM 2912 CE2 PHE X 187 16.107 -8.790 17.738 0.00 0.00 C

ATOM 2913 HE2 PHE X 187 16.083 -8.427 16.723 0.00 0.00 H

ATOM 2914 C PHE X 187 13.860 -8.299 22.128 0.00 0.00 C

ATOM 2915 O PHE X 187 13.013 -8.890 21.419 0.00 0.00 O

ATOM 2916 N ASN X 188 14.230 -8.950 23.240 0.00 0.00 N

ATOM 2917 HN ASN X 188 14.919 -8.485 23.798 0.00 0.00 H

ATOM 2918 CA ASN X 188 13.734 -10.240 23.654 0.00 0.00 C

ATOM 2919 HA ASN X 188 13.633 -10.942 22.833 0.00 0.00 H

ATOM 2920 CB ASN X 188 14.707 -10.865 24.669 0.00 0.00 C

ATOM 2921 HB1 ASN X 188 14.207 -11.773 25.068 0.00 0.00 H

ATOM 2922 HB2 ASN X 188 14.956 -10.185 25.498 0.00 0.00 H

ATOM 2923 CG ASN X 188 16.072 -11.328 24.103 0.00 0.00 C

ATOM 2924 OD1 ASN X 188 16.186 -12.484 23.778 0.00 0.00 O

ATOM 2925 ND2 ASN X 188 17.054 -10.446 24.000 0.00 0.00 N

ATOM 2926 HD21 ASN X 188 16.959 -9.491 24.269 0.00 0.00 H

ATOM 2927 HD22 ASN X 188 17.921 -10.723 23.589 0.00 0.00 H

ATOM 2928 C ASN X 188 12.337 -10.032 24.194 0.00 0.00 C

ATOM 2929 O ASN X 188 11.507 -10.944 24.144 0.00 0.00 O

ATOM 2930 N GLU X 189 12.054 -8.887 24.823 0.00 0.00 N

ATOM 2931 HN GLU X 189 12.708 -8.177 25.049 0.00 0.00 H

ATOM 2932 CA GLU X 189 10.673 -8.621 25.269 0.00 0.00 C

ATOM 2933 HA GLU X 189 10.243 -9.464 25.793 0.00 0.00 H

ATOM 2934 CB GLU X 189 10.546 -7.516 26.341 0.00 0.00 C

ATOM 2935 HB1 GLU X 189 9.441 -7.357 26.432 0.00 0.00 H

ATOM 2936 HB2 GLU X 189 11.007 -6.544 26.065 0.00 0.00 H

ATOM 2937 CG GLU X 189 11.001 -8.117 27.661 0.00 0.00 C

ATOM 2938 HG1 GLU X 189 12.082 -8.325 27.510 0.00 0.00 H

ATOM 2939 HG2 GLU X 189 10.511 -9.109 27.845 0.00 0.00 H

ATOM 2940 CD GLU X 189 10.811 -7.136 28.755 0.00 0.00 C

ATOM 2941 OE1 GLU X 189 9.692 -6.982 29.308 0.00 0.00 O

ATOM 2942 OE2 GLU X 189 11.658 -6.242 29.120 0.00 0.00 O1-

ATOM 2943 C GLU X 189 9.723 -8.499 24.089 0.00 0.00 C

ATOM 2944 O GLU X 189 8.610 -9.091 24.128 0.00 0.00 O

ATOM 2945 N ASN X 190 10.147 -7.813 23.072 0.00 0.00 N

ATOM 2946 HN ASN X 190 11.101 -7.547 23.108 0.00 0.00 H

ATOM 2947 CA ASN X 190 9.341 -7.582 21.900 0.00 0.00 C

ATOM 2948 HA ASN X 190 8.291 -7.479 22.111 0.00 0.00 H

ATOM 2949 CB ASN X 190 9.776 -6.223 21.323 0.00 0.00 C

ATOM 2950 HB1 ASN X 190 9.105 -5.954 20.475 0.00 0.00 H

ATOM 2951 HB2 ASN X 190 10.840 -6.123 21.029 0.00 0.00 H

ATOM 2952 CG ASN X 190 9.446 -5.218 22.470 0.00 0.00 C

ATOM 2953 OD1 ASN X 190 8.912 -5.461 23.547 0.00 0.00 O

ATOM 2954 ND2 ASN X 190 9.805 -3.963 22.113 0.00 0.00 N

ATOM 2955 HD21 ASN X 190 9.545 -3.124 22.604 0.00 0.00 H

ATOM 2956 HD22 ASN X 190 10.539 -3.966 21.447 0.00 0.00 H

ATOM 2957 C ASN X 190 9.341 -8.687 20.915 0.00 0.00 C

ATOM 2958 O ASN X 190 8.496 -8.752 20.070 0.00 0.00 O

ATOM 2959 N PHE X 191 10.147 -9.714 21.071 0.00 0.00 N

ATOM 2960 HN PHE X 191 10.835 -9.747 21.793 0.00 0.00 H

ATOM 2961 CA PHE X 191 10.126 -10.945 20.233 0.00 0.00 C

ATOM 2962 HA PHE X 191 10.260 -10.686 19.192 0.00 0.00 H

ATOM 2963 CB PHE X 191 11.229 -11.933 20.802 0.00 0.00 C

ATOM 2964 HB1 PHE X 191 10.967 -12.423 21.763 0.00 0.00 H

ATOM 2965 HB2 PHE X 191 12.085 -11.342 21.209 0.00 0.00 H

ATOM 2966 CG PHE X 191 11.772 -12.904 19.746 0.00 0.00 C

ATOM 2967 CD1 PHE X 191 13.151 -12.911 19.433 0.00 0.00 C

ATOM 2968 HD1 PHE X 191 13.724 -12.078 19.803 0.00 0.00 H

ATOM 2969 CE1 PHE X 191 13.642 -13.877 18.573 0.00 0.00 C

ATOM 2970 HE1 PHE X 191 14.706 -13.885 18.458 0.00 0.00 H

ATOM 2971 CZ PHE X 191 12.814 -14.926 18.048 0.00 0.00 C

ATOM 2972 HZ PHE X 191 13.143 -15.727 17.411 0.00 0.00 H

ATOM 2973 CD2 PHE X 191 10.938 -13.913 19.278 0.00 0.00 C

ATOM 2974 HD2 PHE X 191 9.879 -13.976 19.469 0.00 0.00 H

ATOM 2975 CE2 PHE X 191 11.411 -14.937 18.349 0.00 0.00 C

ATOM 2976 HE2 PHE X 191 10.810 -15.790 18.058 0.00 0.00 H

ATOM 2977 C PHE X 191 8.806 -11.635 20.386 0.00 0.00 C

ATOM 2978 O PHE X 191 8.234 -11.938 19.306 0.00 0.00 O

ATOM 2979 N GLY X 192 8.290 -12.041 21.597 0.00 0.00 N

ATOM 2980 HN GLY X 192 8.815 -11.842 22.427 0.00 0.00 H

ATOM 2981 CA GLY X 192 6.894 -12.632 21.696 0.00 0.00 C

ATOM 2982 HA1 GLY X 192 6.587 -12.382 22.704 0.00 0.00 H

ATOM 2983 HA2 GLY X 192 7.043 -13.669 21.448 0.00 0.00 H

ATOM 2984 C GLY X 192 5.796 -12.039 20.871 0.00 0.00 C

ATOM 2985 O GLY X 192 5.689 -10.838 20.800 0.00 0.00 O

ATOM 2986 N PRO X 193 4.868 -12.729 20.191 0.00 0.00 N

ATOM 2987 CD PRO X 193 4.920 -14.117 20.057 0.00 0.00 C

ATOM 2988 HD1 PRO X 193 4.970 -14.572 21.070 0.00 0.00 H

ATOM 2989 HD2 PRO X 193 5.737 -14.326 19.333 0.00 0.00 H

ATOM 2990 CA PRO X 193 3.631 -12.113 19.591 0.00 0.00 C

ATOM 2991 HA PRO X 193 3.926 -11.213 19.080 0.00 0.00 H

ATOM 2992 CB PRO X 193 3.020 -13.302 18.676 0.00 0.00 C

ATOM 2993 HB1 PRO X 193 3.531 -13.268 17.691 0.00 0.00 H

ATOM 2994 HB2 PRO X 193 1.919 -13.270 18.534 0.00 0.00 H

ATOM 2995 CG PRO X 193 3.562 -14.588 19.446 0.00 0.00 C

ATOM 2996 HG1 PRO X 193 2.887 -14.762 20.301 0.00 0.00 H

ATOM 2997 HG2 PRO X 193 3.541 -15.581 18.935 0.00 0.00 H

ATOM 2998 C PRO X 193 2.715 -11.691 20.754 0.00 0.00 C

ATOM 2999 O PRO X 193 2.754 -12.279 21.825 0.00 0.00 O

ATOM 3000 N ASP X 194 1.991 -10.639 20.575 0.00 0.00 N

ATOM 3001 HN ASP X 194 1.892 -10.315 19.635 0.00 0.00 H

ATOM 3002 CA ASP X 194 1.155 -9.950 21.446 0.00 0.00 C

ATOM 3003 HA ASP X 194 1.407 -10.309 22.435 0.00 0.00 H

ATOM 3004 CB ASP X 194 1.347 -8.383 21.343 0.00 0.00 C

ATOM 3005 HB1 ASP X 194 0.585 -7.959 20.657 0.00 0.00 H

ATOM 3006 HB2 ASP X 194 2.406 -8.188 21.062 0.00 0.00 H

ATOM 3007 CG ASP X 194 1.200 -7.682 22.651 0.00 0.00 C

ATOM 3008 OD1 ASP X 194 0.675 -8.357 23.626 0.00 0.00 O

ATOM 3009 OD2 ASP X 194 1.628 -6.505 22.791 0.00 0.00 O1-

ATOM 3010 C ASP X 194 -0.270 -10.280 21.121 0.00 0.00 C

ATOM 3011 O ASP X 194 -1.098 -10.829 21.924 0.00 0.00 O

ATOM 3012 N PHE X 195 -0.700 -9.938 19.902 0.00 0.00 N

ATOM 3013 HN PHE X 195 -0.037 -9.639 19.218 0.00 0.00 H

ATOM 3014 CA PHE X 195 -2.010 -10.123 19.287 0.00 0.00 C

ATOM 3015 HA PHE X 195 -2.126 -9.445 18.453 0.00 0.00 H

ATOM 3016 CB PHE X 195 -2.066 -11.524 18.709 0.00 0.00 C

ATOM 3017 HB1 PHE X 195 -3.079 -11.835 18.377 0.00 0.00 H

ATOM 3018 HB2 PHE X 195 -1.828 -12.123 19.611 0.00 0.00 H

ATOM 3019 CG PHE X 195 -1.142 -11.863 17.491 0.00 0.00 C

ATOM 3020 CD1 PHE X 195 -1.118 -11.157 16.278 0.00 0.00 C

ATOM 3021 HD1 PHE X 195 -1.673 -10.231 16.287 0.00 0.00 H

ATOM 3022 CE1 PHE X 195 -0.452 -11.617 15.080 0.00 0.00 C

ATOM 3023 HE1 PHE X 195 -0.542 -10.974 14.221 0.00 0.00 H

ATOM 3024 CZ PHE X 195 0.062 -12.947 15.063 0.00 0.00 C

ATOM 3025 HZ PHE X 195 0.321 -13.345 14.090 0.00 0.00 H

ATOM 3026 CD2 PHE X 195 -0.659 -13.124 17.416 0.00 0.00 C

ATOM 3027 HD2 PHE X 195 -0.826 -13.679 18.322 0.00 0.00 H

ATOM 3028 CE2 PHE X 195 0.004 -13.660 16.304 0.00 0.00 C

ATOM 3029 HE2 PHE X 195 0.301 -14.702 16.265 0.00 0.00 H

ATOM 3030 C PHE X 195 -3.292 -9.686 20.137 0.00 0.00 C

ATOM 3031 O PHE X 195 -4.282 -10.368 20.336 0.00 0.00 O

ATOM 3032 N ARG X 196 -3.222 -8.479 20.649 0.00 0.00 N

ATOM 3033 HN ARG X 196 -2.506 -7.832 20.382 0.00 0.00 H

ATOM 3034 CA ARG X 196 -4.160 -7.865 21.645 0.00 0.00 C

ATOM 3035 HA ARG X 196 -4.252 -8.457 22.531 0.00 0.00 H

ATOM 3036 CB ARG X 196 -3.631 -6.421 21.919 0.00 0.00 C

ATOM 3037 HB1 ARG X 196 -4.403 -5.929 22.548 0.00 0.00 H

ATOM 3038 HB2 ARG X 196 -3.533 -5.952 20.911 0.00 0.00 H

ATOM 3039 CG ARG X 196 -2.219 -6.361 22.624 0.00 0.00 C

ATOM 3040 HG1 ARG X 196 -1.394 -6.564 21.901 0.00 0.00 H

ATOM 3041 HG2 ARG X 196 -2.239 -7.220 23.334 0.00 0.00 H

ATOM 3042 CD ARG X 196 -1.950 -5.131 23.388 0.00 0.00 C

ATOM 3043 HD1 ARG X 196 -2.609 -5.011 24.276 0.00 0.00 H

ATOM 3044 HD2 ARG X 196 -2.086 -4.322 22.634 0.00 0.00 H

ATOM 3045 NE ARG X 196 -0.489 -5.125 23.729 0.00 0.00 N

ATOM 3046 HE ARG X 196 0.129 -5.787 23.316 0.00 0.00 H

ATOM 3047 CZ ARG X 196 -0.015 -4.362 24.677 0.00 0.00 C

ATOM 3048 NH1 ARG X 196 -0.659 -3.479 25.415 0.00 0.00 N1+

ATOM 3049 HH11 ARG X 196 -1.551 -3.247 25.010 0.00 0.00 H

ATOM 3050 HH12 ARG X 196 -0.149 -2.840 25.993 0.00 0.00 H

ATOM 3051 NH2 ARG X 196 1.242 -4.533 24.987 0.00 0.00 N

ATOM 3052 HH21 ARG X 196 1.672 -5.243 24.436 0.00 0.00 H

ATOM 3053 HH22 ARG X 196 1.623 -4.196 25.843 0.00 0.00 H

ATOM 3054 C ARG X 196 -5.588 -7.825 21.043 0.00 0.00 C

ATOM 3055 O ARG X 196 -5.751 -7.506 19.855 0.00 0.00 O

ATOM 3056 N GLU X 197 -6.566 -8.171 21.856 0.00 0.00 N

ATOM 3057 HN GLU X 197 -6.414 -8.521 22.783 0.00 0.00 H

ATOM 3058 CA GLU X 197 -7.999 -8.321 21.468 0.00 0.00 C

ATOM 3059 HA GLU X 197 -8.426 -8.774 22.349 0.00 0.00 H

ATOM 3060 CB GLU X 197 -8.752 -7.053 21.150 0.00 0.00 C

ATOM 3061 HB1 GLU X 197 -9.774 -7.292 20.762 0.00 0.00 H

ATOM 3062 HB2 GLU X 197 -8.168 -6.503 20.391 0.00 0.00 H

ATOM 3063 CG GLU X 197 -8.942 -6.230 22.406 0.00 0.00 C

ATOM 3064 HG1 GLU X 197 -7.958 -5.994 22.860 0.00 0.00 H

ATOM 3065 HG2 GLU X 197 -9.577 -6.756 23.148 0.00 0.00 H

ATOM 3066 CD GLU X 197 -9.582 -4.990 21.951 0.00 0.00 C

ATOM 3067 OE1 GLU X 197 -8.983 -4.279 21.143 0.00 0.00 O

ATOM 3068 OE2 GLU X 197 -10.687 -4.710 22.477 0.00 0.00 O1-

ATOM 3069 C GLU X 197 -8.354 -9.426 20.363 0.00 0.00 C

ATOM 3070 O GLU X 197 -9.188 -9.343 19.480 0.00 0.00 O

ATOM 3071 N GLU X 198 -7.720 -10.561 20.558 0.00 0.00 N

ATOM 3072 HN GLU X 198 -7.253 -10.514 21.443 0.00 0.00 H

ATOM 3073 CA GLU X 198 -7.619 -11.835 19.797 0.00 0.00 C

ATOM 3074 HA GLU X 198 -6.796 -12.263 20.339 0.00 0.00 H

ATOM 3075 CB GLU X 198 -8.915 -12.590 20.059 0.00 0.00 C

ATOM 3076 HB1 GLU X 198 -9.033 -13.359 19.270 0.00 0.00 H

ATOM 3077 HB2 GLU X 198 -9.865 -12.021 20.008 0.00 0.00 H

ATOM 3078 CG GLU X 198 -8.858 -13.346 21.486 0.00 0.00 C

ATOM 3079 HG1 GLU X 198 -9.831 -13.884 21.415 0.00 0.00 H

ATOM 3080 HG2 GLU X 198 -8.737 -12.639 22.329 0.00 0.00 H

ATOM 3081 CD GLU X 198 -7.888 -14.419 21.643 0.00 0.00 C

ATOM 3082 OE1 GLU X 198 -7.529 -15.029 20.583 0.00 0.00 O

ATOM 3083 OE2 GLU X 198 -7.263 -14.604 22.711 0.00 0.00 O1-

ATOM 3084 C GLU X 198 -7.304 -11.676 18.298 0.00 0.00 C

ATOM 3085 O GLU X 198 -8.070 -12.125 17.438 0.00 0.00 O

ATOM 3086 N GLU X 199 -6.077 -11.206 17.958 0.00 0.00 N

ATOM 3087 HN GLU X 199 -5.428 -10.872 18.641 0.00 0.00 H

ATOM 3088 CA GLU X 199 -5.653 -11.093 16.565 0.00 0.00 C

ATOM 3089 HA GLU X 199 -6.529 -10.954 15.946 0.00 0.00 H

ATOM 3090 CB GLU X 199 -4.830 -9.767 16.325 0.00 0.00 C

ATOM 3091 HB1 GLU X 199 -4.366 -9.767 15.317 0.00 0.00 H

ATOM 3092 HB2 GLU X 199 -4.071 -9.659 17.116 0.00 0.00 H

ATOM 3093 CG GLU X 199 -5.633 -8.386 16.357 0.00 0.00 C

ATOM 3094 HG1 GLU X 199 -4.999 -7.475 16.295 0.00 0.00 H

ATOM 3095 HG2 GLU X 199 -6.126 -8.359 17.350 0.00 0.00 H

ATOM 3096 CD GLU X 199 -6.828 -8.329 15.366 0.00 0.00 C

ATOM 3097 OE1 GLU X 199 -7.771 -7.572 15.676 0.00 0.00 O

ATOM 3098 OE2 GLU X 199 -6.751 -8.907 14.233 0.00 0.00 O1-

ATOM 3099 C GLU X 199 -4.890 -12.371 16.078 0.00 0.00 C

ATOM 3100 O GLU X 199 -4.877 -13.357 16.840 0.00 0.00 O

ATOM 3101 N THR X 200 -4.344 -12.311 14.843 0.00 0.00 N

ATOM 3102 HN THR X 200 -4.349 -11.436 14.371 0.00 0.00 H

ATOM 3103 CA THR X 200 -3.838 -13.506 14.090 0.00 0.00 C

ATOM 3104 HA THR X 200 -3.261 -14.103 14.778 0.00 0.00 H

ATOM 3105 CB THR X 200 -5.049 -14.323 13.508 0.00 0.00 C

ATOM 3106 HB THR X 200 -5.689 -14.568 14.382 0.00 0.00 H

ATOM 3107 OG1 THR X 200 -4.629 -15.536 12.778 0.00 0.00 O

ATOM 3108 HG1 THR X 200 -5.413 -16.094 12.790 0.00 0.00 H

ATOM 3109 CG2 THR X 200 -5.857 -13.494 12.468 0.00 0.00 C

ATOM 3110 HG21 THR X 200 -6.316 -12.610 12.934 0.00 0.00 H

ATOM 3111 HG22 THR X 200 -6.644 -14.191 12.106 0.00 0.00 H

ATOM 3112 HG23 THR X 200 -5.283 -13.289 11.539 0.00 0.00 H

ATOM 3113 C THR X 200 -2.876 -13.038 13.015 0.00 0.00 C

ATOM 3114 O THR X 200 -2.789 -11.835 12.723 0.00 0.00 O

ATOM 3115 N PHE X 201 -2.180 -13.996 12.449 0.00 0.00 N

ATOM 3116 HN PHE X 201 -2.190 -14.899 12.881 0.00 0.00 H

ATOM 3117 CA PHE X 201 -1.200 -13.775 11.422 0.00 0.00 C

ATOM 3118 HA PHE X 201 -0.487 -13.057 11.775 0.00 0.00 H

ATOM 3119 CB PHE X 201 -0.501 -15.180 11.198 0.00 0.00 C

ATOM 3120 HB1 PHE X 201 -1.261 -15.978 11.108 0.00 0.00 H

ATOM 3121 HB2 PHE X 201 0.106 -15.412 12.097 0.00 0.00 H

ATOM 3122 CG PHE X 201 0.452 -15.263 10.028 0.00 0.00 C

ATOM 3123 CD1 PHE X 201 -0.065 -15.413 8.741 0.00 0.00 C

ATOM 3124 HD1 PHE X 201 -1.135 -15.465 8.628 0.00 0.00 H

ATOM 3125 CE1 PHE X 201 0.761 -15.418 7.612 0.00 0.00 C

ATOM 3126 HE1 PHE X 201 0.235 -15.536 6.675 0.00 0.00 H

ATOM 3127 CZ PHE X 201 2.107 -15.168 7.732 0.00 0.00 C

ATOM 3128 HZ PHE X 201 2.888 -15.198 6.993 0.00 0.00 H

ATOM 3129 CD2 PHE X 201 1.811 -15.139 10.147 0.00 0.00 C

ATOM 3130 HD2 PHE X 201 2.205 -15.136 11.148 0.00 0.00 H

ATOM 3131 CE2 PHE X 201 2.626 -15.041 9.030 0.00 0.00 C

ATOM 3132 HE2 PHE X 201 3.698 -14.918 9.119 0.00 0.00 H

ATOM 3133 C PHE X 201 -1.730 -13.172 10.125 0.00 0.00 C

ATOM 3134 O PHE X 201 -1.092 -12.483 9.322 0.00 0.00 O

ATOM 3135 N PHE X 202 -2.972 -13.533 9.922 0.00 0.00 N

ATOM 3136 HN PHE X 202 -3.325 -14.116 10.648 0.00 0.00 H

ATOM 3137 CA PHE X 202 -3.667 -13.298 8.681 0.00 0.00 C

ATOM 3138 HA PHE X 202 -3.037 -13.433 7.822 0.00 0.00 H

ATOM 3139 CB PHE X 202 -4.861 -14.277 8.586 0.00 0.00 C

ATOM 3140 HB1 PHE X 202 -5.373 -14.037 7.637 0.00 0.00 H

ATOM 3141 HB2 PHE X 202 -5.498 -14.334 9.498 0.00 0.00 H

ATOM 3142 CG PHE X 202 -4.073 -15.630 8.308 0.00 0.00 C

ATOM 3143 CD1 PHE X 202 -3.689 -15.895 6.997 0.00 0.00 C

ATOM 3144 HD1 PHE X 202 -3.800 -15.172 6.201 0.00 0.00 H

ATOM 3145 CE1 PHE X 202 -2.978 -17.096 6.700 0.00 0.00 C

ATOM 3146 HE1 PHE X 202 -2.710 -17.361 5.688 0.00 0.00 H

ATOM 3147 CZ PHE X 202 -2.911 -18.055 7.658 0.00 0.00 C

ATOM 3148 HZ PHE X 202 -2.430 -19.003 7.502 0.00 0.00 H

ATOM 3149 CD2 PHE X 202 -3.762 -16.491 9.327 0.00 0.00 C

ATOM 3150 HD2 PHE X 202 -3.952 -16.255 10.369 0.00 0.00 H

ATOM 3151 CE2 PHE X 202 -3.172 -17.742 8.990 0.00 0.00 C

ATOM 3152 HE2 PHE X 202 -3.017 -18.392 9.837 0.00 0.00 H

ATOM 3153 C PHE X 202 -4.162 -11.918 8.627 0.00 0.00 C

ATOM 3154 O PHE X 202 -4.201 -11.242 7.579 0.00 0.00 O

ATOM 3155 N SER X 203 -4.763 -11.423 9.758 0.00 0.00 N

ATOM 3156 HN SER X 203 -4.927 -11.922 10.610 0.00 0.00 H

ATOM 3157 CA SER X 203 -5.243 -10.004 9.823 0.00 0.00 C

ATOM 3158 HA SER X 203 -5.805 -9.751 8.939 0.00 0.00 H

ATOM 3159 CB SER X 203 -6.062 -9.795 11.162 0.00 0.00 C

ATOM 3160 HB1 SER X 203 -7.001 -10.394 11.190 0.00 0.00 H

ATOM 3161 HB2 SER X 203 -6.255 -8.704 11.139 0.00 0.00 H

ATOM 3162 OG SER X 203 -5.353 -10.115 12.329 0.00 0.00 O

ATOM 3163 HG1 SER X 203 -5.781 -9.658 13.058 0.00 0.00 H

ATOM 3164 C SER X 203 -4.076 -8.960 9.639 0.00 0.00 C

ATOM 3165 O SER X 203 -4.215 -7.948 8.881 0.00 0.00 O

ATOM 3166 N VAL X 204 -2.898 -9.244 10.301 0.00 0.00 N

ATOM 3167 HN VAL X 204 -2.795 -10.009 10.917 0.00 0.00 H

ATOM 3168 CA VAL X 204 -1.648 -8.422 10.211 0.00 0.00 C

ATOM 3169 HA VAL X 204 -1.993 -7.408 10.368 0.00 0.00 H

ATOM 3170 CB VAL X 204 -0.617 -8.744 11.303 0.00 0.00 C

ATOM 3171 HB VAL X 204 0.150 -7.956 11.203 0.00 0.00 H

ATOM 3172 CG1 VAL X 204 -1.121 -8.564 12.748 0.00 0.00 C

ATOM 3173 HG11 VAL X 204 -1.422 -7.499 12.796 0.00 0.00 H

ATOM 3174 HG12 VAL X 204 -0.336 -8.744 13.521 0.00 0.00 H

ATOM 3175 HG13 VAL X 204 -1.961 -9.229 13.043 0.00 0.00 H

ATOM 3176 CG2 VAL X 204 -0.124 -10.125 11.015 0.00 0.00 C

ATOM 3177 HG21 VAL X 204 -0.912 -10.886 11.151 0.00 0.00 H

ATOM 3178 HG22 VAL X 204 0.693 -10.261 11.768 0.00 0.00 H

ATOM 3179 HG23 VAL X 204 0.416 -10.261 10.055 0.00 0.00 H

ATOM 3180 C VAL X 204 -1.096 -8.545 8.767 0.00 0.00 C

ATOM 3181 O VAL X 204 -0.601 -7.610 8.155 0.00 0.00 O

ATOM 3182 N PHE X 205 -1.173 -9.836 8.119 0.00 0.00 N

ATOM 3183 HN PHE X 205 -1.422 -10.613 8.688 0.00 0.00 H

ATOM 3184 CA PHE X 205 -0.732 -10.000 6.746 0.00 0.00 C

ATOM 3185 HA PHE X 205 0.222 -9.492 6.686 0.00 0.00 H

ATOM 3186 CB PHE X 205 -0.438 -11.499 6.475 0.00 0.00 C

ATOM 3187 HB1 PHE X 205 -1.279 -12.116 6.857 0.00 0.00 H

ATOM 3188 HB2 PHE X 205 0.394 -11.912 7.087 0.00 0.00 H

ATOM 3189 CG PHE X 205 -0.138 -11.825 5.037 0.00 0.00 C

ATOM 3190 CD1 PHE X 205 1.216 -11.793 4.517 0.00 0.00 C

ATOM 3191 HD1 PHE X 205 2.016 -11.539 5.203 0.00 0.00 H

ATOM 3192 CE1 PHE X 205 1.478 -11.928 3.164 0.00 0.00 C

ATOM 3193 HE1 PHE X 205 2.483 -11.892 2.777 0.00 0.00 H

ATOM 3194 CZ PHE X 205 0.414 -12.125 2.244 0.00 0.00 C

ATOM 3195 HZ PHE X 205 0.732 -12.105 1.209 0.00 0.00 H

ATOM 3196 CD2 PHE X 205 -1.108 -12.138 4.055 0.00 0.00 C

ATOM 3197 HD2 PHE X 205 -2.154 -12.258 4.291 0.00 0.00 H

ATOM 3198 CE2 PHE X 205 -0.911 -12.210 2.680 0.00 0.00 C

ATOM 3199 HE2 PHE X 205 -1.735 -12.393 2.007 0.00 0.00 H

ATOM 3200 C PHE X 205 -1.713 -9.268 5.835 0.00 0.00 C

ATOM 3201 O PHE X 205 -1.255 -8.555 4.940 0.00 0.00 O

ATOM 3202 N ALA X 206 -3.042 -9.284 6.003 0.00 0.00 N

ATOM 3203 HN ALA X 206 -3.352 -9.928 6.688 0.00 0.00 H

ATOM 3204 CA ALA X 206 -4.042 -8.660 5.197 0.00 0.00 C

ATOM 3205 HA ALA X 206 -3.930 -9.051 4.197 0.00 0.00 H

ATOM 3206 CB ALA X 206 -5.438 -9.075 5.516 0.00 0.00 C

ATOM 3207 HB1 ALA X 206 -5.597 -10.167 5.390 0.00 0.00 H

ATOM 3208 HB2 ALA X 206 -6.195 -8.540 4.904 0.00 0.00 H

ATOM 3209 HB3 ALA X 206 -5.432 -8.718 6.572 0.00 0.00 H

ATOM 3210 C ALA X 206 -3.942 -7.117 5.287 0.00 0.00 C

ATOM 3211 O ALA X 206 -3.965 -6.394 4.239 0.00 0.00 O

ATOM 3212 N ILE X 207 -3.710 -6.482 6.470 0.00 0.00 N

ATOM 3213 HN ILE X 207 -3.742 -7.083 7.263 0.00 0.00 H

ATOM 3214 CA ILE X 207 -3.402 -5.042 6.650 0.00 0.00 C

ATOM 3215 HA ILE X 207 -4.168 -4.486 6.136 0.00 0.00 H

ATOM 3216 CB ILE X 207 -3.537 -4.588 8.117 0.00 0.00 C

ATOM 3217 HB ILE X 207 -2.749 -5.079 8.730 0.00 0.00 H

ATOM 3218 CG2 ILE X 207 -3.336 -3.036 8.218 0.00 0.00 C

ATOM 3219 HG21 ILE X 207 -2.340 -2.739 7.843 0.00 0.00 H

ATOM 3220 HG22 ILE X 207 -3.480 -2.708 9.269 0.00 0.00 H

ATOM 3221 HG23 ILE X 207 -4.051 -2.557 7.523 0.00 0.00 H

ATOM 3222 CG1 ILE X 207 -4.982 -4.893 8.595 0.00 0.00 C

ATOM 3223 HG11 ILE X 207 -5.361 -5.846 8.173 0.00 0.00 H

ATOM 3224 HG12 ILE X 207 -5.701 -4.147 8.185 0.00 0.00 H

ATOM 3225 CD ILE X 207 -5.256 -4.797 10.162 0.00 0.00 C

ATOM 3226 HD1 ILE X 207 -4.771 -5.666 10.635 0.00 0.00 H

ATOM 3227 HD2 ILE X 207 -6.339 -4.809 10.397 0.00 0.00 H

ATOM 3228 HD3 ILE X 207 -4.702 -3.922 10.577 0.00 0.00 H

ATOM 3229 C ILE X 207 -2.068 -4.675 6.106 0.00 0.00 C

ATOM 3230 O ILE X 207 -2.040 -3.537 5.539 0.00 0.00 O

ATOM 3231 N PHE X 208 -1.015 -5.379 6.348 0.00 0.00 N

ATOM 3232 HN PHE X 208 -1.146 -6.264 6.786 0.00 0.00 H

ATOM 3233 CA PHE X 208 0.377 -5.142 5.900 0.00 0.00 C

ATOM 3234 HA PHE X 208 0.641 -4.153 6.256 0.00 0.00 H

ATOM 3235 CB PHE X 208 1.344 -6.088 6.633 0.00 0.00 C

ATOM 3236 HB1 PHE X 208 0.986 -7.145 6.583 0.00 0.00 H

ATOM 3237 HB2 PHE X 208 1.284 -5.869 7.710 0.00 0.00 H

ATOM 3238 CG PHE X 208 2.743 -6.040 6.187 0.00 0.00 C

ATOM 3239 CD1 PHE X 208 3.520 -4.850 6.405 0.00 0.00 C

ATOM 3240 HD1 PHE X 208 3.085 -3.984 6.884 0.00 0.00 H

ATOM 3241 CE1 PHE X 208 4.896 -4.815 6.059 0.00 0.00 C

ATOM 3242 HE1 PHE X 208 5.394 -3.898 6.344 0.00 0.00 H

ATOM 3243 CZ PHE X 208 5.493 -5.916 5.542 0.00 0.00 C

ATOM 3244 HZ PHE X 208 6.532 -5.869 5.249 0.00 0.00 H

ATOM 3245 CD2 PHE X 208 3.362 -7.134 5.622 0.00 0.00 C

ATOM 3246 HD2 PHE X 208 2.766 -8.000 5.382 0.00 0.00 H

ATOM 3247 CE2 PHE X 208 4.727 -7.077 5.223 0.00 0.00 C

ATOM 3248 HE2 PHE X 208 5.247 -7.980 4.936 0.00 0.00 H

ATOM 3249 C PHE X 208 0.496 -5.206 4.344 0.00 0.00 C

ATOM 3250 O PHE X 208 1.070 -4.342 3.624 0.00 0.00 O

ATOM 3251 N PHE X 209 -0.090 -6.301 3.718 0.00 0.00 N

ATOM 3252 HN PHE X 209 -0.604 -6.984 4.231 0.00 0.00 H

ATOM 3253 CA PHE X 209 0.280 -6.697 2.323 0.00 0.00 C

ATOM 3254 HA PHE X 209 1.358 -6.794 2.366 0.00 0.00 H

ATOM 3255 CB PHE X 209 -0.422 -8.151 2.020 0.00 0.00 C

ATOM 3256 HB1 PHE X 209 -1.465 -8.144 2.380 0.00 0.00 H

ATOM 3257 HB2 PHE X 209 0.142 -8.915 2.606 0.00 0.00 H

ATOM 3258 CG PHE X 209 -0.552 -8.649 0.628 0.00 0.00 C

ATOM 3259 CD1 PHE X 209 -1.792 -8.751 -0.066 0.00 0.00 C

ATOM 3260 HD1 PHE X 209 -2.741 -8.499 0.379 0.00 0.00 H

ATOM 3261 CE1 PHE X 209 -1.821 -9.152 -1.423 0.00 0.00 C

ATOM 3262 HE1 PHE X 209 -2.762 -9.340 -1.917 0.00 0.00 H

ATOM 3263 CZ PHE X 209 -0.654 -9.641 -2.009 0.00 0.00 C

ATOM 3264 HZ PHE X 209 -0.703 -9.917 -3.055 0.00 0.00 H

ATOM 3265 CD2 PHE X 209 0.671 -8.904 -0.109 0.00 0.00 C

ATOM 3266 HD2 PHE X 209 1.565 -8.852 0.499 0.00 0.00 H

ATOM 3267 CE2 PHE X 209 0.604 -9.412 -1.434 0.00 0.00 C

ATOM 3268 HE2 PHE X 209 1.550 -9.734 -1.853 0.00 0.00 H

ATOM 3269 C PHE X 209 -0.027 -5.705 1.175 0.00 0.00 C

ATOM 3270 O PHE X 209 0.940 -5.419 0.425 0.00 0.00 O

ATOM 3271 N PRO X 210 -1.115 -5.048 1.033 0.00 0.00 N

ATOM 3272 CD PRO X 210 -2.432 -5.326 1.694 0.00 0.00 C

ATOM 3273 HD1 PRO X 210 -2.446 -4.959 2.747 0.00 0.00 H

ATOM 3274 HD2 PRO X 210 -2.663 -6.419 1.731 0.00 0.00 H

ATOM 3275 CA PRO X 210 -1.253 -3.946 0.047 0.00 0.00 C

ATOM 3276 HA PRO X 210 -1.289 -4.408 -0.924 0.00 0.00 H

ATOM 3277 CB PRO X 210 -2.586 -3.347 0.568 0.00 0.00 C

ATOM 3278 HB1 PRO X 210 -3.014 -2.743 -0.255 0.00 0.00 H

ATOM 3279 HB2 PRO X 210 -2.404 -2.583 1.354 0.00 0.00 H

ATOM 3280 CG PRO X 210 -3.512 -4.542 1.030 0.00 0.00 C

ATOM 3281 HG1 PRO X 210 -4.405 -4.187 1.595 0.00 0.00 H

ATOM 3282 HG2 PRO X 210 -3.895 -5.033 0.114 0.00 0.00 H

ATOM 3283 C PRO X 210 -0.094 -2.907 -0.137 0.00 0.00 C

ATOM 3284 O PRO X 210 0.168 -2.402 -1.241 0.00 0.00 O

ATOM 3285 N ALA X 211 0.594 -2.655 0.990 0.00 0.00 N

ATOM 3286 HN ALA X 211 0.268 -3.176 1.765 0.00 0.00 H

ATOM 3287 CA ALA X 211 1.736 -1.724 1.073 0.00 0.00 C

ATOM 3288 HA ALA X 211 1.763 -1.193 0.143 0.00 0.00 H

ATOM 3289 CB ALA X 211 1.453 -0.771 2.229 0.00 0.00 C

ATOM 3290 HB1 ALA X 211 2.337 -0.105 2.341 0.00 0.00 H

ATOM 3291 HB2 ALA X 211 1.319 -1.256 3.214 0.00 0.00 H

ATOM 3292 HB3 ALA X 211 0.630 -0.038 2.050 0.00 0.00 H

ATOM 3293 C ALA X 211 3.074 -2.482 1.209 0.00 0.00 C

ATOM 3294 O ALA X 211 4.137 -1.860 1.455 0.00 0.00 O

ATOM 3295 N ALA X 212 3.094 -3.821 1.058 0.00 0.00 N

ATOM 3296 HN ALA X 212 2.194 -4.226 0.896 0.00 0.00 H

ATOM 3297 CA ALA X 212 4.306 -4.563 1.310 0.00 0.00 C

ATOM 3298 HA ALA X 212 5.029 -3.907 1.769 0.00 0.00 H

ATOM 3299 CB ALA X 212 3.922 -5.693 2.240 0.00 0.00 C

ATOM 3300 HB1 ALA X 212 3.452 -5.391 3.187 0.00 0.00 H

ATOM 3301 HB2 ALA X 212 4.913 -6.115 2.548 0.00 0.00 H

ATOM 3302 HB3 ALA X 212 3.284 -6.434 1.716 0.00 0.00 H

ATOM 3303 C ALA X 212 4.930 -5.108 -0.038 0.00 0.00 C

ATOM 3304 O ALA X 212 5.963 -5.771 -0.103 0.00 0.00 O

ATOM 3305 N THR X 213 4.213 -4.855 -1.131 0.00 0.00 N

ATOM 3306 HN THR X 213 3.399 -4.280 -1.096 0.00 0.00 H

ATOM 3307 CA THR X 213 4.674 -5.416 -2.414 0.00 0.00 C

ATOM 3308 HA THR X 213 5.730 -5.198 -2.515 0.00 0.00 H

ATOM 3309 CB THR X 213 4.508 -6.916 -2.655 0.00 0.00 C

ATOM 3310 HB THR X 213 5.106 -7.396 -1.842 0.00 0.00 H

ATOM 3311 OG1 THR X 213 5.167 -7.255 -3.902 0.00 0.00 O

ATOM 3312 HG1 THR X 213 5.447 -8.163 -3.771 0.00 0.00 H

ATOM 3313 CG2 THR X 213 3.117 -7.479 -2.558 0.00 0.00 C

ATOM 3314 HG21 THR X 213 2.747 -7.352 -1.518 0.00 0.00 H

ATOM 3315 HG22 THR X 213 3.112 -8.582 -2.717 0.00 0.00 H

ATOM 3316 HG23 THR X 213 2.395 -7.125 -3.326 0.00 0.00 H

ATOM 3317 C THR X 213 3.918 -4.653 -3.566 0.00 0.00 C

ATOM 3318 O THR X 213 2.874 -4.080 -3.314 0.00 0.00 O

ATOM 3319 N GLY X 214 4.410 -4.614 -4.865 0.00 0.00 N

ATOM 3320 HN GLY X 214 5.230 -5.167 -5.031 0.00 0.00 H

ATOM 3321 CA GLY X 214 3.975 -3.605 -5.877 0.00 0.00 C

ATOM 3322 HA1 GLY X 214 4.527 -2.710 -5.654 0.00 0.00 H

ATOM 3323 HA2 GLY X 214 2.914 -3.456 -5.801 0.00 0.00 H

ATOM 3324 C GLY X 214 4.423 -4.174 -7.242 0.00 0.00 C

ATOM 3325 O GLY X 214 4.961 -3.543 -8.153 0.00 0.00 O

ATOM 3326 N ILE X 215 4.415 -5.574 -7.261 0.00 0.00 N

ATOM 3327 HN ILE X 215 4.114 -6.047 -6.436 0.00 0.00 H

ATOM 3328 CA ILE X 215 4.937 -6.270 -8.418 0.00 0.00 C

ATOM 3329 HA ILE X 215 5.999 -6.082 -8.506 0.00 0.00 H

ATOM 3330 CB ILE X 215 4.861 -7.831 -8.312 0.00 0.00 C

ATOM 3331 HB ILE X 215 5.401 -8.128 -7.387 0.00 0.00 H

ATOM 3332 CG2 ILE X 215 3.527 -8.399 -7.916 0.00 0.00 C

ATOM 3333 HG21 ILE X 215 2.849 -8.332 -8.791 0.00 0.00 H

ATOM 3334 HG22 ILE X 215 3.034 -7.818 -7.111 0.00 0.00 H

ATOM 3335 HG23 ILE X 215 3.646 -9.447 -7.582 0.00 0.00 H

ATOM 3336 CG1 ILE X 215 5.488 -8.598 -9.433 0.00 0.00 C

ATOM 3337 HG11 ILE X 215 6.219 -7.966 -9.970 0.00 0.00 H

ATOM 3338 HG12 ILE X 215 4.698 -8.806 -10.191 0.00 0.00 H

ATOM 3339 CD ILE X 215 6.073 -9.935 -8.978 0.00 0.00 C

ATOM 3340 HD1 ILE X 215 6.852 -9.803 -8.196 0.00 0.00 H

ATOM 3341 HD2 ILE X 215 6.656 -10.285 -9.859 0.00 0.00 H

ATOM 3342 HD3 ILE X 215 5.252 -10.609 -8.670 0.00 0.00 H

ATOM 3343 C ILE X 215 4.309 -5.890 -9.802 0.00 0.00 C

ATOM 3344 O ILE X 215 5.049 -5.728 -10.801 0.00 0.00 O

ATOM 3345 N LEU X 216 3.023 -5.660 -9.897 0.00 0.00 N

ATOM 3346 HN LEU X 216 2.473 -5.657 -9.070 0.00 0.00 H

ATOM 3347 CA LEU X 216 2.315 -5.212 -11.113 0.00 0.00 C

ATOM 3348 HA LEU X 216 2.789 -5.747 -11.923 0.00 0.00 H

ATOM 3349 CB LEU X 216 0.863 -5.452 -10.832 0.00 0.00 C

ATOM 3350 HB1 LEU X 216 0.433 -4.803 -10.025 0.00 0.00 H

ATOM 3351 HB2 LEU X 216 0.724 -6.534 -10.634 0.00 0.00 H

ATOM 3352 CG LEU X 216 -0.056 -5.240 -12.116 0.00 0.00 C

ATOM 3353 HG LEU X 216 -0.060 -4.154 -12.380 0.00 0.00 H

ATOM 3354 CD1 LEU X 216 0.290 -6.099 -13.342 0.00 0.00 C

ATOM 3355 HD11 LEU X 216 -0.402 -5.855 -14.180 0.00 0.00 H

ATOM 3356 HD12 LEU X 216 0.172 -7.126 -12.934 0.00 0.00 H

ATOM 3357 HD13 LEU X 216 1.350 -5.925 -13.611 0.00 0.00 H

ATOM 3358 CD2 LEU X 216 -1.466 -5.636 -11.598 0.00 0.00 C

ATOM 3359 HD21 LEU X 216 -1.654 -5.221 -10.588 0.00 0.00 H

ATOM 3360 HD22 LEU X 216 -1.441 -6.739 -11.472 0.00 0.00 H

ATOM 3361 HD23 LEU X 216 -2.278 -5.317 -12.283 0.00 0.00 H

ATOM 3362 C LEU X 216 2.526 -3.770 -11.327 0.00 0.00 C

ATOM 3363 O LEU X 216 2.610 -3.360 -12.434 0.00 0.00 O

ATOM 3364 N ALA X 217 2.692 -2.939 -10.245 0.00 0.00 N

ATOM 3365 HN ALA X 217 2.341 -3.219 -9.357 0.00 0.00 H

ATOM 3366 CA ALA X 217 2.973 -1.490 -10.482 0.00 0.00 C

ATOM 3367 HA ALA X 217 2.226 -1.193 -11.198 0.00 0.00 H

ATOM 3368 CB ALA X 217 2.798 -0.784 -9.177 0.00 0.00 C

ATOM 3369 HB1 ALA X 217 2.769 0.323 -9.263 0.00 0.00 H

ATOM 3370 HB2 ALA X 217 3.559 -1.068 -8.430 0.00 0.00 H

ATOM 3371 HB3 ALA X 217 1.818 -1.172 -8.835 0.00 0.00 H

ATOM 3372 C ALA X 217 4.342 -1.133 -11.092 0.00 0.00 C

ATOM 3373 O ALA X 217 4.372 -0.310 -11.930 0.00 0.00 O

ATOM 3374 N GLY X 218 5.400 -1.906 -10.764 0.00 0.00 N

ATOM 3375 HN GLY X 218 5.193 -2.479 -9.972 0.00 0.00 H

ATOM 3376 CA GLY X 218 6.650 -1.765 -11.393 0.00 0.00 C

ATOM 3377 HA1 GLY X 218 7.249 -2.565 -10.972 0.00 0.00 H

ATOM 3378 HA2 GLY X 218 7.008 -0.753 -11.230 0.00 0.00 H

ATOM 3379 C GLY X 218 6.675 -2.017 -12.886 0.00 0.00 C

ATOM 3380 O GLY X 218 7.450 -1.468 -13.614 0.00 0.00 O

ATOM 3381 N ALA X 219 5.778 -2.919 -13.327 0.00 0.00 N

ATOM 3382 HN ALA X 219 5.045 -3.148 -12.676 0.00 0.00 H

ATOM 3383 CA ALA X 219 5.548 -3.376 -14.723 0.00 0.00 C

ATOM 3384 HA ALA X 219 6.489 -3.485 -15.238 0.00 0.00 H

ATOM 3385 CB ALA X 219 4.852 -4.718 -14.599 0.00 0.00 C

ATOM 3386 HB1 ALA X 219 4.262 -4.740 -13.664 0.00 0.00 H

ATOM 3387 HB2 ALA X 219 5.564 -5.564 -14.526 0.00 0.00 H

ATOM 3388 HB3 ALA X 219 4.223 -4.900 -15.502 0.00 0.00 H

ATOM 3389 C ALA X 219 4.776 -2.299 -15.544 0.00 0.00 C

ATOM 3390 O ALA X 219 4.559 -2.418 -16.750 0.00 0.00 O

ATOM 3391 N ASN X 220 4.255 -1.255 -14.854 0.00 0.00 N

ATOM 3392 HN ASN X 220 4.459 -1.201 -13.877 0.00 0.00 H

ATOM 3393 CA ASN X 220 3.394 -0.229 -15.439 0.00 0.00 C

ATOM 3394 HA ASN X 220 2.979 -0.656 -16.343 0.00 0.00 H

ATOM 3395 CB ASN X 220 2.269 0.120 -14.406 0.00 0.00 C

ATOM 3396 HB1 ASN X 220 2.819 0.875 -13.791 0.00 0.00 H

ATOM 3397 HB2 ASN X 220 1.922 -0.838 -13.977 0.00 0.00 H

ATOM 3398 CG ASN X 220 1.069 0.777 -15.064 0.00 0.00 C

ATOM 3399 OD1 ASN X 220 0.634 0.525 -16.174 0.00 0.00 O

ATOM 3400 ND2 ASN X 220 0.383 1.714 -14.435 0.00 0.00 N

ATOM 3401 HD21 ASN X 220 -0.457 2.050 -14.862 0.00 0.00 H

ATOM 3402 HD22 ASN X 220 0.470 1.777 -13.442 0.00 0.00 H

ATOM 3403 C ASN X 220 4.220 0.986 -15.883 0.00 0.00 C

ATOM 3404 O ASN X 220 3.680 2.104 -16.047 0.00 0.00 O

ATOM 3405 N ILE X 221 5.517 0.805 -16.103 0.00 0.00 N

ATOM 3406 HN ILE X 221 5.861 -0.098 -15.894 0.00 0.00 H

ATOM 3407 CA ILE X 221 6.393 1.812 -16.629 0.00 0.00 C

ATOM 3408 HA ILE X 221 6.176 2.684 -16.025 0.00 0.00 H

ATOM 3409 CB ILE X 221 7.888 1.366 -16.567 0.00 0.00 C

ATOM 3410 HB ILE X 221 8.236 1.199 -15.520 0.00 0.00 H

ATOM 3411 CG2 ILE X 221 8.016 -0.015 -17.197 0.00 0.00 C

ATOM 3412 HG21 ILE X 221 7.281 -0.738 -16.776 0.00 0.00 H

ATOM 3413 HG22 ILE X 221 8.953 -0.504 -16.851 0.00 0.00 H

ATOM 3414 HG23 ILE X 221 7.991 0.056 -18.304 0.00 0.00 H

ATOM 3415 CG1 ILE X 221 8.897 2.369 -17.239 0.00 0.00 C

ATOM 3416 HG11 ILE X 221 8.527 3.417 -17.248 0.00 0.00 H

ATOM 3417 HG12 ILE X 221 9.160 2.097 -18.281 0.00 0.00 H

ATOM 3418 CD ILE X 221 10.180 2.406 -16.346 0.00 0.00 C

ATOM 3419 HD1 ILE X 221 10.392 1.352 -16.071 0.00 0.00 H

ATOM 3420 HD2 ILE X 221 10.005 2.932 -15.381 0.00 0.00 H

ATOM 3421 HD3 ILE X 221 11.019 2.678 -17.018 0.00 0.00 H

ATOM 3422 C ILE X 221 5.972 2.178 -18.070 0.00 0.00 C

ATOM 3423 O ILE X 221 5.509 1.383 -18.864 0.00 0.00 O

ATOM 3424 N SER X 222 6.050 3.536 -18.514 0.00 0.00 N

ATOM 3425 HN SER X 222 6.403 4.264 -17.930 0.00 0.00 H

ATOM 3426 CA SER X 222 5.607 3.873 -19.876 0.00 0.00 C

ATOM 3427 HA SER X 222 4.707 3.288 -20.026 0.00 0.00 H

ATOM 3428 CB SER X 222 5.390 5.389 -20.030 0.00 0.00 C

ATOM 3429 HB1 SER X 222 4.560 5.601 -19.318 0.00 0.00 H

ATOM 3430 HB2 SER X 222 5.133 5.792 -21.033 0.00 0.00 H

ATOM 3431 OG SER X 222 6.492 6.165 -19.568 0.00 0.00 O

ATOM 3432 HG1 SER X 222 7.181 6.192 -20.241 0.00 0.00 H

ATOM 3433 C SER X 222 6.546 3.408 -20.942 0.00 0.00 C

ATOM 3434 O SER X 222 7.759 3.148 -20.712 0.00 0.00 O

ATOM 3435 N GLY X 223 6.028 3.182 -22.163 0.00 0.00 N

ATOM 3436 HN GLY X 223 5.095 3.489 -22.356 0.00 0.00 H

ATOM 3437 CA GLY X 223 6.854 2.524 -23.199 0.00 0.00 C

ATOM 3438 HA1 GLY X 223 6.119 2.788 -23.952 0.00 0.00 H

ATOM 3439 HA2 GLY X 223 7.017 1.502 -22.891 0.00 0.00 H

ATOM 3440 C GLY X 223 8.142 3.169 -23.696 0.00 0.00 C

ATOM 3441 O GLY X 223 8.515 4.230 -23.171 0.00 0.00 O

ATOM 3442 N ASP X 224 8.897 2.475 -24.565 0.00 0.00 N

ATOM 3443 HN ASP X 224 8.555 1.603 -24.928 0.00 0.00 H

ATOM 3444 CA ASP X 224 10.144 2.839 -25.230 0.00 0.00 C

ATOM 3445 HA ASP X 224 10.224 2.214 -26.107 0.00 0.00 H

ATOM 3446 CB ASP X 224 10.392 4.285 -25.682 0.00 0.00 C

ATOM 3447 HB1 ASP X 224 11.435 4.438 -26.022 0.00 0.00 H

ATOM 3448 HB2 ASP X 224 10.218 4.999 -24.851 0.00 0.00 H

ATOM 3449 CG ASP X 224 9.512 4.594 -26.897 0.00 0.00 C

ATOM 3450 OD1 ASP X 224 8.865 3.714 -27.514 0.00 0.00 O

ATOM 3451 OD2 ASP X 224 9.464 5.853 -27.187 0.00 0.00 O1-

ATOM 3452 C ASP X 224 11.285 2.500 -24.185 0.00 0.00 C

ATOM 3453 O ASP X 224 12.429 2.842 -24.517 0.00 0.00 O

ATOM 3454 N LEU X 225 11.088 1.573 -23.208 0.00 0.00 N

ATOM 3455 HN LEU X 225 10.143 1.255 -23.219 0.00 0.00 H

ATOM 3456 CA LEU X 225 12.175 1.171 -22.347 0.00 0.00 C

ATOM 3457 HA LEU X 225 12.823 1.992 -22.078 0.00 0.00 H

ATOM 3458 CB LEU X 225 11.459 0.730 -21.045 0.00 0.00 C

ATOM 3459 HB1 LEU X 225 10.882 1.558 -20.588 0.00 0.00 H

ATOM 3460 HB2 LEU X 225 12.271 0.514 -20.317 0.00 0.00 H

ATOM 3461 CG LEU X 225 10.635 -0.592 -21.027 0.00 0.00 C

ATOM 3462 HG LEU X 225 11.040 -1.278 -21.804 0.00 0.00 H

ATOM 3463 CD1 LEU X 225 10.780 -1.306 -19.723 0.00 0.00 C

ATOM 3464 HD11 LEU X 225 11.866 -1.412 -19.517 0.00 0.00 H

ATOM 3465 HD12 LEU X 225 10.258 -2.276 -19.837 0.00 0.00 H

ATOM 3466 HD13 LEU X 225 10.433 -0.792 -18.797 0.00 0.00 H

ATOM 3467 CD2 LEU X 225 9.179 -0.231 -21.361 0.00 0.00 C

ATOM 3468 HD21 LEU X 225 8.764 0.394 -20.540 0.00 0.00 H

ATOM 3469 HD22 LEU X 225 8.503 -1.112 -21.325 0.00 0.00 H

ATOM 3470 HD23 LEU X 225 9.043 0.088 -22.415 0.00 0.00 H

ATOM 3471 C LEU X 225 13.077 0.128 -22.774 0.00 0.00 C

ATOM 3472 O LEU X 225 12.702 -0.743 -23.584 0.00 0.00 O

ATOM 3473 N ALA X 226 14.337 0.243 -22.341 0.00 0.00 N

ATOM 3474 HN ALA X 226 14.637 0.975 -21.732 0.00 0.00 H

ATOM 3475 CA ALA X 226 15.364 -0.731 -22.608 0.00 0.00 C

ATOM 3476 HA ALA X 226 15.514 -0.780 -23.678 0.00 0.00 H

ATOM 3477 CB ALA X 226 16.654 -0.303 -21.982 0.00 0.00 C

ATOM 3478 HB1 ALA X 226 16.617 -0.478 -20.885 0.00 0.00 H

ATOM 3479 HB2 ALA X 226 16.759 0.788 -22.123 0.00 0.00 H

ATOM 3480 HB3 ALA X 226 17.592 -0.719 -22.426 0.00 0.00 H

ATOM 3481 C ALA X 226 15.148 -2.139 -22.125 0.00 0.00 C

ATOM 3482 O ALA X 226 14.675 -2.281 -20.968 0.00 0.00 O

ATOM 3483 N ASP X 227 15.488 -3.198 -22.844 0.00 0.00 N

ATOM 3484 HN ASP X 227 15.829 -3.185 -23.776 0.00 0.00 H

ATOM 3485 CA ASP X 227 15.355 -4.556 -22.486 0.00 0.00 C

ATOM 3486 HA ASP X 227 15.270 -5.128 -23.405 0.00 0.00 H

ATOM 3487 CB ASP X 227 16.664 -5.049 -21.867 0.00 0.00 C

ATOM 3488 HB1 ASP X 227 16.683 -6.160 -21.943 0.00 0.00 H

ATOM 3489 HB2 ASP X 227 16.695 -4.912 -20.774 0.00 0.00 H

ATOM 3490 CG ASP X 227 17.997 -4.552 -22.430 0.00 0.00 C

ATOM 3491 OD1 ASP X 227 18.054 -4.184 -23.618 0.00 0.00 O

ATOM 3492 OD2 ASP X 227 19.105 -4.715 -21.784 0.00 0.00 O1-

ATOM 3493 C ASP X 227 14.187 -5.057 -21.613 0.00 0.00 C

ATOM 3494 O ASP X 227 14.442 -5.757 -20.651 0.00 0.00 O

ATOM 3495 N PRO X 228 12.828 -4.760 -21.831 0.00 0.00 N

ATOM 3496 CD PRO X 228 12.405 -4.243 -23.132 0.00 0.00 C

ATOM 3497 HD1 PRO X 228 12.928 -4.533 -24.073 0.00 0.00 H

ATOM 3498 HD2 PRO X 228 12.365 -3.145 -22.967 0.00 0.00 H

ATOM 3499 CA PRO X 228 11.655 -5.181 -20.974 0.00 0.00 C

ATOM 3500 HA PRO X 228 11.891 -4.688 -20.043 0.00 0.00 H

ATOM 3501 CB PRO X 228 10.378 -4.841 -21.801 0.00 0.00 C

ATOM 3502 HB1 PRO X 228 10.040 -3.795 -21.706 0.00 0.00 H

ATOM 3503 HB2 PRO X 228 9.492 -5.510 -21.662 0.00 0.00 H

ATOM 3504 CG PRO X 228 10.893 -4.764 -23.282 0.00 0.00 C

ATOM 3505 HG1 PRO X 228 11.056 -5.743 -23.794 0.00 0.00 H

ATOM 3506 HG2 PRO X 228 10.368 -4.048 -23.962 0.00 0.00 H

ATOM 3507 C PRO X 228 11.639 -6.645 -20.545 0.00 0.00 C

ATOM 3508 O PRO X 228 11.113 -6.933 -19.485 0.00 0.00 O

ATOM 3509 N GLN X 229 12.194 -7.529 -21.384 0.00 0.00 N

ATOM 3510 HN GLN X 229 12.626 -7.161 -22.208 0.00 0.00 H

ATOM 3511 CA GLN X 229 12.273 -9.017 -21.065 0.00 0.00 C

ATOM 3512 HA GLN X 229 11.219 -9.216 -21.005 0.00 0.00 H

ATOM 3513 CB GLN X 229 13.014 -9.921 -22.154 0.00 0.00 C

ATOM 3514 HB1 GLN X 229 12.842 -10.979 -21.891 0.00 0.00 H

ATOM 3515 HB2 GLN X 229 14.125 -9.950 -22.212 0.00 0.00 H

ATOM 3516 CG GLN X 229 12.538 -9.775 -23.607 0.00 0.00 C

ATOM 3517 HG1 GLN X 229 11.431 -9.759 -23.487 0.00 0.00 H

ATOM 3518 HG2 GLN X 229 12.824 -10.701 -24.149 0.00 0.00 H

ATOM 3519 CD GLN X 229 13.066 -8.541 -24.316 0.00 0.00 C

ATOM 3520 OE1 GLN X 229 13.746 -7.705 -23.743 0.00 0.00 O

ATOM 3521 NE2 GLN X 229 12.875 -8.474 -25.612 0.00 0.00 N

ATOM 3522 HE21 GLN X 229 12.502 -9.293 -26.062 0.00 0.00 H

ATOM 3523 HE22 GLN X 229 13.198 -7.673 -26.104 0.00 0.00 H

ATOM 3524 C GLN X 229 13.012 -9.287 -19.766 0.00 0.00 C

ATOM 3525 O GLN X 229 12.654 -10.206 -18.999 0.00 0.00 O

ATOM 3526 N SER X 230 14.173 -8.597 -19.504 0.00 0.00 N

ATOM 3527 HN SER X 230 14.543 -7.889 -20.106 0.00 0.00 H

ATOM 3528 CA SER X 230 14.893 -8.681 -18.341 0.00 0.00 C

ATOM 3529 HA SER X 230 14.627 -9.525 -17.740 0.00 0.00 H

ATOM 3530 CB SER X 230 16.334 -8.728 -18.635 0.00 0.00 C

ATOM 3531 HB1 SER X 230 16.559 -9.589 -19.289 0.00 0.00 H

ATOM 3532 HB2 SER X 230 17.036 -8.917 -17.789 0.00 0.00 H

ATOM 3533 OG SER X 230 16.929 -7.573 -19.362 0.00 0.00 O

ATOM 3534 HG1 SER X 230 17.710 -7.309 -18.861 0.00 0.00 H

ATOM 3535 C SER X 230 14.678 -7.598 -17.358 0.00 0.00 C

ATOM 3536 O SER X 230 14.660 -7.922 -16.119 0.00 0.00 O

ATOM 3537 N ALA X 231 14.687 -6.284 -17.787 0.00 0.00 N

ATOM 3538 HN ALA X 231 14.728 -6.003 -18.749 0.00 0.00 H

ATOM 3539 CA ALA X 231 14.666 -5.153 -16.947 0.00 0.00 C

ATOM 3540 HA ALA X 231 15.617 -5.160 -16.429 0.00 0.00 H

ATOM 3541 CB ALA X 231 14.651 -3.832 -17.823 0.00 0.00 C

ATOM 3542 HB1 ALA X 231 14.758 -2.923 -17.196 0.00 0.00 H

ATOM 3543 HB2 ALA X 231 13.691 -3.810 -18.362 0.00 0.00 H

ATOM 3544 HB3 ALA X 231 15.514 -3.862 -18.523 0.00 0.00 H

ATOM 3545 C ALA X 231 13.495 -5.066 -16.012 0.00 0.00 C

ATOM 3546 O ALA X 231 13.667 -4.752 -14.845 0.00 0.00 O

ATOM 3547 N ILE X 232 12.212 -5.342 -16.456 0.00 0.00 N

ATOM 3548 HN ILE X 232 12.053 -5.534 -17.419 0.00 0.00 H

ATOM 3549 CA ILE X 232 11.020 -5.429 -15.623 0.00 0.00 C

ATOM 3550 HA ILE X 232 11.173 -4.531 -15.041 0.00 0.00 H

ATOM 3551 CB ILE X 232 9.663 -5.249 -16.347 0.00 0.00 C

ATOM 3552 HB ILE X 232 9.545 -5.997 -17.157 0.00 0.00 H

ATOM 3553 CG2 ILE X 232 8.477 -5.580 -15.477 0.00 0.00 C

ATOM 3554 HG21 ILE X 232 7.440 -5.456 -15.871 0.00 0.00 H

ATOM 3555 HG22 ILE X 232 8.534 -4.966 -14.554 0.00 0.00 H

ATOM 3556 HG23 ILE X 232 8.401 -6.667 -15.272 0.00 0.00 H

ATOM 3557 CG1 ILE X 232 9.490 -3.868 -16.902 0.00 0.00 C

ATOM 3558 HG11 ILE X 232 10.423 -3.489 -17.349 0.00 0.00 H

ATOM 3559 HG12 ILE X 232 9.195 -3.208 -16.050 0.00 0.00 H

ATOM 3560 CD ILE X 232 8.376 -3.760 -17.998 0.00 0.00 C

ATOM 3561 HD1 ILE X 232 8.281 -2.783 -18.527 0.00 0.00 H

ATOM 3562 HD2 ILE X 232 7.419 -4.002 -17.502 0.00 0.00 H

ATOM 3563 HD3 ILE X 232 8.568 -4.526 -18.773 0.00 0.00 H

ATOM 3564 C ILE X 232 11.072 -6.575 -14.543 0.00 0.00 C

ATOM 3565 O ILE X 232 10.997 -6.362 -13.346 0.00 0.00 O

ATOM 3566 N PRO X 233 11.337 -7.852 -14.812 0.00 0.00 N

ATOM 3567 CD PRO X 233 11.245 -8.453 -16.083 0.00 0.00 C

ATOM 3568 HD1 PRO X 233 12.315 -8.398 -16.383 0.00 0.00 H

ATOM 3569 HD2 PRO X 233 10.446 -8.116 -16.787 0.00 0.00 H

ATOM 3570 CA PRO X 233 11.564 -8.912 -13.780 0.00 0.00 C

ATOM 3571 HA PRO X 233 10.629 -8.975 -13.232 0.00 0.00 H

ATOM 3572 CB PRO X 233 11.815 -10.199 -14.665 0.00 0.00 C

ATOM 3573 HB1 PRO X 233 11.638 -11.104 -14.060 0.00 0.00 H

ATOM 3574 HB2 PRO X 233 12.897 -10.130 -14.924 0.00 0.00 H

ATOM 3575 CG PRO X 233 10.959 -9.961 -15.893 0.00 0.00 C

ATOM 3576 HG1 PRO X 233 11.184 -10.509 -16.830 0.00 0.00 H

ATOM 3577 HG2 PRO X 233 9.876 -10.030 -15.634 0.00 0.00 H

ATOM 3578 C PRO X 233 12.620 -8.673 -12.783 0.00 0.00 C

ATOM 3579 O PRO X 233 12.288 -8.845 -11.639 0.00 0.00 O

ATOM 3580 N LYS X 234 13.824 -8.262 -13.254 0.00 0.00 N

ATOM 3581 HN LYS X 234 13.919 -8.167 -14.238 0.00 0.00 H

ATOM 3582 CA LYS X 234 14.854 -7.907 -12.338 0.00 0.00 C

ATOM 3583 HA LYS X 234 14.923 -8.704 -11.612 0.00 0.00 H

ATOM 3584 CB LYS X 234 16.190 -7.712 -13.163 0.00 0.00 C

ATOM 3585 HB1 LYS X 234 16.948 -7.403 -12.405 0.00 0.00 H

ATOM 3586 HB2 LYS X 234 16.048 -6.864 -13.870 0.00 0.00 H

ATOM 3587 CG LYS X 234 16.718 -8.920 -13.943 0.00 0.00 C

ATOM 3588 HG1 LYS X 234 15.993 -9.391 -14.636 0.00 0.00 H

ATOM 3589 HG2 LYS X 234 17.000 -9.693 -13.195 0.00 0.00 H

ATOM 3590 CD LYS X 234 18.071 -8.652 -14.680 0.00 0.00 C

ATOM 3591 HD1 LYS X 234 18.816 -8.095 -14.071 0.00 0.00 H

ATOM 3592 HD2 LYS X 234 17.800 -8.009 -15.538 0.00 0.00 H

ATOM 3593 CE LYS X 234 18.654 -9.926 -15.237 0.00 0.00 C

ATOM 3594 HE1 LYS X 234 17.941 -10.491 -15.872 0.00 0.00 H

ATOM 3595 HE2 LYS X 234 18.889 -10.621 -14.399 0.00 0.00 H

ATOM 3596 NZ LYS X 234 19.808 -9.612 -15.989 0.00 0.00 N1+

ATOM 3597 HZ1 LYS X 234 20.499 -9.214 -15.315 0.00 0.00 H

ATOM 3598 HZ2 LYS X 234 19.644 -8.890 -16.710 0.00 0.00 H

ATOM 3599 HZ3 LYS X 234 20.261 -10.416 -16.461 0.00 0.00 H

ATOM 3600 C LYS X 234 14.553 -6.704 -11.463 0.00 0.00 C

ATOM 3601 O LYS X 234 14.818 -6.726 -10.283 0.00 0.00 O

ATOM 3602 N GLY X 235 13.859 -5.675 -12.051 0.00 0.00 N

ATOM 3603 HN GLY X 235 13.620 -5.642 -13.016 0.00 0.00 H

ATOM 3604 CA GLY X 235 13.574 -4.483 -11.334 0.00 0.00 C

ATOM 3605 HA1 GLY X 235 13.416 -3.682 -12.040 0.00 0.00 H

ATOM 3606 HA2 GLY X 235 14.493 -4.209 -10.819 0.00 0.00 H

ATOM 3607 C GLY X 235 12.463 -4.697 -10.413 0.00 0.00 C

ATOM 3608 O GLY X 235 12.467 -4.067 -9.386 0.00 0.00 O

ATOM 3609 N THR X 236 11.365 -5.413 -10.818 0.00 0.00 N

ATOM 3610 HN THR X 236 11.393 -5.870 -11.712 0.00 0.00 H

ATOM 3611 CA THR X 236 10.380 -5.698 -9.797 0.00 0.00 C

ATOM 3612 HA THR X 236 9.975 -4.823 -9.305 0.00 0.00 H

ATOM 3613 CB THR X 236 9.105 -6.330 -10.399 0.00 0.00 C

ATOM 3614 HB THR X 236 8.498 -6.778 -9.578 0.00 0.00 H

ATOM 3615 OG1 THR X 236 9.402 -7.386 -11.234 0.00 0.00 O

ATOM 3616 HG1 THR X 236 9.870 -6.971 -11.976 0.00 0.00 H

ATOM 3617 CG2 THR X 236 8.402 -5.168 -11.117 0.00 0.00 C

ATOM 3618 HG21 THR X 236 9.123 -4.551 -11.704 0.00 0.00 H

ATOM 3619 HG22 THR X 236 7.969 -4.492 -10.352 0.00 0.00 H

ATOM 3620 HG23 THR X 236 7.577 -5.510 -11.789 0.00 0.00 H

ATOM 3621 C THR X 236 10.789 -6.500 -8.651 0.00 0.00 C

ATOM 3622 O THR X 236 10.586 -6.169 -7.475 0.00 0.00 O

ATOM 3623 N LEU X 237 11.516 -7.659 -8.898 0.00 0.00 N

ATOM 3624 HN LEU X 237 11.481 -8.040 -9.814 0.00 0.00 H

ATOM 3625 CA LEU X 237 12.116 -8.494 -7.950 0.00 0.00 C

ATOM 3626 HA LEU X 237 11.327 -8.717 -7.246 0.00 0.00 H

ATOM 3627 CB LEU X 237 12.614 -9.813 -8.595 0.00 0.00 C

ATOM 3628 HB1 LEU X 237 13.282 -10.415 -7.948 0.00 0.00 H

ATOM 3629 HB2 LEU X 237 13.179 -9.541 -9.519 0.00 0.00 H

ATOM 3630 CG LEU X 237 11.330 -10.633 -8.943 0.00 0.00 C

ATOM 3631 HG LEU X 237 10.557 -9.982 -9.423 0.00 0.00 H

ATOM 3632 CD1 LEU X 237 11.668 -11.694 -10.005 0.00 0.00 C

ATOM 3633 HD11 LEU X 237 12.345 -12.451 -9.568 0.00 0.00 H

ATOM 3634 HD12 LEU X 237 12.411 -11.244 -10.690 0.00 0.00 H

ATOM 3635 HD13 LEU X 237 10.708 -12.000 -10.472 0.00 0.00 H

ATOM 3636 CD2 LEU X 237 10.746 -11.356 -7.678 0.00 0.00 C

ATOM 3637 HD21 LEU X 237 11.610 -11.921 -7.274 0.00 0.00 H

ATOM 3638 HD22 LEU X 237 9.997 -12.028 -8.139 0.00 0.00 H

ATOM 3639 HD23 LEU X 237 10.452 -10.599 -6.924 0.00 0.00 H

ATOM 3640 C LEU X 237 13.110 -7.738 -7.106 0.00 0.00 C

ATOM 3641 O LEU X 237 13.268 -8.100 -5.946 0.00 0.00 O

ATOM 3642 N LEU X 238 13.920 -6.816 -7.583 0.00 0.00 N

ATOM 3643 HN LEU X 238 13.860 -6.653 -8.558 0.00 0.00 H

ATOM 3644 CA LEU X 238 14.807 -6.138 -6.704 0.00 0.00 C

ATOM 3645 HA LEU X 238 15.274 -6.904 -6.098 0.00 0.00 H

ATOM 3646 CB LEU X 238 15.912 -5.354 -7.503 0.00 0.00 C

ATOM 3647 HB1 LEU X 238 15.425 -4.639 -8.198 0.00 0.00 H

ATOM 3648 HB2 LEU X 238 16.445 -6.122 -8.099 0.00 0.00 H

ATOM 3649 CG LEU X 238 17.068 -4.567 -6.693 0.00 0.00 C

ATOM 3650 HG LEU X 238 16.590 -3.646 -6.302 0.00 0.00 H

ATOM 3651 CD1 LEU X 238 17.601 -5.512 -5.604 0.00 0.00 C

ATOM 3652 HD11 LEU X 238 16.947 -5.594 -4.712 0.00 0.00 H

ATOM 3653 HD12 LEU X 238 18.581 -5.135 -5.250 0.00 0.00 H

ATOM 3654 HD13 LEU X 238 17.788 -6.565 -5.897 0.00 0.00 H

ATOM 3655 CD2 LEU X 238 18.165 -4.273 -7.661 0.00 0.00 C

ATOM 3656 HD21 LEU X 238 18.737 -5.154 -8.045 0.00 0.00 H

ATOM 3657 HD22 LEU X 238 18.927 -3.741 -7.059 0.00 0.00 H

ATOM 3658 HD23 LEU X 238 17.713 -3.671 -8.477 0.00 0.00 H

ATOM 3659 C LEU X 238 14.144 -5.238 -5.667 0.00 0.00 C

ATOM 3660 O LEU X 238 14.533 -5.135 -4.533 0.00 0.00 O

ATOM 3661 N ALA X 239 13.086 -4.536 -5.990 0.00 0.00 N

ATOM 3662 HN ALA X 239 12.750 -4.577 -6.932 0.00 0.00 H

ATOM 3663 CA ALA X 239 12.322 -3.785 -5.015 0.00 0.00 C

ATOM 3664 HA ALA X 239 13.018 -3.042 -4.665 0.00 0.00 H

ATOM 3665 CB ALA X 239 11.190 -3.003 -5.795 0.00 0.00 C

ATOM 3666 HB1 ALA X 239 11.533 -2.291 -6.581 0.00 0.00 H

ATOM 3667 HB2 ALA X 239 10.505 -2.391 -5.154 0.00 0.00 H

ATOM 3668 HB3 ALA X 239 10.576 -3.762 -6.306 0.00 0.00 H

ATOM 3669 C ALA X 239 11.734 -4.562 -3.868 0.00 0.00 C

ATOM 3670 O ALA X 239 11.821 -4.195 -2.693 0.00 0.00 O

ATOM 3671 N ILE X 240 11.206 -5.811 -4.135 0.00 0.00 N

ATOM 3672 HN ILE X 240 11.194 -6.097 -5.085 0.00 0.00 H

ATOM 3673 CA ILE X 240 10.781 -6.681 -3.045 0.00 0.00 C

ATOM 3674 HA ILE X 240 10.087 -6.182 -2.388 0.00 0.00 H

ATOM 3675 CB ILE X 240 9.893 -7.938 -3.472 0.00 0.00 C

ATOM 3676 HB ILE X 240 9.101 -7.476 -4.093 0.00 0.00 H

ATOM 3677 CG2 ILE X 240 10.634 -8.882 -4.498 0.00 0.00 C

ATOM 3678 HG21 ILE X 240 10.862 -8.496 -5.517 0.00 0.00 H

ATOM 3679 HG22 ILE X 240 10.037 -9.758 -4.807 0.00 0.00 H

ATOM 3680 HG23 ILE X 240 11.561 -9.261 -4.018 0.00 0.00 H

ATOM 3681 CG1 ILE X 240 9.317 -8.748 -2.352 0.00 0.00 C

ATOM 3682 HG11 ILE X 240 10.081 -8.864 -1.545 0.00 0.00 H

ATOM 3683 HG12 ILE X 240 9.228 -9.799 -2.695 0.00 0.00 H

ATOM 3684 CD ILE X 240 8.002 -8.159 -1.818 0.00 0.00 C

ATOM 3685 HD1 ILE X 240 7.673 -8.874 -1.031 0.00 0.00 H

ATOM 3686 HD2 ILE X 240 7.190 -8.190 -2.581 0.00 0.00 H

ATOM 3687 HD3 ILE X 240 8.140 -7.130 -1.438 0.00 0.00 H

ATOM 3688 C ILE X 240 11.921 -7.051 -2.039 0.00 0.00 C

ATOM 3689 O ILE X 240 11.715 -6.913 -0.841 0.00 0.00 O

ATOM 3690 N LEU X 241 13.127 -7.485 -2.504 0.00 0.00 N

ATOM 3691 HN LEU X 241 13.295 -7.461 -3.485 0.00 0.00 H

ATOM 3692 CA LEU X 241 14.270 -7.610 -1.665 0.00 0.00 C

ATOM 3693 HA LEU X 241 14.053 -8.425 -0.999 0.00 0.00 H

ATOM 3694 CB LEU X 241 15.549 -7.928 -2.603 0.00 0.00 C

ATOM 3695 HB1 LEU X 241 15.622 -7.156 -3.403 0.00 0.00 H

ATOM 3696 HB2 LEU X 241 15.451 -8.964 -2.968 0.00 0.00 H

ATOM 3697 CG LEU X 241 17.004 -7.835 -1.957 0.00 0.00 C

ATOM 3698 HG LEU X 241 17.086 -6.841 -1.469 0.00 0.00 H

ATOM 3699 CD1 LEU X 241 17.081 -9.000 -0.873 0.00 0.00 C

ATOM 3700 HD11 LEU X 241 16.406 -8.870 -0.007 0.00 0.00 H

ATOM 3701 HD12 LEU X 241 18.135 -9.123 -0.548 0.00 0.00 H

ATOM 3702 HD13 LEU X 241 16.790 -9.953 -1.365 0.00 0.00 H

ATOM 3703 CD2 LEU X 241 18.056 -8.184 -2.988 0.00 0.00 C

ATOM 3704 HD21 LEU X 241 17.805 -7.501 -3.827 0.00 0.00 H

ATOM 3705 HD22 LEU X 241 17.911 -9.250 -3.256 0.00 0.00 H

ATOM 3706 HD23 LEU X 241 19.089 -8.141 -2.586 0.00 0.00 H

ATOM 3707 C LEU X 241 14.668 -6.418 -0.834 0.00 0.00 C

ATOM 3708 O LEU X 241 14.913 -6.449 0.375 0.00 0.00 O

ATOM 3709 N ILE X 242 14.750 -5.172 -1.492 0.00 0.00 N

ATOM 3710 HN ILE X 242 14.595 -5.135 -2.479 0.00 0.00 H

ATOM 3711 CA ILE X 242 15.096 -3.961 -0.799 0.00 0.00 C

ATOM 3712 HA ILE X 242 15.941 -4.181 -0.168 0.00 0.00 H

ATOM 3713 CB ILE X 242 15.454 -2.842 -1.782 0.00 0.00 C

ATOM 3714 HB ILE X 242 14.537 -2.767 -2.411 0.00 0.00 H

ATOM 3715 CG2 ILE X 242 15.560 -1.378 -1.162 0.00 0.00 C

ATOM 3716 HG21 ILE X 242 16.323 -1.458 -0.347 0.00 0.00 H

ATOM 3717 HG22 ILE X 242 14.610 -1.074 -0.681 0.00 0.00 H

ATOM 3718 HG23 ILE X 242 15.867 -0.666 -1.952 0.00 0.00 H

ATOM 3719 CG1 ILE X 242 16.593 -3.164 -2.765 0.00 0.00 C

ATOM 3720 HG11 ILE X 242 16.387 -4.159 -3.195 0.00 0.00 H

ATOM 3721 HG12 ILE X 242 17.545 -3.249 -2.202 0.00 0.00 H

ATOM 3722 CD ILE X 242 16.675 -2.182 -3.850 0.00 0.00 C

ATOM 3723 HD1 ILE X 242 17.215 -1.258 -3.540 0.00 0.00 H

ATOM 3724 HD2 ILE X 242 15.652 -1.907 -4.181 0.00 0.00 H

ATOM 3725 HD3 ILE X 242 17.196 -2.450 -4.793 0.00 0.00 H

ATOM 3726 C ILE X 242 14.138 -3.480 0.204 0.00 0.00 C

ATOM 3727 O ILE X 242 14.467 -2.999 1.307 0.00 0.00 O

ATOM 3728 N THR X 243 12.832 -3.651 -0.068 0.00 0.00 N

ATOM 3729 HN THR X 243 12.610 -4.213 -0.862 0.00 0.00 H

ATOM 3730 CA THR X 243 11.834 -3.148 0.767 0.00 0.00 C

ATOM 3731 HA THR X 243 12.144 -2.146 1.007 0.00 0.00 H

ATOM 3732 CB THR X 243 10.493 -2.849 0.046 0.00 0.00 C

ATOM 3733 HB THR X 243 9.743 -2.656 0.849 0.00 0.00 H

ATOM 3734 OG1 THR X 243 10.052 -3.954 -0.654 0.00 0.00 O

ATOM 3735 HG1 THR X 243 10.611 -4.028 -1.425 0.00 0.00 H

ATOM 3736 CG2 THR X 243 10.613 -1.672 -0.855 0.00 0.00 C

ATOM 3737 HG21 THR X 243 11.176 -1.897 -1.787 0.00 0.00 H

ATOM 3738 HG22 THR X 243 11.156 -0.841 -0.357 0.00 0.00 H

ATOM 3739 HG23 THR X 243 9.639 -1.177 -1.065 0.00 0.00 H

ATOM 3740 C THR X 243 11.549 -4.055 1.966 0.00 0.00 C

ATOM 3741 O THR X 243 11.527 -3.632 3.115 0.00 0.00 O

ATOM 3742 N THR X 244 11.608 -5.371 1.707 0.00 0.00 N

ATOM 3743 HN THR X 244 11.811 -5.783 0.819 0.00 0.00 H

ATOM 3744 CA THR X 244 11.550 -6.382 2.725 0.00 0.00 C

ATOM 3745 HA THR X 244 10.727 -6.231 3.402 0.00 0.00 H

ATOM 3746 CB THR X 244 11.523 -7.834 2.244 0.00 0.00 C

ATOM 3747 HB THR X 244 11.617 -8.454 3.162 0.00 0.00 H

ATOM 3748 OG1 THR X 244 12.500 -8.151 1.283 0.00 0.00 O

ATOM 3749 HG1 THR X 244 12.299 -7.722 0.456 0.00 0.00 H

ATOM 3750 CG2 THR X 244 10.134 -8.103 1.667 0.00 0.00 C

ATOM 3751 HG21 THR X 244 9.992 -7.518 0.725 0.00 0.00 H

ATOM 3752 HG22 THR X 244 9.238 -7.798 2.244 0.00 0.00 H

ATOM 3753 HG23 THR X 244 10.147 -9.196 1.489 0.00 0.00 H

ATOM 3754 C THR X 244 12.687 -6.287 3.780 0.00 0.00 C

ATOM 3755 O THR X 244 12.508 -6.206 4.955 0.00 0.00 O

ATOM 3756 N LEU X 245 13.991 -6.159 3.400 0.00 0.00 N

ATOM 3757 HN LEU X 245 14.124 -6.244 2.412 0.00 0.00 H

ATOM 3758 CA LEU X 245 15.075 -5.849 4.345 0.00 0.00 C

ATOM 3759 HA LEU X 245 15.122 -6.649 5.068 0.00 0.00 H

ATOM 3760 CB LEU X 245 16.430 -5.901 3.558 0.00 0.00 C

ATOM 3761 HB1 LEU X 245 17.275 -5.432 4.093 0.00 0.00 H

ATOM 3762 HB2 LEU X 245 16.183 -5.375 2.609 0.00 0.00 H

ATOM 3763 CG LEU X 245 16.833 -7.443 3.160 0.00 0.00 C

ATOM 3764 HG LEU X 245 15.963 -7.995 2.759 0.00 0.00 H

ATOM 3765 CD1 LEU X 245 17.908 -7.513 2.022 0.00 0.00 C

ATOM 3766 HD11 LEU X 245 17.478 -6.938 1.171 0.00 0.00 H

ATOM 3767 HD12 LEU X 245 17.968 -8.571 1.696 0.00 0.00 H

ATOM 3768 HD13 LEU X 245 18.869 -7.048 2.318 0.00 0.00 H

ATOM 3769 CD2 LEU X 245 17.249 -8.285 4.421 0.00 0.00 C

ATOM 3770 HD21 LEU X 245 17.448 -9.335 4.099 0.00 0.00 H

ATOM 3771 HD22 LEU X 245 16.444 -8.329 5.183 0.00 0.00 H

ATOM 3772 HD23 LEU X 245 18.239 -8.037 4.845 0.00 0.00 H

ATOM 3773 C LEU X 245 14.966 -4.488 5.079 0.00 0.00 C

ATOM 3774 O LEU X 245 15.211 -4.427 6.261 0.00 0.00 O

ATOM 3775 N VAL X 246 14.592 -3.369 4.389 0.00 0.00 N

ATOM 3776 HN VAL X 246 14.276 -3.457 3.453 0.00 0.00 H

ATOM 3777 CA VAL X 246 14.287 -2.093 5.108 0.00 0.00 C

ATOM 3778 HA VAL X 246 15.199 -1.811 5.633 0.00 0.00 H

ATOM 3779 CB VAL X 246 13.814 -0.991 4.076 0.00 0.00 C

ATOM 3780 HB VAL X 246 13.162 -1.417 3.277 0.00 0.00 H

ATOM 3781 CG1 VAL X 246 13.178 0.271 4.629 0.00 0.00 C

ATOM 3782 HG11 VAL X 246 13.050 1.003 3.810 0.00 0.00 H

ATOM 3783 HG12 VAL X 246 13.871 0.771 5.336 0.00 0.00 H

ATOM 3784 HG13 VAL X 246 12.236 0.057 5.175 0.00 0.00 H

ATOM 3785 CG2 VAL X 246 15.205 -0.632 3.468 0.00 0.00 C

ATOM 3786 HG21 VAL X 246 15.800 -1.428 2.981 0.00 0.00 H

ATOM 3787 HG22 VAL X 246 15.747 -0.359 4.396 0.00 0.00 H

ATOM 3788 HG23 VAL X 246 15.271 0.279 2.830 0.00 0.00 H

ATOM 3789 C VAL X 246 13.271 -2.252 6.228 0.00 0.00 C

ATOM 3790 O VAL X 246 13.573 -1.915 7.377 0.00 0.00 O

ATOM 3791 N TYR X 247 12.078 -2.913 5.980 0.00 0.00 N

ATOM 3792 HN TYR X 247 11.936 -3.059 5.007 0.00 0.00 H

ATOM 3793 CA TYR X 247 11.044 -3.315 6.922 0.00 0.00 C

ATOM 3794 HA TYR X 247 10.745 -2.423 7.455 0.00 0.00 H

ATOM 3795 CB TYR X 247 9.830 -4.049 6.254 0.00 0.00 C

ATOM 3796 HB1 TYR X 247 9.057 -4.471 6.923 0.00 0.00 H

ATOM 3797 HB2 TYR X 247 10.147 -4.925 5.651 0.00 0.00 H

ATOM 3798 CG TYR X 247 8.958 -3.039 5.426 0.00 0.00 C

ATOM 3799 CD1 TYR X 247 8.363 -1.912 6.032 0.00 0.00 C

ATOM 3800 HD1 TYR X 247 8.600 -1.826 7.081 0.00 0.00 H

ATOM 3801 CE1 TYR X 247 7.352 -1.126 5.396 0.00 0.00 C

ATOM 3802 HE1 TYR X 247 6.866 -0.395 6.020 0.00 0.00 H

ATOM 3803 CZ TYR X 247 7.100 -1.436 4.119 0.00 0.00 C

ATOM 3804 OH TYR X 247 6.210 -0.569 3.571 0.00 0.00 O

ATOM 3805 HH TYR X 247 6.030 -0.897 2.686 0.00 0.00 H

ATOM 3806 CD2 TYR X 247 8.520 -3.392 4.122 0.00 0.00 C

ATOM 3807 HD2 TYR X 247 8.939 -4.249 3.609 0.00 0.00 H

ATOM 3808 CE2 TYR X 247 7.691 -2.509 3.398 0.00 0.00 C

ATOM 3809 HE2 TYR X 247 7.535 -2.698 2.355 0.00 0.00 H

ATOM 3810 C TYR X 247 11.536 -4.206 8.073 0.00 0.00 C

ATOM 3811 O TYR X 247 11.281 -3.994 9.251 0.00 0.00 O

ATOM 3812 N VAL X 248 12.395 -5.317 7.717 0.00 0.00 N

ATOM 3813 HN VAL X 248 12.513 -5.506 6.744 0.00 0.00 H

ATOM 3814 CA VAL X 248 12.994 -6.140 8.754 0.00 0.00 C

ATOM 3815 HA VAL X 248 12.190 -6.494 9.365 0.00 0.00 H

ATOM 3816 CB VAL X 248 13.649 -7.398 8.224 0.00 0.00 C

ATOM 3817 HB VAL X 248 14.395 -7.229 7.427 0.00 0.00 H

ATOM 3818 CG1 VAL X 248 14.395 -8.130 9.392 0.00 0.00 C

ATOM 3819 HG11 VAL X 248 14.851 -9.049 8.948 0.00 0.00 H

ATOM 3820 HG12 VAL X 248 13.835 -8.476 10.281 0.00 0.00 H

ATOM 3821 HG13 VAL X 248 15.334 -7.658 9.755 0.00 0.00 H

ATOM 3822 CG2 VAL X 248 12.598 -8.317 7.535 0.00 0.00 C

ATOM 3823 HG21 VAL X 248 13.126 -9.204 7.147 0.00 0.00 H

ATOM 3824 HG22 VAL X 248 12.285 -7.835 6.581 0.00 0.00 H

ATOM 3825 HG23 VAL X 248 11.698 -8.580 8.129 0.00 0.00 H

ATOM 3826 C VAL X 248 13.922 -5.290 9.626 0.00 0.00 C

ATOM 3827 O VAL X 248 13.766 -5.350 10.812 0.00 0.00 O

ATOM 3828 N GLY X 249 14.895 -4.430 9.140 0.00 0.00 N

ATOM 3829 HN GLY X 249 15.104 -4.414 8.164 0.00 0.00 H

ATOM 3830 CA GLY X 249 15.694 -3.425 9.836 0.00 0.00 C

ATOM 3831 HA1 GLY X 249 16.193 -2.940 9.012 0.00 0.00 H

ATOM 3832 HA2 GLY X 249 16.432 -3.980 10.397 0.00 0.00 H

ATOM 3833 C GLY X 249 14.923 -2.383 10.671 0.00 0.00 C

ATOM 3834 O GLY X 249 15.114 -2.223 11.861 0.00 0.00 O

ATOM 3835 N ILE X 250 13.856 -1.733 10.108 0.00 0.00 N

ATOM 3836 HN ILE X 250 13.746 -1.871 9.127 0.00 0.00 H

ATOM 3837 CA ILE X 250 12.989 -0.935 10.868 0.00 0.00 C

ATOM 3838 HA ILE X 250 13.548 -0.165 11.372 0.00 0.00 H

ATOM 3839 CB ILE X 250 11.939 -0.241 9.931 0.00 0.00 C

ATOM 3840 HB ILE X 250 11.496 -1.108 9.396 0.00 0.00 H

ATOM 3841 CG2 ILE X 250 10.840 0.494 10.831 0.00 0.00 C

ATOM 3842 HG21 ILE X 250 10.035 -0.174 11.201 0.00 0.00 H

ATOM 3843 HG22 ILE X 250 10.259 1.263 10.273 0.00 0.00 H

ATOM 3844 HG23 ILE X 250 11.446 0.951 11.643 0.00 0.00 H

ATOM 3845 CG1 ILE X 250 12.660 0.873 8.998 0.00 0.00 C

ATOM 3846 HG11 ILE X 250 11.968 1.169 8.187 0.00 0.00 H

ATOM 3847 HG12 ILE X 250 13.482 0.250 8.585 0.00 0.00 H

ATOM 3848 CD ILE X 250 13.213 2.084 9.664 0.00 0.00 C

ATOM 3849 HD1 ILE X 250 14.099 1.806 10.283 0.00 0.00 H

ATOM 3850 HD2 ILE X 250 12.492 2.604 10.327 0.00 0.00 H

ATOM 3851 HD3 ILE X 250 13.523 2.825 8.892 0.00 0.00 H

ATOM 3852 C ILE X 250 12.284 -1.733 11.993 0.00 0.00 C

ATOM 3853 O ILE X 250 12.317 -1.284 13.146 0.00 0.00 O

ATOM 3854 N ALA X 251 11.670 -2.888 11.680 0.00 0.00 N

ATOM 3855 HN ALA X 251 11.679 -3.242 10.747 0.00 0.00 H

ATOM 3856 CA ALA X 251 10.982 -3.634 12.753 0.00 0.00 C

ATOM 3857 HA ALA X 251 10.245 -2.917 13.089 0.00 0.00 H

ATOM 3858 CB ALA X 251 10.375 -4.887 12.055 0.00 0.00 C

ATOM 3859 HB1 ALA X 251 9.673 -4.492 11.297 0.00 0.00 H

ATOM 3860 HB2 ALA X 251 9.841 -5.527 12.795 0.00 0.00 H

ATOM 3861 HB3 ALA X 251 11.150 -5.504 11.555 0.00 0.00 H

ATOM 3862 C ALA X 251 11.877 -4.034 13.936 0.00 0.00 C

ATOM 3863 O ALA X 251 11.465 -3.949 15.126 0.00 0.00 O

ATOM 3864 N VAL X 252 13.112 -4.524 13.620 0.00 0.00 N

ATOM 3865 HN VAL X 252 13.308 -4.779 12.680 0.00 0.00 H

ATOM 3866 CA VAL X 252 14.127 -4.972 14.577 0.00 0.00 C

ATOM 3867 HA VAL X 252 13.630 -5.561 15.338 0.00 0.00 H

ATOM 3868 CB VAL X 252 15.354 -5.687 13.911 0.00 0.00 C

ATOM 3869 HB VAL X 252 15.746 -5.096 13.058 0.00 0.00 H

ATOM 3870 CG1 VAL X 252 16.532 -5.862 14.918 0.00 0.00 C

ATOM 3871 HG11 VAL X 252 16.271 -6.388 15.862 0.00 0.00 H

ATOM 3872 HG12 VAL X 252 16.919 -4.847 15.135 0.00 0.00 H

ATOM 3873 HG13 VAL X 252 17.323 -6.426 14.377 0.00 0.00 H

ATOM 3874 CG2 VAL X 252 14.898 -7.014 13.345 0.00 0.00 C

ATOM 3875 HG21 VAL X 252 15.814 -7.571 13.038 0.00 0.00 H

ATOM 3876 HG22 VAL X 252 14.176 -7.045 12.492 0.00 0.00 H

ATOM 3877 HG23 VAL X 252 14.300 -7.726 13.962 0.00 0.00 H

ATOM 3878 C VAL X 252 14.523 -3.682 15.341 0.00 0.00 C

ATOM 3879 O VAL X 252 14.473 -3.653 16.573 0.00 0.00 O

ATOM 3880 N SER X 253 14.745 -2.500 14.682 0.00 0.00 N

ATOM 3881 HN SER X 253 14.713 -2.466 13.688 0.00 0.00 H

ATOM 3882 CA SER X 253 14.931 -1.189 15.300 0.00 0.00 C

ATOM 3883 HA SER X 253 15.761 -1.302 15.983 0.00 0.00 H

ATOM 3884 CB SER X 253 15.189 -0.075 14.237 0.00 0.00 C

ATOM 3885 HB1 SER X 253 14.416 -0.108 13.449 0.00 0.00 H

ATOM 3886 HB2 SER X 253 16.102 -0.389 13.692 0.00 0.00 H

ATOM 3887 OG SER X 253 15.340 1.255 14.638 0.00 0.00 O

ATOM 3888 HG1 SER X 253 16.187 1.301 15.081 0.00 0.00 H

ATOM 3889 C SER X 253 13.872 -0.718 16.283 0.00 0.00 C

ATOM 3890 O SER X 253 14.167 -0.658 17.470 0.00 0.00 O

ATOM 3891 N VAL X 254 12.567 -0.550 15.827 0.00 0.00 N

ATOM 3892 HN VAL X 254 12.347 -0.687 14.864 0.00 0.00 H

ATOM 3893 CA VAL X 254 11.552 0.040 16.766 0.00 0.00 C

ATOM 3894 HA VAL X 254 11.964 0.970 17.122 0.00 0.00 H

ATOM 3895 CB VAL X 254 10.187 0.410 16.048 0.00 0.00 C

ATOM 3896 HB VAL X 254 10.481 0.974 15.135 0.00 0.00 H

ATOM 3897 CG1 VAL X 254 9.250 -0.757 15.640 0.00 0.00 C

ATOM 3898 HG11 VAL X 254 9.056 -1.461 16.472 0.00 0.00 H

ATOM 3899 HG12 VAL X 254 9.566 -1.336 14.745 0.00 0.00 H

ATOM 3900 HG13 VAL X 254 8.310 -0.205 15.420 0.00 0.00 H

ATOM 3901 CG2 VAL X 254 9.530 1.439 16.946 0.00 0.00 C

ATOM 3902 HG21 VAL X 254 8.450 1.249 16.760 0.00 0.00 H

ATOM 3903 HG22 VAL X 254 9.779 2.490 16.692 0.00 0.00 H

ATOM 3904 HG23 VAL X 254 9.754 1.328 18.026 0.00 0.00 H

ATOM 3905 C VAL X 254 11.223 -0.987 17.826 0.00 0.00 C

ATOM 3906 O VAL X 254 10.834 -0.623 18.919 0.00 0.00 O

ATOM 3907 N GLY X 255 11.468 -2.320 17.508 0.00 0.00 N

ATOM 3908 HN GLY X 255 11.804 -2.515 16.590 0.00 0.00 H

ATOM 3909 CA GLY X 255 11.196 -3.287 18.561 0.00 0.00 C

ATOM 3910 HA1 GLY X 255 11.227 -4.253 18.084 0.00 0.00 H

ATOM 3911 HA2 GLY X 255 10.295 -3.062 19.113 0.00 0.00 H

ATOM 3912 C GLY X 255 12.227 -3.302 19.602 0.00 0.00 C

ATOM 3913 O GLY X 255 12.116 -3.877 20.616 0.00 0.00 O

ATOM 3914 N SER X 256 13.477 -2.772 19.411 0.00 0.00 N

ATOM 3915 HN SER X 256 13.523 -2.360 18.501 0.00 0.00 H

ATOM 3916 CA SER X 256 14.633 -3.013 20.137 0.00 0.00 C

ATOM 3917 HA SER X 256 14.577 -3.837 20.841 0.00 0.00 H

ATOM 3918 CB SER X 256 15.935 -3.056 19.325 0.00 0.00 C

ATOM 3919 HB1 SER X 256 16.852 -2.951 19.942 0.00 0.00 H

ATOM 3920 HB2 SER X 256 15.863 -2.170 18.653 0.00 0.00 H

ATOM 3921 OG SER X 256 16.044 -4.293 18.612 0.00 0.00 O

ATOM 3922 HG1 SER X 256 15.390 -4.296 17.906 0.00 0.00 H

ATOM 3923 C SER X 256 14.897 -1.761 20.910 0.00 0.00 C

ATOM 3924 O SER X 256 15.786 -1.834 21.778 0.00 0.00 O

ATOM 3925 N CYS X 257 14.137 -0.653 20.693 0.00 0.00 N

ATOM 3926 HN CYS X 257 13.589 -0.639 19.856 0.00 0.00 H

ATOM 3927 CA CYS X 257 14.375 0.486 21.560 0.00 0.00 C

ATOM 3928 HA CYS X 257 15.121 0.401 22.333 0.00 0.00 H

ATOM 3929 CB CYS X 257 15.092 1.613 20.747 0.00 0.00 C

ATOM 3930 HB1 CYS X 257 16.136 1.283 20.581 0.00 0.00 H

ATOM 3931 HB2 CYS X 257 15.268 2.488 21.404 0.00 0.00 H

ATOM 3932 SG CYS X 257 14.362 1.994 19.070 0.00 0.00 S

ATOM 3933 HG1 CYS X 257 14.631 0.785 18.574 0.00 0.00 H

ATOM 3934 C CYS X 257 13.189 1.008 22.304 0.00 0.00 C

ATOM 3935 O CYS X 257 13.361 1.891 23.131 0.00 0.00 O

ATOM 3936 N VAL X 258 11.959 0.466 22.135 0.00 0.00 N

ATOM 3937 HN VAL X 258 11.888 -0.319 21.524 0.00 0.00 H

ATOM 3938 CA VAL X 258 10.718 1.079 22.574 0.00 0.00 C

ATOM 3939 HA VAL X 258 10.937 1.868 23.281 0.00 0.00 H

ATOM 3940 CB VAL X 258 9.839 1.629 21.433 0.00 0.00 C

ATOM 3941 HB VAL X 258 9.569 0.685 20.902 0.00 0.00 H

ATOM 3942 CG1 VAL X 258 8.555 2.262 21.967 0.00 0.00 C

ATOM 3943 HG11 VAL X 258 8.743 3.288 22.353 0.00 0.00 H

ATOM 3944 HG12 VAL X 258 7.975 1.622 22.672 0.00 0.00 H

ATOM 3945 HG13 VAL X 258 7.842 2.534 21.165 0.00 0.00 H

ATOM 3946 CG2 VAL X 258 10.651 2.470 20.458 0.00 0.00 C

ATOM 3947 HG21 VAL X 258 11.543 1.916 20.108 0.00 0.00 H

ATOM 3948 HG22 VAL X 258 10.974 3.410 20.965 0.00 0.00 H

ATOM 3949 HG23 VAL X 258 10.236 2.696 19.460 0.00 0.00 H

ATOM 3950 C VAL X 258 10.003 0.090 23.418 0.00 0.00 C

ATOM 3951 O VAL X 258 9.793 -1.022 22.955 0.00 0.00 O

ATOM 3952 N VAL X 259 9.575 0.415 24.590 0.00 0.00 N

ATOM 3953 HN VAL X 259 9.842 1.309 24.943 0.00 0.00 H

ATOM 3954 CA VAL X 259 9.125 -0.441 25.727 0.00 0.00 C

ATOM 3955 HA VAL X 259 9.479 -1.459 25.672 0.00 0.00 H

ATOM 3956 CB VAL X 259 9.571 0.022 27.086 0.00 0.00 C

ATOM 3957 HB VAL X 259 8.966 0.911 27.333 0.00 0.00 H

ATOM 3958 CG1 VAL X 259 9.415 -1.149 28.114 0.00 0.00 C

ATOM 3959 HG11 VAL X 259 9.908 -2.099 27.797 0.00 0.00 H

ATOM 3960 HG12 VAL X 259 8.347 -1.422 28.227 0.00 0.00 H

ATOM 3961 HG13 VAL X 259 9.802 -0.907 29.128 0.00 0.00 H

ATOM 3962 CG2 VAL X 259 10.992 0.414 27.003 0.00 0.00 C

ATOM 3963 HG21 VAL X 259 11.419 0.598 28.015 0.00 0.00 H

ATOM 3964 HG22 VAL X 259 11.043 1.463 26.646 0.00 0.00 H

ATOM 3965 HG23 VAL X 259 11.590 -0.268 26.367 0.00 0.00 H

ATOM 3966 C VAL X 259 7.587 -0.494 25.764 0.00 0.00 C

ATOM 3967 O VAL X 259 6.924 0.551 25.844 0.00 0.00 O

ATOM 3968 N ARG X 260 7.040 -1.700 25.714 0.00 0.00 N

ATOM 3969 HN ARG X 260 7.589 -2.516 25.576 0.00 0.00 H

ATOM 3970 CA ARG X 260 5.586 -1.950 25.763 0.00 0.00 C

ATOM 3971 HA ARG X 260 5.063 -1.374 25.023 0.00 0.00 H

ATOM 3972 CB ARG X 260 5.122 -3.390 25.556 0.00 0.00 C

ATOM 3973 HB1 ARG X 260 4.014 -3.409 25.689 0.00 0.00 H

ATOM 3974 HB2 ARG X 260 5.552 -3.979 26.388 0.00 0.00 H

ATOM 3975 CG ARG X 260 5.529 -4.022 24.191 0.00 0.00 C

ATOM 3976 HG1 ARG X 260 6.617 -4.008 24.037 0.00 0.00 H

ATOM 3977 HG2 ARG X 260 5.068 -3.559 23.293 0.00 0.00 H

ATOM 3978 CD ARG X 260 5.153 -5.464 24.155 0.00 0.00 C

ATOM 3979 HD1 ARG X 260 4.055 -5.535 24.302 0.00 0.00 H

ATOM 3980 HD2 ARG X 260 5.611 -6.008 25.007 0.00 0.00 H

ATOM 3981 NE ARG X 260 5.487 -6.169 22.797 0.00 0.00 N

ATOM 3982 HE ARG X 260 5.929 -5.662 22.055 0.00 0.00 H

ATOM 3983 CZ ARG X 260 5.335 -7.447 22.603 0.00 0.00 C

ATOM 3984 NH1 ARG X 260 5.096 -8.242 23.597 0.00 0.00 N1+

ATOM 3985 HH11 ARG X 260 4.752 -7.833 24.450 0.00 0.00 H

ATOM 3986 HH12 ARG X 260 4.881 -9.190 23.385 0.00 0.00 H

ATOM 3987 NH2 ARG X 260 5.616 -7.848 21.329 0.00 0.00 N

ATOM 3988 HH21 ARG X 260 5.695 -8.794 21.028 0.00 0.00 H

ATOM 3989 HH22 ARG X 260 5.961 -7.090 20.772 0.00 0.00 H

ATOM 3990 C ARG X 260 5.098 -1.498 27.092 0.00 0.00 C

ATOM 3991 O ARG X 260 5.663 -1.803 28.101 0.00 0.00 O

ATOM 3992 N ASP X 261 3.968 -0.728 27.169 0.00 0.00 N

ATOM 3993 HN ASP X 261 3.589 -0.424 26.301 0.00 0.00 H

ATOM 3994 CA ASP X 261 3.144 -0.440 28.320 0.00 0.00 C

ATOM 3995 HA ASP X 261 2.490 0.345 27.965 0.00 0.00 H

ATOM 3996 CB ASP X 261 2.336 -1.630 28.775 0.00 0.00 C

ATOM 3997 HB1 ASP X 261 1.544 -1.425 29.522 0.00 0.00 H

ATOM 3998 HB2 ASP X 261 2.987 -2.404 29.220 0.00 0.00 H

ATOM 3999 CG ASP X 261 1.636 -2.190 27.496 0.00 0.00 C

ATOM 4000 OD1 ASP X 261 0.976 -1.460 26.690 0.00 0.00 O

ATOM 4001 OD2 ASP X 261 1.492 -3.461 27.494 0.00 0.00 O1-

ATOM 4002 C ASP X 261 3.810 0.323 29.481 0.00 0.00 C

ATOM 4003 O ASP X 261 3.675 0.066 30.714 0.00 0.00 O

ATOM 4004 N ALA X 262 4.542 1.423 29.095 0.00 0.00 N

ATOM 4005 HN ALA X 262 4.643 1.634 28.124 0.00 0.00 H

ATOM 4006 CA ALA X 262 5.267 2.230 30.018 0.00 0.00 C

ATOM 4007 HA ALA X 262 5.681 1.515 30.725 0.00 0.00 H

ATOM 4008 CB ALA X 262 6.307 3.097 29.348 0.00 0.00 C

ATOM 4009 HB1 ALA X 262 6.771 3.850 30.014 0.00 0.00 H

ATOM 4010 HB2 ALA X 262 5.764 3.653 28.556 0.00 0.00 H

ATOM 4011 HB3 ALA X 262 7.078 2.528 28.784 0.00 0.00 H

ATOM 4012 C ALA X 262 4.471 3.307 30.878 0.00 0.00 C

ATOM 4013 O ALA X 262 3.772 4.154 30.402 0.00 0.00 O

ATOM 4014 N THR X 263 4.559 3.268 32.180 0.00 0.00 N

ATOM 4015 HN THR X 263 5.084 2.602 32.705 0.00 0.00 H

ATOM 4016 CA THR X 263 4.006 4.265 33.122 0.00 0.00 C

ATOM 4017 HA THR X 263 3.021 4.569 32.813 0.00 0.00 H

ATOM 4018 CB THR X 263 3.847 3.842 34.585 0.00 0.00 C

ATOM 4019 HB THR X 263 3.578 4.758 35.150 0.00 0.00 H

ATOM 4020 OG1 THR X 263 4.950 3.147 35.084 0.00 0.00 O

ATOM 4021 HG1 THR X 263 5.398 3.851 35.560 0.00 0.00 H

ATOM 4022 CG2 THR X 263 2.622 2.959 34.721 0.00 0.00 C

ATOM 4023 HG21 THR X 263 2.523 2.526 35.737 0.00 0.00 H

ATOM 4024 HG22 THR X 263 2.643 2.060 34.078 0.00 0.00 H

ATOM 4025 HG23 THR X 263 1.769 3.621 34.443 0.00 0.00 H

ATOM 4026 C THR X 263 4.692 5.541 33.092 0.00 0.00 C

ATOM 4027 O THR X 263 4.053 6.573 33.307 0.00 0.00 O

ATOM 4028 N GLY X 264 6.022 5.556 32.732 0.00 0.00 N

ATOM 4029 HN GLY X 264 6.362 4.648 32.487 0.00 0.00 H

ATOM 4030 CA GLY X 264 6.773 6.816 32.620 0.00 0.00 C

ATOM 4031 HA1 GLY X 264 7.814 6.524 32.662 0.00 0.00 H

ATOM 4032 HA2 GLY X 264 6.550 7.440 33.481 0.00 0.00 H

ATOM 4033 C GLY X 264 6.380 7.671 31.431 0.00 0.00 C

ATOM 4034 O GLY X 264 6.939 8.731 31.150 0.00 0.00 O

ATOM 4035 N ASN X 265 5.509 7.153 30.494 0.00 0.00 N

ATOM 4036 HN ASN X 265 5.103 6.295 30.799 0.00 0.00 H

ATOM 4037 CA ASN X 265 4.997 7.963 29.376 0.00 0.00 C

ATOM 4038 HA ASN X 265 5.844 8.437 28.893 0.00 0.00 H

ATOM 4039 CB ASN X 265 4.237 6.935 28.504 0.00 0.00 C

ATOM 4040 HB1 ASN X 265 3.845 7.575 27.683 0.00 0.00 H

ATOM 4041 HB2 ASN X 265 3.483 6.368 29.085 0.00 0.00 H

ATOM 4042 CG ASN X 265 5.199 6.011 27.773 0.00 0.00 C

ATOM 4043 OD1 ASN X 265 6.438 6.079 27.713 0.00 0.00 O

ATOM 4044 ND2 ASN X 265 4.596 5.136 26.989 0.00 0.00 N

ATOM 4045 HD21 ASN X 265 3.622 5.021 27.183 0.00 0.00 H

ATOM 4046 HD22 ASN X 265 5.061 4.566 26.312 0.00 0.00 H

ATOM 4047 C ASN X 265 4.060 9.082 29.734 0.00 0.00 C

ATOM 4048 O ASN X 265 4.211 10.161 29.205 0.00 0.00 O

ATOM 4049 N VAL X 266 3.164 9.003 30.770 0.00 0.00 N

ATOM 4050 HN VAL X 266 3.012 8.177 31.302 0.00 0.00 H

ATOM 4051 CA VAL X 266 2.255 10.025 31.168 0.00 0.00 C

ATOM 4052 HA VAL X 266 2.156 10.813 30.429 0.00 0.00 H

ATOM 4053 CB VAL X 266 0.824 9.453 31.221 0.00 0.00 C

ATOM 4054 HB VAL X 266 0.143 10.299 31.433 0.00 0.00 H

ATOM 4055 CG1 VAL X 266 0.335 9.030 29.744 0.00 0.00 C

ATOM 4056 HG11 VAL X 266 -0.693 8.605 29.710 0.00 0.00 H

ATOM 4057 HG12 VAL X 266 0.901 8.107 29.524 0.00 0.00 H

ATOM 4058 HG13 VAL X 266 0.457 9.961 29.151 0.00 0.00 H

ATOM 4059 CG2 VAL X 266 0.662 8.347 32.224 0.00 0.00 C

ATOM 4060 HG21 VAL X 266 -0.349 7.887 32.286 0.00 0.00 H

ATOM 4061 HG22 VAL X 266 0.952 8.660 33.253 0.00 0.00 H

ATOM 4062 HG23 VAL X 266 1.319 7.510 31.928 0.00 0.00 H

ATOM 4063 C VAL X 266 2.592 10.796 32.484 0.00 0.00 C

ATOM 4064 O VAL X 266 3.345 10.285 33.286 0.00 0.00 O

ATOM 4065 N ASN X 267 2.131 12.076 32.581 0.00 0.00 N

ATOM 4066 HN ASN X 267 1.508 12.488 31.921 0.00 0.00 H

ATOM 4067 CA ASN X 267 2.442 12.994 33.693 0.00 0.00 C

ATOM 4068 HA ASN X 267 1.704 13.746 33.502 0.00 0.00 H

ATOM 4069 CB ASN X 267 2.283 12.402 35.066 0.00 0.00 C

ATOM 4070 HB1 ASN X 267 2.620 13.175 35.787 0.00 0.00 H

ATOM 4071 HB2 ASN X 267 2.941 11.510 35.156 0.00 0.00 H

ATOM 4072 CG ASN X 267 0.892 11.999 35.364 0.00 0.00 C

ATOM 4073 OD1 ASN X 267 -0.045 12.803 35.279 0.00 0.00 O

ATOM 4074 ND2 ASN X 267 0.632 10.818 36.039 0.00 0.00 N

ATOM 4075 HD21 ASN X 267 1.330 10.104 36.082 0.00 0.00 H

ATOM 4076 HD22 ASN X 267 -0.361 10.724 36.118 0.00 0.00 H

ATOM 4077 C ASN X 267 3.802 13.760 33.629 0.00 0.00 C

ATOM 4078 O ASN X 267 4.835 13.148 33.581 0.00 0.00 O

ATOM 4079 N ASP X 268 3.775 15.148 33.554 0.00 0.00 N

ATOM 4080 HN ASP X 268 2.964 15.689 33.329 0.00 0.00 H

ATOM 4081 CA ASP X 268 4.958 15.932 33.601 0.00 0.00 C

ATOM 4082 HA ASP X 268 5.613 15.593 32.808 0.00 0.00 H

ATOM 4083 CB ASP X 268 4.642 17.442 33.355 0.00 0.00 C

ATOM 4084 HB1 ASP X 268 5.570 18.047 33.427 0.00 0.00 H

ATOM 4085 HB2 ASP X 268 3.870 17.857 34.040 0.00 0.00 H

ATOM 4086 CG ASP X 268 4.128 17.547 31.972 0.00 0.00 C

ATOM 4087 OD1 ASP X 268 3.737 16.588 31.216 0.00 0.00 O

ATOM 4088 OD2 ASP X 268 4.060 18.704 31.393 0.00 0.00 O1-

ATOM 4089 C ASP X 268 5.919 15.748 34.888 0.00 0.00 C

ATOM 4090 O ASP X 268 5.500 15.404 35.994 0.00 0.00 O

ATOM 4091 N THR X 269 7.189 15.843 34.616 0.00 0.00 N

ATOM 4092 HN THR X 269 7.493 16.125 33.703 0.00 0.00 H

ATOM 4093 CA THR X 269 8.168 15.674 35.662 0.00 0.00 C

ATOM 4094 HA THR X 269 7.865 14.695 35.996 0.00 0.00 H

ATOM 4095 CB THR X 269 9.633 15.442 35.232 0.00 0.00 C

ATOM 4096 HB THR X 269 9.522 14.603 34.520 0.00 0.00 H

ATOM 4097 OG1 THR X 269 10.595 15.050 36.240 0.00 0.00 O

ATOM 4098 HG1 THR X 269 10.131 14.276 36.554 0.00 0.00 H

ATOM 4099 CG2 THR X 269 10.194 16.751 34.561 0.00 0.00 C

ATOM 4100 HG21 THR X 269 11.298 16.803 34.507 0.00 0.00 H

ATOM 4101 HG22 THR X 269 9.936 17.625 35.201 0.00 0.00 H

ATOM 4102 HG23 THR X 269 9.753 16.873 33.548 0.00 0.00 H

ATOM 4103 C THR X 269 8.028 16.622 36.884 0.00 0.00 C

ATOM 4104 O THR X 269 7.558 17.732 36.850 0.00 0.00 O

ATOM 4105 N ILE X 270 8.405 16.131 38.067 0.00 0.00 N

ATOM 4106 HN ILE X 270 8.806 15.215 38.091 0.00 0.00 H

ATOM 4107 CA ILE X 270 8.431 16.842 39.404 0.00 0.00 C

ATOM 4108 HA ILE X 270 8.077 17.859 39.277 0.00 0.00 H

ATOM 4109 CB ILE X 270 7.502 16.202 40.474 0.00 0.00 C

ATOM 4110 HB ILE X 270 7.538 16.792 41.407 0.00 0.00 H

ATOM 4111 CG2 ILE X 270 6.062 16.136 39.867 0.00 0.00 C

ATOM 4112 HG21 ILE X 270 5.792 15.121 39.512 0.00 0.00 H

ATOM 4113 HG22 ILE X 270 5.816 16.993 39.199 0.00 0.00 H

ATOM 4114 HG23 ILE X 270 5.444 16.157 40.797 0.00 0.00 H

ATOM 4115 CG1 ILE X 270 7.851 14.725 40.973 0.00 0.00 C

ATOM 4116 HG11 ILE X 270 8.942 14.812 41.147 0.00 0.00 H

ATOM 4117 HG12 ILE X 270 7.909 14.082 40.074 0.00 0.00 H

ATOM 4118 CD ILE X 270 7.064 14.149 42.175 0.00 0.00 C

ATOM 4119 HD1 ILE X 270 6.974 14.806 43.060 0.00 0.00 H

ATOM 4120 HD2 ILE X 270 7.564 13.188 42.435 0.00 0.00 H

ATOM 4121 HD3 ILE X 270 6.042 13.889 41.809 0.00 0.00 H

ATOM 4122 C ILE X 270 9.868 17.088 39.847 0.00 0.00 C

ATOM 4123 O ILE X 270 10.129 17.619 40.929 0.00 0.00 O

ATOM 4124 N VAL X 271 10.752 16.794 38.975 0.00 0.00 N

ATOM 4125 HN VAL X 271 10.543 16.307 38.127 0.00 0.00 H

ATOM 4126 CA VAL X 271 12.141 16.942 39.116 0.00 0.00 C

ATOM 4127 HA VAL X 271 12.395 17.076 40.164 0.00 0.00 H

ATOM 4128 CB VAL X 271 12.965 15.870 38.330 0.00 0.00 C

ATOM 4129 HB VAL X 271 12.689 16.022 37.271 0.00 0.00 H

ATOM 4130 CG1 VAL X 271 14.428 16.239 38.404 0.00 0.00 C

ATOM 4131 HG11 VAL X 271 14.789 16.686 39.362 0.00 0.00 H

ATOM 4132 HG12 VAL X 271 14.735 16.868 37.539 0.00 0.00 H

ATOM 4133 HG13 VAL X 271 15.084 15.348 38.505 0.00 0.00 H

ATOM 4134 CG2 VAL X 271 12.709 14.532 38.905 0.00 0.00 C

ATOM 4135 HG21 VAL X 271 11.659 14.186 38.753 0.00 0.00 H

ATOM 4136 HG22 VAL X 271 12.980 14.475 39.977 0.00 0.00 H

ATOM 4137 HG23 VAL X 271 13.327 13.773 38.398 0.00 0.00 H

ATOM 4138 C VAL X 271 12.322 18.323 38.520 0.00 0.00 C

ATOM 4139 O VAL X 271 11.984 18.628 37.374 0.00 0.00 O

ATOM 4140 N THR X 272 12.953 19.213 39.276 0.00 0.00 N

ATOM 4141 HN THR X 272 13.294 19.085 40.199 0.00 0.00 H

ATOM 4142 CA THR X 272 13.630 20.307 38.672 0.00 0.00 C

ATOM 4143 HA THR X 272 13.023 20.622 37.849 0.00 0.00 H

ATOM 4144 CB THR X 272 13.790 21.480 39.640 0.00 0.00 C

ATOM 4145 HB THR X 272 14.609 22.161 39.301 0.00 0.00 H

ATOM 4146 OG1 THR X 272 13.984 20.975 40.995 0.00 0.00 O

ATOM 4147 HG1 THR X 272 14.839 21.329 41.267 0.00 0.00 H

ATOM 4148 CG2 THR X 272 12.427 22.191 39.636 0.00 0.00 C

ATOM 4149 HG21 THR X 272 12.230 22.751 38.690 0.00 0.00 H

ATOM 4150 HG22 THR X 272 12.627 23.047 40.306 0.00 0.00 H

ATOM 4151 HG23 THR X 272 11.608 21.588 40.101 0.00 0.00 H

ATOM 4152 C THR X 272 15.012 19.881 38.206 0.00 0.00 C

ATOM 4153 O THR X 272 15.609 19.098 38.848 0.00 0.00 O

ATOM 4154 N GLU X 273 15.442 20.362 36.991 0.00 0.00 N

ATOM 4155 HN GLU X 273 14.925 21.048 36.486 0.00 0.00 H

ATOM 4156 CA GLU X 273 16.752 20.068 36.428 0.00 0.00 C

ATOM 4157 HA GLU X 273 16.922 20.652 35.541 0.00 0.00 H

ATOM 4158 CB GLU X 273 17.918 20.439 37.327 0.00 0.00 C

ATOM 4159 HB1 GLU X 273 18.890 20.395 36.781 0.00 0.00 H

ATOM 4160 HB2 GLU X 273 17.950 19.690 38.136 0.00 0.00 H

ATOM 4161 CG GLU X 273 17.833 21.970 37.781 0.00 0.00 C

ATOM 4162 HG1 GLU X 273 16.960 22.088 38.464 0.00 0.00 H

ATOM 4163 HG2 GLU X 273 17.796 22.527 36.822 0.00 0.00 H

ATOM 4164 CD GLU X 273 19.008 22.444 38.593 0.00 0.00 C

ATOM 4165 OE1 GLU X 273 20.146 21.948 38.528 0.00 0.00 O

ATOM 4166 OE2 GLU X 273 18.792 23.510 39.204 0.00 0.00 O1-

ATOM 4167 C GLU X 273 16.906 18.647 35.823 0.00 0.00 C

ATOM 4168 O GLU X 273 17.787 17.861 36.214 0.00 0.00 O

ATOM 4169 N LEU X 274 16.091 18.266 34.822 0.00 0.00 N

ATOM 4170 HN LEU X 274 15.322 18.840 34.549 0.00 0.00 H

ATOM 4171 CA LEU X 274 16.264 17.044 34.085 0.00 0.00 C

ATOM 4172 HA LEU X 274 17.306 16.787 33.934 0.00 0.00 H

ATOM 4173 CB LEU X 274 15.417 15.986 34.890 0.00 0.00 C

ATOM 4174 HB1 LEU X 274 14.472 16.436 35.261 0.00 0.00 H

ATOM 4175 HB2 LEU X 274 16.090 15.768 35.744 0.00 0.00 H

ATOM 4176 CG LEU X 274 15.145 14.708 34.139 0.00 0.00 C

ATOM 4177 HG LEU X 274 14.435 15.045 33.353 0.00 0.00 H

ATOM 4178 CD1 LEU X 274 16.401 14.219 33.471 0.00 0.00 C

ATOM 4179 HD11 LEU X 274 16.939 14.949 32.836 0.00 0.00 H

ATOM 4180 HD12 LEU X 274 16.135 13.328 32.866 0.00 0.00 H

ATOM 4181 HD13 LEU X 274 17.183 13.767 34.131 0.00 0.00 H

ATOM 4182 CD2 LEU X 274 14.665 13.632 35.055 0.00 0.00 C

ATOM 4183 HD21 LEU X 274 15.553 13.404 35.681 0.00 0.00 H

ATOM 4184 HD22 LEU X 274 14.337 12.761 34.462 0.00 0.00 H

ATOM 4185 HD23 LEU X 274 13.890 14.146 35.682 0.00 0.00 H

ATOM 4186 C LEU X 274 15.735 17.250 32.623 0.00 0.00 C

ATOM 4187 O LEU X 274 16.278 16.886 31.577 0.00 0.00 O

ATOM 4188 N THR X 275 14.575 17.980 32.468 0.00 0.00 N

ATOM 4189 HN THR X 275 14.069 18.367 33.244 0.00 0.00 H

ATOM 4190 CA THR X 275 13.943 18.337 31.196 0.00 0.00 C

ATOM 4191 HA THR X 275 14.785 18.752 30.665 0.00 0.00 H

ATOM 4192 CB THR X 275 13.479 17.152 30.387 0.00 0.00 C

ATOM 4193 HB THR X 275 14.349 16.519 30.101 0.00 0.00 H

ATOM 4194 OG1 THR X 275 12.791 17.516 29.169 0.00 0.00 O

ATOM 4195 HG1 THR X 275 12.763 16.835 28.495 0.00 0.00 H

ATOM 4196 CG2 THR X 275 12.487 16.278 31.123 0.00 0.00 C

ATOM 4197 HG21 THR X 275 13.014 15.726 31.933 0.00 0.00 H

ATOM 4198 HG22 THR X 275 12.006 15.515 30.466 0.00 0.00 H

ATOM 4199 HG23 THR X 275 11.700 16.958 31.516 0.00 0.00 H

ATOM 4200 C THR X 275 12.822 19.400 31.255 0.00 0.00 C

ATOM 4201 O THR X 275 11.973 19.340 32.114 0.00 0.00 O

ATOM 4202 N ASN X 276 12.844 20.350 30.317 0.00 0.00 N

ATOM 4203 HN ASN X 276 13.532 20.383 29.604 0.00 0.00 H

ATOM 4204 CA ASN X 276 11.875 21.387 30.153 0.00 0.00 C

ATOM 4205 HA ASN X 276 11.434 21.626 31.117 0.00 0.00 H

ATOM 4206 CB ASN X 276 12.469 22.615 29.440 0.00 0.00 C

ATOM 4207 HB1 ASN X 276 11.725 23.411 29.246 0.00 0.00 H

ATOM 4208 HB2 ASN X 276 12.983 22.319 28.498 0.00 0.00 H

ATOM 4209 CG ASN X 276 13.389 23.291 30.403 0.00 0.00 C

ATOM 4210 OD1 ASN X 276 13.022 23.380 31.587 0.00 0.00 O

ATOM 4211 ND2 ASN X 276 14.545 23.872 29.997 0.00 0.00 N

ATOM 4212 HD21 ASN X 276 14.762 23.786 29.028 0.00 0.00 H

ATOM 4213 HD22 ASN X 276 15.321 24.005 30.617 0.00 0.00 H

ATOM 4214 C ASN X 276 10.744 20.893 29.245 0.00 0.00 C

ATOM 4215 O ASN X 276 9.889 21.757 28.944 0.00 0.00 O

ATOM 4216 N CYS X 277 10.819 19.671 28.720 0.00 0.00 N

ATOM 4217 HN CYS X 277 11.578 19.155 29.109 0.00 0.00 H

ATOM 4218 CA CYS X 277 9.785 19.138 27.846 0.00 0.00 C

ATOM 4219 HA CYS X 277 9.299 19.878 27.230 0.00 0.00 H

ATOM 4220 CB CYS X 277 10.407 18.117 26.832 0.00 0.00 C

ATOM 4221 HB1 CYS X 277 10.756 17.150 27.257 0.00 0.00 H

ATOM 4222 HB2 CYS X 277 11.377 18.584 26.575 0.00 0.00 H

ATOM 4223 SG CYS X 277 9.481 17.910 25.172 0.00 0.00 S

ATOM 4224 HG1 CYS X 277 10.424 17.854 24.251 0.00 0.00 H

ATOM 4225 C CYS X 277 8.669 18.226 28.470 0.00 0.00 C

ATOM 4226 O CYS X 277 8.937 17.280 29.167 0.00 0.00 O

ATOM 4227 N THR X 278 7.407 18.616 28.196 0.00 0.00 N

ATOM 4228 HN THR X 278 7.313 19.387 27.570 0.00 0.00 H

ATOM 4229 CA THR X 278 6.044 17.947 28.616 0.00 0.00 C

ATOM 4230 HA THR X 278 6.120 17.857 29.688 0.00 0.00 H

ATOM 4231 CB THR X 278 4.914 18.827 28.273 0.00 0.00 C

ATOM 4232 HB THR X 278 5.064 19.809 28.778 0.00 0.00 H

ATOM 4233 OG1 THR X 278 3.664 18.304 28.766 0.00 0.00 O

ATOM 4234 HG1 THR X 278 3.642 18.373 29.716 0.00 0.00 H

ATOM 4235 CG2 THR X 278 4.679 19.088 26.748 0.00 0.00 C

ATOM 4236 HG21 THR X 278 4.030 18.302 26.296 0.00 0.00 H

ATOM 4237 HG22 THR X 278 5.604 18.920 26.154 0.00 0.00 H

ATOM 4238 HG23 THR X 278 4.209 20.083 26.590 0.00 0.00 H

ATOM 4239 C THR X 278 5.967 16.533 28.127 0.00 0.00 C

ATOM 4240 O THR X 278 6.245 16.231 26.977 0.00 0.00 O

ATOM 4241 N SER X 279 5.339 15.700 28.980 0.00 0.00 N

ATOM 4242 HN SER X 279 5.078 15.981 29.901 0.00 0.00 H

ATOM 4243 CA SER X 279 4.868 14.336 28.656 0.00 0.00 C

ATOM 4244 HA SER X 279 5.745 13.827 28.272 0.00 0.00 H

ATOM 4245 CB SER X 279 4.159 13.525 29.792 0.00 0.00 C

ATOM 4246 HB1 SER X 279 4.956 13.364 30.555 0.00 0.00 H

ATOM 4247 HB2 SER X 279 3.898 12.494 29.459 0.00 0.00 H

ATOM 4248 OG SER X 279 3.052 14.099 30.440 0.00 0.00 O

ATOM 4249 HG1 SER X 279 3.310 15.002 30.650 0.00 0.00 H

ATOM 4250 C SER X 279 3.803 14.370 27.570 0.00 0.00 C

ATOM 4251 O SER X 279 3.599 13.474 26.811 0.00 0.00 O

ATOM 4252 N ALA X 280 2.992 15.493 27.619 0.00 0.00 N

ATOM 4253 HN ALA X 280 3.234 16.279 28.188 0.00 0.00 H

ATOM 4254 CA ALA X 280 1.871 15.713 26.746 0.00 0.00 C

ATOM 4255 HA ALA X 280 1.189 14.898 26.947 0.00 0.00 H

ATOM 4256 CB ALA X 280 1.119 16.903 27.250 0.00 0.00 C

ATOM 4257 HB1 ALA X 280 0.973 16.764 28.342 0.00 0.00 H

ATOM 4258 HB2 ALA X 280 0.090 16.837 26.840 0.00 0.00 H

ATOM 4259 HB3 ALA X 280 1.433 17.859 26.788 0.00 0.00 H

ATOM 4260 C ALA X 280 2.278 15.821 25.311 0.00 0.00 C

ATOM 4261 O ALA X 280 3.351 16.265 25.006 0.00 0.00 O

ATOM 4262 N ALA X 281 1.449 15.310 24.398 0.00 0.00 N

ATOM 4263 HN ALA X 281 0.608 14.893 24.737 0.00 0.00 H

ATOM 4264 CA ALA X 281 1.599 15.495 22.920 0.00 0.00 C

ATOM 4265 HA ALA X 281 2.625 15.704 22.635 0.00 0.00 H

ATOM 4266 CB ALA X 281 1.340 14.195 22.212 0.00 0.00 C

ATOM 4267 HB1 ALA X 281 2.089 13.494 22.632 0.00 0.00 H

ATOM 4268 HB2 ALA X 281 1.453 14.311 21.108 0.00 0.00 H

ATOM 4269 HB3 ALA X 281 0.356 13.829 22.562 0.00 0.00 H

ATOM 4270 C ALA X 281 0.716 16.605 22.347 0.00 0.00 C

ATOM 4271 O ALA X 281 0.869 17.002 21.248 0.00 0.00 O

ATOM 4272 N CYS X 282 -0.218 17.155 23.206 0.00 0.00 N

ATOM 4273 HN CYS X 282 -0.291 16.748 24.113 0.00 0.00 H

ATOM 4274 CA CYS X 282 -0.987 18.345 23.013 0.00 0.00 C

ATOM 4275 HA CYS X 282 -1.247 18.401 21.963 0.00 0.00 H

ATOM 4276 CB CYS X 282 -2.230 18.525 23.914 0.00 0.00 C

ATOM 4277 HB1 CYS X 282 -1.938 18.565 24.982 0.00 0.00 H

ATOM 4278 HB2 CYS X 282 -2.732 17.550 23.753 0.00 0.00 H

ATOM 4279 SG CYS X 282 -3.162 19.987 23.413 0.00 0.00 S

ATOM 4280 HG1 CYS X 282 -2.290 20.647 24.150 0.00 0.00 H

ATOM 4281 C CYS X 282 -0.006 19.523 23.385 0.00 0.00 C

ATOM 4282 O CYS X 282 0.915 19.283 24.197 0.00 0.00 O

ATOM 4283 N LYS X 283 -0.021 20.603 22.606 0.00 0.00 N

ATOM 4284 HN LYS X 283 -0.671 20.706 21.853 0.00 0.00 H

ATOM 4285 CA LYS X 283 0.760 21.801 22.863 0.00 0.00 C

ATOM 4286 HA LYS X 283 1.744 21.377 22.747 0.00 0.00 H

ATOM 4287 CB LYS X 283 0.702 22.801 21.712 0.00 0.00 C

ATOM 4288 HB1 LYS X 283 1.006 23.822 22.030 0.00 0.00 H

ATOM 4289 HB2 LYS X 283 -0.355 22.947 21.395 0.00 0.00 H

ATOM 4290 CG LYS X 283 1.664 22.527 20.525 0.00 0.00 C

ATOM 4291 HG1 LYS X 283 1.393 23.238 19.712 0.00 0.00 H

ATOM 4292 HG2 LYS X 283 1.583 21.461 20.214 0.00 0.00 H

ATOM 4293 CD LYS X 283 3.122 22.787 20.878 0.00 0.00 C

ATOM 4294 HD1 LYS X 283 3.399 22.202 21.784 0.00 0.00 H

ATOM 4295 HD2 LYS X 283 3.253 23.860 21.093 0.00 0.00 H

ATOM 4296 CE LYS X 283 4.199 22.495 19.795 0.00 0.00 C

ATOM 4297 HE1 LYS X 283 5.214 22.847 20.064 0.00 0.00 H

ATOM 4298 HE2 LYS X 283 3.951 22.852 18.767 0.00 0.00 H

ATOM 4299 NZ LYS X 283 4.272 21.075 19.676 0.00 0.00 N1+

ATOM 4300 HZ1 LYS X 283 3.733 20.699 18.866 0.00 0.00 H

ATOM 4301 HZ2 LYS X 283 3.867 20.527 20.464 0.00 0.00 H

ATOM 4302 HZ3 LYS X 283 5.285 20.876 19.558 0.00 0.00 H

ATOM 4303 C LYS X 283 0.630 22.544 24.211 0.00 0.00 C

ATOM 4304 O LYS X 283 1.605 23.104 24.603 0.00 0.00 O

ATOM 4305 N LEU X 284 -0.569 22.626 24.902 0.00 0.00 N

ATOM 4306 HN LEU X 284 -1.360 22.229 24.449 0.00 0.00 H

ATOM 4307 CA LEU X 284 -0.809 23.287 26.193 0.00 0.00 C

ATOM 4308 HA LEU X 284 -1.824 23.078 26.509 0.00 0.00 H

ATOM 4309 CB LEU X 284 0.102 22.889 27.306 0.00 0.00 C

ATOM 4310 HB1 LEU X 284 -0.074 23.435 28.249 0.00 0.00 H

ATOM 4311 HB2 LEU X 284 1.141 23.100 26.972 0.00 0.00 H

ATOM 4312 CG LEU X 284 0.106 21.371 27.599 0.00 0.00 C

ATOM 4313 HG LEU X 284 0.553 20.790 26.759 0.00 0.00 H

ATOM 4314 CD1 LEU X 284 1.048 21.048 28.762 0.00 0.00 C

ATOM 4315 HD11 LEU X 284 2.081 21.381 28.550 0.00 0.00 H

ATOM 4316 HD12 LEU X 284 1.136 19.955 28.925 0.00 0.00 H

ATOM 4317 HD13 LEU X 284 0.667 21.448 29.722 0.00 0.00 H

ATOM 4318 CD2 LEU X 284 -1.295 20.798 27.767 0.00 0.00 C

ATOM 4319 HD21 LEU X 284 -1.192 19.739 28.100 0.00 0.00 H

ATOM 4320 HD22 LEU X 284 -1.957 20.861 26.874 0.00 0.00 H

ATOM 4321 HD23 LEU X 284 -1.642 21.313 28.693 0.00 0.00 H

ATOM 4322 C LEU X 284 -0.801 24.788 25.892 0.00 0.00 C

ATOM 4323 O LEU X 284 -0.946 25.250 24.777 0.00 0.00 O

ATOM 4324 N ASN X 285 -0.818 25.625 26.944 0.00 0.00 N

ATOM 4325 HN ASN X 285 -0.799 25.258 27.867 0.00 0.00 H

ATOM 4326 CA ASN X 285 -0.693 27.081 26.982 0.00 0.00 C

ATOM 4327 HA ASN X 285 -0.948 27.379 27.993 0.00 0.00 H

ATOM 4328 CB ASN X 285 0.764 27.543 26.535 0.00 0.00 C

ATOM 4329 HB1 ASN X 285 0.853 28.643 26.361 0.00 0.00 H

ATOM 4330 HB2 ASN X 285 0.964 27.035 25.573 0.00 0.00 H

ATOM 4331 CG ASN X 285 1.872 27.196 27.425 0.00 0.00 C

ATOM 4332 OD1 ASN X 285 2.916 26.654 27.041 0.00 0.00 O

ATOM 4333 ND2 ASN X 285 1.747 27.376 28.791 0.00 0.00 N

ATOM 4334 HD21 ASN X 285 2.553 27.117 29.330 0.00 0.00 H

ATOM 4335 HD22 ASN X 285 1.043 28.033 29.064 0.00 0.00 H

ATOM 4336 C ASN X 285 -1.761 27.734 26.187 0.00 0.00 C

ATOM 4337 O ASN X 285 -1.608 28.859 25.705 0.00 0.00 O

ATOM 4338 N PHE X 286 -2.943 27.186 26.265 0.00 0.00 N

ATOM 4339 HN PHE X 286 -3.018 26.368 26.826 0.00 0.00 H

ATOM 4340 CA PHE X 286 -4.112 27.744 25.617 0.00 0.00 C

ATOM 4341 HA PHE X 286 -3.861 28.230 24.685 0.00 0.00 H

ATOM 4342 CB PHE X 286 -5.237 26.684 25.302 0.00 0.00 C

ATOM 4343 HB1 PHE X 286 -5.952 27.230 24.654 0.00 0.00 H

ATOM 4344 HB2 PHE X 286 -5.763 26.389 26.232 0.00 0.00 H

ATOM 4345 CG PHE X 286 -4.753 25.511 24.465 0.00 0.00 C

ATOM 4346 CD1 PHE X 286 -3.951 25.760 23.286 0.00 0.00 C

ATOM 4347 HD1 PHE X 286 -3.876 26.759 22.885 0.00 0.00 H

ATOM 4348 CE1 PHE X 286 -3.443 24.667 22.557 0.00 0.00 C

ATOM 4349 HE1 PHE X 286 -2.888 24.862 21.647 0.00 0.00 H

ATOM 4350 CZ PHE X 286 -3.599 23.354 23.001 0.00 0.00 C

ATOM 4351 HZ PHE X 286 -3.157 22.581 22.374 0.00 0.00 H

ATOM 4352 CD2 PHE X 286 -4.998 24.171 24.890 0.00 0.00 C

ATOM 4353 HD2 PHE X 286 -5.500 24.054 25.842 0.00 0.00 H

ATOM 4354 CE2 PHE X 286 -4.361 23.133 24.202 0.00 0.00 C

ATOM 4355 HE2 PHE X 286 -4.420 22.145 24.604 0.00 0.00 H

ATOM 4356 C PHE X 286 -4.700 28.811 26.540 0.00 0.00 C

ATOM 4357 O PHE X 286 -4.383 29.002 27.691 0.00 0.00 O

ATOM 4358 N ASP X 287 -5.473 29.694 25.893 0.00 0.00 N

ATOM 4359 HN ASP X 287 -5.813 29.497 24.972 0.00 0.00 H

ATOM 4360 CA ASP X 287 -6.222 30.841 26.421 0.00 0.00 C

ATOM 4361 HA ASP X 287 -5.448 31.340 26.987 0.00 0.00 H

ATOM 4362 CB ASP X 287 -6.783 31.689 25.285 0.00 0.00 C

ATOM 4363 HB1 ASP X 287 -7.735 31.194 24.993 0.00 0.00 H

ATOM 4364 HB2 ASP X 287 -6.138 31.678 24.379 0.00 0.00 H

ATOM 4365 CG ASP X 287 -7.176 33.050 25.737 0.00 0.00 C

ATOM 4366 OD1 ASP X 287 -7.574 33.780 24.798 0.00 0.00 O

ATOM 4367 OD2 ASP X 287 -6.997 33.462 26.878 0.00 0.00 O1-

ATOM 4368 C ASP X 287 -7.240 30.390 27.449 0.00 0.00 C

ATOM 4369 O ASP X 287 -7.614 29.219 27.526 0.00 0.00 O

ATOM 4370 N PHE X 288 -7.710 31.302 28.345 0.00 0.00 N

ATOM 4371 HN PHE X 288 -7.409 32.221 28.115 0.00 0.00 H

ATOM 4372 CA PHE X 288 -8.782 31.051 29.243 0.00 0.00 C

ATOM 4373 HA PHE X 288 -8.425 30.281 29.908 0.00 0.00 H

ATOM 4374 CB PHE X 288 -9.117 32.263 30.164 0.00 0.00 C

ATOM 4375 HB1 PHE X 288 -10.015 32.102 30.793 0.00 0.00 H

ATOM 4376 HB2 PHE X 288 -9.387 33.071 29.446 0.00 0.00 H

ATOM 4377 CG PHE X 288 -7.948 32.668 31.041 0.00 0.00 C

ATOM 4378 CD1 PHE X 288 -7.367 33.901 30.851 0.00 0.00 C

ATOM 4379 HD1 PHE X 288 -7.517 34.476 29.960 0.00 0.00 H

ATOM 4380 CE1 PHE X 288 -6.383 34.401 31.722 0.00 0.00 C

ATOM 4381 HE1 PHE X 288 -5.966 35.377 31.521 0.00 0.00 H

ATOM 4382 CZ PHE X 288 -5.969 33.667 32.814 0.00 0.00 C

ATOM 4383 HZ PHE X 288 -5.273 33.995 33.574 0.00 0.00 H

ATOM 4384 CD2 PHE X 288 -7.660 32.006 32.205 0.00 0.00 C

ATOM 4385 HD2 PHE X 288 -8.215 31.135 32.515 0.00 0.00 H

ATOM 4386 CE2 PHE X 288 -6.590 32.540 33.083 0.00 0.00 C

ATOM 4387 HE2 PHE X 288 -6.512 31.929 33.969 0.00 0.00 H

ATOM 4388 C PHE X 288 -10.103 30.537 28.498 0.00 0.00 C

ATOM 4389 O PHE X 288 -10.513 30.908 27.390 0.00 0.00 O

ATOM 4390 N SER X 289 -10.672 29.582 29.144 0.00 0.00 N

ATOM 4391 HN SER X 289 -10.186 29.166 29.910 0.00 0.00 H

ATOM 4392 CA SER X 289 -11.967 28.902 28.988 0.00 0.00 C

ATOM 4393 HA SER X 289 -12.088 28.183 29.788 0.00 0.00 H

ATOM 4394 CB SER X 289 -13.148 29.896 29.014 0.00 0.00 C

ATOM 4395 HB1 SER X 289 -14.083 29.310 28.851 0.00 0.00 H

ATOM 4396 HB2 SER X 289 -13.006 30.573 28.148 0.00 0.00 H

ATOM 4397 OG SER X 289 -13.214 30.520 30.246 0.00 0.00 O

ATOM 4398 HG1 SER X 289 -12.859 29.802 30.776 0.00 0.00 H

ATOM 4399 C SER X 289 -11.904 28.033 27.752 0.00 0.00 C

ATOM 4400 O SER X 289 -12.895 27.723 27.059 0.00 0.00 O

ATOM 4401 N SER X 290 -10.741 27.588 27.323 0.00 0.00 N

ATOM 4402 HN SER X 290 -9.890 27.822 27.794 0.00 0.00 H

ATOM 4403 CA SER X 290 -10.558 26.933 26.086 0.00 0.00 C

ATOM 4404 HA SER X 290 -10.944 27.545 25.288 0.00 0.00 H

ATOM 4405 CB SER X 290 -9.007 26.701 25.848 0.00 0.00 C

ATOM 4406 HB1 SER X 290 -8.868 25.807 25.196 0.00 0.00 H

ATOM 4407 HB2 SER X 290 -8.567 26.579 26.862 0.00 0.00 H

ATOM 4408 OG SER X 290 -8.289 27.859 25.254 0.00 0.00 O

ATOM 4409 HG1 SER X 290 -8.277 28.605 25.861 0.00 0.00 H

ATOM 4410 C SER X 290 -11.189 25.569 26.007 0.00 0.00 C

ATOM 4411 O SER X 290 -11.308 24.890 27.088 0.00 0.00 O

ATOM 4412 N CYS X 291 -11.538 25.129 24.779 0.00 0.00 N

ATOM 4413 HN CYS X 291 -11.480 25.642 23.924 0.00 0.00 H

ATOM 4414 CA CYS X 291 -11.964 23.749 24.471 0.00 0.00 C

ATOM 4415 HA CYS X 291 -12.662 23.372 25.196 0.00 0.00 H

ATOM 4416 CB CYS X 291 -12.551 23.644 23.053 0.00 0.00 C

ATOM 4417 HB1 CYS X 291 -11.922 23.993 22.201 0.00 0.00 H

ATOM 4418 HB2 CYS X 291 -13.413 24.344 23.084 0.00 0.00 H

ATOM 4419 SG CYS X 291 -13.295 21.997 22.617 0.00 0.00 S

ATOM 4420 HG1 CYS X 291 -14.258 22.674 22.013 0.00 0.00 H

ATOM 4421 C CYS X 291 -10.795 22.887 24.585 0.00 0.00 C

ATOM 4422 O CYS X 291 -9.794 23.241 23.895 0.00 0.00 O

ATOM 4423 N GLU X 292 -10.825 21.775 25.261 0.00 0.00 N

ATOM 4424 HN GLU X 292 -11.657 21.530 25.751 0.00 0.00 H

ATOM 4425 CA GLU X 292 -9.664 20.902 25.425 0.00 0.00 C

ATOM 4426 HA GLU X 292 -8.877 21.347 24.846 0.00 0.00 H

ATOM 4427 CB GLU X 292 -9.293 20.745 26.948 0.00 0.00 C

ATOM 4428 HB1 GLU X 292 -8.379 20.119 27.029 0.00 0.00 H

ATOM 4429 HB2 GLU X 292 -10.167 20.224 27.401 0.00 0.00 H

ATOM 4430 CG GLU X 292 -8.932 22.136 27.560 0.00 0.00 C

ATOM 4431 HG1 GLU X 292 -9.912 22.656 27.560 0.00 0.00 H

ATOM 4432 HG2 GLU X 292 -8.037 22.609 27.102 0.00 0.00 H

ATOM 4433 CD GLU X 292 -8.488 21.937 28.963 0.00 0.00 C

ATOM 4434 OE1 GLU X 292 -7.433 21.309 29.130 0.00 0.00 O

ATOM 4435 OE2 GLU X 292 -9.173 22.397 29.896 0.00 0.00 O1-

ATOM 4436 C GLU X 292 -9.965 19.579 24.835 0.00 0.00 C

ATOM 4437 O GLU X 292 -10.919 18.877 25.241 0.00 0.00 O

ATOM 4438 N SER X 293 -9.206 19.139 23.868 0.00 0.00 N

ATOM 4439 HN SER X 293 -8.575 19.844 23.558 0.00 0.00 H

ATOM 4440 CA SER X 293 -9.193 17.899 23.155 0.00 0.00 C

ATOM 4441 HA SER X 293 -9.744 17.192 23.757 0.00 0.00 H

ATOM 4442 CB SER X 293 -9.803 18.028 21.699 0.00 0.00 C

ATOM 4443 HB1 SER X 293 -9.100 18.505 20.978 0.00 0.00 H

ATOM 4444 HB2 SER X 293 -10.840 18.426 21.620 0.00 0.00 H

ATOM 4445 OG SER X 293 -9.873 16.647 21.330 0.00 0.00 O

ATOM 4446 HG1 SER X 293 -10.777 16.348 21.432 0.00 0.00 H

ATOM 4447 C SER X 293 -7.738 17.465 23.151 0.00 0.00 C

ATOM 4448 O SER X 293 -6.900 18.322 23.287 0.00 0.00 O

ATOM 4449 N SER X 294 -7.370 16.187 23.156 0.00 0.00 N

ATOM 4450 HN SER X 294 -8.095 15.549 22.917 0.00 0.00 H

ATOM 4451 CA SER X 294 -5.981 15.781 23.314 0.00 0.00 C

ATOM 4452 HA SER X 294 -5.430 16.686 23.092 0.00 0.00 H

ATOM 4453 CB SER X 294 -5.680 15.395 24.754 0.00 0.00 C

ATOM 4454 HB1 SER X 294 -6.270 14.565 25.178 0.00 0.00 H

ATOM 4455 HB2 SER X 294 -5.936 16.186 25.490 0.00 0.00 H

ATOM 4456 OG SER X 294 -4.249 15.269 24.898 0.00 0.00 O

ATOM 4457 HG1 SER X 294 -3.996 14.954 25.773 0.00 0.00 H

ATOM 4458 C SER X 294 -5.667 14.632 22.426 0.00 0.00 C

ATOM 4459 O SER X 294 -6.607 13.960 22.106 0.00 0.00 O

ATOM 4460 N PRO X 295 -4.490 14.281 21.913 0.00 0.00 N

ATOM 4461 CD PRO X 295 -3.455 15.306 21.534 0.00 0.00 C

ATOM 4462 HD1 PRO X 295 -2.846 15.464 22.447 0.00 0.00 H

ATOM 4463 HD2 PRO X 295 -3.872 16.303 21.302 0.00 0.00 H

ATOM 4464 CA PRO X 295 -4.366 13.153 20.953 0.00 0.00 C

ATOM 4465 HA PRO X 295 -5.022 13.238 20.099 0.00 0.00 H

ATOM 4466 CB PRO X 295 -2.894 13.159 20.548 0.00 0.00 C

ATOM 4467 HB1 PRO X 295 -2.797 12.606 19.588 0.00 0.00 H

ATOM 4468 HB2 PRO X 295 -2.193 12.868 21.360 0.00 0.00 H

ATOM 4469 CG PRO X 295 -2.638 14.638 20.377 0.00 0.00 C

ATOM 4470 HG1 PRO X 295 -1.539 14.755 20.298 0.00 0.00 H

ATOM 4471 HG2 PRO X 295 -3.049 15.015 19.410 0.00 0.00 H

ATOM 4472 C PRO X 295 -4.782 11.797 21.552 0.00 0.00 C

ATOM 4473 O PRO X 295 -4.537 11.571 22.753 0.00 0.00 O

ATOM 4474 N CYS X 296 -5.246 10.869 20.676 0.00 0.00 N

ATOM 4475 HN CYS X 296 -5.353 11.100 19.721 0.00 0.00 H

ATOM 4476 CA CYS X 296 -5.640 9.480 21.108 0.00 0.00 C

ATOM 4477 HA CYS X 296 -6.554 9.435 21.687 0.00 0.00 H

ATOM 4478 CB CYS X 296 -5.997 8.813 19.762 0.00 0.00 C

ATOM 4479 HB1 CYS X 296 -5.966 7.716 19.909 0.00 0.00 H

ATOM 4480 HB2 CYS X 296 -5.407 9.132 18.878 0.00 0.00 H

ATOM 4481 SG CYS X 296 -7.702 9.392 19.316 0.00 0.00 S

ATOM 4482 HG1 CYS X 296 -7.386 10.299 18.403 0.00 0.00 H

ATOM 4483 C CYS X 296 -4.531 8.689 21.755 0.00 0.00 C

ATOM 4484 O CYS X 296 -4.878 8.037 22.739 0.00 0.00 O

ATOM 4485 N SER X 297 -3.307 8.733 21.251 0.00 0.00 N

ATOM 4486 HN SER X 297 -3.110 9.338 20.476 0.00 0.00 H

ATOM 4487 CA SER X 297 -2.163 7.989 21.808 0.00 0.00 C

ATOM 4488 HA SER X 297 -2.428 6.947 21.858 0.00 0.00 H

ATOM 4489 CB SER X 297 -0.866 7.929 20.895 0.00 0.00 C

ATOM 4490 HB1 SER X 297 -0.808 8.968 20.520 0.00 0.00 H

ATOM 4491 HB2 SER X 297 -1.017 7.173 20.102 0.00 0.00 H

ATOM 4492 OG SER X 297 0.343 7.600 21.523 0.00 0.00 O

ATOM 4493 HG1 SER X 297 0.596 8.374 22.043 0.00 0.00 H

ATOM 4494 C SER X 297 -1.838 8.321 23.243 0.00 0.00 C

ATOM 4495 O SER X 297 -1.701 7.367 23.997 0.00 0.00 O

ATOM 4496 N TYR X 298 -1.811 9.592 23.726 0.00 0.00 N

ATOM 4497 HN TYR X 298 -1.818 10.231 22.957 0.00 0.00 H

ATOM 4498 CA TYR X 298 -1.575 10.113 25.119 0.00 0.00 C

ATOM 4499 HA TYR X 298 -0.567 9.764 25.343 0.00 0.00 H

ATOM 4500 CB TYR X 298 -1.493 11.645 25.036 0.00 0.00 C

ATOM 4501 HB1 TYR X 298 -2.462 12.121 24.757 0.00 0.00 H

ATOM 4502 HB2 TYR X 298 -0.809 12.014 24.239 0.00 0.00 H

ATOM 4503 CG TYR X 298 -1.069 12.104 26.393 0.00 0.00 C

ATOM 4504 CD1 TYR X 298 -1.957 12.535 27.396 0.00 0.00 C

ATOM 4505 HD1 TYR X 298 -3.002 12.690 27.171 0.00 0.00 H

ATOM 4506 CE1 TYR X 298 -1.479 12.903 28.704 0.00 0.00 C

ATOM 4507 HE1 TYR X 298 -2.213 13.147 29.455 0.00 0.00 H

ATOM 4508 CZ TYR X 298 -0.133 12.963 28.938 0.00 0.00 C

ATOM 4509 OH TYR X 298 0.368 13.501 30.146 0.00 0.00 O

ATOM 4510 HH TYR X 298 1.300 13.703 30.083 0.00 0.00 H

ATOM 4511 CD2 TYR X 298 0.287 12.056 26.675 0.00 0.00 C

ATOM 4512 HD2 TYR X 298 1.039 11.745 25.961 0.00 0.00 H

ATOM 4513 CE2 TYR X 298 0.727 12.486 27.914 0.00 0.00 C

ATOM 4514 HE2 TYR X 298 1.804 12.549 27.987 0.00 0.00 H

ATOM 4515 C TYR X 298 -2.560 9.597 26.134 0.00 0.00 C

ATOM 4516 O TYR X 298 -2.157 9.075 27.231 0.00 0.00 O

ATOM 4517 N GLY X 299 -3.883 9.635 25.796 0.00 0.00 N

ATOM 4518 HN GLY X 299 -4.167 9.823 24.855 0.00 0.00 H

ATOM 4519 CA GLY X 299 -4.922 9.123 26.664 0.00 0.00 C

ATOM 4520 HA1 GLY X 299 -5.930 9.339 26.348 0.00 0.00 H

ATOM 4521 HA2 GLY X 299 -4.730 9.476 27.670 0.00 0.00 H

ATOM 4522 C GLY X 299 -4.751 7.644 26.781 0.00 0.00 C

ATOM 4523 O GLY X 299 -4.969 7.011 27.797 0.00 0.00 O

ATOM 4524 N LEU X 300 -4.293 6.922 25.750 0.00 0.00 N

ATOM 4525 HN LEU X 300 -3.955 7.359 24.921 0.00 0.00 H

ATOM 4526 CA LEU X 300 -3.977 5.490 25.940 0.00 0.00 C

ATOM 4527 HA LEU X 300 -4.607 4.976 26.661 0.00 0.00 H

ATOM 4528 CB LEU X 300 -4.114 4.810 24.498 0.00 0.00 C

ATOM 4529 HB1 LEU X 300 -3.702 3.779 24.420 0.00 0.00 H

ATOM 4530 HB2 LEU X 300 -3.475 5.380 23.784 0.00 0.00 H

ATOM 4531 CG LEU X 300 -5.527 4.740 23.895 0.00 0.00 C

ATOM 4532 HG LEU X 300 -5.993 5.740 24.010 0.00 0.00 H

ATOM 4533 CD1 LEU X 300 -5.449 4.370 22.426 0.00 0.00 C

ATOM 4534 HD11 LEU X 300 -4.723 4.994 21.869 0.00 0.00 H

ATOM 4535 HD12 LEU X 300 -6.486 4.242 22.049 0.00 0.00 H

ATOM 4536 HD13 LEU X 300 -5.095 3.320 22.322 0.00 0.00 H

ATOM 4537 CD2 LEU X 300 -6.444 3.787 24.665 0.00 0.00 C

ATOM 4538 HD21 LEU X 300 -7.331 3.399 24.130 0.00 0.00 H

ATOM 4539 HD22 LEU X 300 -7.009 4.240 25.500 0.00 0.00 H

ATOM 4540 HD23 LEU X 300 -5.862 2.956 25.116 0.00 0.00 H

ATOM 4541 C LEU X 300 -2.558 5.232 26.505 0.00 0.00 C

ATOM 4542 O LEU X 300 -2.108 4.056 26.420 0.00 0.00 O

ATOM 4543 N MET X 301 -1.769 6.232 27.029 0.00 0.00 N

ATOM 4544 HN MET X 301 -2.205 7.132 26.982 0.00 0.00 H

ATOM 4545 CA MET X 301 -0.473 6.091 27.724 0.00 0.00 C

ATOM 4546 HA MET X 301 -0.425 7.023 28.272 0.00 0.00 H

ATOM 4547 CB MET X 301 -0.511 4.982 28.892 0.00 0.00 C

ATOM 4548 HB1 MET X 301 0.452 5.060 29.446 0.00 0.00 H

ATOM 4549 HB2 MET X 301 -0.810 4.018 28.440 0.00 0.00 H

ATOM 4550 CG MET X 301 -1.460 5.314 29.996 0.00 0.00 C

ATOM 4551 HG1 MET X 301 -2.486 5.298 29.583 0.00 0.00 H

ATOM 4552 HG2 MET X 301 -1.300 6.371 30.307 0.00 0.00 H

ATOM 4553 SD MET X 301 -1.461 4.072 31.430 0.00 0.00 S

ATOM 4554 CE MET X 301 -0.093 4.488 32.469 0.00 0.00 C

ATOM 4555 HE1 MET X 301 -0.046 3.767 33.318 0.00 0.00 H

ATOM 4556 HE2 MET X 301 -0.247 5.543 32.785 0.00 0.00 H

ATOM 4557 HE3 MET X 301 0.853 4.520 31.894 0.00 0.00 H

ATOM 4558 C MET X 301 0.653 5.937 26.869 0.00 0.00 C

ATOM 4559 O MET X 301 1.668 5.348 27.241 0.00 0.00 O

ATOM 4560 N ASN X 302 0.557 6.464 25.634 0.00 0.00 N

ATOM 4561 HN ASN X 302 -0.246 7.011 25.419 0.00 0.00 H

ATOM 4562 CA ASN X 302 1.377 6.195 24.552 0.00 0.00 C

ATOM 4563 HA ASN X 302 0.785 6.316 23.652 0.00 0.00 H

ATOM 4564 CB ASN X 302 2.618 7.254 24.385 0.00 0.00 C

ATOM 4565 HB1 ASN X 302 3.183 6.970 23.467 0.00 0.00 H

ATOM 4566 HB2 ASN X 302 3.302 7.250 25.256 0.00 0.00 H

ATOM 4567 CG ASN X 302 2.147 8.687 24.158 0.00 0.00 C

ATOM 4568 OD1 ASN X 302 1.644 8.993 23.115 0.00 0.00 O

ATOM 4569 ND2 ASN X 302 2.438 9.580 25.088 0.00 0.00 N

ATOM 4570 HD21 ASN X 302 2.434 10.543 24.812 0.00 0.00 H

ATOM 4571 HD22 ASN X 302 3.003 9.314 25.863 0.00 0.00 H

ATOM 4572 C ASN X 302 1.802 4.693 24.335 0.00 0.00 C

ATOM 4573 O ASN X 302 2.970 4.338 24.183 0.00 0.00 O

ATOM 4574 N ASN X 303 0.836 3.899 24.212 0.00 0.00 N

ATOM 4575 HN ASN X 303 -0.070 4.152 24.550 0.00 0.00 H

ATOM 4576 CA ASN X 303 1.210 2.482 23.893 0.00 0.00 C

ATOM 4577 HA ASN X 303 1.998 2.234 24.590 0.00 0.00 H

ATOM 4578 CB ASN X 303 -0.099 1.595 24.061 0.00 0.00 C

ATOM 4579 HB1 ASN X 303 -0.832 2.199 23.477 0.00 0.00 H

ATOM 4580 HB2 ASN X 303 -0.490 1.503 25.093 0.00 0.00 H

ATOM 4581 CG ASN X 303 -0.129 0.211 23.474 0.00 0.00 C

ATOM 4582 OD1 ASN X 303 0.713 -0.475 22.855 0.00 0.00 O

ATOM 4583 ND2 ASN X 303 -1.396 -0.293 23.672 0.00 0.00 N

ATOM 4584 HD21 ASN X 303 -2.103 0.244 24.133 0.00 0.00 H

ATOM 4585 HD22 ASN X 303 -1.688 -1.122 23.201 0.00 0.00 H

ATOM 4586 C ASN X 303 2.001 2.285 22.510 0.00 0.00 C

ATOM 4587 O ASN X 303 1.827 3.078 21.562 0.00 0.00 O

ATOM 4588 N PHE X 304 2.828 1.172 22.463 0.00 0.00 N

ATOM 4589 HN PHE X 304 2.799 0.519 23.209 0.00 0.00 H

ATOM 4590 CA PHE X 304 3.764 0.776 21.482 0.00 0.00 C

ATOM 4591 HA PHE X 304 4.622 1.425 21.422 0.00 0.00 H

ATOM 4592 CB PHE X 304 4.516 -0.513 21.831 0.00 0.00 C

ATOM 4593 HB1 PHE X 304 3.704 -1.228 22.062 0.00 0.00 H

ATOM 4594 HB2 PHE X 304 5.030 -0.383 22.816 0.00 0.00 H

ATOM 4595 CG PHE X 304 5.485 -1.046 20.833 0.00 0.00 C

ATOM 4596 CD1 PHE X 304 6.857 -1.103 21.148 0.00 0.00 C

ATOM 4597 HD1 PHE X 304 7.201 -0.774 22.121 0.00 0.00 H

ATOM 4598 CE1 PHE X 304 7.843 -1.425 20.108 0.00 0.00 C

ATOM 4599 HE1 PHE X 304 8.909 -1.278 20.224 0.00 0.00 H

ATOM 4600 CZ PHE X 304 7.425 -1.872 18.902 0.00 0.00 C

ATOM 4601 HZ PHE X 304 8.142 -2.164 18.143 0.00 0.00 H

ATOM 4602 CD2 PHE X 304 5.085 -1.514 19.560 0.00 0.00 C

ATOM 4603 HD2 PHE X 304 4.051 -1.510 19.246 0.00 0.00 H

ATOM 4604 CE2 PHE X 304 6.041 -1.885 18.570 0.00 0.00 C

ATOM 4605 HE2 PHE X 304 5.712 -2.274 17.613 0.00 0.00 H

ATOM 4606 C PHE X 304 3.047 0.734 20.096 0.00 0.00 C

ATOM 4607 O PHE X 304 3.646 1.105 19.099 0.00 0.00 O

ATOM 4608 N GLN X 305 1.815 0.282 20.143 0.00 0.00 N

ATOM 4609 HN GLN X 305 1.547 -0.102 21.016 0.00 0.00 H

ATOM 4610 CA GLN X 305 0.971 0.030 18.989 0.00 0.00 C

ATOM 4611 HA GLN X 305 1.568 -0.292 18.158 0.00 0.00 H

ATOM 4612 CB GLN X 305 -0.089 -1.017 19.417 0.00 0.00 C

ATOM 4613 HB1 GLN X 305 -0.844 -1.184 18.619 0.00 0.00 H

ATOM 4614 HB2 GLN X 305 -0.684 -0.686 20.297 0.00 0.00 H

ATOM 4615 CG GLN X 305 0.579 -2.289 19.765 0.00 0.00 C

ATOM 4616 HG1 GLN X 305 0.933 -2.161 20.814 0.00 0.00 H

ATOM 4617 HG2 GLN X 305 1.413 -2.487 19.043 0.00 0.00 H

ATOM 4618 CD GLN X 305 -0.439 -3.408 19.920 0.00 0.00 C

ATOM 4619 OE1 GLN X 305 -1.608 -3.221 20.345 0.00 0.00 O

ATOM 4620 NE2 GLN X 305 0.053 -4.623 19.782 0.00 0.00 N

ATOM 4621 HE21 GLN X 305 -0.585 -5.376 19.954 0.00 0.00 H

ATOM 4622 HE22 GLN X 305 1.032 -4.732 19.614 0.00 0.00 H

ATOM 4623 C GLN X 305 0.248 1.270 18.495 0.00 0.00 C

ATOM 4624 O GLN X 305 -0.405 1.209 17.450 0.00 0.00 O

ATOM 4625 N VAL X 306 0.251 2.388 19.298 0.00 0.00 N

ATOM 4626 HN VAL X 306 0.857 2.526 20.081 0.00 0.00 H

ATOM 4627 CA VAL X 306 -0.414 3.586 18.743 0.00 0.00 C

ATOM 4628 HA VAL X 306 -0.787 3.500 17.740 0.00 0.00 H

ATOM 4629 CB VAL X 306 -1.666 3.960 19.610 0.00 0.00 C

ATOM 4630 HB VAL X 306 -2.065 4.940 19.259 0.00 0.00 H

ATOM 4631 CG1 VAL X 306 -2.679 2.755 19.458 0.00 0.00 C

ATOM 4632 HG11 VAL X 306 -3.271 2.701 18.520 0.00 0.00 H

ATOM 4633 HG12 VAL X 306 -3.403 2.907 20.286 0.00 0.00 H

ATOM 4634 HG13 VAL X 306 -2.150 1.782 19.580 0.00 0.00 H

ATOM 4635 CG2 VAL X 306 -1.241 4.250 21.048 0.00 0.00 C

ATOM 4636 HG21 VAL X 306 -0.683 5.214 21.035 0.00 0.00 H

ATOM 4637 HG22 VAL X 306 -0.631 3.402 21.413 0.00 0.00 H

ATOM 4638 HG23 VAL X 306 -2.074 4.412 21.763 0.00 0.00 H

ATOM 4639 C VAL X 306 0.466 4.777 18.649 0.00 0.00 C

ATOM 4640 O VAL X 306 -0.010 5.811 18.186 0.00 0.00 O

ATOM 4641 N MET X 307 1.670 4.714 19.208 0.00 0.00 N

ATOM 4642 HN MET X 307 1.976 3.926 19.727 0.00 0.00 H

ATOM 4643 CA MET X 307 2.701 5.660 18.975 0.00 0.00 C

ATOM 4644 HA MET X 307 2.115 6.532 18.742 0.00 0.00 H

ATOM 4645 CB MET X 307 3.635 5.887 20.121 0.00 0.00 C

ATOM 4646 HB1 MET X 307 3.015 5.893 21.051 0.00 0.00 H

ATOM 4647 HB2 MET X 307 4.167 6.849 19.964 0.00 0.00 H

ATOM 4648 CG MET X 307 4.673 4.749 20.451 0.00 0.00 C

ATOM 4649 HG1 MET X 307 4.223 3.745 20.347 0.00 0.00 H

ATOM 4650 HG2 MET X 307 4.959 4.834 21.514 0.00 0.00 H

ATOM 4651 SD MET X 307 6.226 4.743 19.481 0.00 0.00 S

ATOM 4652 CE MET X 307 6.129 3.018 19.031 0.00 0.00 C

ATOM 4653 HE1 MET X 307 7.051 2.837 18.438 0.00 0.00 H

ATOM 4654 HE2 MET X 307 5.189 2.890 18.443 0.00 0.00 H

ATOM 4655 HE3 MET X 307 6.261 2.330 19.887 0.00 0.00 H

ATOM 4656 C MET X 307 3.467 5.472 17.690 0.00 0.00 C

ATOM 4657 O MET X 307 3.541 4.353 17.208 0.00 0.00 O

ATOM 4658 N SER X 308 4.093 6.575 17.222 0.00 0.00 N

ATOM 4659 HN SER X 308 4.159 7.470 17.683 0.00 0.00 H

ATOM 4660 CA SER X 308 5.020 6.543 16.115 0.00 0.00 C

ATOM 4661 HA SER X 308 5.357 5.519 16.008 0.00 0.00 H

ATOM 4662 CB SER X 308 4.403 7.077 14.823 0.00 0.00 C

ATOM 4663 HB1 SER X 308 4.237 8.170 14.944 0.00 0.00 H

ATOM 4664 HB2 SER X 308 3.395 6.610 14.734 0.00 0.00 H

ATOM 4665 OG SER X 308 5.144 6.811 13.621 0.00 0.00 O

ATOM 4666 HG1 SER X 308 4.800 7.451 12.996 0.00 0.00 H

ATOM 4667 C SER X 308 6.214 7.415 16.389 0.00 0.00 C

ATOM 4668 O SER X 308 6.062 8.532 16.974 0.00 0.00 O

ATOM 4669 N MET X 309 7.373 6.895 16.056 0.00 0.00 N

ATOM 4670 HN MET X 309 7.376 5.956 15.726 0.00 0.00 H

ATOM 4671 CA MET X 309 8.677 7.539 16.256 0.00 0.00 C

ATOM 4672 HA MET X 309 8.775 8.248 17.057 0.00 0.00 H

ATOM 4673 CB MET X 309 9.704 6.449 16.451 0.00 0.00 C

ATOM 4674 HB1 MET X 309 10.783 6.721 16.369 0.00 0.00 H

ATOM 4675 HB2 MET X 309 9.452 5.637 15.741 0.00 0.00 H

ATOM 4676 CG MET X 309 9.656 5.773 17.912 0.00 0.00 C

ATOM 4677 HG1 MET X 309 10.467 5.026 18.050 0.00 0.00 H

ATOM 4678 HG2 MET X 309 8.698 5.227 18.066 0.00 0.00 H

ATOM 4679 SD MET X 309 9.778 7.154 19.164 0.00 0.00 S

ATOM 4680 CE MET X 309 9.294 6.268 20.602 0.00 0.00 C

ATOM 4681 HE1 MET X 309 9.688 6.800 21.487 0.00 0.00 H

ATOM 4682 HE2 MET X 309 9.763 5.261 20.508 0.00 0.00 H

ATOM 4683 HE3 MET X 309 8.216 6.336 20.863 0.00 0.00 H

ATOM 4684 C MET X 309 9.045 8.230 14.930 0.00 0.00 C

ATOM 4685 O MET X 309 10.146 8.813 14.800 0.00 0.00 O

ATOM 4686 N VAL X 310 8.185 8.255 13.937 0.00 0.00 N

ATOM 4687 HN VAL X 310 7.322 7.768 13.875 0.00 0.00 H

ATOM 4688 CA VAL X 310 8.644 8.847 12.597 0.00 0.00 C

ATOM 4689 HA VAL X 310 9.682 8.577 12.456 0.00 0.00 H

ATOM 4690 CB VAL X 310 7.762 8.458 11.418 0.00 0.00 C

ATOM 4691 HB VAL X 310 6.705 8.739 11.556 0.00 0.00 H

ATOM 4692 CG1 VAL X 310 8.328 9.015 10.102 0.00 0.00 C

ATOM 4693 HG11 VAL X 310 8.022 10.072 9.948 0.00 0.00 H

ATOM 4694 HG12 VAL X 310 7.934 8.505 9.195 0.00 0.00 H

ATOM 4695 HG13 VAL X 310 9.404 8.754 10.025 0.00 0.00 H

ATOM 4696 CG2 VAL X 310 7.902 6.959 11.281 0.00 0.00 C

ATOM 4697 HG21 VAL X 310 8.895 6.534 11.034 0.00 0.00 H

ATOM 4698 HG22 VAL X 310 7.317 6.603 10.404 0.00 0.00 H

ATOM 4699 HG23 VAL X 310 7.443 6.447 12.152 0.00 0.00 H

ATOM 4700 C VAL X 310 8.801 10.405 12.773 0.00 0.00 C

ATOM 4701 O VAL X 310 7.949 11.100 13.249 0.00 0.00 O

ATOM 4702 N SER X 311 9.922 10.948 12.174 0.00 0.00 N

ATOM 4703 HN SER X 311 10.593 10.355 11.742 0.00 0.00 H

ATOM 4704 CA SER X 311 10.317 12.406 12.322 0.00 0.00 C

ATOM 4705 HA SER X 311 11.117 12.489 11.607 0.00 0.00 H

ATOM 4706 CB SER X 311 9.238 13.391 11.786 0.00 0.00 C

ATOM 4707 HB1 SER X 311 8.767 12.996 10.863 0.00 0.00 H

ATOM 4708 HB2 SER X 311 9.787 14.349 11.601 0.00 0.00 H

ATOM 4709 OG SER X 311 8.173 13.685 12.658 0.00 0.00 O

ATOM 4710 HG1 SER X 311 7.785 12.842 12.894 0.00 0.00 H

ATOM 4711 C SER X 311 10.929 12.605 13.686 0.00 0.00 C

ATOM 4712 O SER X 311 10.274 12.267 14.655 0.00 0.00 O

ATOM 4713 N GLY X 312 12.198 13.156 13.727 0.00 0.00 N

ATOM 4714 HN GLY X 312 12.768 13.106 12.906 0.00 0.00 H

ATOM 4715 CA GLY X 312 12.597 13.973 14.906 0.00 0.00 C

ATOM 4716 HA1 GLY X 312 12.657 13.455 15.854 0.00 0.00 H

ATOM 4717 HA2 GLY X 312 11.946 14.811 15.092 0.00 0.00 H

ATOM 4718 C GLY X 312 13.936 14.571 14.871 0.00 0.00 C

ATOM 4719 O GLY X 312 14.417 14.977 15.915 0.00 0.00 O

ATOM 4720 N PHE X 313 14.592 14.425 13.723 0.00 0.00 N

ATOM 4721 HN PHE X 313 14.271 14.089 12.843 0.00 0.00 H

ATOM 4722 CA PHE X 313 16.062 14.748 13.600 0.00 0.00 C

ATOM 4723 HA PHE X 313 16.365 15.366 14.418 0.00 0.00 H

ATOM 4724 CB PHE X 313 16.812 13.407 13.589 0.00 0.00 C

ATOM 4725 HB1 PHE X 313 16.437 12.999 12.628 0.00 0.00 H

ATOM 4726 HB2 PHE X 313 16.514 12.649 14.353 0.00 0.00 H

ATOM 4727 CG PHE X 313 18.269 13.537 13.447 0.00 0.00 C

ATOM 4728 CD1 PHE X 313 18.921 13.702 12.287 0.00 0.00 C

ATOM 4729 HD1 PHE X 313 18.391 13.823 11.355 0.00 0.00 H

ATOM 4730 CE1 PHE X 313 20.258 13.935 12.166 0.00 0.00 C

ATOM 4731 HE1 PHE X 313 20.695 14.408 11.295 0.00 0.00 H

ATOM 4732 CZ PHE X 313 21.076 13.826 13.371 0.00 0.00 C

ATOM 4733 HZ PHE X 313 22.137 14.021 13.252 0.00 0.00 H

ATOM 4734 CD2 PHE X 313 19.038 13.574 14.637 0.00 0.00 C

ATOM 4735 HD2 PHE X 313 18.447 13.363 15.515 0.00 0.00 H

ATOM 4736 CE2 PHE X 313 20.405 13.575 14.605 0.00 0.00 C

ATOM 4737 HE2 PHE X 313 20.953 13.464 15.529 0.00 0.00 H

ATOM 4738 C PHE X 313 16.318 15.657 12.468 0.00 0.00 C

ATOM 4739 O PHE X 313 17.232 16.485 12.473 0.00 0.00 O

ATOM 4740 N THR X 314 15.473 15.596 11.392 0.00 0.00 N

ATOM 4741 HN THR X 314 14.845 14.827 11.395 0.00 0.00 H

ATOM 4742 CA THR X 314 15.562 16.523 10.231 0.00 0.00 C

ATOM 4743 HA THR X 314 16.519 17.039 10.221 0.00 0.00 H

ATOM 4744 CB THR X 314 15.559 15.886 8.882 0.00 0.00 C

ATOM 4745 HB THR X 314 14.608 15.360 8.713 0.00 0.00 H

ATOM 4746 OG1 THR X 314 16.451 14.769 9.013 0.00 0.00 O

ATOM 4747 HG1 THR X 314 16.110 14.308 9.773 0.00 0.00 H

ATOM 4748 CG2 THR X 314 15.943 16.737 7.678 0.00 0.00 C

ATOM 4749 HG21 THR X 314 16.045 16.176 6.728 0.00 0.00 H

ATOM 4750 HG22 THR X 314 16.979 17.122 7.826 0.00 0.00 H

ATOM 4751 HG23 THR X 314 15.230 17.572 7.535 0.00 0.00 H

ATOM 4752 C THR X 314 14.409 17.506 10.302 0.00 0.00 C

ATOM 4753 O THR X 314 13.253 17.065 10.155 0.00 0.00 O

ATOM 4754 N PRO X 315 14.582 18.771 10.469 0.00 0.00 N

ATOM 4755 CD PRO X 315 15.674 19.260 11.272 0.00 0.00 C

ATOM 4756 HD1 PRO X 315 16.606 19.388 10.679 0.00 0.00 H

ATOM 4757 HD2 PRO X 315 15.951 18.593 12.112 0.00 0.00 H

ATOM 4758 CA PRO X 315 13.525 19.724 10.301 0.00 0.00 C

ATOM 4759 HA PRO X 315 12.807 19.395 11.031 0.00 0.00 H

ATOM 4760 CB PRO X 315 14.277 21.072 10.555 0.00 0.00 C

ATOM 4761 HB1 PRO X 315 13.487 21.805 10.824 0.00 0.00 H

ATOM 4762 HB2 PRO X 315 14.889 21.446 9.700 0.00 0.00 H

ATOM 4763 CG PRO X 315 15.126 20.597 11.768 0.00 0.00 C

ATOM 4764 HG1 PRO X 315 15.935 21.281 12.104 0.00 0.00 H

ATOM 4765 HG2 PRO X 315 14.373 20.560 12.586 0.00 0.00 H

ATOM 4766 C PRO X 315 12.970 19.709 8.941 0.00 0.00 C

ATOM 4767 O PRO X 315 13.694 19.515 7.994 0.00 0.00 O

ATOM 4768 N LEU X 316 11.654 19.915 8.814 0.00 0.00 N

ATOM 4769 HN LEU X 316 11.138 19.918 9.664 0.00 0.00 H

ATOM 4770 CA LEU X 316 10.993 20.135 7.568 0.00 0.00 C

ATOM 4771 HA LEU X 316 10.033 20.506 7.882 0.00 0.00 H

ATOM 4772 CB LEU X 316 11.414 21.306 6.749 0.00 0.00 C

ATOM 4773 HB1 LEU X 316 10.594 21.480 6.013 0.00 0.00 H

ATOM 4774 HB2 LEU X 316 12.265 21.078 6.071 0.00 0.00 H

ATOM 4775 CG LEU X 316 11.781 22.511 7.556 0.00 0.00 C

ATOM 4776 HG LEU X 316 12.525 22.231 8.322 0.00 0.00 H

ATOM 4777 CD1 LEU X 316 12.431 23.696 6.782 0.00 0.00 C

ATOM 4778 HD11 LEU X 316 12.525 24.546 7.482 0.00 0.00 H

ATOM 4779 HD12 LEU X 316 11.934 24.020 5.840 0.00 0.00 H

ATOM 4780 HD13 LEU X 316 13.448 23.386 6.460 0.00 0.00 H

ATOM 4781 CD2 LEU X 316 10.544 23.000 8.306 0.00 0.00 C

ATOM 4782 HD21 LEU X 316 10.295 22.239 9.076 0.00 0.00 H

ATOM 4783 HD22 LEU X 316 9.716 23.286 7.632 0.00 0.00 H

ATOM 4784 HD23 LEU X 316 10.909 23.870 8.893 0.00 0.00 H

ATOM 4785 C LEU X 316 10.737 18.826 6.699 0.00 0.00 C

ATOM 4786 O LEU X 316 10.205 18.933 5.575 0.00 0.00 O

ATOM 4787 N ILE X 317 11.088 17.581 7.193 0.00 0.00 N

ATOM 4788 HN ILE X 317 11.467 17.365 8.086 0.00 0.00 H

ATOM 4789 CA ILE X 317 10.989 16.271 6.523 0.00 0.00 C

ATOM 4790 HA ILE X 317 11.566 16.434 5.618 0.00 0.00 H

ATOM 4791 CB ILE X 317 11.700 15.167 7.269 0.00 0.00 C

ATOM 4792 HB ILE X 317 12.700 15.558 7.544 0.00 0.00 H

ATOM 4793 CG2 ILE X 317 10.917 15.000 8.606 0.00 0.00 C

ATOM 4794 HG21 ILE X 317 10.758 15.952 9.160 0.00 0.00 H

ATOM 4795 HG22 ILE X 317 11.389 14.273 9.290 0.00 0.00 H

ATOM 4796 HG23 ILE X 317 9.894 14.664 8.315 0.00 0.00 H

ATOM 4797 CG1 ILE X 317 11.943 13.844 6.506 0.00 0.00 C

ATOM 4798 HG11 ILE X 317 10.913 13.430 6.381 0.00 0.00 H

ATOM 4799 HG12 ILE X 317 12.533 13.095 7.084 0.00 0.00 H

ATOM 4800 CD ILE X 317 12.638 14.050 5.177 0.00 0.00 C

ATOM 4801 HD1 ILE X 317 13.663 14.456 5.272 0.00 0.00 H

ATOM 4802 HD2 ILE X 317 12.153 14.712 4.435 0.00 0.00 H

ATOM 4803 HD3 ILE X 317 12.803 13.131 4.571 0.00 0.00 H

ATOM 4804 C ILE X 317 9.511 16.012 6.097 0.00 0.00 C

ATOM 4805 O ILE X 317 9.207 15.358 5.109 0.00 0.00 O

ATOM 4806 N SER X 318 8.528 16.419 6.951 0.00 0.00 N

ATOM 4807 HN SER X 318 8.875 16.962 7.708 0.00 0.00 H

ATOM 4808 CA SER X 318 7.160 16.136 6.951 0.00 0.00 C

ATOM 4809 HA SER X 318 7.114 15.068 7.067 0.00 0.00 H

ATOM 4810 CB SER X 318 6.372 16.671 8.140 0.00 0.00 C

ATOM 4811 HB1 SER X 318 5.346 16.260 8.106 0.00 0.00 H

ATOM 4812 HB2 SER X 318 6.354 17.781 8.024 0.00 0.00 H

ATOM 4813 OG SER X 318 6.918 16.089 9.337 0.00 0.00 O

ATOM 4814 HG1 SER X 318 7.757 16.534 9.470 0.00 0.00 H

ATOM 4815 C SER X 318 6.341 16.498 5.739 0.00 0.00 C

ATOM 4816 O SER X 318 5.518 15.769 5.143 0.00 0.00 O

ATOM 4817 N ALA X 319 6.614 17.754 5.201 0.00 0.00 N

ATOM 4818 HN ALA X 319 7.094 18.502 5.658 0.00 0.00 H

ATOM 4819 CA ALA X 319 6.197 18.134 3.931 0.00 0.00 C

ATOM 4820 HA ALA X 319 5.122 18.065 3.889 0.00 0.00 H

ATOM 4821 CB ALA X 319 6.580 19.622 3.561 0.00 0.00 C

ATOM 4822 HB1 ALA X 319 6.471 20.289 4.443 0.00 0.00 H

ATOM 4823 HB2 ALA X 319 5.922 19.925 2.723 0.00 0.00 H

ATOM 4824 HB3 ALA X 319 7.645 19.669 3.231 0.00 0.00 H

ATOM 4825 C ALA X 319 6.671 17.346 2.775 0.00 0.00 C

ATOM 4826 O ALA X 319 6.074 17.274 1.727 0.00 0.00 O

ATOM 4827 N GLY X 320 7.957 16.853 2.807 0.00 0.00 N

ATOM 4828 HN GLY X 320 8.501 17.155 3.582 0.00 0.00 H

ATOM 4829 CA GLY X 320 8.547 16.107 1.661 0.00 0.00 C

ATOM 4830 HA1 GLY X 320 9.584 16.081 1.977 0.00 0.00 H

ATOM 4831 HA2 GLY X 320 8.409 16.582 0.696 0.00 0.00 H

ATOM 4832 C GLY X 320 7.968 14.729 1.669 0.00 0.00 C

ATOM 4833 O GLY X 320 7.784 14.196 0.545 0.00 0.00 O

ATOM 4834 N ILE X 321 7.821 14.107 2.773 0.00 0.00 N

ATOM 4835 HN ILE X 321 8.181 14.591 3.566 0.00 0.00 H

ATOM 4836 CA ILE X 321 7.262 12.749 2.822 0.00 0.00 C

ATOM 4837 HA ILE X 321 7.574 12.172 1.967 0.00 0.00 H

ATOM 4838 CB ILE X 321 7.762 12.013 4.095 0.00 0.00 C

ATOM 4839 HB ILE X 321 7.419 10.953 4.037 0.00 0.00 H

ATOM 4840 CG2 ILE X 321 9.294 12.091 4.095 0.00 0.00 C

ATOM 4841 HG21 ILE X 321 9.703 13.089 4.346 0.00 0.00 H

ATOM 4842 HG22 ILE X 321 9.744 11.785 3.127 0.00 0.00 H

ATOM 4843 HG23 ILE X 321 9.696 11.290 4.757 0.00 0.00 H

ATOM 4844 CG1 ILE X 321 7.179 12.643 5.421 0.00 0.00 C

ATOM 4845 HG11 ILE X 321 6.084 12.857 5.338 0.00 0.00 H

ATOM 4846 HG12 ILE X 321 7.607 13.656 5.602 0.00 0.00 H

ATOM 4847 CD ILE X 321 7.540 11.796 6.646 0.00 0.00 C

ATOM 4848 HD1 ILE X 321 8.604 11.512 6.811 0.00 0.00 H

ATOM 4849 HD2 ILE X 321 7.011 10.816 6.572 0.00 0.00 H

ATOM 4850 HD3 ILE X 321 7.186 12.361 7.527 0.00 0.00 H

ATOM 4851 C ILE X 321 5.794 12.701 2.473 0.00 0.00 C

ATOM 4852 O ILE X 321 5.242 11.833 1.755 0.00 0.00 O

ATOM 4853 N PHE X 322 4.982 13.723 2.841 0.00 0.00 N

ATOM 4854 HN PHE X 322 5.364 14.445 3.410 0.00 0.00 H

ATOM 4855 CA PHE X 322 3.659 13.936 2.189 0.00 0.00 C

ATOM 4856 HA PHE X 322 3.133 12.985 2.144 0.00 0.00 H

ATOM 4857 CB PHE X 322 2.669 14.876 2.996 0.00 0.00 C

ATOM 4858 HB1 PHE X 322 1.806 15.343 2.488 0.00 0.00 H

ATOM 4859 HB2 PHE X 322 3.303 15.731 3.321 0.00 0.00 H

ATOM 4860 CG PHE X 322 2.294 14.081 4.218 0.00 0.00 C

ATOM 4861 CD1 PHE X 322 1.454 12.912 4.012 0.00 0.00 C

ATOM 4862 HD1 PHE X 322 1.016 12.739 3.048 0.00 0.00 H

ATOM 4863 CE1 PHE X 322 1.185 12.062 5.108 0.00 0.00 C

ATOM 4864 HE1 PHE X 322 0.331 11.399 5.049 0.00 0.00 H

ATOM 4865 CZ PHE X 322 1.741 12.405 6.386 0.00 0.00 C

ATOM 4866 HZ PHE X 322 1.412 11.845 7.244 0.00 0.00 H

ATOM 4867 CD2 PHE X 322 2.732 14.425 5.452 0.00 0.00 C

ATOM 4868 HD2 PHE X 322 3.305 15.334 5.586 0.00 0.00 H

ATOM 4869 CE2 PHE X 322 2.588 13.519 6.505 0.00 0.00 C

ATOM 4870 HE2 PHE X 322 2.867 13.889 7.479 0.00 0.00 H

ATOM 4871 C PHE X 322 3.737 14.353 0.728 0.00 0.00 C

ATOM 4872 O PHE X 322 2.913 13.896 -0.078 0.00 0.00 O

ATOM 4873 N SER X 323 4.713 15.114 0.300 0.00 0.00 N

ATOM 4874 HN SER X 323 5.266 15.610 0.952 0.00 0.00 H

ATOM 4875 CA SER X 323 4.994 15.349 -1.096 0.00 0.00 C

ATOM 4876 HA SER X 323 4.090 15.871 -1.361 0.00 0.00 H

ATOM 4877 CB SER X 323 6.194 16.219 -1.467 0.00 0.00 C

ATOM 4878 HB1 SER X 323 6.371 16.294 -2.561 0.00 0.00 H

ATOM 4879 HB2 SER X 323 7.098 15.719 -1.053 0.00 0.00 H

ATOM 4880 OG SER X 323 5.893 17.544 -0.942 0.00 0.00 O

ATOM 4881 HG1 SER X 323 5.940 17.527 0.012 0.00 0.00 H

ATOM 4882 C SER X 323 5.136 14.098 -1.986 0.00 0.00 C

ATOM 4883 O SER X 323 4.364 13.883 -2.953 0.00 0.00 O

ATOM 4884 N ALA X 324 5.996 13.107 -1.574 0.00 0.00 N

ATOM 4885 HN ALA X 324 6.614 13.228 -0.799 0.00 0.00 H

ATOM 4886 CA ALA X 324 6.193 11.816 -2.142 0.00 0.00 C

ATOM 4887 HA ALA X 324 6.316 11.968 -3.208 0.00 0.00 H

ATOM 4888 CB ALA X 324 7.454 11.145 -1.537 0.00 0.00 C

ATOM 4889 HB1 ALA X 324 8.357 11.437 -2.113 0.00 0.00 H

ATOM 4890 HB2 ALA X 324 7.364 10.049 -1.585 0.00 0.00 H

ATOM 4891 HB3 ALA X 324 7.516 11.362 -0.446 0.00 0.00 H

ATOM 4892 C ALA X 324 4.949 10.923 -1.987 0.00 0.00 C

ATOM 4893 O ALA X 324 4.421 10.406 -2.998 0.00 0.00 O

ATOM 4894 N THR X 325 4.360 10.805 -0.793 0.00 0.00 N

ATOM 4895 HN THR X 325 4.754 11.218 0.019 0.00 0.00 H

ATOM 4896 CA THR X 325 3.094 10.141 -0.625 0.00 0.00 C

ATOM 4897 HA THR X 325 3.170 9.109 -0.922 0.00 0.00 H

ATOM 4898 CB THR X 325 2.644 10.238 0.841 0.00 0.00 C

ATOM 4899 HB THR X 325 3.038 11.189 1.255 0.00 0.00 H

ATOM 4900 OG1 THR X 325 3.343 9.177 1.512 0.00 0.00 O

ATOM 4901 HG1 THR X 325 4.068 8.984 0.909 0.00 0.00 H

ATOM 4902 CG2 THR X 325 1.147 10.013 1.096 0.00 0.00 C

ATOM 4903 HG21 THR X 325 0.977 9.683 2.148 0.00 0.00 H

ATOM 4904 HG22 THR X 325 0.759 9.320 0.324 0.00 0.00 H

ATOM 4905 HG23 THR X 325 0.664 10.970 0.809 0.00 0.00 H

ATOM 4906 C THR X 325 1.882 10.665 -1.397 0.00 0.00 C

ATOM 4907 O THR X 325 1.272 9.928 -2.201 0.00 0.00 O

ATOM 4908 N LEU X 326 1.562 11.963 -1.407 0.00 0.00 N

ATOM 4909 HN LEU X 326 1.978 12.670 -0.844 0.00 0.00 H

ATOM 4910 CA LEU X 326 0.488 12.485 -2.245 0.00 0.00 C

ATOM 4911 HA LEU X 326 -0.369 11.856 -2.045 0.00 0.00 H

ATOM 4912 CB LEU X 326 0.001 13.849 -1.673 0.00 0.00 C

ATOM 4913 HB1 LEU X 326 -0.747 14.254 -2.396 0.00 0.00 H

ATOM 4914 HB2 LEU X 326 0.772 14.646 -1.732 0.00 0.00 H

ATOM 4915 CG LEU X 326 -0.520 13.893 -0.251 0.00 0.00 C

ATOM 4916 HG LEU X 326 0.357 13.909 0.439 0.00 0.00 H

ATOM 4917 CD1 LEU X 326 -1.336 15.185 -0.012 0.00 0.00 C

ATOM 4918 HD11 LEU X 326 -1.998 15.423 -0.866 0.00 0.00 H

ATOM 4919 HD12 LEU X 326 -0.539 15.952 0.024 0.00 0.00 H

ATOM 4920 HD13 LEU X 326 -1.955 15.155 0.910 0.00 0.00 H

ATOM 4921 CD2 LEU X 326 -1.369 12.634 0.073 0.00 0.00 C

ATOM 4922 HD21 LEU X 326 -1.741 12.567 1.116 0.00 0.00 H

ATOM 4923 HD22 LEU X 326 -0.813 11.676 0.006 0.00 0.00 H

ATOM 4924 HD23 LEU X 326 -2.233 12.637 -0.626 0.00 0.00 H

ATOM 4925 C LEU X 326 0.783 12.553 -3.780 0.00 0.00 C

ATOM 4926 O LEU X 326 -0.211 12.610 -4.517 0.00 0.00 O

ATOM 4927 N SER X 327 2.057 12.394 -4.245 0.00 0.00 N

ATOM 4928 HN SER X 327 2.848 12.488 -3.631 0.00 0.00 H

ATOM 4929 CA SER X 327 2.407 12.076 -5.597 0.00 0.00 C

ATOM 4930 HA SER X 327 2.026 12.844 -6.254 0.00 0.00 H

ATOM 4931 CB SER X 327 3.921 11.962 -5.767 0.00 0.00 C

ATOM 4932 HB1 SER X 327 4.232 11.560 -6.755 0.00 0.00 H

ATOM 4933 HB2 SER X 327 4.331 11.265 -5.001 0.00 0.00 H

ATOM 4934 OG SER X 327 4.560 13.235 -5.772 0.00 0.00 O

ATOM 4935 HG1 SER X 327 4.451 13.542 -4.867 0.00 0.00 H

ATOM 4936 C SER X 327 1.920 10.766 -6.067 0.00 0.00 C

ATOM 4937 O SER X 327 1.343 10.739 -7.147 0.00 0.00 O

ATOM 4938 N SER X 328 2.096 9.734 -5.284 0.00 0.00 N

ATOM 4939 HN SER X 328 2.415 9.907 -4.353 0.00 0.00 H

ATOM 4940 CA SER X 328 1.609 8.354 -5.558 0.00 0.00 C

ATOM 4941 HA SER X 328 2.071 8.027 -6.474 0.00 0.00 H

ATOM 4942 CB SER X 328 2.193 7.246 -4.493 0.00 0.00 C

ATOM 4943 HB1 SER X 328 1.815 7.475 -3.471 0.00 0.00 H

ATOM 4944 HB2 SER X 328 3.280 7.427 -4.498 0.00 0.00 H

ATOM 4945 OG SER X 328 1.949 5.949 -4.908 0.00 0.00 O

ATOM 4946 HG1 SER X 328 1.753 5.482 -4.102 0.00 0.00 H

ATOM 4947 C SER X 328 0.085 8.324 -5.601 0.00 0.00 C

ATOM 4948 O SER X 328 -0.475 7.568 -6.429 0.00 0.00 O

ATOM 4949 N ALA X 329 -0.582 9.107 -4.779 0.00 0.00 N

ATOM 4950 HN ALA X 329 0.092 9.575 -4.212 0.00 0.00 H

ATOM 4951 CA ALA X 329 -2.029 9.331 -4.750 0.00 0.00 C

ATOM 4952 HA ALA X 329 -2.434 8.357 -4.941 0.00 0.00 H

ATOM 4953 CB ALA X 329 -2.650 10.100 -3.558 0.00 0.00 C

ATOM 4954 HB1 ALA X 329 -2.464 11.181 -3.720 0.00 0.00 H

ATOM 4955 HB2 ALA X 329 -2.137 9.911 -2.592 0.00 0.00 H

ATOM 4956 HB3 ALA X 329 -3.764 10.104 -3.569 0.00 0.00 H

ATOM 4957 C ALA X 329 -2.468 10.047 -6.025 0.00 0.00 C

ATOM 4958 O ALA X 329 -3.401 9.617 -6.723 0.00 0.00 O

ATOM 4959 N LEU X 330 -1.807 11.136 -6.499 0.00 0.00 N

ATOM 4960 HN LEU X 330 -1.142 11.655 -5.956 0.00 0.00 H

ATOM 4961 CA LEU X 330 -1.974 11.687 -7.824 0.00 0.00 C

ATOM 4962 HA LEU X 330 -3.019 11.806 -8.046 0.00 0.00 H

ATOM 4963 CB LEU X 330 -1.341 13.106 -7.970 0.00 0.00 C

ATOM 4964 HB1 LEU X 330 -1.566 13.378 -9.021 0.00 0.00 H

ATOM 4965 HB2 LEU X 330 -0.230 13.109 -7.970 0.00 0.00 H

ATOM 4966 CG LEU X 330 -1.763 14.193 -7.009 0.00 0.00 C

ATOM 4967 HG LEU X 330 -2.121 13.721 -6.069 0.00 0.00 H

ATOM 4968 CD1 LEU X 330 -0.545 15.107 -6.796 0.00 0.00 C

ATOM 4969 HD11 LEU X 330 -0.482 15.710 -7.726 0.00 0.00 H

ATOM 4970 HD12 LEU X 330 0.441 14.646 -6.577 0.00 0.00 H

ATOM 4971 HD13 LEU X 330 -0.722 15.925 -6.073 0.00 0.00 H

ATOM 4972 CD2 LEU X 330 -2.921 14.918 -7.718 0.00 0.00 C

ATOM 4973 HD21 LEU X 330 -2.785 15.263 -8.765 0.00 0.00 H

ATOM 4974 HD22 LEU X 330 -3.400 15.732 -7.139 0.00 0.00 H

ATOM 4975 HD23 LEU X 330 -3.732 14.154 -7.756 0.00 0.00 H

ATOM 4976 C LEU X 330 -1.578 10.768 -9.020 0.00 0.00 C

ATOM 4977 O LEU X 330 -2.226 10.710 -9.984 0.00 0.00 O

ATOM 4978 N ALA X 331 -0.466 10.081 -8.944 0.00 0.00 N

ATOM 4979 HN ALA X 331 0.159 10.229 -8.180 0.00 0.00 H

ATOM 4980 CA ALA X 331 -0.114 8.988 -9.871 0.00 0.00 C

ATOM 4981 HA ALA X 331 -0.067 9.492 -10.827 0.00 0.00 H

ATOM 4982 CB ALA X 331 1.234 8.390 -9.503 0.00 0.00 C

ATOM 4983 HB1 ALA X 331 1.552 7.594 -10.222 0.00 0.00 H

ATOM 4984 HB2 ALA X 331 1.152 7.932 -8.493 0.00 0.00 H

ATOM 4985 HB3 ALA X 331 2.018 9.169 -9.580 0.00 0.00 H

ATOM 4986 C ALA X 331 -1.146 7.881 -10.011 0.00 0.00 C

ATOM 4987 O ALA X 331 -1.570 7.649 -11.121 0.00 0.00 O

ATOM 4988 N SER X 332 -1.647 7.337 -8.911 0.00 0.00 N

ATOM 4989 HN SER X 332 -1.032 7.373 -8.120 0.00 0.00 H

ATOM 4990 CA SER X 332 -2.811 6.492 -8.790 0.00 0.00 C

ATOM 4991 HA SER X 332 -2.596 5.580 -9.331 0.00 0.00 H

ATOM 4992 CB SER X 332 -3.146 5.978 -7.401 0.00 0.00 C

ATOM 4993 HB1 SER X 332 -3.905 5.189 -7.603 0.00 0.00 H

ATOM 4994 HB2 SER X 332 -3.502 6.882 -6.855 0.00 0.00 H

ATOM 4995 OG SER X 332 -2.015 5.350 -6.722 0.00 0.00 O

ATOM 4996 HG1 SER X 332 -1.423 6.095 -6.646 0.00 0.00 H

ATOM 4997 C SER X 332 -4.128 7.030 -9.393 0.00 0.00 C

ATOM 4998 O SER X 332 -4.888 6.409 -10.211 0.00 0.00 O

ATOM 4999 N LEU X 333 -4.403 8.375 -9.109 0.00 0.00 N

ATOM 5000 HN LEU X 333 -3.645 8.857 -8.665 0.00 0.00 H

ATOM 5001 CA LEU X 333 -5.641 8.967 -9.616 0.00 0.00 C

ATOM 5002 HA LEU X 333 -6.426 8.277 -9.333 0.00 0.00 H

ATOM 5003 CB LEU X 333 -5.955 10.305 -8.842 0.00 0.00 C

ATOM 5004 HB1 LEU X 333 -5.197 11.072 -9.143 0.00 0.00 H

ATOM 5005 HB2 LEU X 333 -5.922 10.276 -7.727 0.00 0.00 H

ATOM 5006 CG LEU X 333 -7.405 10.992 -9.126 0.00 0.00 C

ATOM 5007 HG LEU X 333 -7.595 11.352 -10.149 0.00 0.00 H

ATOM 5008 CD1 LEU X 333 -8.556 9.949 -9.011 0.00 0.00 C

ATOM 5009 HD11 LEU X 333 -8.140 9.130 -8.377 0.00 0.00 H

ATOM 5010 HD12 LEU X 333 -8.764 9.548 -10.026 0.00 0.00 H

ATOM 5011 HD13 LEU X 333 -9.502 10.420 -8.662 0.00 0.00 H

ATOM 5012 CD2 LEU X 333 -7.407 12.200 -8.259 0.00 0.00 C

ATOM 5013 HD21 LEU X 333 -6.492 12.827 -8.403 0.00 0.00 H

ATOM 5014 HD22 LEU X 333 -7.428 11.950 -7.176 0.00 0.00 H

ATOM 5015 HD23 LEU X 333 -8.281 12.865 -8.429 0.00 0.00 H

ATOM 5016 C LEU X 333 -5.566 9.262 -11.069 0.00 0.00 C

ATOM 5017 O LEU X 333 -6.591 8.986 -11.788 0.00 0.00 O

ATOM 5018 N VAL X 334 -4.414 9.691 -11.687 0.00 0.00 N

ATOM 5019 HN VAL X 334 -3.617 9.869 -11.098 0.00 0.00 H

ATOM 5020 CA VAL X 334 -4.198 9.726 -13.158 0.00 0.00 C

ATOM 5021 HA VAL X 334 -5.029 10.231 -13.635 0.00 0.00 H

ATOM 5022 CB VAL X 334 -2.985 10.502 -13.491 0.00 0.00 C

ATOM 5023 HB VAL X 334 -2.086 10.123 -12.954 0.00 0.00 H

ATOM 5024 CG1 VAL X 334 -2.649 10.546 -15.050 0.00 0.00 C

ATOM 5025 HG11 VAL X 334 -1.707 11.101 -15.226 0.00 0.00 H

ATOM 5026 HG12 VAL X 334 -3.399 11.072 -15.671 0.00 0.00 H

ATOM 5027 HG13 VAL X 334 -2.368 9.517 -15.354 0.00 0.00 H

ATOM 5028 CG2 VAL X 334 -3.270 12.002 -13.178 0.00 0.00 C

ATOM 5029 HG21 VAL X 334 -2.528 12.669 -13.676 0.00 0.00 H

ATOM 5030 HG22 VAL X 334 -3.128 12.281 -12.112 0.00 0.00 H

ATOM 5031 HG23 VAL X 334 -4.283 12.349 -13.473 0.00 0.00 H

ATOM 5032 C VAL X 334 -4.113 8.343 -13.854 0.00 0.00 C

ATOM 5033 O VAL X 334 -4.586 8.113 -14.979 0.00 0.00 O

ATOM 5034 N SER X 335 -3.530 7.316 -13.248 0.00 0.00 N

ATOM 5035 HN SER X 335 -3.053 7.440 -12.378 0.00 0.00 H

ATOM 5036 CA SER X 335 -3.414 5.983 -13.812 0.00 0.00 C

ATOM 5037 HA SER X 335 -2.800 6.088 -14.700 0.00 0.00 H

ATOM 5038 CB SER X 335 -2.666 5.090 -12.865 0.00 0.00 C

ATOM 5039 HB1 SER X 335 -3.219 4.801 -11.938 0.00 0.00 H

ATOM 5040 HB2 SER X 335 -1.900 5.676 -12.326 0.00 0.00 H

ATOM 5041 OG SER X 335 -2.096 3.947 -13.463 0.00 0.00 O

ATOM 5042 HG1 SER X 335 -1.218 4.229 -13.728 0.00 0.00 H

ATOM 5043 C SER X 335 -4.709 5.225 -14.095 0.00 0.00 C

ATOM 5044 O SER X 335 -4.732 4.550 -15.101 0.00 0.00 O

ATOM 5045 N ALA X 336 -5.690 5.138 -13.148 0.00 0.00 N

ATOM 5046 HN ALA X 336 -5.593 5.385 -12.186 0.00 0.00 H

ATOM 5047 CA ALA X 336 -6.912 4.370 -13.331 0.00 0.00 C

ATOM 5048 HA ALA X 336 -6.642 3.347 -13.536 0.00 0.00 H

ATOM 5049 CB ALA X 336 -7.886 4.465 -12.153 0.00 0.00 C

ATOM 5050 HB1 ALA X 336 -8.223 5.495 -11.915 0.00 0.00 H

ATOM 5051 HB2 ALA X 336 -7.445 4.029 -11.229 0.00 0.00 H

ATOM 5052 HB3 ALA X 336 -8.732 3.767 -12.330 0.00 0.00 H

ATOM 5053 C ALA X 336 -7.671 4.718 -14.586 0.00 0.00 C

ATOM 5054 O ALA X 336 -8.088 3.748 -15.202 0.00 0.00 O

ATOM 5055 N PRO X 337 -7.913 5.968 -15.139 0.00 0.00 N

ATOM 5056 CD PRO X 337 -7.853 7.130 -14.373 0.00 0.00 C

ATOM 5057 HD1 PRO X 337 -6.808 7.464 -14.210 0.00 0.00 H

ATOM 5058 HD2 PRO X 337 -8.402 6.957 -13.423 0.00 0.00 H

ATOM 5059 CA PRO X 337 -8.559 6.210 -16.475 0.00 0.00 C

ATOM 5060 HA PRO X 337 -9.525 5.736 -16.378 0.00 0.00 H

ATOM 5061 CB PRO X 337 -8.621 7.719 -16.724 0.00 0.00 C

ATOM 5062 HB1 PRO X 337 -9.541 8.090 -17.212 0.00 0.00 H

ATOM 5063 HB2 PRO X 337 -7.792 8.208 -17.270 0.00 0.00 H

ATOM 5064 CG PRO X 337 -8.548 8.220 -15.217 0.00 0.00 C

ATOM 5065 HG1 PRO X 337 -7.857 9.093 -15.209 0.00 0.00 H

ATOM 5066 HG2 PRO X 337 -9.497 8.503 -14.714 0.00 0.00 H

ATOM 5067 C PRO X 337 -7.872 5.445 -17.610 0.00 0.00 C

ATOM 5068 O PRO X 337 -8.529 4.965 -18.517 0.00 0.00 O

ATOM 5069 N LYS X 338 -6.528 5.480 -17.627 0.00 0.00 N

ATOM 5070 HN LYS X 338 -6.020 5.889 -16.863 0.00 0.00 H

ATOM 5071 CA LYS X 338 -5.680 4.953 -18.699 0.00 0.00 C

ATOM 5072 HA LYS X 338 -5.982 5.449 -19.602 0.00 0.00 H

ATOM 5073 CB LYS X 338 -4.122 5.161 -18.412 0.00 0.00 C

ATOM 5074 HB1 LYS X 338 -3.526 4.809 -19.281 0.00 0.00 H

ATOM 5075 HB2 LYS X 338 -3.803 4.670 -17.467 0.00 0.00 H

ATOM 5076 CG LYS X 338 -3.824 6.612 -18.193 0.00 0.00 C

ATOM 5077 HG1 LYS X 338 -4.584 7.026 -17.497 0.00 0.00 H

ATOM 5078 HG2 LYS X 338 -4.021 7.018 -19.210 0.00 0.00 H

ATOM 5079 CD LYS X 338 -2.448 6.822 -17.553 0.00 0.00 C

ATOM 5080 HD1 LYS X 338 -2.525 6.155 -16.670 0.00 0.00 H

ATOM 5081 HD2 LYS X 338 -2.393 7.867 -17.173 0.00 0.00 H

ATOM 5082 CE LYS X 338 -1.295 6.540 -18.407 0.00 0.00 C

ATOM 5083 HE1 LYS X 338 -1.208 5.496 -18.761 0.00 0.00 H

ATOM 5084 HE2 LYS X 338 -0.371 6.671 -17.810 0.00 0.00 H

ATOM 5085 NZ LYS X 338 -1.396 7.440 -19.546 0.00 0.00 N1+

ATOM 5086 HZ1 LYS X 338 -1.729 6.998 -20.430 0.00 0.00 H

ATOM 5087 HZ2 LYS X 338 -0.397 7.561 -19.808 0.00 0.00 H

ATOM 5088 HZ3 LYS X 338 -1.873 8.343 -19.376 0.00 0.00 H

ATOM 5089 C LYS X 338 -5.804 3.423 -18.923 0.00 0.00 C

ATOM 5090 O LYS X 338 -5.900 2.953 -20.012 0.00 0.00 O

ATOM 5091 N ILE X 339 -5.930 2.750 -17.763 0.00 0.00 N

ATOM 5092 HN ILE X 339 -5.839 3.267 -16.921 0.00 0.00 H

ATOM 5093 CA ILE X 339 -6.085 1.315 -17.766 0.00 0.00 C

ATOM 5094 HA ILE X 339 -5.468 0.869 -18.536 0.00 0.00 H

ATOM 5095 CB ILE X 339 -5.887 0.613 -16.458 0.00 0.00 C

ATOM 5096 HB ILE X 339 -6.514 1.090 -15.671 0.00 0.00 H

ATOM 5097 CG2 ILE X 339 -6.108 -0.867 -16.635 0.00 0.00 C

ATOM 5098 HG21 ILE X 339 -5.471 -1.190 -17.492 0.00 0.00 H

ATOM 5099 HG22 ILE X 339 -7.144 -1.109 -16.974 0.00 0.00 H

ATOM 5100 HG23 ILE X 339 -5.764 -1.459 -15.762 0.00 0.00 H

ATOM 5101 CG1 ILE X 339 -4.512 0.900 -15.853 0.00 0.00 C

ATOM 5102 HG11 ILE X 339 -4.152 1.913 -16.133 0.00 0.00 H

ATOM 5103 HG12 ILE X 339 -3.811 0.110 -16.198 0.00 0.00 H

ATOM 5104 CD ILE X 339 -4.539 0.881 -14.292 0.00 0.00 C

ATOM 5105 HD1 ILE X 339 -3.591 1.358 -13.938 0.00 0.00 H

ATOM 5106 HD2 ILE X 339 -4.620 -0.187 -14.018 0.00 0.00 H

ATOM 5107 HD3 ILE X 339 -5.519 1.300 -13.983 0.00 0.00 H

ATOM 5108 C ILE X 339 -7.461 1.003 -18.278 0.00 0.00 C

ATOM 5109 O ILE X 339 -7.647 0.124 -19.097 0.00 0.00 O

ATOM 5110 N PHE X 340 -8.505 1.634 -17.698 0.00 0.00 N

ATOM 5111 HN PHE X 340 -8.317 2.348 -17.029 0.00 0.00 H

ATOM 5112 CA PHE X 340 -9.866 1.484 -18.079 0.00 0.00 C

ATOM 5113 HA PHE X 340 -10.216 0.470 -17.949 0.00 0.00 H

ATOM 5114 CB PHE X 340 -10.761 2.341 -17.104 0.00 0.00 C

ATOM 5115 HB1 PHE X 340 -10.514 3.424 -17.119 0.00 0.00 H

ATOM 5116 HB2 PHE X 340 -10.410 2.012 -16.106 0.00 0.00 H

ATOM 5117 CG PHE X 340 -12.287 2.224 -17.152 0.00 0.00 C

ATOM 5118 CD1 PHE X 340 -12.922 1.021 -17.010 0.00 0.00 C

ATOM 5119 HD1 PHE X 340 -12.388 0.082 -16.930 0.00 0.00 H

ATOM 5120 CE1 PHE X 340 -14.329 1.018 -16.892 0.00 0.00 C

ATOM 5121 HE1 PHE X 340 -14.763 0.062 -16.643 0.00 0.00 H

ATOM 5122 CZ PHE X 340 -15.029 2.180 -17.105 0.00 0.00 C

ATOM 5123 HZ PHE X 340 -16.101 2.202 -17.022 0.00 0.00 H

ATOM 5124 CD2 PHE X 340 -13.007 3.360 -17.538 0.00 0.00 C

ATOM 5125 HD2 PHE X 340 -12.543 4.326 -17.602 0.00 0.00 H

ATOM 5126 CE2 PHE X 340 -14.385 3.299 -17.452 0.00 0.00 C

ATOM 5127 HE2 PHE X 340 -14.982 4.192 -17.567 0.00 0.00 H

ATOM 5128 C PHE X 340 -10.215 1.871 -19.534 0.00 0.00 C

ATOM 5129 O PHE X 340 -11.106 1.238 -20.073 0.00 0.00 O

ATOM 5130 N GLN X 341 -9.693 2.893 -20.170 0.00 0.00 N

ATOM 5131 HN GLN X 341 -9.136 3.500 -19.615 0.00 0.00 H

ATOM 5132 CA GLN X 341 -9.861 3.306 -21.526 0.00 0.00 C

ATOM 5133 HA GLN X 341 -10.918 3.282 -21.761 0.00 0.00 H

ATOM 5134 CB GLN X 341 -9.391 4.745 -21.792 0.00 0.00 C

ATOM 5135 HB1 GLN X 341 -9.099 4.755 -22.860 0.00 0.00 H

ATOM 5136 HB2 GLN X 341 -8.459 4.811 -21.180 0.00 0.00 H

ATOM 5137 CG GLN X 341 -10.403 5.860 -21.533 0.00 0.00 C

ATOM 5138 HG1 GLN X 341 -9.761 6.771 -21.543 0.00 0.00 H

ATOM 5139 HG2 GLN X 341 -10.877 5.681 -20.544 0.00 0.00 H

ATOM 5140 CD GLN X 341 -11.537 5.949 -22.499 0.00 0.00 C

ATOM 5141 OE1 GLN X 341 -12.693 6.025 -22.066 0.00 0.00 O

ATOM 5142 NE2 GLN X 341 -11.321 5.954 -23.847 0.00 0.00 N

ATOM 5143 HE21 GLN X 341 -12.124 5.964 -24.449 0.00 0.00 H

ATOM 5144 HE22 GLN X 341 -10.391 5.790 -24.178 0.00 0.00 H

ATOM 5145 C GLN X 341 -9.263 2.300 -22.493 0.00 0.00 C

ATOM 5146 O GLN X 341 -9.749 2.010 -23.555 0.00 0.00 O

ATOM 5147 N ALA X 342 -8.090 1.724 -22.084 0.00 0.00 N

ATOM 5148 HN ALA X 342 -7.750 1.956 -21.176 0.00 0.00 H

ATOM 5149 CA ALA X 342 -7.408 0.619 -22.773 0.00 0.00 C

ATOM 5150 HA ALA X 342 -7.236 0.893 -23.801 0.00 0.00 H

ATOM 5151 CB ALA X 342 -6.063 0.322 -22.133 0.00 0.00 C

ATOM 5152 HB1 ALA X 342 -5.619 1.168 -21.576 0.00 0.00 H

ATOM 5153 HB2 ALA X 342 -5.262 -0.048 -22.808 0.00 0.00 H

ATOM 5154 HB3 ALA X 342 -6.203 -0.490 -21.389 0.00 0.00 H

ATOM 5155 C ALA X 342 -8.298 -0.614 -22.839 0.00 0.00 C

ATOM 5156 O ALA X 342 -8.377 -1.237 -23.895 0.00 0.00 O

ATOM 5157 N LEU X 343 -8.862 -0.983 -21.656 0.00 0.00 N

ATOM 5158 HN LEU X 343 -8.627 -0.537 -20.793 0.00 0.00 H

ATOM 5159 CA LEU X 343 -9.975 -1.940 -21.543 0.00 0.00 C

ATOM 5160 HA LEU X 343 -9.667 -2.943 -21.796 0.00 0.00 H

ATOM 5161 CB LEU X 343 -10.394 -2.004 -19.992 0.00 0.00 C

ATOM 5162 HB1 LEU X 343 -10.856 -1.021 -19.764 0.00 0.00 H

ATOM 5163 HB2 LEU X 343 -9.512 -2.121 -19.323 0.00 0.00 H

ATOM 5164 CG LEU X 343 -11.383 -3.066 -19.575 0.00 0.00 C

ATOM 5165 HG LEU X 343 -12.301 -2.939 -20.178 0.00 0.00 H

ATOM 5166 CD1 LEU X 343 -10.943 -4.483 -19.786 0.00 0.00 C

ATOM 5167 HD11 LEU X 343 -11.622 -5.269 -19.395 0.00 0.00 H

ATOM 5168 HD12 LEU X 343 -10.172 -4.660 -19.015 0.00 0.00 H

ATOM 5169 HD13 LEU X 343 -10.557 -4.671 -20.810 0.00 0.00 H

ATOM 5170 CD2 LEU X 343 -11.845 -2.823 -18.140 0.00 0.00 C

ATOM 5171 HD21 LEU X 343 -12.451 -1.908 -17.964 0.00 0.00 H

ATOM 5172 HD22 LEU X 343 -11.054 -2.780 -17.361 0.00 0.00 H

ATOM 5173 HD23 LEU X 343 -12.498 -3.628 -17.754 0.00 0.00 H

ATOM 5174 C LEU X 343 -11.212 -1.628 -22.505 0.00 0.00 C

ATOM 5175 O LEU X 343 -11.714 -2.498 -23.218 0.00 0.00 O

ATOM 5176 N CYS X 344 -11.694 -0.425 -22.587 0.00 0.00 N

ATOM 5177 HN CYS X 344 -11.389 0.238 -21.902 0.00 0.00 H

ATOM 5178 CA CYS X 344 -12.680 -0.050 -23.607 0.00 0.00 C

ATOM 5179 HA CYS X 344 -13.477 -0.733 -23.386 0.00 0.00 H

ATOM 5180 CB CYS X 344 -13.324 1.298 -23.263 0.00 0.00 C

ATOM 5181 HB1 CYS X 344 -14.281 1.407 -23.808 0.00 0.00 H

ATOM 5182 HB2 CYS X 344 -12.657 2.179 -23.349 0.00 0.00 H

ATOM 5183 SG CYS X 344 -13.801 1.448 -21.550 0.00 0.00 S

ATOM 5184 HG1 CYS X 344 -12.575 1.783 -21.197 0.00 0.00 H

ATOM 5185 C CYS X 344 -12.255 -0.196 -25.123 0.00 0.00 C

ATOM 5186 O CYS X 344 -13.010 -0.665 -25.921 0.00 0.00 O

ATOM 5187 N LYS X 345 -11.013 0.230 -25.409 0.00 0.00 N

ATOM 5188 HN LYS X 345 -10.573 0.801 -24.724 0.00 0.00 H

ATOM 5189 CA LYS X 345 -10.456 0.124 -26.753 0.00 0.00 C

ATOM 5190 HA LYS X 345 -10.975 0.827 -27.390 0.00 0.00 H

ATOM 5191 CB LYS X 345 -9.029 0.683 -26.624 0.00 0.00 C

ATOM 5192 HB1 LYS X 345 -8.381 0.322 -27.457 0.00 0.00 H

ATOM 5193 HB2 LYS X 345 -8.545 0.413 -25.666 0.00 0.00 H

ATOM 5194 CG LYS X 345 -9.013 2.278 -26.718 0.00 0.00 C

ATOM 5195 HG1 LYS X 345 -9.323 2.715 -25.743 0.00 0.00 H

ATOM 5196 HG2 LYS X 345 -9.775 2.598 -27.458 0.00 0.00 H

ATOM 5197 CD LYS X 345 -7.558 2.757 -26.944 0.00 0.00 C

ATOM 5198 HD1 LYS X 345 -7.595 3.868 -26.961 0.00 0.00 H

ATOM 5199 HD2 LYS X 345 -7.269 2.317 -27.932 0.00 0.00 H

ATOM 5200 CE LYS X 345 -6.521 2.271 -25.932 0.00 0.00 C

ATOM 5201 HE1 LYS X 345 -6.543 1.190 -25.681 0.00 0.00 H

ATOM 5202 HE2 LYS X 345 -6.645 2.752 -24.929 0.00 0.00 H

ATOM 5203 NZ LYS X 345 -5.240 2.607 -26.449 0.00 0.00 N1+

ATOM 5204 HZ1 LYS X 345 -5.200 3.611 -26.723 0.00 0.00 H

ATOM 5205 HZ2 LYS X 345 -5.041 2.094 -27.341 0.00 0.00 H

ATOM 5206 HZ3 LYS X 345 -4.443 2.528 -25.793 0.00 0.00 H

ATOM 5207 C LYS X 345 -10.427 -1.334 -27.288 0.00 0.00 C

ATOM 5208 O LYS X 345 -10.862 -1.581 -28.448 0.00 0.00 O

ATOM 5209 N ASP X 346 -9.957 -2.404 -26.531 0.00 0.00 N

ATOM 5210 HN ASP X 346 -9.330 -2.243 -25.767 0.00 0.00 H

ATOM 5211 CA ASP X 346 -10.045 -3.786 -26.993 0.00 0.00 C

ATOM 5212 HA ASP X 346 -9.689 -3.751 -28.015 0.00 0.00 H

ATOM 5213 CB ASP X 346 -9.148 -4.692 -26.149 0.00 0.00 C

ATOM 5214 HB1 ASP X 346 -9.194 -5.787 -26.324 0.00 0.00 H

ATOM 5215 HB2 ASP X 346 -9.484 -4.563 -25.096 0.00 0.00 H

ATOM 5216 CG ASP X 346 -7.737 -4.294 -26.143 0.00 0.00 C

ATOM 5217 OD1 ASP X 346 -7.215 -3.705 -27.113 0.00 0.00 O

ATOM 5218 OD2 ASP X 346 -7.066 -4.701 -25.155 0.00 0.00 O1-

ATOM 5219 C ASP X 346 -11.470 -4.350 -26.909 0.00 0.00 C

ATOM 5220 O ASP X 346 -11.901 -5.174 -27.718 0.00 0.00 O

ATOM 5221 N ASN X 347 -12.316 -3.810 -26.019 0.00 0.00 N

ATOM 5222 HN ASN X 347 -12.015 -2.976 -25.558 0.00 0.00 H

ATOM 5223 CA ASN X 347 -13.790 -3.869 -26.156 0.00 0.00 C

ATOM 5224 HA ASN X 347 -14.059 -3.091 -25.451 0.00 0.00 H

ATOM 5225 CB ASN X 347 -14.244 -3.474 -27.573 0.00 0.00 C

ATOM 5226 HB1 ASN X 347 -13.898 -4.198 -28.354 0.00 0.00 H

ATOM 5227 HB2 ASN X 347 -13.734 -2.536 -27.892 0.00 0.00 H

ATOM 5228 CG ASN X 347 -15.823 -3.377 -27.741 0.00 0.00 C

ATOM 5229 OD1 ASN X 347 -16.398 -4.077 -28.534 0.00 0.00 O

ATOM 5230 ND2 ASN X 347 -16.409 -2.705 -26.801 0.00 0.00 N

ATOM 5231 HD21 ASN X 347 -17.393 -2.805 -26.630 0.00 0.00 H

ATOM 5232 HD22 ASN X 347 -15.894 -2.193 -26.114 0.00 0.00 H

ATOM 5233 C ASN X 347 -14.401 -5.165 -25.582 0.00 0.00 C

ATOM 5234 O ASN X 347 -15.344 -5.784 -26.027 0.00 0.00 O

ATOM 5235 N ILE X 348 -13.743 -5.667 -24.519 0.00 0.00 N

ATOM 5236 HN ILE X 348 -12.975 -5.160 -24.126 0.00 0.00 H

ATOM 5237 CA ILE X 348 -14.043 -6.939 -23.782 0.00 0.00 C

ATOM 5238 HA ILE X 348 -14.132 -7.740 -24.505 0.00 0.00 H

ATOM 5239 CB ILE X 348 -13.056 -7.205 -22.598 0.00 0.00 C

ATOM 5240 HB ILE X 348 -13.132 -6.474 -21.768 0.00 0.00 H

ATOM 5241 CG2 ILE X 348 -13.451 -8.635 -22.113 0.00 0.00 C

ATOM 5242 HG21 ILE X 348 -13.469 -9.348 -22.975 0.00 0.00 H

ATOM 5243 HG22 ILE X 348 -14.453 -8.768 -21.659 0.00 0.00 H

ATOM 5244 HG23 ILE X 348 -12.743 -9.104 -21.400 0.00 0.00 H

ATOM 5245 CG1 ILE X 348 -11.605 -7.295 -23.160 0.00 0.00 C

ATOM 5246 HG11 ILE X 348 -11.620 -7.939 -24.066 0.00 0.00 H

ATOM 5247 HG12 ILE X 348 -10.958 -7.787 -22.411 0.00 0.00 H

ATOM 5248 CD ILE X 348 -10.987 -5.966 -23.443 0.00 0.00 C

ATOM 5249 HD1 ILE X 348 -9.875 -6.031 -23.508 0.00 0.00 H

ATOM 5250 HD2 ILE X 348 -11.330 -5.146 -22.778 0.00 0.00 H

ATOM 5251 HD3 ILE X 348 -11.188 -5.589 -24.467 0.00 0.00 H

ATOM 5252 C ILE X 348 -15.430 -6.787 -23.167 0.00 0.00 C

ATOM 5253 O ILE X 348 -16.263 -7.728 -23.246 0.00 0.00 O

ATOM 5254 N TYR X 349 -15.733 -5.605 -22.613 0.00 0.00 N

ATOM 5255 HN TYR X 349 -15.027 -4.904 -22.533 0.00 0.00 H

ATOM 5256 CA TYR X 349 -16.923 -5.368 -21.821 0.00 0.00 C

ATOM 5257 HA TYR X 349 -17.495 -6.267 -21.713 0.00 0.00 H

ATOM 5258 CB TYR X 349 -16.545 -4.954 -20.396 0.00 0.00 C

ATOM 5259 HB1 TYR X 349 -17.430 -4.763 -19.739 0.00 0.00 H

ATOM 5260 HB2 TYR X 349 -15.892 -4.060 -20.405 0.00 0.00 H

ATOM 5261 CG TYR X 349 -15.735 -6.069 -19.770 0.00 0.00 C

ATOM 5262 CD1 TYR X 349 -14.554 -5.728 -19.160 0.00 0.00 C

ATOM 5263 HD1 TYR X 349 -14.272 -4.680 -19.148 0.00 0.00 H

ATOM 5264 CE1 TYR X 349 -13.880 -6.662 -18.319 0.00 0.00 C

ATOM 5265 HE1 TYR X 349 -13.040 -6.334 -17.725 0.00 0.00 H

ATOM 5266 CZ TYR X 349 -14.300 -7.981 -18.216 0.00 0.00 C

ATOM 5267 OH TYR X 349 -13.661 -8.822 -17.234 0.00 0.00 O

ATOM 5268 HH TYR X 349 -13.857 -9.750 -17.395 0.00 0.00 H

ATOM 5269 CD2 TYR X 349 -16.132 -7.422 -19.673 0.00 0.00 C

ATOM 5270 HD2 TYR X 349 -17.122 -7.679 -19.996 0.00 0.00 H

ATOM 5271 CE2 TYR X 349 -15.486 -8.329 -18.857 0.00 0.00 C

ATOM 5272 HE2 TYR X 349 -15.929 -9.296 -18.720 0.00 0.00 H

ATOM 5273 C TYR X 349 -17.749 -4.266 -22.459 0.00 0.00 C

ATOM 5274 O TYR X 349 -17.681 -3.140 -22.002 0.00 0.00 O

ATOM 5275 N PRO X 350 -18.587 -4.545 -23.409 0.00 0.00 N

ATOM 5276 CD PRO X 350 -18.661 -5.859 -24.055 0.00 0.00 C

ATOM 5277 HD1 PRO X 350 -18.774 -6.753 -23.413 0.00 0.00 H

ATOM 5278 HD2 PRO X 350 -17.703 -6.008 -24.586 0.00 0.00 H

ATOM 5279 CA PRO X 350 -19.450 -3.570 -24.001 0.00 0.00 C

ATOM 5280 HA PRO X 350 -18.911 -2.679 -24.273 0.00 0.00 H

ATOM 5281 CB PRO X 350 -19.958 -4.280 -25.360 0.00 0.00 C

ATOM 5282 HB1 PRO X 350 -19.198 -4.113 -26.155 0.00 0.00 H

ATOM 5283 HB2 PRO X 350 -21.024 -4.042 -25.583 0.00 0.00 H

ATOM 5284 CG PRO X 350 -19.827 -5.767 -24.935 0.00 0.00 C

ATOM 5285 HG1 PRO X 350 -20.791 -5.876 -24.397 0.00 0.00 H

ATOM 5286 HG2 PRO X 350 -19.811 -6.555 -25.717 0.00 0.00 H

ATOM 5287 C PRO X 350 -20.588 -3.060 -23.141 0.00 0.00 C

ATOM 5288 O PRO X 350 -21.471 -2.351 -23.637 0.00 0.00 O

ATOM 5289 N ALA X 351 -20.593 -3.370 -21.861 0.00 0.00 N

ATOM 5290 HN ALA X 351 -20.124 -4.076 -21.359 0.00 0.00 H

ATOM 5291 CA ALA X 351 -21.374 -2.670 -20.833 0.00 0.00 C

ATOM 5292 HA ALA X 351 -22.310 -2.455 -21.322 0.00 0.00 H

ATOM 5293 CB ALA X 351 -21.500 -3.621 -19.584 0.00 0.00 C

ATOM 5294 HB1 ALA X 351 -20.515 -4.082 -19.355 0.00 0.00 H

ATOM 5295 HB2 ALA X 351 -22.164 -4.505 -19.709 0.00 0.00 H

ATOM 5296 HB3 ALA X 351 -21.873 -3.046 -18.705 0.00 0.00 H

ATOM 5297 C ALA X 351 -20.781 -1.375 -20.370 0.00 0.00 C

ATOM 5298 O ALA X 351 -21.450 -0.464 -19.919 0.00 0.00 O

ATOM 5299 N PHE X 352 -19.439 -1.221 -20.486 0.00 0.00 N

ATOM 5300 HN PHE X 352 -18.885 -1.887 -20.966 0.00 0.00 H

ATOM 5301 CA PHE X 352 -18.692 -0.100 -19.834 0.00 0.00 C

ATOM 5302 HA PHE X 352 -19.248 0.343 -19.027 0.00 0.00 H

ATOM 5303 CB PHE X 352 -17.360 -0.610 -19.191 0.00 0.00 C

ATOM 5304 HB1 PHE X 352 -16.843 0.158 -18.581 0.00 0.00 H

ATOM 5305 HB2 PHE X 352 -16.695 -0.981 -20.001 0.00 0.00 H

ATOM 5306 CG PHE X 352 -17.622 -1.753 -18.146 0.00 0.00 C

ATOM 5307 CD1 PHE X 352 -18.882 -1.950 -17.524 0.00 0.00 C

ATOM 5308 HD1 PHE X 352 -19.813 -1.407 -17.591 0.00 0.00 H

ATOM 5309 CE1 PHE X 352 -18.984 -3.030 -16.617 0.00 0.00 C

ATOM 5310 HE1 PHE X 352 -19.983 -3.197 -16.241 0.00 0.00 H

ATOM 5311 CZ PHE X 352 -17.877 -3.857 -16.300 0.00 0.00 C

ATOM 5312 HZ PHE X 352 -18.065 -4.631 -15.573 0.00 0.00 H

ATOM 5313 CD2 PHE X 352 -16.518 -2.593 -17.828 0.00 0.00 C

ATOM 5314 HD2 PHE X 352 -15.492 -2.487 -18.157 0.00 0.00 H

ATOM 5315 CE2 PHE X 352 -16.680 -3.654 -16.929 0.00 0.00 C

ATOM 5316 HE2 PHE X 352 -15.804 -4.279 -16.842 0.00 0.00 H

ATOM 5317 C PHE X 352 -18.553 1.087 -20.754 0.00 0.00 C

ATOM 5318 O PHE X 352 -17.722 1.980 -20.501 0.00 0.00 O

ATOM 5319 N GLN X 353 -19.212 1.044 -21.902 0.00 0.00 N

ATOM 5320 HN GLN X 353 -19.825 0.256 -21.927 0.00 0.00 H

ATOM 5321 CA GLN X 353 -19.214 2.043 -22.977 0.00 0.00 C

ATOM 5322 HA GLN X 353 -18.160 2.062 -23.196 0.00 0.00 H

ATOM 5323 CB GLN X 353 -19.972 1.640 -24.242 0.00 0.00 C

ATOM 5324 HB1 GLN X 353 -20.274 2.440 -24.954 0.00 0.00 H

ATOM 5325 HB2 GLN X 353 -20.930 1.141 -24.012 0.00 0.00 H

ATOM 5326 CG GLN X 353 -19.316 0.462 -25.061 0.00 0.00 C

ATOM 5327 HG1 GLN X 353 -19.924 0.176 -25.945 0.00 0.00 H

ATOM 5328 HG2 GLN X 353 -19.308 -0.434 -24.405 0.00 0.00 H

ATOM 5329 CD GLN X 353 -17.880 0.751 -25.437 0.00 0.00 C

ATOM 5330 OE1 GLN X 353 -17.038 -0.095 -25.118 0.00 0.00 O

ATOM 5331 NE2 GLN X 353 -17.836 1.807 -26.319 0.00 0.00 N

ATOM 5332 HE21 GLN X 353 -16.894 1.961 -26.600 0.00 0.00 H

ATOM 5333 HE22 GLN X 353 -18.673 2.156 -26.756 0.00 0.00 H

ATOM 5334 C GLN X 353 -19.603 3.413 -22.485 0.00 0.00 C

ATOM 5335 O GLN X 353 -18.996 4.409 -22.848 0.00 0.00 O

ATOM 5336 N MET X 354 -20.638 3.593 -21.622 0.00 0.00 N

ATOM 5337 HN MET X 354 -21.264 2.827 -21.519 0.00 0.00 H

ATOM 5338 CA MET X 354 -21.225 4.771 -21.129 0.00 0.00 C

ATOM 5339 HA MET X 354 -21.399 5.379 -22.005 0.00 0.00 H

ATOM 5340 CB MET X 354 -22.416 4.432 -20.289 0.00 0.00 C

ATOM 5341 HB1 MET X 354 -23.050 3.827 -20.983 0.00 0.00 H

ATOM 5342 HB2 MET X 354 -22.919 5.382 -20.004 0.00 0.00 H

ATOM 5343 CG MET X 354 -22.183 3.730 -18.967 0.00 0.00 C

ATOM 5344 HG1 MET X 354 -21.953 4.380 -18.098 0.00 0.00 H

ATOM 5345 HG2 MET X 354 -21.300 3.059 -19.068 0.00 0.00 H

ATOM 5346 SD MET X 354 -23.569 2.693 -18.475 0.00 0.00 S

ATOM 5347 CE MET X 354 -24.527 4.204 -18.145 0.00 0.00 C

ATOM 5348 HE1 MET X 354 -23.836 4.823 -17.536 0.00 0.00 H

ATOM 5349 HE2 MET X 354 -25.523 3.974 -17.725 0.00 0.00 H

ATOM 5350 HE3 MET X 354 -24.807 4.657 -19.123 0.00 0.00 H

ATOM 5351 C MET X 354 -19.995 5.547 -20.401 0.00 0.00 C

ATOM 5352 O MET X 354 -19.955 6.824 -20.552 0.00 0.00 O

ATOM 5353 N PHE X 355 -19.138 4.864 -19.597 0.00 0.00 N

ATOM 5354 HN PHE X 355 -18.982 3.886 -19.636 0.00 0.00 H

ATOM 5355 CA PHE X 355 -18.110 5.595 -18.838 0.00 0.00 C

ATOM 5356 HA PHE X 355 -18.470 6.597 -18.692 0.00 0.00 H

ATOM 5357 CB PHE X 355 -17.849 5.027 -17.471 0.00 0.00 C

ATOM 5358 HB1 PHE X 355 -17.617 5.870 -16.786 0.00 0.00 H

ATOM 5359 HB2 PHE X 355 -17.052 4.246 -17.514 0.00 0.00 H

ATOM 5360 CG PHE X 355 -19.044 4.417 -16.734 0.00 0.00 C

ATOM 5361 CD1 PHE X 355 -19.851 5.187 -15.855 0.00 0.00 C

ATOM 5362 HD1 PHE X 355 -19.649 6.226 -15.620 0.00 0.00 H

ATOM 5363 CE1 PHE X 355 -20.807 4.545 -15.101 0.00 0.00 C

ATOM 5364 HE1 PHE X 355 -21.334 5.207 -14.426 0.00 0.00 H

ATOM 5365 CZ PHE X 355 -21.003 3.170 -15.262 0.00 0.00 C

ATOM 5366 HZ PHE X 355 -21.704 2.712 -14.583 0.00 0.00 H

ATOM 5367 CD2 PHE X 355 -19.274 3.000 -16.857 0.00 0.00 C

ATOM 5368 HD2 PHE X 355 -18.719 2.522 -17.652 0.00 0.00 H

ATOM 5369 CE2 PHE X 355 -20.244 2.448 -16.089 0.00 0.00 C

ATOM 5370 HE2 PHE X 355 -20.391 1.379 -16.105 0.00 0.00 H

ATOM 5371 C PHE X 355 -16.780 5.727 -19.659 0.00 0.00 C

ATOM 5372 O PHE X 355 -15.790 6.196 -19.119 0.00 0.00 O

ATOM 5373 N ALA X 356 -16.763 5.249 -20.916 0.00 0.00 N

ATOM 5374 HN ALA X 356 -17.569 4.759 -21.264 0.00 0.00 H

ATOM 5375 CA ALA X 356 -15.720 5.470 -21.976 0.00 0.00 C

ATOM 5376 HA ALA X 356 -14.717 5.433 -21.572 0.00 0.00 H

ATOM 5377 CB ALA X 356 -15.832 4.362 -23.054 0.00 0.00 C

ATOM 5378 HB1 ALA X 356 -15.905 3.325 -22.671 0.00 0.00 H

ATOM 5379 HB2 ALA X 356 -14.951 4.532 -23.705 0.00 0.00 H

ATOM 5380 HB3 ALA X 356 -16.698 4.613 -23.700 0.00 0.00 H

ATOM 5381 C ALA X 356 -15.853 6.900 -22.539 0.00 0.00 C

ATOM 5382 O ALA X 356 -16.935 7.394 -22.761 0.00 0.00 O

ATOM 5383 N LYS X 357 -14.771 7.673 -22.866 0.00 0.00 N

ATOM 5384 HN LYS X 357 -13.909 7.180 -22.810 0.00 0.00 H

ATOM 5385 CA LYS X 357 -14.748 9.004 -23.506 0.00 0.00 C

ATOM 5386 HA LYS X 357 -15.175 9.835 -22.963 0.00 0.00 H

ATOM 5387 CB LYS X 357 -13.259 9.403 -23.773 0.00 0.00 C

ATOM 5388 HB1 LYS X 357 -12.708 8.605 -24.326 0.00 0.00 H

ATOM 5389 HB2 LYS X 357 -12.805 9.495 -22.772 0.00 0.00 H

ATOM 5390 CG LYS X 357 -12.998 10.670 -24.615 0.00 0.00 C

ATOM 5391 HG1 LYS X 357 -13.276 10.448 -25.669 0.00 0.00 H

ATOM 5392 HG2 LYS X 357 -11.892 10.660 -24.605 0.00 0.00 H

ATOM 5393 CD LYS X 357 -13.532 11.912 -23.919 0.00 0.00 C

ATOM 5394 HD1 LYS X 357 -12.922 11.976 -22.990 0.00 0.00 H

ATOM 5395 HD2 LYS X 357 -14.555 11.882 -23.495 0.00 0.00 H

ATOM 5396 CE LYS X 357 -13.380 13.195 -24.734 0.00 0.00 C

ATOM 5397 HE1 LYS X 357 -12.409 13.187 -25.273 0.00 0.00 H

ATOM 5398 HE2 LYS X 357 -13.437 14.093 -24.094 0.00 0.00 H

ATOM 5399 NZ LYS X 357 -14.440 13.375 -25.749 0.00 0.00 N1+

ATOM 5400 HZ1 LYS X 357 -15.329 12.976 -25.396 0.00 0.00 H

ATOM 5401 HZ2 LYS X 357 -14.221 12.789 -26.573 0.00 0.00 H

ATOM 5402 HZ3 LYS X 357 -14.572 14.379 -25.991 0.00 0.00 H

ATOM 5403 C LYS X 357 -15.494 9.103 -24.784 0.00 0.00 C

ATOM 5404 O LYS X 357 -15.318 8.341 -25.712 0.00 0.00 O

ATOM 5405 N GLY X 358 -16.480 10.068 -24.865 0.00 0.00 N

ATOM 5406 HN GLY X 358 -16.666 10.721 -24.142 0.00 0.00 H

ATOM 5407 CA GLY X 358 -17.170 10.315 -26.126 0.00 0.00 C

ATOM 5408 HA1 GLY X 358 -17.965 11.023 -25.917 0.00 0.00 H

ATOM 5409 HA2 GLY X 358 -17.621 9.407 -26.488 0.00 0.00 H

ATOM 5410 C GLY X 358 -16.382 11.076 -27.219 0.00 0.00 C

ATOM 5411 O GLY X 358 -15.160 11.272 -27.230 0.00 0.00 O

ATOM 5412 N TYR X 359 -17.185 11.568 -28.176 0.00 0.00 N

ATOM 5413 HN TYR X 359 -18.153 11.331 -28.115 0.00 0.00 H

ATOM 5414 CA TYR X 359 -16.673 12.317 -29.340 0.00 0.00 C

ATOM 5415 HA TYR X 359 -15.873 11.707 -29.736 0.00 0.00 H

ATOM 5416 CB TYR X 359 -17.670 12.491 -30.529 0.00 0.00 C

ATOM 5417 HB1 TYR X 359 -17.725 11.599 -31.191 0.00 0.00 H

ATOM 5418 HB2 TYR X 359 -17.378 13.258 -31.284 0.00 0.00 H

ATOM 5419 CG TYR X 359 -19.091 12.872 -30.104 0.00 0.00 C

ATOM 5420 CD1 TYR X 359 -19.345 14.032 -29.332 0.00 0.00 C

ATOM 5421 HD1 TYR X 359 -18.536 14.685 -29.045 0.00 0.00 H

ATOM 5422 CE1 TYR X 359 -20.678 14.562 -29.220 0.00 0.00 C

ATOM 5423 HE1 TYR X 359 -20.757 15.550 -28.790 0.00 0.00 H

ATOM 5424 CZ TYR X 359 -21.707 13.777 -29.583 0.00 0.00 C

ATOM 5425 OH TYR X 359 -22.968 14.292 -29.311 0.00 0.00 O

ATOM 5426 HH TYR X 359 -22.829 14.932 -28.602 0.00 0.00 H

ATOM 5427 CD2 TYR X 359 -20.219 12.127 -30.490 0.00 0.00 C

ATOM 5428 HD2 TYR X 359 -20.025 11.192 -31.002 0.00 0.00 H

ATOM 5429 CE2 TYR X 359 -21.506 12.693 -30.336 0.00 0.00 C

ATOM 5430 HE2 TYR X 359 -22.341 12.080 -30.648 0.00 0.00 H

ATOM 5431 C TYR X 359 -15.945 13.566 -28.990 0.00 0.00 C

ATOM 5432 O TYR X 359 -16.112 14.231 -27.934 0.00 0.00 O

ATOM 5433 N GLY X 360 -14.900 13.900 -29.765 0.00 0.00 N

ATOM 5434 HN GLY X 360 -14.827 13.485 -30.667 0.00 0.00 H

ATOM 5435 CA GLY X 360 -13.863 14.800 -29.410 0.00 0.00 C

ATOM 5436 HA1 GLY X 360 -14.199 15.415 -28.590 0.00 0.00 H

ATOM 5437 HA2 GLY X 360 -13.591 15.408 -30.257 0.00 0.00 H

ATOM 5438 C GLY X 360 -12.716 13.981 -29.049 0.00 0.00 C

ATOM 5439 O GLY X 360 -12.757 12.985 -28.271 0.00 0.00 O

ATOM 5440 N LYS X 361 -11.564 14.394 -29.696 0.00 0.00 N

ATOM 5441 HN LYS X 361 -11.605 15.191 -30.288 0.00 0.00 H

ATOM 5442 CA LYS X 361 -10.276 13.670 -29.508 0.00 0.00 C

ATOM 5443 HA LYS X 361 -10.333 12.722 -28.997 0.00 0.00 H

ATOM 5444 CB LYS X 361 -9.693 13.266 -30.940 0.00 0.00 C

ATOM 5445 HB1 LYS X 361 -8.862 12.570 -30.712 0.00 0.00 H

ATOM 5446 HB2 LYS X 361 -9.179 14.139 -31.395 0.00 0.00 H

ATOM 5447 CG LYS X 361 -10.498 12.489 -31.883 0.00 0.00 C

ATOM 5448 HG1 LYS X 361 -11.540 12.870 -31.891 0.00 0.00 H

ATOM 5449 HG2 LYS X 361 -10.719 11.475 -31.497 0.00 0.00 H

ATOM 5450 CD LYS X 361 -9.859 12.476 -33.214 0.00 0.00 C

ATOM 5451 HD1 LYS X 361 -10.322 11.641 -33.773 0.00 0.00 H

ATOM 5452 HD2 LYS X 361 -8.784 12.190 -33.208 0.00 0.00 H

ATOM 5453 CE LYS X 361 -10.100 13.714 -34.028 0.00 0.00 C

ATOM 5454 HE1 LYS X 361 -9.627 13.518 -35.012 0.00 0.00 H

ATOM 5455 HE2 LYS X 361 -9.655 14.621 -33.566 0.00 0.00 H

ATOM 5456 NZ LYS X 361 -11.585 13.969 -34.298 0.00 0.00 N1+

ATOM 5457 HZ1 LYS X 361 -12.096 14.514 -33.583 0.00 0.00 H

ATOM 5458 HZ2 LYS X 361 -12.090 13.072 -34.495 0.00 0.00 H

ATOM 5459 HZ3 LYS X 361 -11.601 14.540 -35.164 0.00 0.00 H

ATOM 5460 C LYS X 361 -9.194 14.540 -28.792 0.00 0.00 C

ATOM 5461 O LYS X 361 -9.373 15.769 -28.581 0.00 0.00 O

ATOM 5462 N ASN X 362 -8.079 13.874 -28.404 0.00 0.00 N

ATOM 5463 HN ASN X 362 -8.058 12.895 -28.579 0.00 0.00 H

ATOM 5464 CA ASN X 362 -6.935 14.524 -27.647 0.00 0.00 C

ATOM 5465 HA ASN X 362 -6.382 13.630 -27.417 0.00 0.00 H

ATOM 5466 CB ASN X 362 -6.225 15.645 -28.300 0.00 0.00 C

ATOM 5467 HB1 ASN X 362 -5.359 15.856 -27.634 0.00 0.00 H

ATOM 5468 HB2 ASN X 362 -6.787 16.595 -28.434 0.00 0.00 H

ATOM 5469 CG ASN X 362 -5.733 15.149 -29.614 0.00 0.00 C

ATOM 5470 OD1 ASN X 362 -4.869 14.345 -29.708 0.00 0.00 O

ATOM 5471 ND2 ASN X 362 -6.206 15.809 -30.698 0.00 0.00 N

ATOM 5472 HD21 ASN X 362 -6.816 16.592 -30.574 0.00 0.00 H

ATOM 5473 HD22 ASN X 362 -5.666 15.678 -31.527 0.00 0.00 H

ATOM 5474 C ASN X 362 -7.195 14.853 -26.125 0.00 0.00 C

ATOM 5475 O ASN X 362 -6.382 14.535 -25.269 0.00 0.00 O

ATOM 5476 N ASN X 363 -8.361 15.326 -25.749 0.00 0.00 N

ATOM 5477 HN ASN X 363 -8.978 15.523 -26.505 0.00 0.00 H

ATOM 5478 CA ASN X 363 -8.990 15.724 -24.483 0.00 0.00 C

ATOM 5479 HA ASN X 363 -8.305 16.417 -24.001 0.00 0.00 H

ATOM 5480 CB ASN X 363 -10.423 16.212 -24.622 0.00 0.00 C

ATOM 5481 HB1 ASN X 363 -10.960 16.469 -23.679 0.00 0.00 H

ATOM 5482 HB2 ASN X 363 -11.043 15.584 -25.293 0.00 0.00 H

ATOM 5483 CG ASN X 363 -10.407 17.505 -25.502 0.00 0.00 C

ATOM 5484 OD1 ASN X 363 -10.239 17.604 -26.720 0.00 0.00 O

ATOM 5485 ND2 ASN X 363 -10.838 18.611 -24.786 0.00 0.00 N

ATOM 5486 HD21 ASN X 363 -11.121 18.580 -23.827 0.00 0.00 H

ATOM 5487 HD22 ASN X 363 -10.863 19.451 -25.328 0.00 0.00 H

ATOM 5488 C ASN X 363 -9.170 14.520 -23.485 0.00 0.00 C

ATOM 5489 O ASN X 363 -9.380 13.388 -23.917 0.00 0.00 O

ATOM 5490 N GLU X 364 -9.002 14.862 -22.179 0.00 0.00 N

ATOM 5491 HN GLU X 364 -8.942 15.852 -22.057 0.00 0.00 H

ATOM 5492 CA GLU X 364 -8.985 13.886 -21.014 0.00 0.00 C

ATOM 5493 HA GLU X 364 -8.066 13.344 -21.140 0.00 0.00 H

ATOM 5494 CB GLU X 364 -9.105 14.711 -19.731 0.00 0.00 C

ATOM 5495 HB1 GLU X 364 -9.435 14.063 -18.887 0.00 0.00 H

ATOM 5496 HB2 GLU X 364 -9.965 15.419 -19.821 0.00 0.00 H

ATOM 5497 CG GLU X 364 -7.815 15.482 -19.329 0.00 0.00 C

ATOM 5498 HG1 GLU X 364 -8.002 15.932 -18.330 0.00 0.00 H

ATOM 5499 HG2 GLU X 364 -7.596 16.332 -20.016 0.00 0.00 H

ATOM 5500 CD GLU X 364 -6.556 14.583 -19.303 0.00 0.00 C

ATOM 5501 OE1 GLU X 364 -5.833 14.646 -20.317 0.00 0.00 O

ATOM 5502 OE2 GLU X 364 -6.330 13.837 -18.362 0.00 0.00 O1-

ATOM 5503 C GLU X 364 -10.179 12.990 -21.034 0.00 0.00 C

ATOM 5504 O GLU X 364 -11.319 13.357 -21.455 0.00 0.00 O

ATOM 5505 N PRO X 365 -10.067 11.777 -20.548 0.00 0.00 N

ATOM 5506 CD PRO X 365 -8.812 11.014 -20.391 0.00 0.00 C

ATOM 5507 HD1 PRO X 365 -8.059 11.541 -19.779 0.00 0.00 H

ATOM 5508 HD2 PRO X 365 -8.322 10.830 -21.374 0.00 0.00 H

ATOM 5509 CA PRO X 365 -11.281 11.064 -20.109 0.00 0.00 C

ATOM 5510 HA PRO X 365 -12.103 11.270 -20.777 0.00 0.00 H

ATOM 5511 CB PRO X 365 -10.737 9.649 -20.278 0.00 0.00 C

ATOM 5512 HB1 PRO X 365 -10.914 9.405 -21.350 0.00 0.00 H

ATOM 5513 HB2 PRO X 365 -11.346 8.938 -19.683 0.00 0.00 H

ATOM 5514 CG PRO X 365 -9.286 9.764 -19.795 0.00 0.00 C

ATOM 5515 HG1 PRO X 365 -9.332 9.924 -18.692 0.00 0.00 H

ATOM 5516 HG2 PRO X 365 -8.744 8.874 -20.181 0.00 0.00 H

ATOM 5517 C PRO X 365 -11.740 11.407 -18.660 0.00 0.00 C

ATOM 5518 O PRO X 365 -11.469 10.667 -17.714 0.00 0.00 O

ATOM 5519 N LEU X 366 -12.530 12.468 -18.645 0.00 0.00 N

ATOM 5520 HN LEU X 366 -12.637 12.941 -19.515 0.00 0.00 H

ATOM 5521 CA LEU X 366 -13.254 12.868 -17.430 0.00 0.00 C

ATOM 5522 HA LEU X 366 -12.598 13.071 -16.595 0.00 0.00 H

ATOM 5523 CB LEU X 366 -13.976 14.228 -17.498 0.00 0.00 C

ATOM 5524 HB1 LEU X 366 -14.707 14.410 -16.683 0.00 0.00 H

ATOM 5525 HB2 LEU X 366 -14.586 14.200 -18.430 0.00 0.00 H

ATOM 5526 CG LEU X 366 -12.916 15.339 -17.466 0.00 0.00 C

ATOM 5527 HG LEU X 366 -12.028 14.875 -17.933 0.00 0.00 H

ATOM 5528 CD1 LEU X 366 -13.407 16.550 -18.235 0.00 0.00 C

ATOM 5529 HD11 LEU X 366 -12.596 17.267 -18.457 0.00 0.00 H

ATOM 5530 HD12 LEU X 366 -14.129 17.209 -17.693 0.00 0.00 H

ATOM 5531 HD13 LEU X 366 -14.022 16.237 -19.108 0.00 0.00 H

ATOM 5532 CD2 LEU X 366 -12.524 15.735 -16.069 0.00 0.00 C

ATOM 5533 HD21 LEU X 366 -13.229 16.425 -15.556 0.00 0.00 H

ATOM 5534 HD22 LEU X 366 -11.541 16.257 -16.157 0.00 0.00 H

ATOM 5535 HD23 LEU X 366 -12.322 14.873 -15.405 0.00 0.00 H

ATOM 5536 C LEU X 366 -14.265 11.850 -16.938 0.00 0.00 C

ATOM 5537 O LEU X 366 -14.313 11.574 -15.736 0.00 0.00 O

ATOM 5538 N ARG X 367 -14.992 11.143 -17.747 0.00 0.00 N

ATOM 5539 HN ARG X 367 -14.860 11.290 -18.720 0.00 0.00 H

ATOM 5540 CA ARG X 367 -15.835 10.059 -17.495 0.00 0.00 C

ATOM 5541 HA ARG X 367 -16.664 10.501 -16.959 0.00 0.00 H

ATOM 5542 CB ARG X 367 -16.416 9.323 -18.780 0.00 0.00 C

ATOM 5543 HB1 ARG X 367 -17.014 8.446 -18.474 0.00 0.00 H

ATOM 5544 HB2 ARG X 367 -15.525 9.011 -19.377 0.00 0.00 H

ATOM 5545 CG ARG X 367 -17.331 10.204 -19.633 0.00 0.00 C

ATOM 5546 HG1 ARG X 367 -17.098 11.284 -19.770 0.00 0.00 H

ATOM 5547 HG2 ARG X 367 -18.305 10.327 -19.123 0.00 0.00 H

ATOM 5548 CD ARG X 367 -17.576 9.457 -20.951 0.00 0.00 C

ATOM 5549 HD1 ARG X 367 -17.992 8.460 -20.729 0.00 0.00 H

ATOM 5550 HD2 ARG X 367 -16.609 9.349 -21.494 0.00 0.00 H

ATOM 5551 NE ARG X 367 -18.477 10.382 -21.726 0.00 0.00 N

ATOM 5552 HE ARG X 367 -18.215 11.332 -21.892 0.00 0.00 H

ATOM 5553 CZ ARG X 367 -19.278 9.850 -22.665 0.00 0.00 C

ATOM 5554 NH1 ARG X 367 -19.820 8.645 -22.634 0.00 0.00 N1+

ATOM 5555 HH11 ARG X 367 -19.558 7.914 -22.013 0.00 0.00 H

ATOM 5556 HH12 ARG X 367 -20.338 8.345 -23.437 0.00 0.00 H

ATOM 5557 NH2 ARG X 367 -19.632 10.575 -23.670 0.00 0.00 N

ATOM 5558 HH21 ARG X 367 -19.421 11.545 -23.802 0.00 0.00 H

ATOM 5559 HH22 ARG X 367 -20.344 10.221 -24.278 0.00 0.00 H

ATOM 5560 C ARG X 367 -15.200 8.973 -16.517 0.00 0.00 C

ATOM 5561 O ARG X 367 -15.830 8.631 -15.524 0.00 0.00 O

ATOM 5562 N GLY X 368 -13.979 8.526 -16.888 0.00 0.00 N

ATOM 5563 HN GLY X 368 -13.578 8.819 -17.751 0.00 0.00 H

ATOM 5564 CA GLY X 368 -13.090 7.743 -16.084 0.00 0.00 C

ATOM 5565 HA1 GLY X 368 -12.257 7.684 -16.765 0.00 0.00 H

ATOM 5566 HA2 GLY X 368 -13.556 6.816 -15.811 0.00 0.00 H

ATOM 5567 C GLY X 368 -12.499 8.380 -14.862 0.00 0.00 C

ATOM 5568 O GLY X 368 -12.426 7.695 -13.871 0.00 0.00 O

ATOM 5569 N TYR X 369 -11.982 9.654 -14.880 0.00 0.00 N

ATOM 5570 HN TYR X 369 -11.798 10.040 -15.773 0.00 0.00 H

ATOM 5571 CA TYR X 369 -11.584 10.304 -13.599 0.00 0.00 C

ATOM 5572 HA TYR X 369 -10.758 9.755 -13.160 0.00 0.00 H

ATOM 5573 CB TYR X 369 -11.100 11.823 -13.607 0.00 0.00 C

ATOM 5574 HB1 TYR X 369 -11.004 12.282 -12.599 0.00 0.00 H

ATOM 5575 HB2 TYR X 369 -11.937 12.388 -14.059 0.00 0.00 H

ATOM 5576 CG TYR X 369 -9.845 12.070 -14.293 0.00 0.00 C

ATOM 5577 CD1 TYR X 369 -8.658 11.991 -13.503 0.00 0.00 C

ATOM 5578 HD1 TYR X 369 -8.739 11.744 -12.457 0.00 0.00 H

ATOM 5579 CE1 TYR X 369 -7.483 12.160 -14.182 0.00 0.00 C

ATOM 5580 HE1 TYR X 369 -6.540 11.988 -13.685 0.00 0.00 H

ATOM 5581 CZ TYR X 369 -7.415 12.337 -15.588 0.00 0.00 C

ATOM 5582 OH TYR X 369 -6.163 12.364 -16.179 0.00 0.00 O

ATOM 5583 HH TYR X 369 -6.350 12.893 -16.960 0.00 0.00 H

ATOM 5584 CD2 TYR X 369 -9.825 12.279 -15.666 0.00 0.00 C

ATOM 5585 HD2 TYR X 369 -10.766 12.414 -16.181 0.00 0.00 H

ATOM 5586 CE2 TYR X 369 -8.577 12.432 -16.376 0.00 0.00 C

ATOM 5587 HE2 TYR X 369 -8.521 12.539 -17.446 0.00 0.00 H

ATOM 5588 C TYR X 369 -12.649 10.331 -12.558 0.00 0.00 C

ATOM 5589 O TYR X 369 -12.440 9.951 -11.421 0.00 0.00 O

ATOM 5590 N ILE X 370 -13.870 10.661 -13.026 0.00 0.00 N

ATOM 5591 HN ILE X 370 -14.031 10.831 -13.992 0.00 0.00 H

ATOM 5592 CA ILE X 370 -15.034 10.765 -12.182 0.00 0.00 C

ATOM 5593 HA ILE X 370 -14.789 11.473 -11.396 0.00 0.00 H

ATOM 5594 CB ILE X 370 -16.296 11.411 -12.877 0.00 0.00 C

ATOM 5595 HB ILE X 370 -16.648 10.948 -13.822 0.00 0.00 H

ATOM 5596 CG2 ILE X 370 -17.511 11.446 -11.842 0.00 0.00 C

ATOM 5597 HG21 ILE X 370 -17.121 11.667 -10.826 0.00 0.00 H

ATOM 5598 HG22 ILE X 370 -17.955 10.434 -11.773 0.00 0.00 H

ATOM 5599 HG23 ILE X 370 -18.313 12.131 -12.207 0.00 0.00 H

ATOM 5600 CG1 ILE X 370 -15.991 12.881 -13.344 0.00 0.00 C

ATOM 5601 HG11 ILE X 370 -16.853 13.280 -13.908 0.00 0.00 H

ATOM 5602 HG12 ILE X 370 -15.186 12.925 -14.106 0.00 0.00 H

ATOM 5603 CD ILE X 370 -15.666 13.859 -12.134 0.00 0.00 C

ATOM 5604 HD1 ILE X 370 -15.717 14.940 -12.382 0.00 0.00 H

ATOM 5605 HD2 ILE X 370 -14.655 13.648 -11.717 0.00 0.00 H

ATOM 5606 HD3 ILE X 370 -16.312 13.571 -11.278 0.00 0.00 H

ATOM 5607 C ILE X 370 -15.472 9.485 -11.449 0.00 0.00 C

ATOM 5608 O ILE X 370 -15.890 9.491 -10.275 0.00 0.00 O

ATOM 5609 N LEU X 371 -15.347 8.351 -12.197 0.00 0.00 N

ATOM 5610 HN LEU X 371 -15.110 8.362 -13.170 0.00 0.00 H

ATOM 5611 CA LEU X 371 -15.701 7.012 -11.773 0.00 0.00 C

ATOM 5612 HA LEU X 371 -16.614 7.038 -11.209 0.00 0.00 H

ATOM 5613 CB LEU X 371 -15.894 6.011 -12.908 0.00 0.00 C

ATOM 5614 HB1 LEU X 371 -14.974 5.738 -13.454 0.00 0.00 H

ATOM 5615 HB2 LEU X 371 -16.676 6.410 -13.594 0.00 0.00 H

ATOM 5616 CG LEU X 371 -16.444 4.633 -12.433 0.00 0.00 C

ATOM 5617 HG LEU X 371 -15.686 4.114 -11.801 0.00 0.00 H

ATOM 5618 CD1 LEU X 371 -17.648 4.739 -11.553 0.00 0.00 C

ATOM 5619 HD11 LEU X 371 -18.139 3.753 -11.410 0.00 0.00 H

ATOM 5620 HD12 LEU X 371 -18.490 5.147 -12.146 0.00 0.00 H

ATOM 5621 HD13 LEU X 371 -17.488 5.270 -10.593 0.00 0.00 H

ATOM 5622 CD2 LEU X 371 -16.844 3.821 -13.652 0.00 0.00 C

ATOM 5623 HD21 LEU X 371 -15.942 3.410 -14.151 0.00 0.00 H

ATOM 5624 HD22 LEU X 371 -17.337 4.478 -14.395 0.00 0.00 H

ATOM 5625 HD23 LEU X 371 -17.462 2.965 -13.280 0.00 0.00 H

ATOM 5626 C LEU X 371 -14.628 6.542 -10.728 0.00 0.00 C

ATOM 5627 O LEU X 371 -15.051 6.030 -9.682 0.00 0.00 O

ATOM 5628 N THR X 372 -13.308 6.855 -11.068 0.00 0.00 N

ATOM 5629 HN THR X 372 -13.113 7.260 -11.961 0.00 0.00 H

ATOM 5630 CA THR X 372 -12.152 6.631 -10.131 0.00 0.00 C

ATOM 5631 HA THR X 372 -12.221 5.581 -9.867 0.00 0.00 H

ATOM 5632 CB THR X 372 -10.862 7.038 -10.784 0.00 0.00 C

ATOM 5633 HB THR X 372 -10.745 8.143 -10.884 0.00 0.00 H

ATOM 5634 OG1 THR X 372 -10.706 6.430 -12.077 0.00 0.00 O

ATOM 5635 HG1 THR X 372 -11.291 6.954 -12.639 0.00 0.00 H

ATOM 5636 CG2 THR X 372 -9.631 6.693 -9.951 0.00 0.00 C

ATOM 5637 HG21 THR X 372 -9.628 7.128 -8.931 0.00 0.00 H

ATOM 5638 HG22 THR X 372 -8.652 7.067 -10.302 0.00 0.00 H

ATOM 5639 HG23 THR X 372 -9.502 5.587 -9.904 0.00 0.00 H

ATOM 5640 C THR X 372 -12.375 7.504 -8.883 0.00 0.00 C

ATOM 5641 O THR X 372 -12.258 6.944 -7.814 0.00 0.00 O

ATOM 5642 N PHE X 373 -12.797 8.754 -9.092 0.00 0.00 N

ATOM 5643 HN PHE X 373 -12.838 9.118 -10.018 0.00 0.00 H

ATOM 5644 CA PHE X 373 -13.150 9.551 -8.024 0.00 0.00 C

ATOM 5645 HA PHE X 373 -12.330 9.541 -7.329 0.00 0.00 H

ATOM 5646 CB PHE X 373 -13.417 10.996 -8.533 0.00 0.00 C

ATOM 5647 HB1 PHE X 373 -14.177 10.945 -9.348 0.00 0.00 H

ATOM 5648 HB2 PHE X 373 -12.544 11.517 -8.976 0.00 0.00 H

ATOM 5649 CG PHE X 373 -14.017 11.819 -7.483 0.00 0.00 C

ATOM 5650 CD1 PHE X 373 -13.151 12.647 -6.728 0.00 0.00 C

ATOM 5651 HD1 PHE X 373 -12.083 12.586 -6.900 0.00 0.00 H

ATOM 5652 CE1 PHE X 373 -13.531 13.582 -5.753 0.00 0.00 C

ATOM 5653 HE1 PHE X 373 -12.850 14.201 -5.185 0.00 0.00 H

ATOM 5654 CZ PHE X 373 -14.939 13.508 -5.448 0.00 0.00 C

ATOM 5655 HZ PHE X 373 -15.359 14.242 -4.766 0.00 0.00 H

ATOM 5656 CD2 PHE X 373 -15.450 11.985 -7.247 0.00 0.00 C

ATOM 5657 HD2 PHE X 373 -16.123 11.413 -7.868 0.00 0.00 H

ATOM 5658 CE2 PHE X 373 -15.815 12.834 -6.174 0.00 0.00 C

ATOM 5659 HE2 PHE X 373 -16.866 12.901 -5.961 0.00 0.00 H

ATOM 5660 C PHE X 373 -14.372 9.019 -7.114 0.00 0.00 C

ATOM 5661 O PHE X 373 -14.353 9.091 -5.907 0.00 0.00 O

ATOM 5662 N LEU X 374 -15.376 8.388 -7.797 0.00 0.00 N

ATOM 5663 HN LEU X 374 -15.416 8.452 -8.793 0.00 0.00 H

ATOM 5664 CA LEU X 374 -16.574 7.795 -7.183 0.00 0.00 C

ATOM 5665 HA LEU X 374 -16.946 8.610 -6.582 0.00 0.00 H

ATOM 5666 CB LEU X 374 -17.682 7.344 -8.151 0.00 0.00 C

ATOM 5667 HB1 LEU X 374 -17.480 6.313 -8.524 0.00 0.00 H

ATOM 5668 HB2 LEU X 374 -17.610 7.952 -9.080 0.00 0.00 H

ATOM 5669 CG LEU X 374 -19.122 7.423 -7.638 0.00 0.00 C

ATOM 5670 HG LEU X 374 -19.206 8.434 -7.190 0.00 0.00 H

ATOM 5671 CD1 LEU X 374 -20.156 7.536 -8.736 0.00 0.00 C

ATOM 5672 HD11 LEU X 374 -21.170 7.388 -8.323 0.00 0.00 H

ATOM 5673 HD12 LEU X 374 -19.973 6.827 -9.576 0.00 0.00 H

ATOM 5674 HD13 LEU X 374 -20.036 8.530 -9.212 0.00 0.00 H

ATOM 5675 CD2 LEU X 374 -19.490 6.418 -6.573 0.00 0.00 C

ATOM 5676 HD21 LEU X 374 -19.226 5.372 -6.827 0.00 0.00 H

ATOM 5677 HD22 LEU X 374 -20.545 6.647 -6.300 0.00 0.00 H

ATOM 5678 HD23 LEU X 374 -18.866 6.700 -5.707 0.00 0.00 H

ATOM 5679 C LEU X 374 -16.189 6.624 -6.307 0.00 0.00 C

ATOM 5680 O LEU X 374 -16.408 6.548 -5.106 0.00 0.00 O

ATOM 5681 N ILE X 375 -15.426 5.690 -6.847 0.00 0.00 N

ATOM 5682 HN ILE X 375 -15.330 5.961 -7.802 0.00 0.00 H

ATOM 5683 CA ILE X 375 -14.873 4.520 -6.346 0.00 0.00 C

ATOM 5684 HA ILE X 375 -15.653 3.900 -5.926 0.00 0.00 H

ATOM 5685 CB ILE X 375 -14.297 3.572 -7.400 0.00 0.00 C

ATOM 5686 HB ILE X 375 -13.643 4.170 -8.064 0.00 0.00 H

ATOM 5687 CG2 ILE X 375 -13.295 2.523 -6.868 0.00 0.00 C

ATOM 5688 HG21 ILE X 375 -12.529 3.109 -6.329 0.00 0.00 H

ATOM 5689 HG22 ILE X 375 -12.774 1.839 -7.582 0.00 0.00 H

ATOM 5690 HG23 ILE X 375 -13.890 1.825 -6.251 0.00 0.00 H

ATOM 5691 CG1 ILE X 375 -15.428 2.996 -8.372 0.00 0.00 C

ATOM 5692 HG11 ILE X 375 -15.067 2.720 -9.393 0.00 0.00 H

ATOM 5693 HG12 ILE X 375 -16.222 3.769 -8.490 0.00 0.00 H

ATOM 5694 CD ILE X 375 -16.056 1.698 -7.901 0.00 0.00 C

ATOM 5695 HD1 ILE X 375 -16.972 1.352 -8.431 0.00 0.00 H

ATOM 5696 HD2 ILE X 375 -16.396 1.798 -6.856 0.00 0.00 H

ATOM 5697 HD3 ILE X 375 -15.264 0.930 -8.005 0.00 0.00 H

ATOM 5698 C ILE X 375 -13.965 4.746 -5.163 0.00 0.00 C

ATOM 5699 O ILE X 375 -13.910 3.984 -4.209 0.00 0.00 O

ATOM 5700 N ALA X 376 -13.120 5.814 -5.283 0.00 0.00 N

ATOM 5701 HN ALA X 376 -13.025 6.394 -6.087 0.00 0.00 H

ATOM 5702 CA ALA X 376 -12.084 6.156 -4.305 0.00 0.00 C

ATOM 5703 HA ALA X 376 -11.672 5.199 -4.036 0.00 0.00 H

ATOM 5704 CB ALA X 376 -11.243 7.265 -4.983 0.00 0.00 C

ATOM 5705 HB1 ALA X 376 -10.543 6.818 -5.724 0.00 0.00 H

ATOM 5706 HB2 ALA X 376 -10.642 7.883 -4.293 0.00 0.00 H

ATOM 5707 HB3 ALA X 376 -11.881 7.927 -5.601 0.00 0.00 H

ATOM 5708 C ALA X 376 -12.870 6.705 -3.117 0.00 0.00 C

ATOM 5709 O ALA X 376 -12.627 6.341 -1.947 0.00 0.00 O

ATOM 5710 N LEU X 377 -13.878 7.558 -3.374 0.00 0.00 N

ATOM 5711 HN LEU X 377 -14.073 7.546 -4.353 0.00 0.00 H

ATOM 5712 CA LEU X 377 -14.791 8.342 -2.498 0.00 0.00 C

ATOM 5713 HA LEU X 377 -14.235 8.879 -1.737 0.00 0.00 H

ATOM 5714 CB LEU X 377 -15.701 9.420 -3.253 0.00 0.00 C

ATOM 5715 HB1 LEU X 377 -16.032 8.715 -4.039 0.00 0.00 H

ATOM 5716 HB2 LEU X 377 -15.020 10.158 -3.742 0.00 0.00 H

ATOM 5717 CG LEU X 377 -16.760 10.152 -2.571 0.00 0.00 C

ATOM 5718 HG LEU X 377 -17.228 9.372 -1.935 0.00 0.00 H

ATOM 5719 CD1 LEU X 377 -16.285 11.301 -1.547 0.00 0.00 C

ATOM 5720 HD11 LEU X 377 -15.661 12.118 -1.976 0.00 0.00 H

ATOM 5721 HD12 LEU X 377 -15.638 10.810 -0.788 0.00 0.00 H

ATOM 5722 HD13 LEU X 377 -17.236 11.703 -1.143 0.00 0.00 H

ATOM 5723 CD2 LEU X 377 -17.868 10.694 -3.625 0.00 0.00 C

ATOM 5724 HD21 LEU X 377 -17.534 11.529 -4.269 0.00 0.00 H

ATOM 5725 HD22 LEU X 377 -18.728 11.142 -3.063 0.00 0.00 H

ATOM 5726 HD23 LEU X 377 -18.195 9.823 -4.228 0.00 0.00 H

ATOM 5727 C LEU X 377 -15.493 7.382 -1.593 0.00 0.00 C

ATOM 5728 O LEU X 377 -15.637 7.571 -0.412 0.00 0.00 O

ATOM 5729 N GLY X 378 -15.989 6.232 -2.085 0.00 0.00 N

ATOM 5730 HN GLY X 378 -16.065 6.080 -3.071 0.00 0.00 H

ATOM 5731 CA GLY X 378 -16.716 5.336 -1.299 0.00 0.00 C

ATOM 5732 HA1 GLY X 378 -17.135 4.487 -1.822 0.00 0.00 H

ATOM 5733 HA2 GLY X 378 -17.450 5.986 -0.847 0.00 0.00 H

ATOM 5734 C GLY X 378 -15.857 4.710 -0.175 0.00 0.00 C

ATOM 5735 O GLY X 378 -16.255 4.653 0.975 0.00 0.00 O

ATOM 5736 N PHE X 379 -14.572 4.412 -0.479 0.00 0.00 N

ATOM 5737 HN PHE X 379 -14.183 4.532 -1.392 0.00 0.00 H

ATOM 5738 CA PHE X 379 -13.719 3.948 0.532 0.00 0.00 C

ATOM 5739 HA PHE X 379 -14.012 3.156 1.208 0.00 0.00 H

ATOM 5740 CB PHE X 379 -12.438 3.343 -0.044 0.00 0.00 C

ATOM 5741 HB1 PHE X 379 -11.706 3.132 0.755 0.00 0.00 H

ATOM 5742 HB2 PHE X 379 -12.019 4.063 -0.792 0.00 0.00 H

ATOM 5743 CG PHE X 379 -12.840 2.015 -0.696 0.00 0.00 C

ATOM 5744 CD1 PHE X 379 -13.198 0.922 0.205 0.00 0.00 C

ATOM 5745 HD1 PHE X 379 -13.043 1.111 1.258 0.00 0.00 H

ATOM 5746 CE1 PHE X 379 -13.569 -0.269 -0.311 0.00 0.00 C

ATOM 5747 HE1 PHE X 379 -13.754 -1.143 0.292 0.00 0.00 H

ATOM 5748 CZ PHE X 379 -13.610 -0.488 -1.692 0.00 0.00 C

ATOM 5749 HZ PHE X 379 -13.744 -1.443 -2.167 0.00 0.00 H

ATOM 5750 CD2 PHE X 379 -12.903 1.829 -2.081 0.00 0.00 C

ATOM 5751 HD2 PHE X 379 -12.709 2.594 -2.817 0.00 0.00 H

ATOM 5752 CE2 PHE X 379 -13.172 0.544 -2.630 0.00 0.00 C

ATOM 5753 HE2 PHE X 379 -13.341 0.466 -3.692 0.00 0.00 H

ATOM 5754 C PHE X 379 -13.299 5.097 1.469 0.00 0.00 C

ATOM 5755 O PHE X 379 -13.020 4.826 2.617 0.00 0.00 O

ATOM 5756 N ILE X 380 -13.309 6.335 1.023 0.00 0.00 N

ATOM 5757 HN ILE X 380 -13.534 6.561 0.076 0.00 0.00 H

ATOM 5758 CA ILE X 380 -13.048 7.460 1.854 0.00 0.00 C

ATOM 5759 HA ILE X 380 -12.226 7.100 2.452 0.00 0.00 H

ATOM 5760 CB ILE X 380 -12.604 8.675 0.975 0.00 0.00 C

ATOM 5761 HB ILE X 380 -13.306 8.740 0.118 0.00 0.00 H

ATOM 5762 CG2 ILE X 380 -12.704 9.903 1.840 0.00 0.00 C

ATOM 5763 HG21 ILE X 380 -13.764 10.227 1.945 0.00 0.00 H

ATOM 5764 HG22 ILE X 380 -12.124 10.749 1.404 0.00 0.00 H

ATOM 5765 HG23 ILE X 380 -12.294 9.824 2.878 0.00 0.00 H

ATOM 5766 CG1 ILE X 380 -11.201 8.366 0.463 0.00 0.00 C

ATOM 5767 HG11 ILE X 380 -11.189 7.308 0.111 0.00 0.00 H

ATOM 5768 HG12 ILE X 380 -10.475 8.575 1.264 0.00 0.00 H

ATOM 5769 CD ILE X 380 -10.827 9.233 -0.753 0.00 0.00 C

ATOM 5770 HD1 ILE X 380 -11.578 9.151 -1.566 0.00 0.00 H

ATOM 5771 HD2 ILE X 380 -9.874 8.768 -1.092 0.00 0.00 H

ATOM 5772 HD3 ILE X 380 -10.738 10.289 -0.461 0.00 0.00 H

ATOM 5773 C ILE X 380 -14.128 7.754 2.795 0.00 0.00 C

ATOM 5774 O ILE X 380 -13.760 8.141 3.899 0.00 0.00 O

ATOM 5775 N LEU X 381 -15.462 7.624 2.517 0.00 0.00 N

ATOM 5776 HN LEU X 381 -15.870 7.636 1.605 0.00 0.00 H

ATOM 5777 CA LEU X 381 -16.526 7.672 3.527 0.00 0.00 C

ATOM 5778 HA LEU X 381 -16.558 8.618 4.045 0.00 0.00 H

ATOM 5779 CB LEU X 381 -17.865 7.569 2.692 0.00 0.00 C

ATOM 5780 HB1 LEU X 381 -18.749 7.580 3.351 0.00 0.00 H

ATOM 5781 HB2 LEU X 381 -17.774 6.610 2.141 0.00 0.00 H

ATOM 5782 CG LEU X 381 -18.162 8.730 1.675 0.00 0.00 C

ATOM 5783 HG LEU X 381 -17.484 8.542 0.814 0.00 0.00 H

ATOM 5784 CD1 LEU X 381 -19.542 8.538 1.084 0.00 0.00 C

ATOM 5785 HD11 LEU X 381 -20.334 8.893 1.780 0.00 0.00 H

ATOM 5786 HD12 LEU X 381 -19.770 7.489 0.816 0.00 0.00 H

ATOM 5787 HD13 LEU X 381 -19.679 9.049 0.107 0.00 0.00 H

ATOM 5788 CD2 LEU X 381 -17.980 10.233 2.210 0.00 0.00 C

ATOM 5789 HD21 LEU X 381 -18.180 10.846 1.311 0.00 0.00 H

ATOM 5790 HD22 LEU X 381 -16.924 10.412 2.515 0.00 0.00 H

ATOM 5791 HD23 LEU X 381 -18.729 10.518 2.977 0.00 0.00 H

ATOM 5792 C LEU X 381 -16.459 6.564 4.556 0.00 0.00 C

ATOM 5793 O LEU X 381 -16.565 6.764 5.801 0.00 0.00 O

ATOM 5794 N ILE X 382 -16.199 5.309 4.113 0.00 0.00 N

ATOM 5795 HN ILE X 382 -16.340 5.128 3.142 0.00 0.00 H

ATOM 5796 CA ILE X 382 -15.743 4.217 4.951 0.00 0.00 C

ATOM 5797 HA ILE X 382 -16.502 3.930 5.663 0.00 0.00 H

ATOM 5798 CB ILE X 382 -15.415 2.916 4.115 0.00 0.00 C

ATOM 5799 HB ILE X 382 -14.704 3.142 3.291 0.00 0.00 H

ATOM 5800 CG2 ILE X 382 -14.645 1.805 5.140 0.00 0.00 C

ATOM 5801 HG21 ILE X 382 -15.183 1.847 6.107 0.00 0.00 H

ATOM 5802 HG22 ILE X 382 -13.599 2.184 5.199 0.00 0.00 H

ATOM 5803 HG23 ILE X 382 -14.691 0.857 4.553 0.00 0.00 H

ATOM 5804 CG1 ILE X 382 -16.628 2.428 3.299 0.00 0.00 C

ATOM 5805 HG11 ILE X 382 -17.201 3.281 2.868 0.00 0.00 H

ATOM 5806 HG12 ILE X 382 -17.199 1.876 4.070 0.00 0.00 H

ATOM 5807 CD ILE X 382 -16.233 1.448 2.188 0.00 0.00 C

ATOM 5808 HD1 ILE X 382 -17.114 0.811 1.922 0.00 0.00 H

ATOM 5809 HD2 ILE X 382 -15.455 0.690 2.404 0.00 0.00 H

ATOM 5810 HD3 ILE X 382 -15.867 1.823 1.215 0.00 0.00 H

ATOM 5811 C ILE X 382 -14.616 4.491 5.953 0.00 0.00 C

ATOM 5812 O ILE X 382 -14.815 4.384 7.178 0.00 0.00 O

ATOM 5813 N ALA X 383 -13.505 4.898 5.462 0.00 0.00 N

ATOM 5814 HN ALA X 383 -13.483 5.154 4.500 0.00 0.00 H

ATOM 5815 CA ALA X 383 -12.234 5.219 6.195 0.00 0.00 C

ATOM 5816 HA ALA X 383 -11.572 5.411 5.357 0.00 0.00 H

ATOM 5817 CB ALA X 383 -12.499 6.601 6.880 0.00 0.00 C

ATOM 5818 HB1 ALA X 383 -13.039 6.485 7.847 0.00 0.00 H

ATOM 5819 HB2 ALA X 383 -12.998 7.411 6.308 0.00 0.00 H

ATOM 5820 HB3 ALA X 383 -11.582 7.106 7.239 0.00 0.00 H

ATOM 5821 C ALA X 383 -11.681 4.303 7.052 0.00 0.00 C

ATOM 5822 O ALA X 383 -12.021 3.169 7.140 0.00 0.00 O

ATOM 5823 N GLU X 384 -10.625 4.704 7.826 0.00 0.00 N

ATOM 5824 HN GLU X 384 -10.334 5.655 7.784 0.00 0.00 H

ATOM 5825 CA GLU X 384 -9.716 3.894 8.604 0.00 0.00 C

ATOM 5826 HA GLU X 384 -9.098 4.698 8.981 0.00 0.00 H

ATOM 5827 CB GLU X 384 -10.243 3.248 9.880 0.00 0.00 C

ATOM 5828 HB1 GLU X 384 -11.184 2.691 9.693 0.00 0.00 H

ATOM 5829 HB2 GLU X 384 -10.525 4.064 10.571 0.00 0.00 H

ATOM 5830 CG GLU X 384 -9.238 2.306 10.556 0.00 0.00 C

ATOM 5831 HG1 GLU X 384 -8.879 1.416 9.999 0.00 0.00 H

ATOM 5832 HG2 GLU X 384 -9.722 1.932 11.486 0.00 0.00 H

ATOM 5833 CD GLU X 384 -7.963 2.875 11.050 0.00 0.00 C

ATOM 5834 OE1 GLU X 384 -7.920 4.066 11.400 0.00 0.00 O

ATOM 5835 OE2 GLU X 384 -6.919 2.124 10.993 0.00 0.00 O1-

ATOM 5836 C GLU X 384 -8.792 3.033 7.814 0.00 0.00 C

ATOM 5837 O GLU X 384 -9.402 2.213 7.107 0.00 0.00 O

ATOM 5838 N LEU X 385 -7.426 3.065 7.840 0.00 0.00 N

ATOM 5839 HN LEU X 385 -6.986 3.882 8.207 0.00 0.00 H

ATOM 5840 CA LEU X 385 -6.474 2.220 7.204 0.00 0.00 C

ATOM 5841 HA LEU X 385 -6.620 2.215 6.130 0.00 0.00 H

ATOM 5842 CB LEU X 385 -5.077 2.761 7.613 0.00 0.00 C

ATOM 5843 HB1 LEU X 385 -4.184 2.105 7.572 0.00 0.00 H

ATOM 5844 HB2 LEU X 385 -5.242 3.029 8.676 0.00 0.00 H

ATOM 5845 CG LEU X 385 -4.666 4.041 6.927 0.00 0.00 C

ATOM 5846 HG LEU X 385 -5.384 4.875 7.056 0.00 0.00 H

ATOM 5847 CD1 LEU X 385 -3.333 4.579 7.612 0.00 0.00 C

ATOM 5848 HD11 LEU X 385 -2.656 3.707 7.754 0.00 0.00 H

ATOM 5849 HD12 LEU X 385 -3.558 5.014 8.607 0.00 0.00 H

ATOM 5850 HD13 LEU X 385 -2.952 5.352 6.906 0.00 0.00 H

ATOM 5851 CD2 LEU X 385 -4.549 3.747 5.377 0.00 0.00 C

ATOM 5852 HD21 LEU X 385 -3.891 2.889 5.102 0.00 0.00 H

ATOM 5853 HD22 LEU X 385 -3.912 4.561 4.959 0.00 0.00 H

ATOM 5854 HD23 LEU X 385 -5.584 3.597 5.002 0.00 0.00 H

ATOM 5855 C LEU X 385 -6.581 0.765 7.611 0.00 0.00 C

ATOM 5856 O LEU X 385 -6.374 -0.060 6.691 0.00 0.00 O

ATOM 5857 N ASN X 386 -6.773 0.412 8.864 0.00 0.00 N

ATOM 5858 HN ASN X 386 -6.784 1.131 9.561 0.00 0.00 H

ATOM 5859 CA ASN X 386 -7.199 -0.918 9.178 0.00 0.00 C

ATOM 5860 HA ASN X 386 -6.364 -1.537 8.897 0.00 0.00 H

ATOM 5861 CB ASN X 386 -7.347 -1.156 10.781 0.00 0.00 C

ATOM 5862 HB1 ASN X 386 -7.696 -2.207 10.903 0.00 0.00 H

ATOM 5863 HB2 ASN X 386 -8.105 -0.447 11.165 0.00 0.00 H

ATOM 5864 CG ASN X 386 -6.040 -1.051 11.531 0.00 0.00 C

ATOM 5865 OD1 ASN X 386 -5.042 -0.730 10.883 0.00 0.00 O

ATOM 5866 ND2 ASN X 386 -5.973 -1.417 12.843 0.00 0.00 N

ATOM 5867 HD21 ASN X 386 -6.801 -1.336 13.393 0.00 0.00 H

ATOM 5868 HD22 ASN X 386 -5.026 -1.454 13.171 0.00 0.00 H

ATOM 5869 C ASN X 386 -8.418 -1.520 8.520 0.00 0.00 C

ATOM 5870 O ASN X 386 -8.542 -2.757 8.226 0.00 0.00 O

ATOM 5871 N VAL X 387 -9.449 -0.696 8.172 0.00 0.00 N

ATOM 5872 HN VAL X 387 -9.464 0.265 8.449 0.00 0.00 H

ATOM 5873 CA VAL X 387 -10.736 -1.145 7.718 0.00 0.00 C

ATOM 5874 HA VAL X 387 -10.948 -2.165 8.015 0.00 0.00 H

ATOM 5875 CB VAL X 387 -11.841 -0.214 7.969 0.00 0.00 C

ATOM 5876 HB VAL X 387 -11.636 0.763 7.483 0.00 0.00 H

ATOM 5877 CG1 VAL X 387 -13.234 -0.789 7.472 0.00 0.00 C

ATOM 5878 HG11 VAL X 387 -13.269 -0.918 6.368 0.00 0.00 H

ATOM 5879 HG12 VAL X 387 -14.124 -0.154 7.696 0.00 0.00 H

ATOM 5880 HG13 VAL X 387 -13.350 -1.832 7.841 0.00 0.00 H

ATOM 5881 CG2 VAL X 387 -12.217 -0.010 9.417 0.00 0.00 C

ATOM 5882 HG21 VAL X 387 -11.420 0.374 10.093 0.00 0.00 H

ATOM 5883 HG22 VAL X 387 -12.413 -1.012 9.845 0.00 0.00 H

ATOM 5884 HG23 VAL X 387 -13.096 0.649 9.580 0.00 0.00 H

ATOM 5885 C VAL X 387 -10.692 -1.295 6.170 0.00 0.00 C

ATOM 5886 O VAL X 387 -11.051 -2.343 5.666 0.00 0.00 O

ATOM 5887 N ILE X 388 -9.974 -0.410 5.434 0.00 0.00 N

ATOM 5888 HN ILE X 388 -9.670 0.463 5.812 0.00 0.00 H

ATOM 5889 CA ILE X 388 -10.060 -0.574 3.974 0.00 0.00 C

ATOM 5890 HA ILE X 388 -10.997 -0.939 3.583 0.00 0.00 H

ATOM 5891 CB ILE X 388 -9.713 0.831 3.364 0.00 0.00 C

ATOM 5892 HB ILE X 388 -9.543 0.555 2.302 0.00 0.00 H

ATOM 5893 CG2 ILE X 388 -10.987 1.708 3.429 0.00 0.00 C

ATOM 5894 HG21 ILE X 388 -11.238 1.906 4.487 0.00 0.00 H

ATOM 5895 HG22 ILE X 388 -11.857 1.176 2.996 0.00 0.00 H

ATOM 5896 HG23 ILE X 388 -10.931 2.598 2.771 0.00 0.00 H

ATOM 5897 CG1 ILE X 388 -8.413 1.530 3.802 0.00 0.00 C

ATOM 5898 HG11 ILE X 388 -7.556 0.828 3.871 0.00 0.00 H

ATOM 5899 HG12 ILE X 388 -8.574 2.053 4.768 0.00 0.00 H

ATOM 5900 CD ILE X 388 -8.034 2.549 2.788 0.00 0.00 C

ATOM 5901 HD1 ILE X 388 -8.770 3.341 2.534 0.00 0.00 H

ATOM 5902 HD2 ILE X 388 -7.669 2.138 1.826 0.00 0.00 H

ATOM 5903 HD3 ILE X 388 -7.157 3.113 3.158 0.00 0.00 H

ATOM 5904 C ILE X 388 -9.063 -1.645 3.498 0.00 0.00 C

ATOM 5905 O ILE X 388 -9.389 -2.339 2.511 0.00 0.00 O

ATOM 5906 N ALA X 389 -8.011 -1.915 4.256 0.00 0.00 N

ATOM 5907 HN ALA X 389 -7.888 -1.313 5.036 0.00 0.00 H

ATOM 5908 CA ALA X 389 -6.921 -2.715 3.803 0.00 0.00 C

ATOM 5909 HA ALA X 389 -6.630 -2.406 2.806 0.00 0.00 H

ATOM 5910 CB ALA X 389 -5.572 -2.545 4.554 0.00 0.00 C

ATOM 5911 HB1 ALA X 389 -5.674 -2.904 5.595 0.00 0.00 H

ATOM 5912 HB2 ALA X 389 -5.220 -1.502 4.452 0.00 0.00 H

ATOM 5913 HB3 ALA X 389 -4.819 -3.180 4.033 0.00 0.00 H

ATOM 5914 C ALA X 389 -7.182 -4.234 3.533 0.00 0.00 C

ATOM 5915 O ALA X 389 -6.866 -4.649 2.425 0.00 0.00 O

ATOM 5916 N PRO X 390 -7.958 -5.055 4.309 0.00 0.00 N

ATOM 5917 CD PRO X 390 -8.101 -4.833 5.720 0.00 0.00 C

ATOM 5918 HD1 PRO X 390 -9.123 -4.423 5.823 0.00 0.00 H

ATOM 5919 HD2 PRO X 390 -7.399 -4.068 6.109 0.00 0.00 H

ATOM 5920 CA PRO X 390 -8.105 -6.453 4.013 0.00 0.00 C

ATOM 5921 HA PRO X 390 -7.183 -6.931 3.722 0.00 0.00 H

ATOM 5922 CB PRO X 390 -8.668 -7.069 5.333 0.00 0.00 C

ATOM 5923 HB1 PRO X 390 -8.333 -8.128 5.420 0.00 0.00 H

ATOM 5924 HB2 PRO X 390 -9.764 -6.997 5.451 0.00 0.00 H

ATOM 5925 CG PRO X 390 -7.977 -6.294 6.365 0.00 0.00 C

ATOM 5926 HG1 PRO X 390 -8.422 -6.232 7.381 0.00 0.00 H

ATOM 5927 HG2 PRO X 390 -6.884 -6.456 6.485 0.00 0.00 H

ATOM 5928 C PRO X 390 -9.029 -6.634 2.795 0.00 0.00 C

ATOM 5929 O PRO X 390 -8.965 -7.588 2.069 0.00 0.00 O

ATOM 5930 N ILE X 391 -9.873 -5.581 2.582 0.00 0.00 N

ATOM 5931 HN ILE X 391 -10.005 -4.840 3.235 0.00 0.00 H

ATOM 5932 CA ILE X 391 -10.866 -5.727 1.516 0.00 0.00 C

ATOM 5933 HA ILE X 391 -11.398 -6.642 1.750 0.00 0.00 H

ATOM 5934 CB ILE X 391 -11.899 -4.508 1.746 0.00 0.00 C

ATOM 5935 HB ILE X 391 -11.438 -3.494 1.779 0.00 0.00 H

ATOM 5936 CG2 ILE X 391 -12.865 -4.541 0.574 0.00 0.00 C

ATOM 5937 HG21 ILE X 391 -12.406 -4.410 -0.423 0.00 0.00 H

ATOM 5938 HG22 ILE X 391 -13.623 -3.735 0.700 0.00 0.00 H

ATOM 5939 HG23 ILE X 391 -13.440 -5.490 0.514 0.00 0.00 H

ATOM 5940 CG1 ILE X 391 -12.695 -4.508 3.040 0.00 0.00 C

ATOM 5941 HG11 ILE X 391 -12.064 -4.651 3.945 0.00 0.00 H

ATOM 5942 HG12 ILE X 391 -13.401 -5.351 3.150 0.00 0.00 H

ATOM 5943 CD ILE X 391 -13.506 -3.260 3.282 0.00 0.00 C

ATOM 5944 HD1 ILE X 391 -14.001 -2.819 2.392 0.00 0.00 H

ATOM 5945 HD2 ILE X 391 -12.849 -2.530 3.819 0.00 0.00 H

ATOM 5946 HD3 ILE X 391 -14.339 -3.540 3.966 0.00 0.00 H

ATOM 5947 C ILE X 391 -10.210 -5.704 0.094 0.00 0.00 C

ATOM 5948 O ILE X 391 -10.613 -6.415 -0.820 0.00 0.00 O

ATOM 5949 N ILE X 392 -9.089 -4.963 -0.125 0.00 0.00 N

ATOM 5950 HN ILE X 392 -8.722 -4.376 0.592 0.00 0.00 H

ATOM 5951 CA ILE X 392 -8.321 -4.854 -1.369 0.00 0.00 C

ATOM 5952 HA ILE X 392 -8.970 -5.214 -2.152 0.00 0.00 H

ATOM 5953 CB ILE X 392 -7.931 -3.380 -1.697 0.00 0.00 C

ATOM 5954 HB ILE X 392 -8.879 -2.801 -1.688 0.00 0.00 H

ATOM 5955 CG2 ILE X 392 -7.120 -2.827 -0.566 0.00 0.00 C

ATOM 5956 HG21 ILE X 392 -6.690 -1.817 -0.768 0.00 0.00 H

ATOM 5957 HG22 ILE X 392 -6.334 -3.554 -0.261 0.00 0.00 H

ATOM 5958 HG23 ILE X 392 -7.693 -2.714 0.382 0.00 0.00 H

ATOM 5959 CG1 ILE X 392 -7.277 -3.042 -3.009 0.00 0.00 C

ATOM 5960 HG11 ILE X 392 -6.334 -3.633 -2.998 0.00 0.00 H

ATOM 5961 HG12 ILE X 392 -7.024 -1.962 -3.073 0.00 0.00 H

ATOM 5962 CD ILE X 392 -7.958 -3.541 -4.290 0.00 0.00 C

ATOM 5963 HD1 ILE X 392 -8.055 -4.653 -4.247 0.00 0.00 H

ATOM 5964 HD2 ILE X 392 -7.265 -3.341 -5.135 0.00 0.00 H

ATOM 5965 HD3 ILE X 392 -8.976 -3.206 -4.565 0.00 0.00 H

ATOM 5966 C ILE X 392 -7.139 -5.864 -1.425 0.00 0.00 C

ATOM 5967 O ILE X 392 -6.600 -6.133 -2.468 0.00 0.00 O

ATOM 5968 N SER X 393 -6.851 -6.469 -0.285 0.00 0.00 N

ATOM 5969 HN SER X 393 -7.284 -6.122 0.542 0.00 0.00 H

ATOM 5970 CA SER X 393 -5.854 -7.435 -0.183 0.00 0.00 C

ATOM 5971 HA SER X 393 -4.894 -7.190 -0.588 0.00 0.00 H

ATOM 5972 CB SER X 393 -5.625 -7.841 1.281 0.00 0.00 C

ATOM 5973 HB1 SER X 393 -6.580 -8.017 1.813 0.00 0.00 H

ATOM 5974 HB2 SER X 393 -5.118 -6.995 1.794 0.00 0.00 H

ATOM 5975 OG SER X 393 -4.844 -9.085 1.468 0.00 0.00 O

ATOM 5976 HG1 SER X 393 -5.378 -9.756 1.031 0.00 0.00 H

ATOM 5977 C SER X 393 -6.349 -8.803 -0.852 0.00 0.00 C

ATOM 5978 O SER X 393 -5.651 -9.382 -1.665 0.00 0.00 O

ATOM 5979 N ASN X 394 -7.606 -9.179 -0.661 0.00 0.00 N

ATOM 5980 HN ASN X 394 -8.053 -8.696 0.084 0.00 0.00 H

ATOM 5981 CA ASN X 394 -8.206 -10.327 -1.246 0.00 0.00 C

ATOM 5982 HA ASN X 394 -7.639 -11.208 -0.994 0.00 0.00 H

ATOM 5983 CB ASN X 394 -9.604 -10.586 -0.665 0.00 0.00 C

ATOM 5984 HB1 ASN X 394 -10.046 -11.351 -1.348 0.00 0.00 H

ATOM 5985 HB2 ASN X 394 -10.249 -9.680 -0.715 0.00 0.00 H

ATOM 5986 CG ASN X 394 -9.402 -11.141 0.725 0.00 0.00 C

ATOM 5987 OD1 ASN X 394 -8.332 -11.185 1.278 0.00 0.00 O

ATOM 5988 ND2 ASN X 394 -10.522 -11.696 1.234 0.00 0.00 N

ATOM 5989 HD21 ASN X 394 -10.424 -12.309 2.011 0.00 0.00 H

ATOM 5990 HD22 ASN X 394 -11.359 -11.605 0.683 0.00 0.00 H

ATOM 5991 C ASN X 394 -8.268 -10.144 -2.767 0.00 0.00 C

ATOM 5992 O ASN X 394 -8.046 -11.081 -3.517 0.00 0.00 O

ATOM 5993 N PHE X 395 -8.503 -8.913 -3.227 0.00 0.00 N

ATOM 5994 HN PHE X 395 -8.840 -8.164 -2.668 0.00 0.00 H

ATOM 5995 CA PHE X 395 -8.441 -8.584 -4.643 0.00 0.00 C

ATOM 5996 HA PHE X 395 -8.958 -9.445 -5.046 0.00 0.00 H

ATOM 5997 CB PHE X 395 -9.166 -7.332 -5.037 0.00 0.00 C

ATOM 5998 HB1 PHE X 395 -8.848 -6.853 -5.992 0.00 0.00 H

ATOM 5999 HB2 PHE X 395 -9.094 -6.567 -4.233 0.00 0.00 H

ATOM 6000 CG PHE X 395 -10.588 -7.595 -5.155 0.00 0.00 C

ATOM 6001 CD1 PHE X 395 -11.380 -7.588 -4.002 0.00 0.00 C

ATOM 6002 HD1 PHE X 395 -10.929 -7.383 -3.039 0.00 0.00 H

ATOM 6003 CE1 PHE X 395 -12.781 -7.529 -4.078 0.00 0.00 C

ATOM 6004 HE1 PHE X 395 -13.444 -7.417 -3.230 0.00 0.00 H

ATOM 6005 CZ PHE X 395 -13.408 -7.751 -5.361 0.00 0.00 C

ATOM 6006 HZ PHE X 395 -14.486 -7.761 -5.398 0.00 0.00 H

ATOM 6007 CD2 PHE X 395 -11.267 -7.812 -6.414 0.00 0.00 C

ATOM 6008 HD2 PHE X 395 -10.601 -8.004 -7.246 0.00 0.00 H

ATOM 6009 CE2 PHE X 395 -12.671 -7.834 -6.536 0.00 0.00 C

ATOM 6010 HE2 PHE X 395 -13.091 -7.961 -7.521 0.00 0.00 H

ATOM 6011 C PHE X 395 -7.101 -8.643 -5.320 0.00 0.00 C

ATOM 6012 O PHE X 395 -6.982 -9.014 -6.462 0.00 0.00 O

ATOM 6013 N PHE X 396 -6.025 -8.176 -4.598 0.00 0.00 N

ATOM 6014 HN PHE X 396 -6.112 -7.849 -3.661 0.00 0.00 H

ATOM 6015 CA PHE X 396 -4.650 -8.428 -5.087 0.00 0.00 C

ATOM 6016 HA PHE X 396 -4.581 -8.024 -6.084 0.00 0.00 H

ATOM 6017 CB PHE X 396 -3.633 -7.792 -4.090 0.00 0.00 C

ATOM 6018 HB1 PHE X 396 -2.572 -8.121 -3.973 0.00 0.00 H

ATOM 6019 HB2 PHE X 396 -4.043 -7.910 -3.059 0.00 0.00 H

ATOM 6020 CG PHE X 396 -3.446 -6.341 -4.388 0.00 0.00 C

ATOM 6021 CD1 PHE X 396 -3.154 -5.798 -5.622 0.00 0.00 C

ATOM 6022 HD1 PHE X 396 -3.085 -6.386 -6.533 0.00 0.00 H

ATOM 6023 CE1 PHE X 396 -2.848 -4.446 -5.810 0.00 0.00 C

ATOM 6024 HE1 PHE X 396 -2.570 -4.119 -6.799 0.00 0.00 H

ATOM 6025 CZ PHE X 396 -2.912 -3.586 -4.634 0.00 0.00 C

ATOM 6026 HZ PHE X 396 -2.719 -2.535 -4.783 0.00 0.00 H

ATOM 6027 CD2 PHE X 396 -3.490 -5.484 -3.321 0.00 0.00 C

ATOM 6028 HD2 PHE X 396 -3.699 -5.963 -2.374 0.00 0.00 H

ATOM 6029 CE2 PHE X 396 -3.296 -4.078 -3.453 0.00 0.00 C

ATOM 6030 HE2 PHE X 396 -3.527 -3.383 -2.658 0.00 0.00 H

ATOM 6031 C PHE X 396 -4.263 -9.901 -5.007 0.00 0.00 C

ATOM 6032 O PHE X 396 -3.594 -10.388 -5.970 0.00 0.00 O

ATOM 6033 N LEU X 397 -4.799 -10.580 -4.012 0.00 0.00 N

ATOM 6034 HN LEU X 397 -5.244 -10.231 -3.197 0.00 0.00 H

ATOM 6035 CA LEU X 397 -4.611 -12.039 -4.006 0.00 0.00 C

ATOM 6036 HA LEU X 397 -3.550 -12.215 -4.181 0.00 0.00 H

ATOM 6037 CB LEU X 397 -5.116 -12.600 -2.653 0.00 0.00 C

ATOM 6038 HB1 LEU X 397 -5.252 -13.695 -2.746 0.00 0.00 H

ATOM 6039 HB2 LEU X 397 -6.084 -12.101 -2.429 0.00 0.00 H

ATOM 6040 CG LEU X 397 -4.114 -12.366 -1.520 0.00 0.00 C

ATOM 6041 HG LEU X 397 -3.665 -11.367 -1.709 0.00 0.00 H

ATOM 6042 CD1 LEU X 397 -4.802 -12.422 -0.191 0.00 0.00 C

ATOM 6043 HD11 LEU X 397 -5.611 -11.665 -0.087 0.00 0.00 H

ATOM 6044 HD12 LEU X 397 -4.103 -12.321 0.665 0.00 0.00 H

ATOM 6045 HD13 LEU X 397 -5.256 -13.434 -0.153 0.00 0.00 H

ATOM 6046 CD2 LEU X 397 -2.898 -13.267 -1.560 0.00 0.00 C

ATOM 6047 HD21 LEU X 397 -2.140 -13.122 -0.761 0.00 0.00 H

ATOM 6048 HD22 LEU X 397 -2.395 -13.078 -2.526 0.00 0.00 H

ATOM 6049 HD23 LEU X 397 -3.283 -14.308 -1.543 0.00 0.00 H

ATOM 6050 C LEU X 397 -5.354 -12.667 -5.215 0.00 0.00 C

ATOM 6051 O LEU X 397 -4.688 -13.341 -6.035 0.00 0.00 O

ATOM 6052 N ALA X 398 -6.673 -12.459 -5.483 0.00 0.00 N

ATOM 6053 HN ALA X 398 -7.174 -11.787 -4.947 0.00 0.00 H

ATOM 6054 CA ALA X 398 -7.361 -12.867 -6.681 0.00 0.00 C

ATOM 6055 HA ALA X 398 -7.508 -13.929 -6.566 0.00 0.00 H

ATOM 6056 CB ALA X 398 -8.716 -12.104 -6.788 0.00 0.00 C

ATOM 6057 HB1 ALA X 398 -9.198 -12.226 -7.791 0.00 0.00 H

ATOM 6058 HB2 ALA X 398 -8.659 -11.000 -6.694 0.00 0.00 H

ATOM 6059 HB3 ALA X 398 -9.419 -12.427 -5.997 0.00 0.00 H

ATOM 6060 C ALA X 398 -6.555 -12.507 -7.957 0.00 0.00 C

ATOM 6061 O ALA X 398 -6.324 -13.357 -8.823 0.00 0.00 O

ATOM 6062 N SER X 399 -6.021 -11.314 -8.119 0.00 0.00 N

ATOM 6063 HN SER X 399 -6.315 -10.579 -7.515 0.00 0.00 H

ATOM 6064 CA SER X 399 -5.230 -10.915 -9.219 0.00 0.00 C

ATOM 6065 HA SER X 399 -5.779 -10.998 -10.140 0.00 0.00 H

ATOM 6066 CB SER X 399 -4.876 -9.408 -9.129 0.00 0.00 C

ATOM 6067 HB1 SER X 399 -4.337 -9.204 -10.072 0.00 0.00 H

ATOM 6068 HB2 SER X 399 -4.140 -9.251 -8.313 0.00 0.00 H

ATOM 6069 OG SER X 399 -5.954 -8.514 -9.104 0.00 0.00 O

ATOM 6070 HG1 SER X 399 -6.535 -8.566 -8.348 0.00 0.00 H

ATOM 6071 C SER X 399 -3.888 -11.672 -9.526 0.00 0.00 C

ATOM 6072 O SER X 399 -3.597 -11.975 -10.664 0.00 0.00 O

ATOM 6073 N TYR X 400 -3.182 -12.062 -8.496 0.00 0.00 N

ATOM 6074 HN TYR X 400 -3.278 -11.536 -7.654 0.00 0.00 H

ATOM 6075 CA TYR X 400 -1.885 -12.614 -8.771 0.00 0.00 C

ATOM 6076 HA TYR X 400 -1.437 -12.451 -9.741 0.00 0.00 H

ATOM 6077 CB TYR X 400 -0.981 -12.294 -7.585 0.00 0.00 C

ATOM 6078 HB1 TYR X 400 -0.015 -12.837 -7.685 0.00 0.00 H

ATOM 6079 HB2 TYR X 400 -1.392 -12.601 -6.601 0.00 0.00 H

ATOM 6080 CG TYR X 400 -0.665 -10.812 -7.588 0.00 0.00 C

ATOM 6081 CD1 TYR X 400 -0.525 -10.058 -6.403 0.00 0.00 C

ATOM 6082 HD1 TYR X 400 -0.640 -10.585 -5.464 0.00 0.00 H

ATOM 6083 CE1 TYR X 400 -0.175 -8.699 -6.405 0.00 0.00 C

ATOM 6084 HE1 TYR X 400 -0.022 -8.211 -5.453 0.00 0.00 H

ATOM 6085 CZ TYR X 400 0.039 -8.029 -7.575 0.00 0.00 C

ATOM 6086 OH TYR X 400 0.665 -6.765 -7.662 0.00 0.00 O

ATOM 6087 HH TYR X 400 0.766 -6.463 -6.763 0.00 0.00 H

ATOM 6088 CD2 TYR X 400 -0.436 -10.089 -8.791 0.00 0.00 C

ATOM 6089 HD2 TYR X 400 -0.619 -10.550 -9.748 0.00 0.00 H

ATOM 6090 CE2 TYR X 400 -0.095 -8.730 -8.834 0.00 0.00 C

ATOM 6091 HE2 TYR X 400 0.034 -8.257 -9.799 0.00 0.00 H

ATOM 6092 C TYR X 400 -2.166 -14.106 -9.015 0.00 0.00 C

ATOM 6093 O TYR X 400 -1.408 -14.904 -9.596 0.00 0.00 O

ATOM 6094 N ALA X 401 -3.404 -14.533 -8.578 0.00 0.00 N

ATOM 6095 HN ALA X 401 -3.918 -13.838 -8.075 0.00 0.00 H

ATOM 6096 CA ALA X 401 -3.936 -15.888 -8.808 0.00 0.00 C

ATOM 6097 HA ALA X 401 -3.032 -16.478 -8.771 0.00 0.00 H

ATOM 6098 CB ALA X 401 -5.022 -16.450 -7.811 0.00 0.00 C

ATOM 6099 HB1 ALA X 401 -4.758 -16.353 -6.730 0.00 0.00 H

ATOM 6100 HB2 ALA X 401 -5.373 -17.435 -8.166 0.00 0.00 H

ATOM 6101 HB3 ALA X 401 -5.860 -15.723 -7.880 0.00 0.00 H

ATOM 6102 C ALA X 401 -4.260 -16.024 -10.236 0.00 0.00 C

ATOM 6103 O ALA X 401 -3.955 -17.018 -10.923 0.00 0.00 O

ATOM 6104 N LEU X 402 -4.988 -15.013 -10.866 0.00 0.00 N

ATOM 6105 HN LEU X 402 -5.238 -14.190 -10.363 0.00 0.00 H

ATOM 6106 CA LEU X 402 -5.447 -14.954 -12.241 0.00 0.00 C

ATOM 6107 HA LEU X 402 -6.004 -15.842 -12.474 0.00 0.00 H

ATOM 6108 CB LEU X 402 -6.299 -13.706 -12.669 0.00 0.00 C

ATOM 6109 HB1 LEU X 402 -6.472 -13.922 -13.742 0.00 0.00 H

ATOM 6110 HB2 LEU X 402 -5.784 -12.738 -12.445 0.00 0.00 H

ATOM 6111 CG LEU X 402 -7.760 -13.549 -12.102 0.00 0.00 C

ATOM 6112 HG LEU X 402 -7.747 -13.387 -11.003 0.00 0.00 H

ATOM 6113 CD1 LEU X 402 -8.432 -12.343 -12.649 0.00 0.00 C

ATOM 6114 HD11 LEU X 402 -9.419 -12.125 -12.176 0.00 0.00 H

ATOM 6115 HD12 LEU X 402 -8.523 -12.353 -13.757 0.00 0.00 H

ATOM 6116 HD13 LEU X 402 -7.835 -11.471 -12.289 0.00 0.00 H

ATOM 6117 CD2 LEU X 402 -8.547 -14.785 -12.429 0.00 0.00 C

ATOM 6118 HD21 LEU X 402 -8.545 -14.915 -13.539 0.00 0.00 H

ATOM 6119 HD22 LEU X 402 -9.556 -14.766 -11.976 0.00 0.00 H

ATOM 6120 HD23 LEU X 402 -8.049 -15.657 -11.944 0.00 0.00 H

ATOM 6121 C LEU X 402 -4.048 -15.036 -13.020 0.00 0.00 C

ATOM 6122 O LEU X 402 -4.013 -15.939 -13.844 0.00 0.00 O

ATOM 6123 N ILE X 403 -3.057 -14.358 -12.634 0.00 0.00 N

ATOM 6124 HN ILE X 403 -3.152 -13.678 -11.904 0.00 0.00 H

ATOM 6125 CA ILE X 403 -1.619 -14.394 -13.181 0.00 0.00 C

ATOM 6126 HA ILE X 403 -1.722 -14.073 -14.201 0.00 0.00 H

ATOM 6127 CB ILE X 403 -0.658 -13.440 -12.585 0.00 0.00 C

ATOM 6128 HB ILE X 403 -0.759 -13.450 -11.476 0.00 0.00 H

ATOM 6129 CG2 ILE X 403 0.811 -13.638 -13.228 0.00 0.00 C

ATOM 6130 HG21 ILE X 403 1.226 -14.607 -12.876 0.00 0.00 H

ATOM 6131 HG22 ILE X 403 1.454 -12.819 -12.859 0.00 0.00 H

ATOM 6132 HG23 ILE X 403 0.683 -13.684 -14.338 0.00 0.00 H

ATOM 6133 CG1 ILE X 403 -0.975 -11.950 -12.799 0.00 0.00 C

ATOM 6134 HG11 ILE X 403 -0.309 -11.313 -12.194 0.00 0.00 H

ATOM 6135 HG12 ILE X 403 -1.987 -11.632 -12.437 0.00 0.00 H

ATOM 6136 CD ILE X 403 -0.850 -11.647 -14.348 0.00 0.00 C

ATOM 6137 HD1 ILE X 403 -1.713 -11.052 -14.714 0.00 0.00 H

ATOM 6138 HD2 ILE X 403 -0.981 -12.579 -14.935 0.00 0.00 H

ATOM 6139 HD3 ILE X 403 0.083 -11.093 -14.575 0.00 0.00 H

ATOM 6140 C ILE X 403 -1.139 -15.813 -13.246 0.00 0.00 C

ATOM 6141 O ILE X 403 -1.001 -16.415 -14.320 0.00 0.00 O

ATOM 6142 N ASN X 404 -0.946 -16.410 -12.026 0.00 0.00 N

ATOM 6143 HN ASN X 404 -1.133 -15.853 -11.216 0.00 0.00 H

ATOM 6144 CA ASN X 404 -0.331 -17.683 -11.773 0.00 0.00 C

ATOM 6145 HA ASN X 404 0.638 -17.659 -12.244 0.00 0.00 H

ATOM 6146 CB ASN X 404 -0.115 -17.876 -10.285 0.00 0.00 C

ATOM 6147 HB1 ASN X 404 0.143 -18.926 -10.016 0.00 0.00 H

ATOM 6148 HB2 ASN X 404 -1.116 -17.728 -9.810 0.00 0.00 H

ATOM 6149 CG ASN X 404 1.009 -17.035 -9.715 0.00 0.00 C

ATOM 6150 OD1 ASN X 404 1.843 -16.607 -10.468 0.00 0.00 O

ATOM 6151 ND2 ASN X 404 0.936 -16.823 -8.365 0.00 0.00 N

ATOM 6152 HD21 ASN X 404 1.454 -16.051 -7.997 0.00 0.00 H

ATOM 6153 HD22 ASN X 404 0.152 -17.179 -7.853 0.00 0.00 H

ATOM 6154 C ASN X 404 -1.133 -18.799 -12.382 0.00 0.00 C

ATOM 6155 O ASN X 404 -0.607 -19.692 -13.051 0.00 0.00 O

ATOM 6156 N PHE X 405 -2.464 -18.861 -12.271 0.00 0.00 N

ATOM 6157 HN PHE X 405 -2.888 -18.059 -11.866 0.00 0.00 H

ATOM 6158 CA PHE X 405 -3.300 -19.789 -13.016 0.00 0.00 C

ATOM 6159 HA PHE X 405 -2.866 -20.728 -12.715 0.00 0.00 H

ATOM 6160 CB PHE X 405 -4.802 -19.794 -12.545 0.00 0.00 C

ATOM 6161 HB1 PHE X 405 -5.141 -18.744 -12.682 0.00 0.00 H

ATOM 6162 HB2 PHE X 405 -4.862 -20.110 -11.482 0.00 0.00 H

ATOM 6163 CG PHE X 405 -5.629 -20.820 -13.343 0.00 0.00 C

ATOM 6164 CD1 PHE X 405 -6.886 -20.454 -13.988 0.00 0.00 C

ATOM 6165 HD1 PHE X 405 -7.187 -19.420 -13.989 0.00 0.00 H

ATOM 6166 CE1 PHE X 405 -7.617 -21.354 -14.775 0.00 0.00 C

ATOM 6167 HE1 PHE X 405 -8.537 -21.004 -15.241 0.00 0.00 H

ATOM 6168 CZ PHE X 405 -7.220 -22.669 -14.825 0.00 0.00 C

ATOM 6169 HZ PHE X 405 -7.760 -23.537 -15.169 0.00 0.00 H

ATOM 6170 CD2 PHE X 405 -5.261 -22.155 -13.476 0.00 0.00 C

ATOM 6171 HD2 PHE X 405 -4.336 -22.477 -13.011 0.00 0.00 H

ATOM 6172 CE2 PHE X 405 -6.050 -23.078 -14.247 0.00 0.00 C

ATOM 6173 HE2 PHE X 405 -5.669 -24.071 -14.422 0.00 0.00 H

ATOM 6174 C PHE X 405 -3.170 -19.695 -14.530 0.00 0.00 C

ATOM 6175 O PHE X 405 -3.123 -20.651 -15.359 0.00 0.00 O

ATOM 6176 N SER X 406 -3.171 -18.427 -15.068 0.00 0.00 N

ATOM 6177 HN SER X 406 -3.376 -17.721 -14.402 0.00 0.00 H

ATOM 6178 CA SER X 406 -3.165 -18.067 -16.503 0.00 0.00 C

ATOM 6179 HA SER X 406 -3.948 -18.632 -16.985 0.00 0.00 H

ATOM 6180 CB SER X 406 -3.400 -16.547 -16.801 0.00 0.00 C

ATOM 6181 HB1 SER X 406 -3.320 -16.329 -17.885 0.00 0.00 H

ATOM 6182 HB2 SER X 406 -2.693 -15.973 -16.157 0.00 0.00 H

ATOM 6183 OG SER X 406 -4.746 -16.162 -16.459 0.00 0.00 O

ATOM 6184 HG1 SER X 406 -4.744 -16.149 -15.502 0.00 0.00 H

ATOM 6185 C SER X 406 -1.879 -18.511 -17.170 0.00 0.00 C

ATOM 6186 O SER X 406 -1.821 -18.935 -18.315 0.00 0.00 O

ATOM 6187 N VAL X 407 -0.734 -18.396 -16.438 0.00 0.00 N

ATOM 6188 HN VAL X 407 -0.731 -17.854 -15.606 0.00 0.00 H

ATOM 6189 CA VAL X 407 0.556 -18.944 -16.825 0.00 0.00 C

ATOM 6190 HA VAL X 407 0.751 -18.653 -17.841 0.00 0.00 H

ATOM 6191 CB VAL X 407 1.726 -18.339 -16.002 0.00 0.00 C

ATOM 6192 HB VAL X 407 1.561 -18.467 -14.912 0.00 0.00 H

ATOM 6193 CG1 VAL X 407 3.074 -18.988 -16.426 0.00 0.00 C

ATOM 6194 HG11 VAL X 407 3.226 -20.033 -16.058 0.00 0.00 H

ATOM 6195 HG12 VAL X 407 3.891 -18.293 -16.131 0.00 0.00 H

ATOM 6196 HG13 VAL X 407 3.119 -19.074 -17.525 0.00 0.00 H

ATOM 6197 CG2 VAL X 407 1.703 -16.842 -16.346 0.00 0.00 C

ATOM 6198 HG21 VAL X 407 0.717 -16.472 -16.004 0.00 0.00 H

ATOM 6199 HG22 VAL X 407 1.739 -16.703 -17.440 0.00 0.00 H

ATOM 6200 HG23 VAL X 407 2.561 -16.294 -15.915 0.00 0.00 H

ATOM 6201 C VAL X 407 0.506 -20.515 -16.827 0.00 0.00 C

ATOM 6202 O VAL X 407 0.883 -21.196 -17.789 0.00 0.00 O

ATOM 6203 N PHE X 408 -0.059 -21.167 -15.847 0.00 0.00 N

ATOM 6204 HN PHE X 408 -0.348 -20.732 -14.990 0.00 0.00 H

ATOM 6205 CA PHE X 408 -0.209 -22.588 -15.860 0.00 0.00 C

ATOM 6206 HA PHE X 408 0.767 -23.034 -16.006 0.00 0.00 H

ATOM 6207 CB PHE X 408 -0.507 -23.102 -14.467 0.00 0.00 C

ATOM 6208 HB1 PHE X 408 -1.397 -22.514 -14.150 0.00 0.00 H

ATOM 6209 HB2 PHE X 408 0.302 -22.848 -13.741 0.00 0.00 H

ATOM 6210 CG PHE X 408 -0.729 -24.605 -14.382 0.00 0.00 C

ATOM 6211 CD1 PHE X 408 0.354 -25.478 -14.720 0.00 0.00 C

ATOM 6212 HD1 PHE X 408 1.273 -25.019 -15.043 0.00 0.00 H

ATOM 6213 CE1 PHE X 408 0.217 -26.861 -14.774 0.00 0.00 C

ATOM 6214 HE1 PHE X 408 1.150 -27.365 -14.961 0.00 0.00 H

ATOM 6215 CZ PHE X 408 -1.037 -27.454 -14.418 0.00 0.00 C

ATOM 6216 HZ PHE X 408 -1.147 -28.528 -14.389 0.00 0.00 H

ATOM 6217 CD2 PHE X 408 -1.899 -25.159 -14.059 0.00 0.00 C

ATOM 6218 HD2 PHE X 408 -2.714 -24.516 -13.788 0.00 0.00 H

ATOM 6219 CE2 PHE X 408 -2.135 -26.589 -14.151 0.00 0.00 C

ATOM 6220 HE2 PHE X 408 -3.169 -26.767 -13.869 0.00 0.00 H

ATOM 6221 C PHE X 408 -1.205 -23.103 -16.953 0.00 0.00 C

ATOM 6222 O PHE X 408 -0.893 -24.147 -17.606 0.00 0.00 O

ATOM 6223 N HSD X 409 -2.403 -22.486 -17.215 0.00 0.00 N

ATOM 6224 HN HSD X 409 -2.638 -21.831 -16.508 0.00 0.00 H

ATOM 6225 CA HSD X 409 -3.324 -22.889 -18.217 0.00 0.00 C

ATOM 6226 HA HSD X 409 -3.474 -23.939 -18.002 0.00 0.00 H

ATOM 6227 CB HSD X 409 -4.645 -22.144 -18.098 0.00 0.00 C

ATOM 6228 HB1 HSD X 409 -4.446 -21.092 -18.357 0.00 0.00 H

ATOM 6229 HB2 HSD X 409 -5.090 -22.227 -17.082 0.00 0.00 H

ATOM 6230 ND1 HSD X 409 -5.754 -22.235 -20.390 0.00 0.00 N

ATOM 6231 HD1 HSD X 409 -5.273 -21.555 -20.937 0.00 0.00 H

ATOM 6232 CG HSD X 409 -5.698 -22.575 -19.011 0.00 0.00 C

ATOM 6233 CE1 HSD X 409 -6.799 -22.874 -20.925 0.00 0.00 C

ATOM 6234 HE1 HSD X 409 -7.092 -22.877 -21.975 0.00 0.00 H

ATOM 6235 NE2 HSD X 409 -7.492 -23.561 -20.018 0.00 0.00 N

ATOM 6236 CD2 HSD X 409 -6.752 -23.379 -18.780 0.00 0.00 C

ATOM 6237 HD2 HSD X 409 -6.941 -23.988 -17.903 0.00 0.00 H

ATOM 6238 C HSD X 409 -2.759 -22.840 -19.620 0.00 0.00 C

ATOM 6239 O HSD X 409 -2.773 -23.782 -20.406 0.00 0.00 O

ATOM 6240 N ALA X 410 -2.002 -21.754 -19.882 0.00 0.00 N

ATOM 6241 HN ALA X 410 -2.013 -20.995 -19.238 0.00 0.00 H

ATOM 6242 CA ALA X 410 -1.344 -21.516 -21.146 0.00 0.00 C

ATOM 6243 HA ALA X 410 -2.025 -21.812 -21.927 0.00 0.00 H

ATOM 6244 CB ALA X 410 -0.885 -20.065 -21.182 0.00 0.00 C

ATOM 6245 HB1 ALA X 410 -0.199 -19.768 -21.994 0.00 0.00 H

ATOM 6246 HB2 ALA X 410 -0.379 -19.684 -20.265 0.00 0.00 H

ATOM 6247 HB3 ALA X 410 -1.783 -19.433 -21.383 0.00 0.00 H

ATOM 6248 C ALA X 410 -0.099 -22.372 -21.357 0.00 0.00 C

ATOM 6249 O ALA X 410 0.239 -22.729 -22.496 0.00 0.00 O

ATOM 6250 N SER X 411 0.533 -22.885 -20.336 0.00 0.00 N

ATOM 6251 HN SER X 411 0.261 -22.588 -19.418 0.00 0.00 H

ATOM 6252 CA SER X 411 1.730 -23.688 -20.364 0.00 0.00 C

ATOM 6253 HA SER X 411 2.235 -23.505 -21.299 0.00 0.00 H

ATOM 6254 CB SER X 411 2.793 -23.222 -19.412 0.00 0.00 C

ATOM 6255 HB1 SER X 411 2.878 -22.130 -19.627 0.00 0.00 H

ATOM 6256 HB2 SER X 411 3.780 -23.720 -19.554 0.00 0.00 H

ATOM 6257 OG SER X 411 2.570 -23.279 -17.971 0.00 0.00 O

ATOM 6258 HG1 SER X 411 1.835 -22.697 -17.747 0.00 0.00 H

ATOM 6259 C SER X 411 1.571 -25.186 -20.363 0.00 0.00 C

ATOM 6260 O SER X 411 2.435 -25.879 -20.878 0.00 0.00 O

ATOM 6261 N LEU X 412 0.431 -25.673 -19.699 0.00 0.00 N

ATOM 6262 HN LEU X 412 -0.158 -25.146 -19.092 0.00 0.00 H

ATOM 6263 CA LEU X 412 0.112 -27.081 -19.852 0.00 0.00 C

ATOM 6264 HA LEU X 412 1.022 -27.627 -19.615 0.00 0.00 H

ATOM 6265 CB LEU X 412 -0.970 -27.620 -18.795 0.00 0.00 C

ATOM 6266 HB1 LEU X 412 -1.888 -27.017 -18.935 0.00 0.00 H

ATOM 6267 HB2 LEU X 412 -0.501 -27.259 -17.850 0.00 0.00 H

ATOM 6268 CG LEU X 412 -1.397 -28.992 -18.781 0.00 0.00 C

ATOM 6269 HG LEU X 412 -1.851 -29.227 -19.772 0.00 0.00 H

ATOM 6270 CD1 LEU X 412 -0.196 -29.986 -18.553 0.00 0.00 C

ATOM 6271 HD11 LEU X 412 0.087 -29.879 -17.491 0.00 0.00 H

ATOM 6272 HD12 LEU X 412 0.645 -29.751 -19.243 0.00 0.00 H

ATOM 6273 HD13 LEU X 412 -0.469 -31.060 -18.613 0.00 0.00 H

ATOM 6274 CD2 LEU X 412 -2.520 -29.198 -17.750 0.00 0.00 C

ATOM 6275 HD21 LEU X 412 -3.237 -28.387 -17.963 0.00 0.00 H

ATOM 6276 HD22 LEU X 412 -2.221 -29.189 -16.680 0.00 0.00 H

ATOM 6277 HD23 LEU X 412 -3.136 -30.089 -17.980 0.00 0.00 H

ATOM 6278 C LEU X 412 -0.360 -27.355 -21.296 0.00 0.00 C

ATOM 6279 O LEU X 412 0.019 -28.405 -21.790 0.00 0.00 O

ATOM 6280 N ALA X 413 -1.099 -26.440 -21.871 0.00 0.00 N

ATOM 6281 HN ALA X 413 -1.164 -25.670 -21.244 0.00 0.00 H

ATOM 6282 CA ALA X 413 -1.437 -26.254 -23.271 0.00 0.00 C

ATOM 6283 HA ALA X 413 -1.989 -27.125 -23.576 0.00 0.00 H

ATOM 6284 CB ALA X 413 -2.395 -25.064 -23.515 0.00 0.00 C

ATOM 6285 HB1 ALA X 413 -1.982 -24.038 -23.428 0.00 0.00 H

ATOM 6286 HB2 ALA X 413 -3.223 -25.126 -22.775 0.00 0.00 H

ATOM 6287 HB3 ALA X 413 -2.843 -25.237 -24.521 0.00 0.00 H

ATOM 6288 C ALA X 413 -0.244 -26.112 -24.139 0.00 0.00 C

ATOM 6289 O ALA X 413 0.817 -25.576 -23.726 0.00 0.00 O

ATOM 6290 N LYS X 414 -0.420 -26.589 -25.417 0.00 0.00 N

ATOM 6291 HN LYS X 414 -1.358 -26.803 -25.676 0.00 0.00 H

ATOM 6292 CA LYS X 414 0.639 -26.505 -26.415 0.00 0.00 C

ATOM 6293 HA LYS X 414 1.548 -26.021 -26.076 0.00 0.00 H

ATOM 6294 CB LYS X 414 0.991 -28.037 -26.679 0.00 0.00 C

ATOM 6295 HB1 LYS X 414 1.450 -28.272 -25.696 0.00 0.00 H

ATOM 6296 HB2 LYS X 414 1.759 -28.024 -27.483 0.00 0.00 H

ATOM 6297 CG LYS X 414 -0.213 -28.996 -27.008 0.00 0.00 C

ATOM 6298 HG1 LYS X 414 -0.701 -28.615 -27.934 0.00 0.00 H

ATOM 6299 HG2 LYS X 414 -0.924 -29.098 -26.162 0.00 0.00 H

ATOM 6300 CD LYS X 414 0.212 -30.472 -27.308 0.00 0.00 C

ATOM 6301 HD1 LYS X 414 0.950 -30.483 -28.144 0.00 0.00 H

ATOM 6302 HD2 LYS X 414 -0.721 -30.932 -27.702 0.00 0.00 H

ATOM 6303 CE LYS X 414 0.695 -31.378 -26.046 0.00 0.00 C

ATOM 6304 HE1 LYS X 414 -0.226 -31.573 -25.458 0.00 0.00 H

ATOM 6305 HE2 LYS X 414 1.612 -30.955 -25.570 0.00 0.00 H

ATOM 6306 NZ LYS X 414 1.182 -32.710 -26.559 0.00 0.00 N1+

ATOM 6307 HZ1 LYS X 414 1.579 -33.281 -25.782 0.00 0.00 H

ATOM 6308 HZ2 LYS X 414 1.904 -32.671 -27.300 0.00 0.00 H

ATOM 6309 HZ3 LYS X 414 0.305 -33.171 -26.876 0.00 0.00 H

ATOM 6310 C LYS X 414 0.185 -25.852 -27.718 0.00 0.00 C

ATOM 6311 O LYS X 414 1.042 -25.437 -28.499 0.00 0.00 O

ATOM 6312 N SER X 415 -1.173 -25.752 -27.964 0.00 0.00 N

ATOM 6313 HN SER X 415 -1.775 -26.209 -27.324 0.00 0.00 H

ATOM 6314 CA SER X 415 -1.863 -25.077 -29.031 0.00 0.00 C

ATOM 6315 HA SER X 415 -1.786 -25.793 -29.843 0.00 0.00 H

ATOM 6316 CB SER X 415 -3.393 -25.005 -28.739 0.00 0.00 C

ATOM 6317 HB1 SER X 415 -3.813 -26.022 -28.809 0.00 0.00 H

ATOM 6318 HB2 SER X 415 -3.975 -24.395 -29.479 0.00 0.00 H

ATOM 6319 OG SER X 415 -3.713 -24.499 -27.397 0.00 0.00 O

ATOM 6320 HG1 SER X 415 -3.621 -25.181 -26.736 0.00 0.00 H

ATOM 6321 C SER X 415 -1.490 -23.687 -29.527 0.00 0.00 C

ATOM 6322 O SER X 415 -1.054 -22.820 -28.690 0.00 0.00 O

ATOM 6323 N PRO X 416 -1.659 -23.386 -30.815 0.00 0.00 N

ATOM 6324 CD PRO X 416 -1.483 -24.313 -31.884 0.00 0.00 C

ATOM 6325 HD1 PRO X 416 -2.472 -24.807 -31.989 0.00 0.00 H

ATOM 6326 HD2 PRO X 416 -0.705 -25.062 -31.598 0.00 0.00 H

ATOM 6327 CA PRO X 416 -1.549 -22.001 -31.298 0.00 0.00 C

ATOM 6328 HA PRO X 416 -0.514 -21.709 -31.295 0.00 0.00 H

ATOM 6329 CB PRO X 416 -1.870 -22.226 -32.770 0.00 0.00 C

ATOM 6330 HB1 PRO X 416 -1.555 -21.364 -33.388 0.00 0.00 H

ATOM 6331 HB2 PRO X 416 -2.933 -22.519 -32.907 0.00 0.00 H

ATOM 6332 CG PRO X 416 -1.042 -23.494 -33.069 0.00 0.00 C

ATOM 6333 HG1 PRO X 416 -1.213 -24.101 -33.995 0.00 0.00 H

ATOM 6334 HG2 PRO X 416 0.037 -23.258 -32.977 0.00 0.00 H

ATOM 6335 C PRO X 416 -2.255 -20.980 -30.495 0.00 0.00 C

ATOM 6336 O PRO X 416 -3.415 -21.154 -30.177 0.00 0.00 O

ATOM 6337 N GLY X 417 -1.638 -19.761 -30.348 0.00 0.00 N

ATOM 6338 HN GLY X 417 -1.011 -19.580 -31.098 0.00 0.00 H

ATOM 6339 CA GLY X 417 -1.970 -18.684 -29.367 0.00 0.00 C

ATOM 6340 HA1 GLY X 417 -2.756 -18.996 -28.706 0.00 0.00 H

ATOM 6341 HA2 GLY X 417 -2.285 -17.840 -29.961 0.00 0.00 H

ATOM 6342 C GLY X 417 -0.653 -18.369 -28.720 0.00 0.00 C

ATOM 6343 O GLY X 417 0.404 -18.882 -28.863 0.00 0.00 O

ATOM 6344 N TRP X 418 -0.690 -17.403 -27.770 0.00 0.00 N

ATOM 6345 HN TRP X 418 -1.495 -16.833 -27.643 0.00 0.00 H

ATOM 6346 CA TRP X 418 0.393 -17.155 -26.922 0.00 0.00 C

ATOM 6347 HA TRP X 418 1.287 -16.939 -27.484 0.00 0.00 H

ATOM 6348 CB TRP X 418 0.080 -15.878 -26.073 0.00 0.00 C

ATOM 6349 HB1 TRP X 418 -0.845 -15.927 -25.465 0.00 0.00 H

ATOM 6350 HB2 TRP X 418 -0.118 -14.972 -26.699 0.00 0.00 H

ATOM 6351 CG TRP X 418 1.302 -15.621 -25.221 0.00 0.00 C

ATOM 6352 CD1 TRP X 418 2.318 -14.857 -25.554 0.00 0.00 C

ATOM 6353 HD1 TRP X 418 2.432 -14.381 -26.511 0.00 0.00 H

ATOM 6354 NE1 TRP X 418 3.217 -14.700 -24.650 0.00 0.00 N

ATOM 6355 HE1 TRP X 418 4.150 -14.432 -24.677 0.00 0.00 H

ATOM 6356 CE2 TRP X 418 2.910 -15.560 -23.618 0.00 0.00 C

ATOM 6357 CD2 TRP X 418 1.679 -16.071 -23.910 0.00 0.00 C

ATOM 6358 CE3 TRP X 418 1.063 -16.895 -22.965 0.00 0.00 C

ATOM 6359 HE3 TRP X 418 0.030 -17.183 -23.038 0.00 0.00 H

ATOM 6360 CZ3 TRP X 418 1.729 -16.994 -21.736 0.00 0.00 C

ATOM 6361 HZ3 TRP X 418 1.332 -17.593 -20.923 0.00 0.00 H

ATOM 6362 CZ2 TRP X 418 3.721 -15.891 -22.580 0.00 0.00 C

ATOM 6363 HZ2 TRP X 418 4.630 -15.342 -22.386 0.00 0.00 H

ATOM 6364 CH2 TRP X 418 3.073 -16.635 -21.603 0.00 0.00 C

ATOM 6365 HH2 TRP X 418 3.609 -16.853 -20.694 0.00 0.00 H

ATOM 6366 C TRP X 418 0.576 -18.354 -25.986 0.00 0.00 C

ATOM 6367 O TRP X 418 -0.409 -18.873 -25.333 0.00 0.00 O

ATOM 6368 N ARG X 419 1.791 -18.828 -25.993 0.00 0.00 N

ATOM 6369 HN ARG X 419 2.578 -18.412 -26.429 0.00 0.00 H

ATOM 6370 CA ARG X 419 2.291 -20.049 -25.252 0.00 0.00 C

ATOM 6371 HA ARG X 419 1.786 -20.000 -24.303 0.00 0.00 H

ATOM 6372 CB ARG X 419 2.175 -21.415 -25.991 0.00 0.00 C

ATOM 6373 HB1 ARG X 419 2.437 -22.276 -25.341 0.00 0.00 H

ATOM 6374 HB2 ARG X 419 2.851 -21.402 -26.876 0.00 0.00 H

ATOM 6375 CG ARG X 419 0.674 -21.836 -26.429 0.00 0.00 C

ATOM 6376 HG1 ARG X 419 0.812 -22.729 -27.071 0.00 0.00 H

ATOM 6377 HG2 ARG X 419 0.159 -21.038 -27.002 0.00 0.00 H

ATOM 6378 CD ARG X 419 -0.324 -22.216 -25.333 0.00 0.00 C

ATOM 6379 HD1 ARG X 419 -0.317 -21.425 -24.548 0.00 0.00 H

ATOM 6380 HD2 ARG X 419 -0.123 -23.156 -24.785 0.00 0.00 H

ATOM 6381 NE ARG X 419 -1.682 -22.354 -25.896 0.00 0.00 N

ATOM 6382 HE ARG X 419 -1.784 -22.834 -26.772 0.00 0.00 H

ATOM 6383 CZ ARG X 419 -2.745 -21.656 -25.524 0.00 0.00 C

ATOM 6384 NH1 ARG X 419 -2.780 -20.492 -24.819 0.00 0.00 N1+

ATOM 6385 HH11 ARG X 419 -1.946 -19.977 -24.591 0.00 0.00 H

ATOM 6386 HH12 ARG X 419 -3.597 -19.932 -24.965 0.00 0.00 H

ATOM 6387 NH2 ARG X 419 -3.910 -21.934 -26.106 0.00 0.00 N

ATOM 6388 HH21 ARG X 419 -3.797 -22.644 -26.793 0.00 0.00 H

ATOM 6389 HH22 ARG X 419 -4.574 -21.187 -26.077 0.00 0.00 H

ATOM 6390 C ARG X 419 3.771 -19.910 -25.090 0.00 0.00 C

ATOM 6391 O ARG X 419 4.382 -19.170 -25.829 0.00 0.00 O

ATOM 6392 N PRO X 420 4.452 -20.642 -24.221 0.00 0.00 N

ATOM 6393 CD PRO X 420 3.947 -21.209 -23.009 0.00 0.00 C

ATOM 6394 HD1 PRO X 420 3.893 -22.280 -23.307 0.00 0.00 H

ATOM 6395 HD2 PRO X 420 2.930 -20.857 -22.730 0.00 0.00 H

ATOM 6396 CA PRO X 420 5.883 -20.482 -24.106 0.00 0.00 C

ATOM 6397 HA PRO X 420 6.121 -19.429 -24.210 0.00 0.00 H

ATOM 6398 CB PRO X 420 6.236 -21.184 -22.761 0.00 0.00 C

ATOM 6399 HB1 PRO X 420 7.112 -20.856 -22.163 0.00 0.00 H

ATOM 6400 HB2 PRO X 420 6.431 -22.257 -22.971 0.00 0.00 H

ATOM 6401 CG PRO X 420 5.010 -21.005 -21.939 0.00 0.00 C

ATOM 6402 HG1 PRO X 420 4.981 -21.771 -21.135 0.00 0.00 H

ATOM 6403 HG2 PRO X 420 4.941 -19.977 -21.530 0.00 0.00 H

ATOM 6404 C PRO X 420 6.648 -21.147 -25.249 0.00 0.00 C

ATOM 6405 O PRO X 420 6.069 -21.906 -25.927 0.00 0.00 O

ATOM 6406 N ALA X 421 7.955 -20.763 -25.363 0.00 0.00 N

ATOM 6407 HN ALA X 421 8.263 -20.121 -24.667 0.00 0.00 H

ATOM 6408 CA ALA X 421 8.873 -21.426 -26.268 0.00 0.00 C

ATOM 6409 HA ALA X 421 8.513 -22.400 -26.585 0.00 0.00 H

ATOM 6410 CB ALA X 421 8.966 -20.631 -27.611 0.00 0.00 C

ATOM 6411 HB1 ALA X 421 7.937 -20.285 -27.847 0.00 0.00 H

ATOM 6412 HB2 ALA X 421 9.243 -21.301 -28.453 0.00 0.00 H

ATOM 6413 HB3 ALA X 421 9.640 -19.749 -27.636 0.00 0.00 H

ATOM 6414 C ALA X 421 10.206 -21.459 -25.596 0.00 0.00 C

ATOM 6415 O ALA X 421 10.683 -20.426 -25.068 0.00 0.00 O

ATOM 6416 N PHE X 422 10.849 -22.630 -25.528 0.00 0.00 N

ATOM 6417 HN PHE X 422 10.211 -23.385 -25.719 0.00 0.00 H

ATOM 6418 CA PHE X 422 12.254 -22.746 -25.095 0.00 0.00 C

ATOM 6419 HA PHE X 422 12.525 -23.699 -25.506 0.00 0.00 H

ATOM 6420 CB PHE X 422 13.264 -21.687 -25.630 0.00 0.00 C

ATOM 6421 HB1 PHE X 422 14.319 -21.950 -25.366 0.00 0.00 H

ATOM 6422 HB2 PHE X 422 12.986 -20.710 -25.191 0.00 0.00 H

ATOM 6423 CG PHE X 422 13.133 -21.820 -27.087 0.00 0.00 C

ATOM 6424 CD1 PHE X 422 13.703 -22.928 -27.869 0.00 0.00 C

ATOM 6425 HD1 PHE X 422 14.374 -23.610 -27.377 0.00 0.00 H

ATOM 6426 CE1 PHE X 422 13.321 -23.077 -29.201 0.00 0.00 C

ATOM 6427 HE1 PHE X 422 13.546 -23.983 -29.741 0.00 0.00 H

ATOM 6428 CZ PHE X 422 12.586 -22.093 -29.820 0.00 0.00 C

ATOM 6429 HZ PHE X 422 12.314 -22.344 -30.841 0.00 0.00 H

ATOM 6430 CD2 PHE X 422 12.364 -20.832 -27.781 0.00 0.00 C

ATOM 6431 HD2 PHE X 422 11.921 -20.135 -27.089 0.00 0.00 H

ATOM 6432 CE2 PHE X 422 12.133 -20.917 -29.111 0.00 0.00 C

ATOM 6433 HE2 PHE X 422 11.567 -20.301 -29.798 0.00 0.00 H

ATOM 6434 C PHE X 422 12.503 -22.881 -23.607 0.00 0.00 C

ATOM 6435 O PHE X 422 13.362 -23.628 -23.091 0.00 0.00 O

ATOM 6436 N LYS X 423 11.791 -22.108 -22.728 0.00 0.00 N

ATOM 6437 HN LYS X 423 11.305 -21.305 -23.056 0.00 0.00 H

ATOM 6438 CA LYS X 423 11.781 -22.214 -21.271 0.00 0.00 C

ATOM 6439 HA LYS X 423 12.067 -23.229 -21.061 0.00 0.00 H

ATOM 6440 CB LYS X 423 12.774 -21.140 -20.750 0.00 0.00 C

ATOM 6441 HB1 LYS X 423 12.582 -20.867 -19.690 0.00 0.00 H

ATOM 6442 HB2 LYS X 423 12.503 -20.145 -21.168 0.00 0.00 H

ATOM 6443 CG LYS X 423 14.248 -21.211 -20.927 0.00 0.00 C

ATOM 6444 HG1 LYS X 423 14.715 -20.383 -20.356 0.00 0.00 H

ATOM 6445 HG2 LYS X 423 14.618 -21.194 -21.976 0.00 0.00 H

ATOM 6446 CD LYS X 423 14.837 -22.446 -20.105 0.00 0.00 C

ATOM 6447 HD1 LYS X 423 14.417 -23.397 -20.491 0.00 0.00 H

ATOM 6448 HD2 LYS X 423 14.498 -22.295 -19.053 0.00 0.00 H

ATOM 6449 CE LYS X 423 16.338 -22.619 -20.202 0.00 0.00 C

ATOM 6450 HE1 LYS X 423 16.765 -21.602 -20.243 0.00 0.00 H

ATOM 6451 HE2 LYS X 423 16.651 -23.155 -21.120 0.00 0.00 H

ATOM 6452 NZ LYS X 423 16.858 -23.269 -19.013 0.00 0.00 N1+

ATOM 6453 HZ1 LYS X 423 16.690 -22.727 -18.140 0.00 0.00 H

ATOM 6454 HZ2 LYS X 423 17.891 -23.376 -19.085 0.00 0.00 H

ATOM 6455 HZ3 LYS X 423 16.463 -24.232 -18.912 0.00 0.00 H

ATOM 6456 C LYS X 423 10.426 -22.080 -20.591 0.00 0.00 C

ATOM 6457 O LYS X 423 9.493 -21.606 -21.228 0.00 0.00 O

ATOM 6458 N TYR X 424 10.204 -22.443 -19.351 0.00 0.00 N

ATOM 6459 HN TYR X 424 10.865 -22.887 -18.755 0.00 0.00 H

ATOM 6460 CA TYR X 424 8.870 -22.120 -18.721 0.00 0.00 C

ATOM 6461 HA TYR X 424 9.119 -22.406 -17.707 0.00 0.00 H

ATOM 6462 CB TYR X 424 8.549 -20.612 -18.695 0.00 0.00 C

ATOM 6463 HB1 TYR X 424 7.639 -20.432 -18.081 0.00 0.00 H

ATOM 6464 HB2 TYR X 424 8.112 -20.301 -19.671 0.00 0.00 H

ATOM 6465 CG TYR X 424 9.584 -19.602 -18.145 0.00 0.00 C

ATOM 6466 CD1 TYR X 424 9.645 -19.492 -16.684 0.00 0.00 C

ATOM 6467 HD1 TYR X 424 9.132 -20.236 -16.096 0.00 0.00 H

ATOM 6468 CE1 TYR X 424 10.522 -18.649 -16.050 0.00 0.00 C

ATOM 6469 HE1 TYR X 424 10.660 -18.615 -14.988 0.00 0.00 H

ATOM 6470 CZ TYR X 424 11.361 -17.859 -16.918 0.00 0.00 C

ATOM 6471 OH TYR X 424 12.392 -17.002 -16.398 0.00 0.00 O

ATOM 6472 HH TYR X 424 13.213 -17.422 -16.664 0.00 0.00 H

ATOM 6473 CD2 TYR X 424 10.329 -18.753 -18.917 0.00 0.00 C

ATOM 6474 HD2 TYR X 424 10.263 -18.767 -20.002 0.00 0.00 H

ATOM 6475 CE2 TYR X 424 11.259 -17.921 -18.266 0.00 0.00 C

ATOM 6476 HE2 TYR X 424 11.938 -17.430 -18.950 0.00 0.00 H

ATOM 6477 C TYR X 424 7.703 -22.890 -19.181 0.00 0.00 C

ATOM 6478 O TYR X 424 6.541 -22.472 -19.043 0.00 0.00 O

ATOM 6479 N TYR X 425 7.974 -24.141 -19.643 0.00 0.00 N

ATOM 6480 HN TYR X 425 8.918 -24.368 -19.887 0.00 0.00 H

ATOM 6481 CA TYR X 425 6.916 -25.125 -19.904 0.00 0.00 C

ATOM 6482 HA TYR X 425 6.072 -24.654 -20.381 0.00 0.00 H

ATOM 6483 CB TYR X 425 7.420 -26.397 -20.763 0.00 0.00 C

ATOM 6484 HB1 TYR X 425 6.624 -27.150 -20.961 0.00 0.00 H

ATOM 6485 HB2 TYR X 425 8.230 -26.864 -20.170 0.00 0.00 H

ATOM 6486 CG TYR X 425 7.896 -25.953 -22.112 0.00 0.00 C

ATOM 6487 CD1 TYR X 425 9.136 -26.386 -22.563 0.00 0.00 C

ATOM 6488 HD1 TYR X 425 9.686 -27.127 -22.000 0.00 0.00 H

ATOM 6489 CE1 TYR X 425 9.524 -25.928 -23.821 0.00 0.00 C

ATOM 6490 HE1 TYR X 425 10.500 -26.134 -24.245 0.00 0.00 H

ATOM 6491 CZ TYR X 425 8.621 -25.265 -24.649 0.00 0.00 C

ATOM 6492 OH TYR X 425 9.077 -25.067 -25.933 0.00 0.00 O

ATOM 6493 HH TYR X 425 9.892 -25.566 -26.033 0.00 0.00 H

ATOM 6494 CD2 TYR X 425 7.020 -25.228 -22.880 0.00 0.00 C

ATOM 6495 HD2 TYR X 425 5.997 -25.141 -22.564 0.00 0.00 H

ATOM 6496 CE2 TYR X 425 7.350 -24.928 -24.215 0.00 0.00 C

ATOM 6497 HE2 TYR X 425 6.598 -24.422 -24.805 0.00 0.00 H

ATOM 6498 C TYR X 425 6.409 -25.783 -18.636 0.00 0.00 C

ATOM 6499 O TYR X 425 7.077 -25.875 -17.539 0.00 0.00 O

ATOM 6500 N ASN X 426 5.104 -26.133 -18.543 0.00 0.00 N

ATOM 6501 HN ASN X 426 4.483 -26.000 -19.305 0.00 0.00 H

ATOM 6502 CA ASN X 426 4.538 -26.763 -17.394 0.00 0.00 C

ATOM 6503 HA ASN X 426 3.482 -26.648 -17.597 0.00 0.00 H

ATOM 6504 CB ASN X 426 4.721 -28.326 -17.331 0.00 0.00 C

ATOM 6505 HB1 ASN X 426 4.369 -28.651 -16.329 0.00 0.00 H

ATOM 6506 HB2 ASN X 426 5.745 -28.697 -17.517 0.00 0.00 H

ATOM 6507 CG ASN X 426 3.821 -29.075 -18.397 0.00 0.00 C

ATOM 6508 OD1 ASN X 426 3.358 -28.431 -19.287 0.00 0.00 O

ATOM 6509 ND2 ASN X 426 3.796 -30.444 -18.289 0.00 0.00 N

ATOM 6510 HD21 ASN X 426 3.333 -30.940 -19.022 0.00 0.00 H

ATOM 6511 HD22 ASN X 426 4.461 -30.859 -17.675 0.00 0.00 H

ATOM 6512 C ASN X 426 4.637 -26.185 -15.980 0.00 0.00 C

ATOM 6513 O ASN X 426 5.189 -26.818 -15.091 0.00 0.00 O

ATOM 6514 N MET X 427 4.080 -24.979 -15.687 0.00 0.00 N

ATOM 6515 HN MET X 427 3.574 -24.536 -16.427 0.00 0.00 H

ATOM 6516 CA MET X 427 4.373 -24.270 -14.523 0.00 0.00 C

ATOM 6517 HA MET X 427 5.432 -24.372 -14.330 0.00 0.00 H

ATOM 6518 CB MET X 427 4.187 -22.670 -14.740 0.00 0.00 C

ATOM 6519 HB1 MET X 427 4.437 -22.370 -13.704 0.00 0.00 H

ATOM 6520 HB2 MET X 427 3.200 -22.369 -15.150 0.00 0.00 H

ATOM 6521 CG MET X 427 5.158 -22.084 -15.711 0.00 0.00 C

ATOM 6522 HG1 MET X 427 4.941 -21.000 -15.837 0.00 0.00 H

ATOM 6523 HG2 MET X 427 4.992 -22.585 -16.684 0.00 0.00 H

ATOM 6524 SD MET X 427 6.890 -22.000 -15.326 0.00 0.00 S

ATOM 6525 CE MET X 427 6.734 -20.948 -13.800 0.00 0.00 C

ATOM 6526 HE1 MET X 427 7.720 -20.883 -13.273 0.00 0.00 H

ATOM 6527 HE2 MET X 427 6.144 -21.422 -12.986 0.00 0.00 H

ATOM 6528 HE3 MET X 427 6.348 -19.970 -14.139 0.00 0.00 H

ATOM 6529 C MET X 427 3.689 -24.777 -13.233 0.00 0.00 C

ATOM 6530 O MET X 427 2.862 -24.038 -12.656 0.00 0.00 O

ATOM 6531 N TRP X 428 4.040 -25.868 -12.669 0.00 0.00 N

ATOM 6532 HN TRP X 428 4.572 -26.572 -13.148 0.00 0.00 H

ATOM 6533 CA TRP X 428 3.688 -26.330 -11.346 0.00 0.00 C

ATOM 6534 HA TRP X 428 2.649 -26.615 -11.369 0.00 0.00 H

ATOM 6535 CB TRP X 428 4.662 -27.499 -11.021 0.00 0.00 C

ATOM 6536 HB1 TRP X 428 4.163 -28.122 -10.237 0.00 0.00 H

ATOM 6537 HB2 TRP X 428 5.642 -27.142 -10.635 0.00 0.00 H

ATOM 6538 CG TRP X 428 4.921 -28.473 -12.127 0.00 0.00 C

ATOM 6539 CD1 TRP X 428 6.212 -28.693 -12.597 0.00 0.00 C

ATOM 6540 HD1 TRP X 428 7.077 -28.103 -12.330 0.00 0.00 H

ATOM 6541 NE1 TRP X 428 6.117 -29.733 -13.526 0.00 0.00 N

ATOM 6542 HE1 TRP X 428 6.764 -30.169 -14.112 0.00 0.00 H

ATOM 6543 CE2 TRP X 428 4.833 -30.080 -13.603 0.00 0.00 C

ATOM 6544 CD2 TRP X 428 4.023 -29.293 -12.846 0.00 0.00 C

ATOM 6545 CE3 TRP X 428 2.645 -29.458 -12.814 0.00 0.00 C

ATOM 6546 HE3 TRP X 428 2.016 -28.954 -12.090 0.00 0.00 H

ATOM 6547 CZ3 TRP X 428 2.073 -30.310 -13.758 0.00 0.00 C

ATOM 6548 HZ3 TRP X 428 1.003 -30.393 -13.919 0.00 0.00 H

ATOM 6549 CZ2 TRP X 428 4.250 -31.064 -14.488 0.00 0.00 C

ATOM 6550 HZ2 TRP X 428 4.961 -31.553 -15.144 0.00 0.00 H

ATOM 6551 CH2 TRP X 428 2.863 -31.137 -14.534 0.00 0.00 C

ATOM 6552 HH2 TRP X 428 2.453 -31.761 -15.314 0.00 0.00 H

ATOM 6553 C TRP X 428 3.817 -25.345 -10.187 0.00 0.00 C

ATOM 6554 O TRP X 428 2.890 -25.340 -9.361 0.00 0.00 O

ATOM 6555 N ILE X 429 4.814 -24.501 -10.124 0.00 0.00 N

ATOM 6556 HN ILE X 429 5.673 -24.507 -10.635 0.00 0.00 H

ATOM 6557 CA ILE X 429 4.958 -23.468 -9.129 0.00 0.00 C

ATOM 6558 HA ILE X 429 4.780 -23.887 -8.153 0.00 0.00 H

ATOM 6559 CB ILE X 429 6.375 -22.960 -9.058 0.00 0.00 C

ATOM 6560 HB ILE X 429 6.723 -22.663 -10.068 0.00 0.00 H

ATOM 6561 CG2 ILE X 429 6.477 -21.677 -8.154 0.00 0.00 C

ATOM 6562 HG21 ILE X 429 6.108 -20.760 -8.669 0.00 0.00 H

ATOM 6563 HG22 ILE X 429 7.552 -21.576 -7.883 0.00 0.00 H

ATOM 6564 HG23 ILE X 429 5.865 -21.824 -7.234 0.00 0.00 H

ATOM 6565 CG1 ILE X 429 7.281 -24.099 -8.541 0.00 0.00 C

ATOM 6566 HG11 ILE X 429 8.283 -23.628 -8.574 0.00 0.00 H

ATOM 6567 HG12 ILE X 429 7.406 -24.990 -9.194 0.00 0.00 H

ATOM 6568 CD ILE X 429 6.965 -24.520 -7.096 0.00 0.00 C

ATOM 6569 HD1 ILE X 429 7.244 -23.734 -6.351 0.00 0.00 H

ATOM 6570 HD2 ILE X 429 7.557 -25.419 -6.830 0.00 0.00 H

ATOM 6571 HD3 ILE X 429 5.939 -24.935 -7.069 0.00 0.00 H

ATOM 6572 C ILE X 429 3.890 -22.395 -9.256 0.00 0.00 C

ATOM 6573 O ILE X 429 3.401 -21.892 -8.254 0.00 0.00 O

ATOM 6574 N SER X 430 3.503 -22.083 -10.468 0.00 0.00 N

ATOM 6575 HN SER X 430 3.994 -22.355 -11.294 0.00 0.00 H

ATOM 6576 CA SER X 430 2.361 -21.123 -10.697 0.00 0.00 C

ATOM 6577 HA SER X 430 2.519 -20.254 -10.082 0.00 0.00 H

ATOM 6578 CB SER X 430 2.313 -20.593 -12.139 0.00 0.00 C

ATOM 6579 HB1 SER X 430 1.514 -19.842 -12.306 0.00 0.00 H

ATOM 6580 HB2 SER X 430 2.000 -21.406 -12.824 0.00 0.00 H

ATOM 6581 OG SER X 430 3.597 -20.110 -12.565 0.00 0.00 O

ATOM 6582 HG1 SER X 430 3.573 -19.150 -12.510 0.00 0.00 H

ATOM 6583 C SER X 430 0.991 -21.820 -10.447 0.00 0.00 C

ATOM 6584 O SER X 430 -0.008 -21.237 -10.071 0.00 0.00 O

ATOM 6585 N LEU X 431 0.944 -23.146 -10.616 0.00 0.00 N

ATOM 6586 HN LEU X 431 1.752 -23.570 -11.014 0.00 0.00 H

ATOM 6587 CA LEU X 431 -0.234 -23.850 -10.285 0.00 0.00 C

ATOM 6588 HA LEU X 431 -1.042 -23.275 -10.714 0.00 0.00 H

ATOM 6589 CB LEU X 431 -0.354 -25.388 -10.851 0.00 0.00 C

ATOM 6590 HB1 LEU X 431 0.650 -25.865 -10.740 0.00 0.00 H

ATOM 6591 HB2 LEU X 431 -0.432 -25.329 -11.962 0.00 0.00 H

ATOM 6592 CG LEU X 431 -1.486 -26.239 -10.233 0.00 0.00 C

ATOM 6593 HG LEU X 431 -1.222 -26.410 -9.164 0.00 0.00 H

ATOM 6594 CD1 LEU X 431 -2.910 -25.571 -10.342 0.00 0.00 C

ATOM 6595 HD11 LEU X 431 -3.208 -25.495 -11.408 0.00 0.00 H

ATOM 6596 HD12 LEU X 431 -2.778 -24.580 -9.859 0.00 0.00 H

ATOM 6597 HD13 LEU X 431 -3.487 -26.280 -9.712 0.00 0.00 H

ATOM 6598 CD2 LEU X 431 -1.451 -27.681 -10.849 0.00 0.00 C

ATOM 6599 HD21 LEU X 431 -2.105 -28.392 -10.298 0.00 0.00 H

ATOM 6600 HD22 LEU X 431 -0.388 -27.889 -11.092 0.00 0.00 H

ATOM 6601 HD23 LEU X 431 -1.753 -27.669 -11.921 0.00 0.00 H

ATOM 6602 C LEU X 431 -0.447 -23.861 -8.735 0.00 0.00 C

ATOM 6603 O LEU X 431 -1.358 -23.295 -8.216 0.00 0.00 O

ATOM 6604 N LEU X 432 0.584 -24.262 -7.950 0.00 0.00 N

ATOM 6605 HN LEU X 432 1.255 -24.835 -8.414 0.00 0.00 H

ATOM 6606 CA LEU X 432 0.702 -23.906 -6.570 0.00 0.00 C

ATOM 6607 HA LEU X 432 -0.105 -24.438 -6.088 0.00 0.00 H

ATOM 6608 CB LEU X 432 2.141 -24.551 -6.189 0.00 0.00 C

ATOM 6609 HB1 LEU X 432 2.876 -24.004 -6.830 0.00 0.00 H

ATOM 6610 HB2 LEU X 432 2.107 -25.638 -6.443 0.00 0.00 H

ATOM 6611 CG LEU X 432 2.477 -24.385 -4.691 0.00 0.00 C

ATOM 6612 HG LEU X 432 2.515 -23.305 -4.426 0.00 0.00 H

ATOM 6613 CD1 LEU X 432 1.550 -25.192 -3.791 0.00 0.00 C

ATOM 6614 HD11 LEU X 432 0.549 -24.738 -3.900 0.00 0.00 H

ATOM 6615 HD12 LEU X 432 1.945 -25.231 -2.747 0.00 0.00 H

ATOM 6616 HD13 LEU X 432 1.594 -26.267 -4.068 0.00 0.00 H

ATOM 6617 CD2 LEU X 432 3.983 -24.760 -4.431 0.00 0.00 C

ATOM 6618 HD21 LEU X 432 4.125 -25.813 -4.769 0.00 0.00 H

ATOM 6619 HD22 LEU X 432 4.167 -24.690 -3.337 0.00 0.00 H

ATOM 6620 HD23 LEU X 432 4.580 -24.016 -5.000 0.00 0.00 H

ATOM 6621 C LEU X 432 0.528 -22.510 -6.137 0.00 0.00 C

ATOM 6622 O LEU X 432 -0.275 -22.230 -5.235 0.00 0.00 O

ATOM 6623 N GLY X 433 1.162 -21.613 -6.823 0.00 0.00 N

ATOM 6624 HN GLY X 433 1.813 -21.883 -7.531 0.00 0.00 H

ATOM 6625 CA GLY X 433 1.033 -20.181 -6.519 0.00 0.00 C

ATOM 6626 HA1 GLY X 433 1.600 -19.694 -7.306 0.00 0.00 H

ATOM 6627 HA2 GLY X 433 1.368 -20.022 -5.505 0.00 0.00 H

ATOM 6628 C GLY X 433 -0.252 -19.603 -6.728 0.00 0.00 C

ATOM 6629 O GLY X 433 -0.700 -18.636 -6.155 0.00 0.00 O

ATOM 6630 N ALA X 434 -1.017 -20.094 -7.716 0.00 0.00 N

ATOM 6631 HN ALA X 434 -0.528 -20.744 -8.295 0.00 0.00 H

ATOM 6632 CA ALA X 434 -2.437 -19.726 -7.916 0.00 0.00 C

ATOM 6633 HA ALA X 434 -2.470 -18.643 -7.891 0.00 0.00 H

ATOM 6634 CB ALA X 434 -2.881 -20.303 -9.237 0.00 0.00 C

ATOM 6635 HB1 ALA X 434 -2.913 -21.416 -9.257 0.00 0.00 H

ATOM 6636 HB2 ALA X 434 -2.123 -20.099 -10.014 0.00 0.00 H

ATOM 6637 HB3 ALA X 434 -3.759 -19.802 -9.688 0.00 0.00 H

ATOM 6638 C ALA X 434 -3.343 -20.277 -6.839 0.00 0.00 C

ATOM 6639 O ALA X 434 -4.140 -19.545 -6.279 0.00 0.00 O

ATOM 6640 N ILE X 435 -3.155 -21.523 -6.433 0.00 0.00 N

ATOM 6641 HN ILE X 435 -2.533 -22.048 -7.016 0.00 0.00 H

ATOM 6642 CA ILE X 435 -3.761 -22.151 -5.258 0.00 0.00 C

ATOM 6643 HA ILE X 435 -4.792 -22.277 -5.564 0.00 0.00 H

ATOM 6644 CB ILE X 435 -3.207 -23.610 -4.919 0.00 0.00 C

ATOM 6645 HB ILE X 435 -2.113 -23.674 -4.715 0.00 0.00 H

ATOM 6646 CG2 ILE X 435 -3.884 -24.130 -3.578 0.00 0.00 C

ATOM 6647 HG21 ILE X 435 -4.991 -24.036 -3.513 0.00 0.00 H

ATOM 6648 HG22 ILE X 435 -3.471 -23.456 -2.801 0.00 0.00 H

ATOM 6649 HG23 ILE X 435 -3.603 -25.121 -3.179 0.00 0.00 H

ATOM 6650 CG1 ILE X 435 -3.466 -24.584 -6.062 0.00 0.00 C

ATOM 6651 HG11 ILE X 435 -3.095 -25.590 -5.764 0.00 0.00 H

ATOM 6652 HG12 ILE X 435 -2.781 -24.200 -6.850 0.00 0.00 H

ATOM 6653 CD ILE X 435 -4.932 -24.788 -6.501 0.00 0.00 C

ATOM 6654 HD1 ILE X 435 -5.595 -24.173 -5.858 0.00 0.00 H

ATOM 6655 HD2 ILE X 435 -5.221 -25.805 -6.163 0.00 0.00 H

ATOM 6656 HD3 ILE X 435 -5.080 -24.503 -7.574 0.00 0.00 H

ATOM 6657 C ILE X 435 -3.548 -21.356 -3.957 0.00 0.00 C

ATOM 6658 O ILE X 435 -4.442 -20.907 -3.227 0.00 0.00 O

ATOM 6659 N LEU X 436 -2.259 -21.016 -3.698 0.00 0.00 N

ATOM 6660 HN LEU X 436 -1.607 -21.399 -4.357 0.00 0.00 H

ATOM 6661 CA LEU X 436 -1.640 -20.365 -2.504 0.00 0.00 C

ATOM 6662 HA LEU X 436 -2.172 -20.902 -1.730 0.00 0.00 H

ATOM 6663 CB LEU X 436 -0.095 -20.612 -2.299 0.00 0.00 C

ATOM 6664 HB1 LEU X 436 0.305 -20.237 -3.258 0.00 0.00 H

ATOM 6665 HB2 LEU X 436 0.170 -21.693 -2.208 0.00 0.00 H

ATOM 6666 CG LEU X 436 0.656 -19.825 -1.200 0.00 0.00 C

ATOM 6667 HG LEU X 436 0.397 -18.743 -1.230 0.00 0.00 H

ATOM 6668 CD1 LEU X 436 0.206 -20.469 0.154 0.00 0.00 C

ATOM 6669 HD11 LEU X 436 0.471 -19.757 0.962 0.00 0.00 H

ATOM 6670 HD12 LEU X 436 0.702 -21.454 0.354 0.00 0.00 H

ATOM 6671 HD13 LEU X 436 -0.901 -20.556 0.231 0.00 0.00 H

ATOM 6672 CD2 LEU X 436 2.196 -19.871 -1.352 0.00 0.00 C

ATOM 6673 HD21 LEU X 436 2.756 -19.553 -0.445 0.00 0.00 H

ATOM 6674 HD22 LEU X 436 2.456 -19.226 -2.223 0.00 0.00 H

ATOM 6675 HD23 LEU X 436 2.509 -20.873 -1.705 0.00 0.00 H

ATOM 6676 C LEU X 436 -2.048 -18.965 -2.228 0.00 0.00 C

ATOM 6677 O LEU X 436 -2.245 -18.514 -1.090 0.00 0.00 O

ATOM 6678 N CYS X 437 -2.299 -18.082 -3.292 0.00 0.00 N

ATOM 6679 HN CYS X 437 -1.987 -18.386 -4.185 0.00 0.00 H

ATOM 6680 CA CYS X 437 -2.832 -16.781 -3.215 0.00 0.00 C

ATOM 6681 HA CYS X 437 -2.341 -16.357 -2.352 0.00 0.00 H

ATOM 6682 CB CYS X 437 -2.784 -15.982 -4.482 0.00 0.00 C

ATOM 6683 HB1 CYS X 437 -3.402 -15.055 -4.430 0.00 0.00 H

ATOM 6684 HB2 CYS X 437 -3.250 -16.575 -5.290 0.00 0.00 H

ATOM 6685 SG CYS X 437 -1.145 -15.794 -5.253 0.00 0.00 S

ATOM 6686 HG1 CYS X 437 -0.919 -17.094 -5.243 0.00 0.00 H

ATOM 6687 C CYS X 437 -4.325 -17.008 -2.818 0.00 0.00 C

ATOM 6688 O CYS X 437 -4.775 -16.414 -1.868 0.00 0.00 O

ATOM 6689 N CYS X 438 -5.115 -17.874 -3.603 0.00 0.00 N

ATOM 6690 HN CYS X 438 -4.765 -18.313 -4.433 0.00 0.00 H

ATOM 6691 CA CYS X 438 -6.537 -18.085 -3.341 0.00 0.00 C

ATOM 6692 HA CYS X 438 -7.064 -17.149 -3.481 0.00 0.00 H

ATOM 6693 CB CYS X 438 -7.091 -19.158 -4.308 0.00 0.00 C

ATOM 6694 HB1 CYS X 438 -8.026 -19.649 -3.964 0.00 0.00 H

ATOM 6695 HB2 CYS X 438 -6.279 -19.902 -4.412 0.00 0.00 H

ATOM 6696 SG CYS X 438 -7.519 -18.399 -6.018 0.00 0.00 S

ATOM 6697 HG1 CYS X 438 -6.425 -18.662 -6.718 0.00 0.00 H

ATOM 6698 C CYS X 438 -6.844 -18.588 -1.919 0.00 0.00 C

ATOM 6699 O CYS X 438 -7.810 -18.233 -1.343 0.00 0.00 O

ATOM 6700 N ILE X 439 -6.066 -19.592 -1.385 0.00 0.00 N

ATOM 6701 HN ILE X 439 -5.325 -20.071 -1.857 0.00 0.00 H

ATOM 6702 CA ILE X 439 -6.365 -20.240 -0.084 0.00 0.00 C

ATOM 6703 HA ILE X 439 -7.394 -20.577 -0.093 0.00 0.00 H

ATOM 6704 CB ILE X 439 -5.630 -21.545 0.143 0.00 0.00 C

ATOM 6705 HB ILE X 439 -5.708 -22.127 -0.794 0.00 0.00 H

ATOM 6706 CG2 ILE X 439 -4.101 -21.382 0.420 0.00 0.00 C

ATOM 6707 HG21 ILE X 439 -3.565 -22.345 0.321 0.00 0.00 H

ATOM 6708 HG22 ILE X 439 -3.941 -20.964 1.438 0.00 0.00 H

ATOM 6709 HG23 ILE X 439 -3.704 -20.520 -0.158 0.00 0.00 H

ATOM 6710 CG1 ILE X 439 -6.240 -22.376 1.271 0.00 0.00 C

ATOM 6711 HG11 ILE X 439 -7.305 -22.050 1.244 0.00 0.00 H

ATOM 6712 HG12 ILE X 439 -5.941 -22.186 2.330 0.00 0.00 H

ATOM 6713 CD ILE X 439 -6.181 -23.872 0.853 0.00 0.00 C

ATOM 6714 HD1 ILE X 439 -6.708 -24.019 -0.113 0.00 0.00 H

ATOM 6715 HD2 ILE X 439 -6.761 -24.326 1.676 0.00 0.00 H

ATOM 6716 HD3 ILE X 439 -5.127 -24.182 0.696 0.00 0.00 H

ATOM 6717 C ILE X 439 -6.216 -19.234 1.126 0.00 0.00 C

ATOM 6718 O ILE X 439 -7.167 -19.195 1.967 0.00 0.00 O

ATOM 6719 N VAL X 440 -5.200 -18.375 1.119 0.00 0.00 N

ATOM 6720 HN VAL X 440 -4.640 -18.357 0.302 0.00 0.00 H

ATOM 6721 CA VAL X 440 -4.943 -17.350 2.127 0.00 0.00 C

ATOM 6722 HA VAL X 440 -4.607 -17.757 3.068 0.00 0.00 H

ATOM 6723 CB VAL X 440 -3.651 -16.676 1.639 0.00 0.00 C

ATOM 6724 HB VAL X 440 -3.690 -16.391 0.567 0.00 0.00 H

ATOM 6725 CG1 VAL X 440 -3.248 -15.461 2.530 0.00 0.00 C

ATOM 6726 HG11 VAL X 440 -4.071 -14.741 2.684 0.00 0.00 H

ATOM 6727 HG12 VAL X 440 -2.431 -14.888 2.026 0.00 0.00 H

ATOM 6728 HG13 VAL X 440 -2.828 -15.833 3.487 0.00 0.00 H

ATOM 6729 CG2 VAL X 440 -2.455 -17.730 1.638 0.00 0.00 C

ATOM 6730 HG21 VAL X 440 -1.735 -17.432 0.850 0.00 0.00 H

ATOM 6731 HG22 VAL X 440 -2.715 -18.766 1.356 0.00 0.00 H

ATOM 6732 HG23 VAL X 440 -1.995 -17.754 2.647 0.00 0.00 H

ATOM 6733 C VAL X 440 -6.036 -16.307 2.332 0.00 0.00 C

ATOM 6734 O VAL X 440 -6.463 -16.030 3.416 0.00 0.00 O

ATOM 6735 N MET X 441 -6.653 -15.813 1.274 0.00 0.00 N

ATOM 6736 HN MET X 441 -6.280 -16.157 0.410 0.00 0.00 H

ATOM 6737 CA MET X 441 -7.894 -15.100 1.223 0.00 0.00 C

ATOM 6738 HA MET X 441 -7.608 -14.073 1.397 0.00 0.00 H

ATOM 6739 CB MET X 441 -8.485 -15.249 -0.233 0.00 0.00 C

ATOM 6740 HB1 MET X 441 -9.371 -14.582 -0.233 0.00 0.00 H

ATOM 6741 HB2 MET X 441 -8.834 -16.286 -0.445 0.00 0.00 H

ATOM 6742 CG MET X 441 -7.660 -14.591 -1.377 0.00 0.00 C

ATOM 6743 HG1 MET X 441 -6.612 -14.964 -1.338 0.00 0.00 H

ATOM 6744 HG2 MET X 441 -7.494 -13.542 -1.065 0.00 0.00 H

ATOM 6745 SD MET X 441 -8.160 -14.814 -3.100 0.00 0.00 S

ATOM 6746 CE MET X 441 -9.847 -14.216 -2.890 0.00 0.00 C

ATOM 6747 HE1 MET X 441 -10.359 -14.187 -3.876 0.00 0.00 H

ATOM 6748 HE2 MET X 441 -9.848 -13.155 -2.568 0.00 0.00 H

ATOM 6749 HE3 MET X 441 -10.426 -14.774 -2.120 0.00 0.00 H

ATOM 6750 C MET X 441 -9.013 -15.509 2.257 0.00 0.00 C

ATOM 6751 O MET X 441 -9.471 -14.711 3.051 0.00 0.00 O

ATOM 6752 N PHE X 442 -9.425 -16.776 2.256 0.00 0.00 N

ATOM 6753 HN PHE X 442 -8.895 -17.419 1.711 0.00 0.00 H

ATOM 6754 CA PHE X 442 -10.525 -17.316 3.038 0.00 0.00 C

ATOM 6755 HA PHE X 442 -11.352 -16.637 2.933 0.00 0.00 H

ATOM 6756 CB PHE X 442 -10.780 -18.742 2.631 0.00 0.00 C

ATOM 6757 HB1 PHE X 442 -11.628 -19.220 3.175 0.00 0.00 H

ATOM 6758 HB2 PHE X 442 -9.907 -19.422 2.600 0.00 0.00 H

ATOM 6759 CG PHE X 442 -11.379 -18.794 1.218 0.00 0.00 C

ATOM 6760 CD1 PHE X 442 -12.725 -18.577 0.876 0.00 0.00 C

ATOM 6761 HD1 PHE X 442 -13.467 -18.518 1.648 0.00 0.00 H

ATOM 6762 CE1 PHE X 442 -13.143 -18.609 -0.559 0.00 0.00 C

ATOM 6763 HE1 PHE X 442 -14.169 -18.378 -0.767 0.00 0.00 H

ATOM 6764 CZ PHE X 442 -12.199 -18.892 -1.556 0.00 0.00 C

ATOM 6765 HZ PHE X 442 -12.471 -18.783 -2.601 0.00 0.00 H

ATOM 6766 CD2 PHE X 442 -10.414 -18.991 0.127 0.00 0.00 C

ATOM 6767 HD2 PHE X 442 -9.372 -19.108 0.380 0.00 0.00 H

ATOM 6768 CE2 PHE X 442 -10.828 -19.071 -1.243 0.00 0.00 C

ATOM 6769 HE2 PHE X 442 -10.067 -19.213 -1.998 0.00 0.00 H

ATOM 6770 C PHE X 442 -10.250 -17.311 4.540 0.00 0.00 C

ATOM 6771 O PHE X 442 -11.184 -17.220 5.329 0.00 0.00 O

ATOM 6772 N VAL X 443 -8.991 -17.478 4.960 0.00 0.00 N

ATOM 6773 HN VAL X 443 -8.346 -17.467 4.204 0.00 0.00 H

ATOM 6774 CA VAL X 443 -8.489 -17.597 6.303 0.00 0.00 C

ATOM 6775 HA VAL X 443 -9.073 -18.338 6.823 0.00 0.00 H

ATOM 6776 CB VAL X 443 -6.956 -17.917 6.311 0.00 0.00 C

ATOM 6777 HB VAL X 443 -6.333 -17.084 5.910 0.00 0.00 H

ATOM 6778 CG1 VAL X 443 -6.464 -18.261 7.738 0.00 0.00 C

ATOM 6779 HG11 VAL X 443 -7.130 -19.065 8.112 0.00 0.00 H

ATOM 6780 HG12 VAL X 443 -6.379 -17.478 8.522 0.00 0.00 H

ATOM 6781 HG13 VAL X 443 -5.375 -18.473 7.689 0.00 0.00 H

ATOM 6782 CG2 VAL X 443 -6.788 -19.178 5.485 0.00 0.00 C

ATOM 6783 HG21 VAL X 443 -7.003 -18.937 4.417 0.00 0.00 H

ATOM 6784 HG22 VAL X 443 -7.312 -20.066 5.880 0.00 0.00 H

ATOM 6785 HG23 VAL X 443 -5.699 -19.425 5.449 0.00 0.00 H

ATOM 6786 C VAL X 443 -8.628 -16.297 7.009 0.00 0.00 C

ATOM 6787 O VAL X 443 -9.103 -16.238 8.142 0.00 0.00 O

ATOM 6788 N ILE X 444 -8.252 -15.213 6.302 0.00 0.00 N

ATOM 6789 HN ILE X 444 -7.961 -15.284 5.351 0.00 0.00 H

ATOM 6790 CA ILE X 444 -8.486 -13.798 6.717 0.00 0.00 C

ATOM 6791 HA ILE X 444 -7.983 -13.575 7.644 0.00 0.00 H

ATOM 6792 CB ILE X 444 -8.071 -12.768 5.605 0.00 0.00 C

ATOM 6793 HB ILE X 444 -8.714 -13.097 4.760 0.00 0.00 H

ATOM 6794 CG2 ILE X 444 -8.403 -11.361 5.919 0.00 0.00 C

ATOM 6795 HG21 ILE X 444 -8.215 -10.648 5.096 0.00 0.00 H

ATOM 6796 HG22 ILE X 444 -7.944 -10.984 6.861 0.00 0.00 H

ATOM 6797 HG23 ILE X 444 -9.487 -11.243 6.136 0.00 0.00 H

ATOM 6798 CG1 ILE X 444 -6.566 -13.048 5.254 0.00 0.00 C

ATOM 6799 HG11 ILE X 444 -6.369 -14.127 5.075 0.00 0.00 H

ATOM 6800 HG12 ILE X 444 -6.000 -12.645 6.125 0.00 0.00 H

ATOM 6801 CD ILE X 444 -6.186 -12.493 3.847 0.00 0.00 C

ATOM 6802 HD1 ILE X 444 -5.090 -12.586 3.751 0.00 0.00 H

ATOM 6803 HD2 ILE X 444 -6.496 -11.434 3.733 0.00 0.00 H

ATOM 6804 HD3 ILE X 444 -6.772 -13.018 3.062 0.00 0.00 H

ATOM 6805 C ILE X 444 -10.006 -13.495 7.055 0.00 0.00 C

ATOM 6806 O ILE X 444 -10.351 -12.960 8.111 0.00 0.00 O

ATOM 6807 N ASN X 445 -10.884 -13.952 6.153 0.00 0.00 N

ATOM 6808 HN ASN X 445 -10.520 -14.414 5.346 0.00 0.00 H

ATOM 6809 CA ASN X 445 -12.221 -13.672 6.140 0.00 0.00 C

ATOM 6810 HA ASN X 445 -12.594 -13.806 7.136 0.00 0.00 H

ATOM 6811 CB ASN X 445 -12.536 -12.180 5.674 0.00 0.00 C

ATOM 6812 HB1 ASN X 445 -12.424 -12.187 4.567 0.00 0.00 H

ATOM 6813 HB2 ASN X 445 -11.845 -11.500 6.208 0.00 0.00 H

ATOM 6814 CG ASN X 445 -13.981 -11.745 5.900 0.00 0.00 C

ATOM 6815 OD1 ASN X 445 -14.952 -12.479 5.984 0.00 0.00 O

ATOM 6816 ND2 ASN X 445 -14.055 -10.401 6.085 0.00 0.00 N

ATOM 6817 HD21 ASN X 445 -13.290 -9.756 6.015 0.00 0.00 H

ATOM 6818 HD22 ASN X 445 -14.892 -10.022 6.473 0.00 0.00 H

ATOM 6819 C ASN X 445 -12.877 -14.698 5.128 0.00 0.00 C

ATOM 6820 O ASN X 445 -12.692 -14.495 3.875 0.00 0.00 O

ATOM 6821 N TRP X 446 -13.684 -15.688 5.539 0.00 0.00 N

ATOM 6822 HN TRP X 446 -13.853 -15.922 6.494 0.00 0.00 H

ATOM 6823 CA TRP X 446 -14.311 -16.581 4.562 0.00 0.00 C

ATOM 6824 HA TRP X 446 -13.468 -16.877 3.958 0.00 0.00 H

ATOM 6825 CB TRP X 446 -14.798 -17.833 5.379 0.00 0.00 C

ATOM 6826 HB1 TRP X 446 -15.345 -17.412 6.249 0.00 0.00 H

ATOM 6827 HB2 TRP X 446 -13.972 -18.512 5.675 0.00 0.00 H

ATOM 6828 CG TRP X 446 -15.855 -18.744 4.689 0.00 0.00 C

ATOM 6829 CD1 TRP X 446 -17.228 -18.571 4.860 0.00 0.00 C

ATOM 6830 HD1 TRP X 446 -17.601 -17.796 5.524 0.00 0.00 H

ATOM 6831 NE1 TRP X 446 -17.978 -19.404 4.037 0.00 0.00 N

ATOM 6832 HE1 TRP X 446 -18.923 -19.623 4.120 0.00 0.00 H

ATOM 6833 CE2 TRP X 446 -17.005 -20.206 3.350 0.00 0.00 C

ATOM 6834 CD2 TRP X 446 -15.689 -19.732 3.663 0.00 0.00 C

ATOM 6835 CE3 TRP X 446 -14.583 -20.305 3.197 0.00 0.00 C

ATOM 6836 HE3 TRP X 446 -13.673 -19.940 3.635 0.00 0.00 H

ATOM 6837 CZ3 TRP X 446 -14.740 -21.432 2.458 0.00 0.00 C

ATOM 6838 HZ3 TRP X 446 -13.871 -21.968 2.095 0.00 0.00 H

ATOM 6839 CZ2 TRP X 446 -17.208 -21.328 2.525 0.00 0.00 C

ATOM 6840 HZ2 TRP X 446 -18.207 -21.713 2.371 0.00 0.00 H

ATOM 6841 CH2 TRP X 446 -16.006 -21.960 2.133 0.00 0.00 C

ATOM 6842 HH2 TRP X 446 -16.058 -22.930 1.668 0.00 0.00 H

ATOM 6843 C TRP X 446 -15.421 -16.022 3.729 0.00 0.00 C

ATOM 6844 O TRP X 446 -15.422 -16.171 2.488 0.00 0.00 O

ATOM 6845 N TRP X 447 -16.404 -15.231 4.295 0.00 0.00 N

ATOM 6846 HN TRP X 447 -16.470 -15.282 5.289 0.00 0.00 H

ATOM 6847 CA TRP X 447 -17.448 -14.704 3.521 0.00 0.00 C

ATOM 6848 HA TRP X 447 -17.990 -15.417 2.914 0.00 0.00 H

ATOM 6849 CB TRP X 447 -18.484 -14.015 4.491 0.00 0.00 C

ATOM 6850 HB1 TRP X 447 -19.231 -13.460 3.879 0.00 0.00 H

ATOM 6851 HB2 TRP X 447 -17.892 -13.263 5.050 0.00 0.00 H

ATOM 6852 CG TRP X 447 -19.182 -14.982 5.407 0.00 0.00 C

ATOM 6853 CD1 TRP X 447 -19.153 -14.833 6.771 0.00 0.00 C

ATOM 6854 HD1 TRP X 447 -18.719 -14.008 7.318 0.00 0.00 H

ATOM 6855 NE1 TRP X 447 -19.840 -15.901 7.442 0.00 0.00 N

ATOM 6856 HE1 TRP X 447 -20.052 -15.802 8.380 0.00 0.00 H

ATOM 6857 CE2 TRP X 447 -20.409 -16.683 6.464 0.00 0.00 C

ATOM 6858 CD2 TRP X 447 -19.953 -16.129 5.207 0.00 0.00 C

ATOM 6859 CE3 TRP X 447 -20.375 -16.810 4.077 0.00 0.00 C

ATOM 6860 HE3 TRP X 447 -20.136 -16.513 3.065 0.00 0.00 H

ATOM 6861 CZ3 TRP X 447 -21.237 -17.981 4.232 0.00 0.00 C

ATOM 6862 HZ3 TRP X 447 -21.682 -18.402 3.345 0.00 0.00 H

ATOM 6863 CZ2 TRP X 447 -21.348 -17.682 6.569 0.00 0.00 C

ATOM 6864 HZ2 TRP X 447 -21.813 -17.959 7.505 0.00 0.00 H

ATOM 6865 CH2 TRP X 447 -21.748 -18.340 5.410 0.00 0.00 C

ATOM 6866 HH2 TRP X 447 -22.380 -19.215 5.460 0.00 0.00 H

ATOM 6867 C TRP X 447 -16.937 -13.590 2.474 0.00 0.00 C

ATOM 6868 O TRP X 447 -17.477 -13.422 1.396 0.00 0.00 O

ATOM 6869 N ALA X 448 -15.992 -12.793 2.887 0.00 0.00 N

ATOM 6870 HN ALA X 448 -15.706 -13.054 3.813 0.00 0.00 H

ATOM 6871 CA ALA X 448 -15.382 -11.750 1.977 0.00 0.00 C

ATOM 6872 HA ALA X 448 -16.167 -11.064 1.715 0.00 0.00 H

ATOM 6873 CB ALA X 448 -14.299 -10.962 2.618 0.00 0.00 C

ATOM 6874 HB1 ALA X 448 -13.351 -11.535 2.739 0.00 0.00 H

ATOM 6875 HB2 ALA X 448 -14.714 -10.620 3.588 0.00 0.00 H

ATOM 6876 HB3 ALA X 448 -14.184 -10.023 2.034 0.00 0.00 H

ATOM 6877 C ALA X 448 -14.778 -12.344 0.722 0.00 0.00 C

ATOM 6878 O ALA X 448 -14.961 -11.844 -0.370 0.00 0.00 O

ATOM 6879 N ALA X 449 -14.009 -13.407 0.876 0.00 0.00 N

ATOM 6880 HN ALA X 449 -13.996 -13.868 1.765 0.00 0.00 H

ATOM 6881 CA ALA X 449 -13.190 -14.133 -0.085 0.00 0.00 C

ATOM 6882 HA ALA X 449 -12.759 -13.434 -0.790 0.00 0.00 H

ATOM 6883 CB ALA X 449 -12.190 -14.995 0.575 0.00 0.00 C

ATOM 6884 HB1 ALA X 449 -11.433 -14.358 1.089 0.00 0.00 H

ATOM 6885 HB2 ALA X 449 -11.645 -15.508 -0.249 0.00 0.00 H

ATOM 6886 HB3 ALA X 449 -12.684 -15.740 1.236 0.00 0.00 H

ATOM 6887 C ALA X 449 -14.243 -14.871 -0.957 0.00 0.00 C

ATOM 6888 O ALA X 449 -14.130 -15.008 -2.216 0.00 0.00 O

ATOM 6889 N LEU X 450 -15.332 -15.397 -0.295 0.00 0.00 N

ATOM 6890 HN LEU X 450 -15.304 -15.265 0.697 0.00 0.00 H

ATOM 6891 CA LEU X 450 -16.462 -15.886 -1.082 0.00 0.00 C

ATOM 6892 HA LEU X 450 -16.085 -16.627 -1.778 0.00 0.00 H

ATOM 6893 CB LEU X 450 -17.482 -16.594 -0.148 0.00 0.00 C

ATOM 6894 HB1 LEU X 450 -17.832 -15.842 0.581 0.00 0.00 H

ATOM 6895 HB2 LEU X 450 -16.954 -17.311 0.512 0.00 0.00 H

ATOM 6896 CG LEU X 450 -18.665 -17.273 -0.746 0.00 0.00 C

ATOM 6897 HG LEU X 450 -19.313 -16.485 -1.202 0.00 0.00 H

ATOM 6898 CD1 LEU X 450 -18.132 -18.335 -1.807 0.00 0.00 C

ATOM 6899 HD11 LEU X 450 -17.446 -19.056 -1.321 0.00 0.00 H

ATOM 6900 HD12 LEU X 450 -17.572 -17.834 -2.626 0.00 0.00 H

ATOM 6901 HD13 LEU X 450 -18.998 -18.977 -2.091 0.00 0.00 H

ATOM 6902 CD2 LEU X 450 -19.486 -17.990 0.210 0.00 0.00 C

ATOM 6903 HD21 LEU X 450 -18.870 -18.785 0.673 0.00 0.00 H

ATOM 6904 HD22 LEU X 450 -20.379 -18.293 -0.382 0.00 0.00 H

ATOM 6905 HD23 LEU X 450 -19.949 -17.364 1.015 0.00 0.00 H

ATOM 6906 C LEU X 450 -17.179 -14.835 -1.925 0.00 0.00 C

ATOM 6907 O LEU X 450 -17.537 -15.157 -3.123 0.00 0.00 O

ATOM 6908 N LEU X 451 -17.467 -13.600 -1.404 0.00 0.00 N

ATOM 6909 HN LEU X 451 -17.240 -13.437 -0.452 0.00 0.00 H

ATOM 6910 CA LEU X 451 -18.000 -12.551 -2.267 0.00 0.00 C

ATOM 6911 HA LEU X 451 -18.812 -13.054 -2.776 0.00 0.00 H

ATOM 6912 CB LEU X 451 -18.433 -11.392 -1.343 0.00 0.00 C

ATOM 6913 HB1 LEU X 451 -17.606 -10.896 -0.792 0.00 0.00 H

ATOM 6914 HB2 LEU X 451 -19.171 -11.782 -0.611 0.00 0.00 H

ATOM 6915 CG LEU X 451 -19.122 -10.223 -2.161 0.00 0.00 C

ATOM 6916 HG LEU X 451 -18.394 -9.749 -2.859 0.00 0.00 H

ATOM 6917 CD1 LEU X 451 -20.161 -10.718 -3.169 0.00 0.00 C

ATOM 6918 HD11 LEU X 451 -20.746 -9.853 -3.543 0.00 0.00 H

ATOM 6919 HD12 LEU X 451 -20.789 -11.434 -2.606 0.00 0.00 H

ATOM 6920 HD13 LEU X 451 -19.746 -11.209 -4.079 0.00 0.00 H

ATOM 6921 CD2 LEU X 451 -19.716 -9.208 -1.154 0.00 0.00 C

ATOM 6922 HD21 LEU X 451 -20.479 -9.646 -0.473 0.00 0.00 H

ATOM 6923 HD22 LEU X 451 -20.247 -8.359 -1.642 0.00 0.00 H

ATOM 6924 HD23 LEU X 451 -18.895 -8.684 -0.626 0.00 0.00 H

ATOM 6925 C LEU X 451 -16.949 -12.196 -3.366 0.00 0.00 C

ATOM 6926 O LEU X 451 -17.387 -11.803 -4.448 0.00 0.00 O

ATOM 6927 N THR X 452 -15.589 -12.226 -3.145 0.00 0.00 N

ATOM 6928 HN THR X 452 -15.282 -12.411 -2.212 0.00 0.00 H

ATOM 6929 CA THR X 452 -14.491 -11.989 -4.099 0.00 0.00 C

ATOM 6930 HA THR X 452 -14.776 -11.136 -4.691 0.00 0.00 H

ATOM 6931 CB THR X 452 -13.146 -11.957 -3.360 0.00 0.00 C

ATOM 6932 HB THR X 452 -12.990 -12.948 -2.884 0.00 0.00 H

ATOM 6933 OG1 THR X 452 -13.278 -11.039 -2.306 0.00 0.00 O

ATOM 6934 HG1 THR X 452 -13.896 -11.385 -1.655 0.00 0.00 H

ATOM 6935 CG2 THR X 452 -11.947 -11.504 -4.207 0.00 0.00 C

ATOM 6936 HG21 THR X 452 -11.868 -12.359 -4.898 0.00 0.00 H

ATOM 6937 HG22 THR X 452 -11.010 -11.354 -3.620 0.00 0.00 H

ATOM 6938 HG23 THR X 452 -12.239 -10.560 -4.715 0.00 0.00 H

ATOM 6939 C THR X 452 -14.503 -13.031 -5.181 0.00 0.00 C

ATOM 6940 O THR X 452 -14.639 -12.722 -6.305 0.00 0.00 O

ATOM 6941 N TYR X 453 -14.591 -14.327 -4.852 0.00 0.00 N

ATOM 6942 HN TYR X 453 -14.655 -14.574 -3.885 0.00 0.00 H

ATOM 6943 CA TYR X 453 -14.752 -15.370 -5.839 0.00 0.00 C

ATOM 6944 HA TYR X 453 -13.798 -15.410 -6.347 0.00 0.00 H

ATOM 6945 CB TYR X 453 -14.834 -16.812 -5.198 0.00 0.00 C

ATOM 6946 HB1 TYR X 453 -15.390 -16.796 -4.241 0.00 0.00 H

ATOM 6947 HB2 TYR X 453 -13.749 -17.062 -5.153 0.00 0.00 H

ATOM 6948 CG TYR X 453 -15.549 -17.778 -6.099 0.00 0.00 C

ATOM 6949 CD1 TYR X 453 -16.814 -18.175 -5.757 0.00 0.00 C

ATOM 6950 HD1 TYR X 453 -17.352 -17.707 -4.939 0.00 0.00 H

ATOM 6951 CE1 TYR X 453 -17.510 -19.162 -6.554 0.00 0.00 C

ATOM 6952 HE1 TYR X 453 -18.556 -19.364 -6.382 0.00 0.00 H

ATOM 6953 CZ TYR X 453 -16.861 -19.859 -7.626 0.00 0.00 C

ATOM 6954 OH TYR X 453 -17.578 -20.772 -8.490 0.00 0.00 O

ATOM 6955 HH TYR X 453 -18.405 -20.882 -8.009 0.00 0.00 H

ATOM 6956 CD2 TYR X 453 -14.992 -18.367 -7.259 0.00 0.00 C

ATOM 6957 HD2 TYR X 453 -13.944 -18.155 -7.370 0.00 0.00 H

ATOM 6958 CE2 TYR X 453 -15.569 -19.431 -7.967 0.00 0.00 C

ATOM 6959 HE2 TYR X 453 -15.040 -19.960 -8.741 0.00 0.00 H

ATOM 6960 C TYR X 453 -15.969 -15.102 -6.857 0.00 0.00 C

ATOM 6961 O TYR X 453 -15.866 -15.103 -8.055 0.00 0.00 O

ATOM 6962 N VAL X 454 -17.175 -14.821 -6.271 0.00 0.00 N

ATOM 6963 HN VAL X 454 -17.305 -14.954 -5.298 0.00 0.00 H

ATOM 6964 CA VAL X 454 -18.404 -14.497 -6.921 0.00 0.00 C

ATOM 6965 HA VAL X 454 -18.737 -15.385 -7.442 0.00 0.00 H

ATOM 6966 CB VAL X 454 -19.590 -14.299 -5.984 0.00 0.00 C

ATOM 6967 HB VAL X 454 -19.217 -13.735 -5.114 0.00 0.00 H

ATOM 6968 CG1 VAL X 454 -20.798 -13.513 -6.551 0.00 0.00 C

ATOM 6969 HG11 VAL X 454 -20.665 -12.490 -6.968 0.00 0.00 H

ATOM 6970 HG12 VAL X 454 -21.570 -13.375 -5.768 0.00 0.00 H

ATOM 6971 HG13 VAL X 454 -21.317 -14.102 -7.335 0.00 0.00 H

ATOM 6972 CG2 VAL X 454 -20.119 -15.620 -5.289 0.00 0.00 C

ATOM 6973 HG21 VAL X 454 -20.782 -15.327 -4.443 0.00 0.00 H

ATOM 6974 HG22 VAL X 454 -19.274 -16.273 -4.992 0.00 0.00 H

ATOM 6975 HG23 VAL X 454 -20.662 -16.287 -6.000 0.00 0.00 H

ATOM 6976 C VAL X 454 -18.366 -13.351 -7.910 0.00 0.00 C

ATOM 6977 O VAL X 454 -18.908 -13.465 -9.000 0.00 0.00 O

ATOM 6978 N ILE X 455 -17.737 -12.194 -7.485 0.00 0.00 N

ATOM 6979 HN ILE X 455 -17.293 -12.221 -6.587 0.00 0.00 H

ATOM 6980 CA ILE X 455 -17.506 -11.070 -8.431 0.00 0.00 C

ATOM 6981 HA ILE X 455 -18.446 -10.667 -8.762 0.00 0.00 H

ATOM 6982 CB ILE X 455 -16.969 -9.849 -7.627 0.00 0.00 C

ATOM 6983 HB ILE X 455 -16.230 -10.260 -6.904 0.00 0.00 H

ATOM 6984 CG2 ILE X 455 -16.348 -8.833 -8.605 0.00 0.00 C

ATOM 6985 HG21 ILE X 455 -17.072 -8.595 -9.425 0.00 0.00 H

ATOM 6986 HG22 ILE X 455 -15.482 -9.219 -9.180 0.00 0.00 H

ATOM 6987 HG23 ILE X 455 -16.176 -7.889 -8.043 0.00 0.00 H

ATOM 6988 CG1 ILE X 455 -18.089 -9.200 -6.804 0.00 0.00 C

ATOM 6989 HG11 ILE X 455 -18.333 -9.999 -6.072 0.00 0.00 H

ATOM 6990 HG12 ILE X 455 -18.983 -8.762 -7.284 0.00 0.00 H

ATOM 6991 CD ILE X 455 -17.617 -8.172 -5.773 0.00 0.00 C

ATOM 6992 HD1 ILE X 455 -18.467 -7.965 -5.083 0.00 0.00 H

ATOM 6993 HD2 ILE X 455 -17.276 -7.206 -6.203 0.00 0.00 H

ATOM 6994 HD3 ILE X 455 -16.758 -8.585 -5.208 0.00 0.00 H

ATOM 6995 C ILE X 455 -16.690 -11.435 -9.578 0.00 0.00 C

ATOM 6996 O ILE X 455 -17.116 -11.301 -10.742 0.00 0.00 O

ATOM 6997 N VAL X 456 -15.537 -12.000 -9.334 0.00 0.00 N

ATOM 6998 HN VAL X 456 -15.125 -12.245 -8.466 0.00 0.00 H

ATOM 6999 CA VAL X 456 -14.652 -12.548 -10.391 0.00 0.00 C

ATOM 7000 HA VAL X 456 -14.459 -11.797 -11.135 0.00 0.00 H

ATOM 7001 CB VAL X 456 -13.272 -12.974 -9.889 0.00 0.00 C

ATOM 7002 HB VAL X 456 -13.392 -13.580 -8.975 0.00 0.00 H

ATOM 7003 CG1 VAL X 456 -12.340 -13.733 -10.947 0.00 0.00 C

ATOM 7004 HG11 VAL X 456 -11.327 -13.894 -10.523 0.00 0.00 H

ATOM 7005 HG12 VAL X 456 -12.322 -13.321 -11.980 0.00 0.00 H

ATOM 7006 HG13 VAL X 456 -12.749 -14.756 -11.115 0.00 0.00 H

ATOM 7007 CG2 VAL X 456 -12.560 -11.750 -9.379 0.00 0.00 C

ATOM 7008 HG21 VAL X 456 -12.468 -11.139 -10.296 0.00 0.00 H

ATOM 7009 HG22 VAL X 456 -11.505 -11.911 -9.071 0.00 0.00 H

ATOM 7010 HG23 VAL X 456 -13.133 -11.114 -8.667 0.00 0.00 H

ATOM 7011 C VAL X 456 -15.235 -13.749 -11.262 0.00 0.00 C

ATOM 7012 O VAL X 456 -14.983 -13.845 -12.468 0.00 0.00 O

ATOM 7013 N LEU X 457 -16.057 -14.698 -10.663 0.00 0.00 N

ATOM 7014 HN LEU X 457 -16.153 -14.758 -9.672 0.00 0.00 H

ATOM 7015 CA LEU X 457 -16.705 -15.800 -11.387 0.00 0.00 C
[truncated: 211,773 more chars]
