# Supplementary figures and images for: The structural basis of function and regulation of neuronal cotransporters NKCC1 and KCC2
Source: Commun Biol. 2021 Feb 17;4:226. doi: 10.1038/s42003-021-01750-w (PMC7889885; doi:10.1038/s42003-021-01750-w)

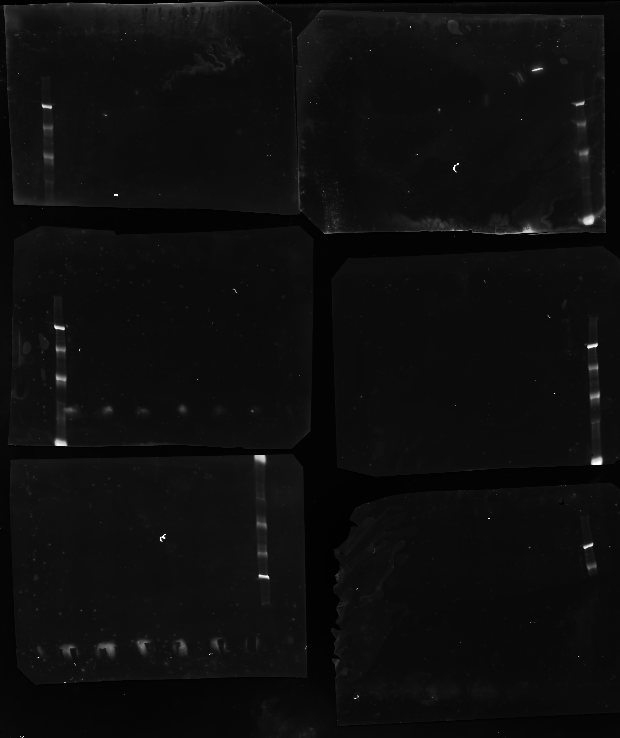

Supplement: Supplementary file 6 — Supplementary Data 3 [file 42003_2021_1750_MOESM6_ESM.zip › CuPhe crosslinking 700.TIF]

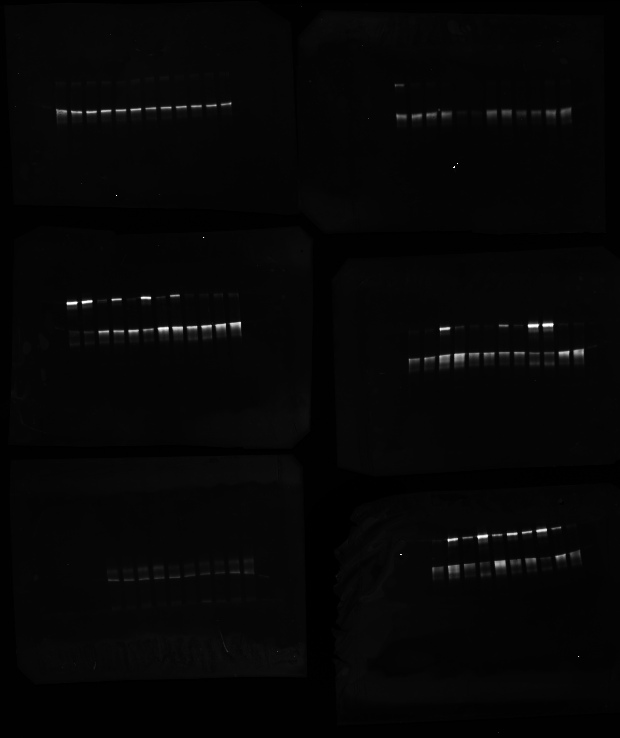

Supplement: Supplementary file 6 — Supplementary Data 3 [file 42003_2021_1750_MOESM6_ESM.zip › CuPhe crosslinking 800.TIF]

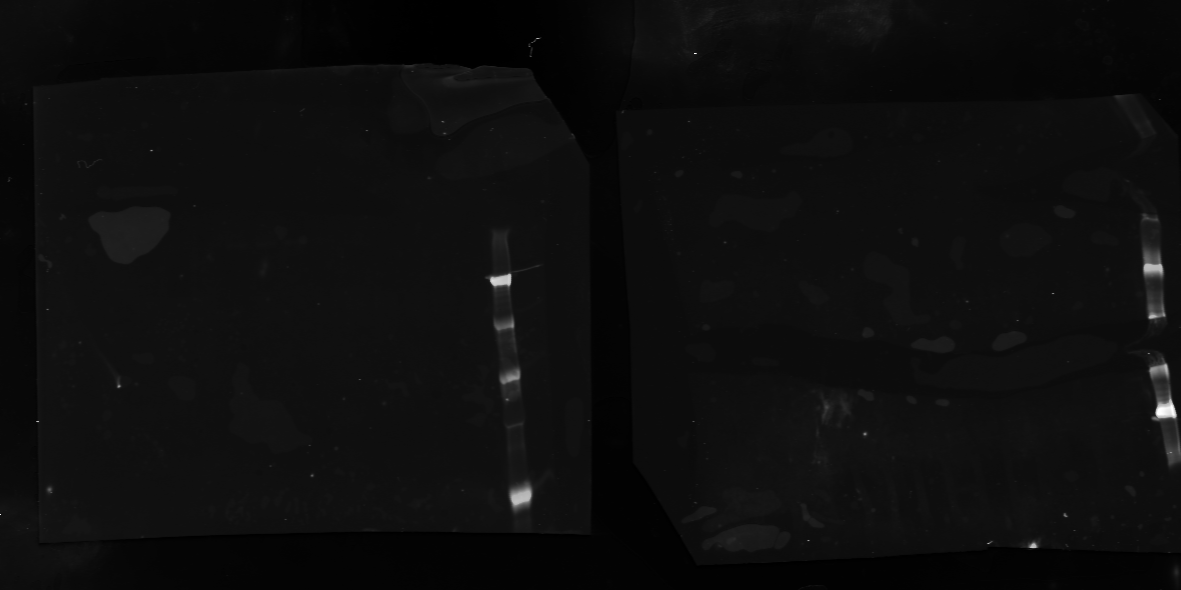

Supplement: Supplementary file 6 — Supplementary Data 3 [file 42003_2021_1750_MOESM6_ESM.zip › iodine crosslinking 700.TIF]

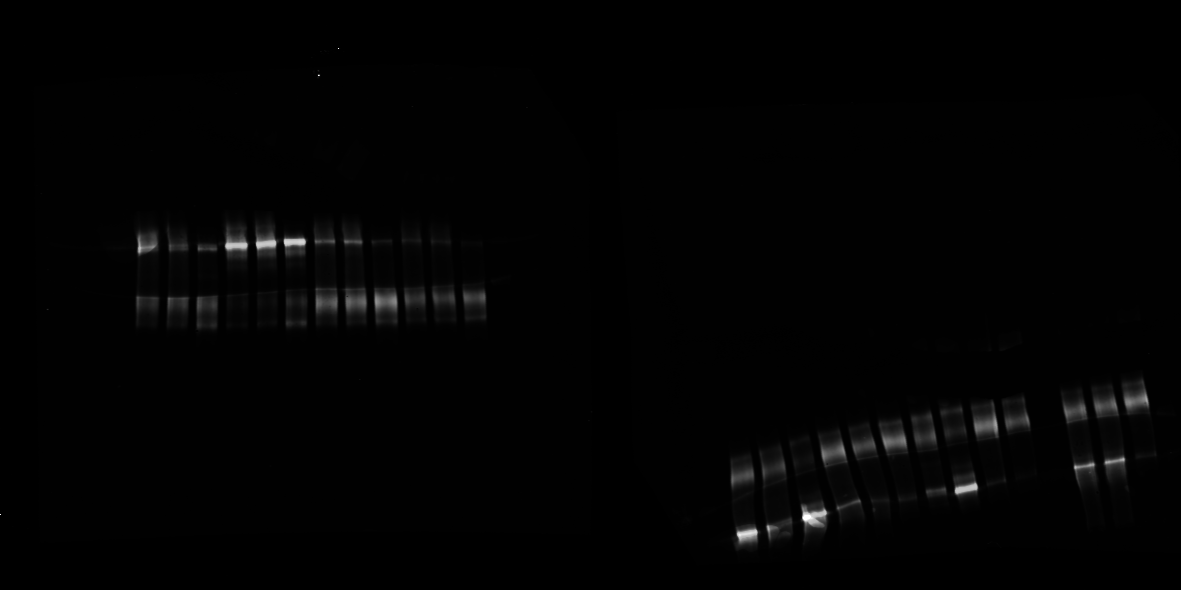

Supplement: Supplementary file 6 — Supplementary Data 3 [file 42003_2021_1750_MOESM6_ESM.zip › iodine crosslinking 800.TIF]

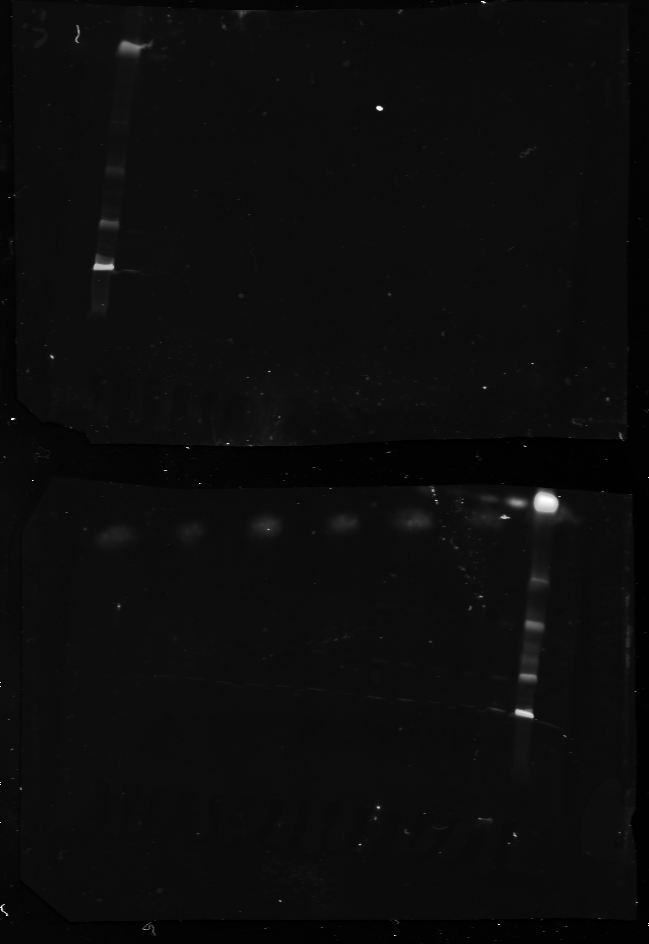

Supplement: Supplementary file 6 — Supplementary Data 3 [file 42003_2021_1750_MOESM6_ESM.zip › MTS crosslinking 700.TIF]

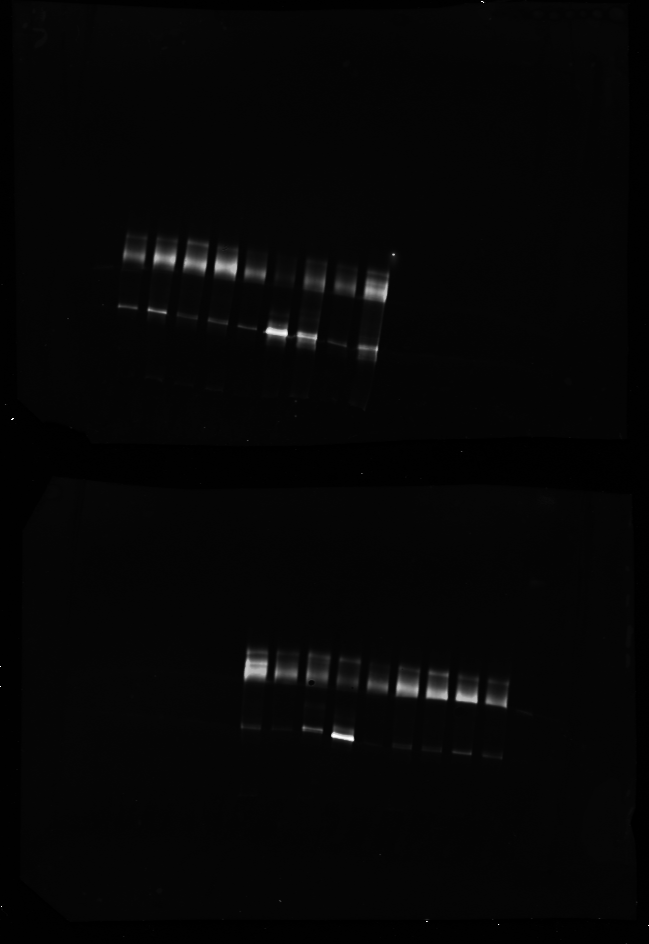

Supplement: Supplementary file 6 — Supplementary Data 3 [file 42003_2021_1750_MOESM6_ESM.zip › MTS crosslinking 800.TIF]
