# Supplementary material for: The structural basis of function and regulation of neuronal cotransporters NKCC1 and KCC2
Source: Commun Biol. 2021 Feb 17;4:226. doi: 10.1038/s42003-021-01750-w (PMC7889885; doi:10.1038/s42003-021-01750-w)
Supplement: Supplementary file 7 — Reporting Summary [file 42003_2021_1750_MOESM7_ESM.pdf]

nature research

Corresponding author(s): Maojun Yang

Last updated by author(s): Dec 14, 2020

Reporting Summary

Nature Research wishes to improve the reproducibility of the work that we publish. This form provides structure for consistency and transparency in reporting. For further information on Nature Research policies, see our Editorial Policies and the Editorial Policy Checklist.

Statistics

For all statistical analyses, confirm that the following items are present in the figure legend, table legend, main text, or Methods section.

n/a	Confirmed	
☐	☒	The exact sample size (*n*) for each experimental group/condition, given as a discrete number and unit of measurement
☐	☒	A statement on whether measurements were taken from distinct samples or whether the same sample was measured repeatedly
☐	☒	The statistical test(s) used AND whether they are one- or two-sided<br>*Only common tests should be described solely by name; describe more complex techniques in the Methods section.*
☒	☐	A description of all covariates tested
☒	☐	A description of any assumptions or corrections, such as tests of normality and adjustment for multiple comparisons
☐	☒	A full description of the statistical parameters including central tendency (e.g. means) or other basic estimates (e.g. regression coefficient) AND variation (e.g. standard deviation) or associated estimates of uncertainty (e.g. confidence intervals)
☐	☒	For null hypothesis testing, the test statistic (e.g. *F*, *t*, *r*) with confidence intervals, effect sizes, degrees of freedom and *P* value noted<br>*Give P values as exact values whenever suitable.*
☒	☐	For Bayesian analysis, information on the choice of priors and Markov chain Monte Carlo settings
☒	☐	For hierarchical and complex designs, identification of the appropriate level for tests and full reporting of outcomes
☒	☐	Estimates of effect sizes (e.g. Cohen's *d*, Pearson's *r*), indicating how they were calculated

Our web collection on statistics for biologists contains articles on many of the points above.

Software and code

Policy information about availability of computer code

Data collection	SerialEM3.6 for EM data collection.
Data analysis	For data processing: RELION 3.0, MotionCorr2.1, CTFFIND4, GCTF1.06, PHENIX1.14-3374, ResMap1.1.4,<br>For atomic model refinement and analysis: Coot0.8.9, PHENIX1.14-3374, MolProbity (part of PHENIX package),<br>For figure preparation: PyMol2.1.0, Chimera1.12.

For manuscripts utilizing custom algorithms or software that are central to the research but not yet described in published literature, software must be made available to editors and reviewers. We strongly encourage code deposition in a community repository (e.g. GitHub). See the Nature Research guidelines for submitting code & software for further information.

Data

Policy information about availability of data

All manuscripts must include a data availability statement. This statement should provide the following information, where applicable:

- Accession codes, unique identifiers, or web links for publicly available datasets
- A list of figures that have associated raw data
- A description of any restrictions on data availability

The 3D cryo-electron microscopy density maps and the coordinates of atomic models have been deposited in the Electron Microscopy Data Bank (EMDB) and in the Protein Data Bank (PDB)with accession code EMD-30542 and PDB 7D10 for human NKCC1, and EMD-30543 and PDB 7D14 for mouse KCC2.

Field-specific reporting

Please select the one below that is the best fit for your research. If you are not sure, read the appropriate sections before making your selection.

☒ Life sciences ☐ Behavioural & social sciences ☐ Ecological, evolutionary & environmental sciences

For a reference copy of the document with all sections, see nature.com/documents/nr-reporting-summary-flat.pdf

Life sciences study design

All studies must disclose on these points even when the disclosure is negative.

Sample size	No sample-size calculation was performed. Sample sizes are listed for each experiment that is statistically analyzed.
Data exclusions	No data were excluded from the analyses.
Replication	All attempts at replication were successful.
Randomization	Randomization is not relevant to the majority of experiments of this study, because protein samples are not required to be allocated into experimental groups in the biochemical studies. Randomization was used only in cryo-EM image processing and structure refinement.
Blinding	Not applicable. No human research participants are involved and no experiments were analyzed subjectively in this study.

Reporting for specific materials, systems and methods

We require information from authors about some types of materials, experimental systems and methods used in many studies. Here, indicate whether each material, system or method listed is relevant to your study. If you are not sure if a list item applies to your research, read the appropriate section before selecting a response.

Materials & experimental systems

n/a	Involved in the study
☐	☒ Antibodies
☐	☒ Eukaryotic cell lines
☒	☐ Palaeontology and archaeology
☒	☐ Animals and other organisms
☒	☐ Human research participants
☒	☐ Clinical data
☒	☐ Dual use research of concern

Methods

n/a	Involved in the study
☒	☐ ChIP-seq
☒	☐ Flow cytometry
☒	☐ MRI-based neuroimaging

Antibodies

Antibodies used	T4 monoclonal antibody, Developmental Studies Hybridoma Bank, Forbush lab origin, 1995. Li-Cor IRDye® 800CW Goat anti-Rabbit IgG Secondary Antibody.
Validation	The T4 antibody has been in use since 1995 by laboratories worldwide and used in hundreds of papers. It has been widely validated (originally in the Forbush lab) for use in Western blotting, the application in this paper. This validation is referenced in the Developmental Studies Hybridoma Bank information. The Li-Cor secondary is validated in Li-Cor material.

Eukaryotic cell lines

Policy information about cell lines

Cell line source(s)	FreeStyle™ 293-F Cells; and HEK-293 cells ATTC.
Authentication	Invitrogen, Catalog number, R790-07. ATTC Catalog CRL-1573.
Mycoplasma contamination	The cell lines tested negative for mycoplasma contamination.
Commonly misidentified lines (See ICLAC register)	No commonly misidentified cell lines were used.
